# Anti-Infective Properties, Cytotoxicity, and *In Silico* ADME Parameters of Novel 4′-(Piperazin-1-yl)benzanilides

**DOI:** 10.3390/ph18071004

**Published:** 2025-07-03

**Authors:** Theresa Hermann, Sarah Harzl, Robin Wallner, Elke Prettner, Eva-Maria Pferschy-Wenzig, Monica Cal, Pascal Mäser, Robert Weis

**Affiliations:** 1 Pharmaceutical Chemistry, Institute of Pharmaceutical Sciences, University of Graz, Schubertstraße 1, 8010 Graz, Austria; harzl.sarah@gmail.com (S.H.); wallner.rob@gmail.com (R.W.); robert.weis@uni-graz.at (R.W.); 2 Pharmacognosy, Institute of Pharmaceutical Sciences, University of Graz, Beethovenstraße 8, 8010 Graz, Austria; eva-maria.wenzig@uni-graz.at; 3 Swiss Tropical and Public Health Institute, Kreuzstraße 2, CH-4123 Allschwil, Switzerland; monica.cal@swisstph.ch (M.C.); pascal.maeser@swisstph.ch (P.M.); 4 Faculty of Science, University of Basel, Petersplatz 1, CH-4003 Basel, Switzerland

**Keywords:** malaria, *Plasmodium falciparum*, bacteria, ESKAPE, antibiotics, ADMET

## Abstract

**Background**: The benzamide **MMV030666** from MMV’s Malaria Box Project, the starting point of herein presented study, was initially tested against various *Plasmodium falciparum* strains as well as Gram-positive and Gram-negative bacteria. It exhibits multi-stage antiplasmodial potencies and lacks resistance development. **Methods**: The favorable structural features from previous series were kept while the influence of the *N*-Boc-piperazinyl substituent per se, as well as its ring position and its replacement by various heteroaromatic rings, was evaluated. Thus, this paper describes the preparation of the **MMV030666**-derived 4′-(piperazin-1-yl)benzanilides for the first time, exhibiting broad-spectrum activity not only against plasmodia but also various bacterial strains. **Results**: A series of insightful structure–activity relationships were determined. Furthermore, pharmacokinetic and physicochemical parameters of the new compounds were determined experimentally or *in silico*. Drug-likeliness according to Lipinski’s rules was calculated as well. **Conclusions**: A diarylthioether derivative of the lead compound was promisingly active against *P. falciparum* and exhibited broad-spectrum antibacterial activity against Gram-positive as well as Gram-negative bacteria. It is considered for testing against multi-resistant bacterial strains and *in vivo* studies.

## 1. Introduction

The 2024 World Malaria Report once again highlights the ever-increasing threat caused by multi-resistant pathogens of the *Plasmodium* species. The current gold standard for malaria treatment are artemisinin-based combination therapies (ACTs), consisting of a short-lived but fast acting artemisinin derivative combined with one or two partner drugs with longer elimination half-lives. However, the once very successful malaria treatment is now exposed to an inexorable rise in resistance development. Partial artemisinin resistance and high levels of treatment failure due to several *PfKelch13* mutations in *Plasmodium falciparum*, the most prominent malaria pathogen, have been detected in the WHO African region as well as in the South-East Asian and the Western Pacific regions [1,2,3].

Additionally, scientists face severe challenges when it comes to antimalarial vaccine development. Multiple possible targets within the complex life cycle of plasmodia, for example, make the exploration of effective vaccines inducing long-lasting protective immunity a rather difficult task to undertake. Currently, there are more than a dozen whole-parasite and sub-unit vaccine candidates in various stages of clinical development; however, none of them exhibit protective immunity and the durability of their effect is limited [4,5].

And as if that was not enough, the COVID-19 pandemic has put an additional halt to the fight against malaria. Over the last few years, disruption to health systems and supply chains as well as less financial support has led to a significant increase in malaria cases and deaths, with children under the age of five and pregnant women still carrying the majority of the burden [1].

**Figure 1:**
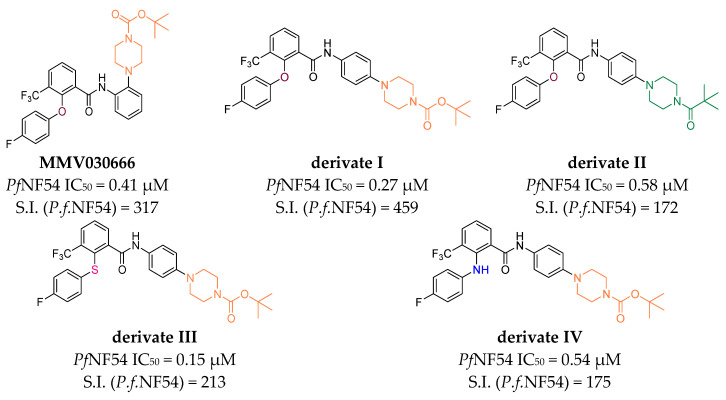
MMV’s benzanilide **MMV030666** and its most promising derivates **I**–**IV** from our latest studies [12,13].

Similarly to malaria, we are on the verge of being unable to treat infectious diseases caused by bacteria. In May of 2024, the WHO published an update on the critical priority ESKAPE pathogens (*Enterococcus faecium*, *Staphylococcus aureus*, *Klebsiella pneumoniae*, *Acinetobacter baumannii*, *Pseudomonas aeruginosa*, and *Enterobacteriaceae*) with resistance to front-line antibiotics such as beta-lactams, macrolides, or carbapenems. Antimicrobial research and development have not kept pace with the rapid resistance evolution, partly due to insufficient funding, resulting in an alarming gap in the ability to successfully treat bacterial infections. Of particular concern are carbapenem-resistant *Acinetobacter baumannii* as well as carbapenem- and third generation cephalosporin-resistant *Enterobacteriaceae* [6,7]. The Swiss non-profit organization Medicines for Malaria Venture (MMV) made it their business to support researchers in developing novel antimalarial agents. In 2016, they published the MMV Malaria Box, a compound collection of 400 diverse drug- and probe-like compounds. They are the result of a huge screening campaign and possess activities against various strains of *Plasmodia*. The starting point of the herein presented newly synthesized compounds, the benzamide **MMV030666** from MMV’s Malaria Box Project, was initially tested against various *P. falciparum* strains as well as bacteria [8,9,10,11]. Within our latest studies, we have focused on optimizing the antiplasmodial properties of compounds while maintaining the low cytotoxicity. We reported the positive impact of 4-fluoro-substituted 2-phenoxy-, 2-phenylsulfanyl- or 2-anilino groups as well as an electron withdrawing group in position 3 of the benzanilide. Furthermore, we observed the particular importance of the ring position of the piperazinyl substituent, whereby *para*-substituted compounds showed the highest antiplasmodial activity (Figure 1) [12,13].

This paper describes the preparation of 4′-(piperazin-1-yl)benzanilides with broad-spectrum activity not only against plasmodia but also against various Gram-positive and Gram-negative bacteria from the WHO ESKAPE panel. We maintained the favorable structural features while evaluating the influence of the *N*-*Boc*-piperazinyl substituent per se as well as its ring position and its replacement by various heteroaromatic rings. Furthermore, tertiary amides were prepared in order to investigate the importance of the amide hydrogen.

## 2. Results and Discussion

### 2.1. Chemistry

**Figure 2:**
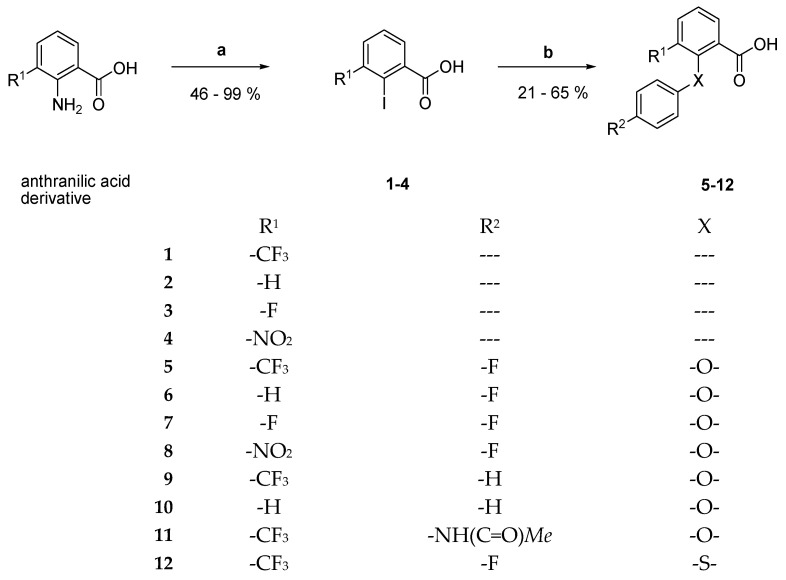
Preparation of compounds **5**–**12**. Reagents and conditions: (a) (1) H_2_SO_4_ 30%, dimethyl sulfoxide (DMSO), 0 °C, for 5 min; (2) NaNO_2_, rt, for 2 h; (3) KI, H_2_O, rt, for 1 h; (4) KI, H_2_O, rt, for 1 h; (b) corresponding phenol or benzenethiol, Cu, CuI, 1,8-diazabicyclo[5.4.0]undec-7-ene (DBU), dry pyridine, dry dimethylformamide (DMF), 160 °C, for 2 h (compounds **5**–**10**) or 48 h (compounds **11** and **12**).

The new derivatives were prepared by firstly synthesizing the corresponding carboxylic acids and aniline derivatives with subsequent amide bond formation. Synthesis of the benzoic acid derivatives was started by reaction of the respective anthranilic acid with sodium nitrite under acidic conditions, giving the corresponding diazonium salt. A Sandmeyer-like reaction of the latter with an aqueous solution of potassium iodide gave the desired 2-iodobenzoic acid derivatives **1**, **2**, **3**, and **4** as brownish amorphous solids in moderate to high yields [14]. In the course of a copper-catalyzed Ullmann-type ether synthesis, the prepared 2-iodobenzoic acids **1**, **2**, **3**, and **4** were coupled with 4-fluorophenol, phenol, *N*-(4-hydroxyphenyl)acetamide, or 4-fluorothiophenol, respectively, whereby variously substituted diarylethers (compounds **5**–**11**) and a diarylthioether (compound **12**) were obtained (Figure 2) [15].

**Figure 3:**
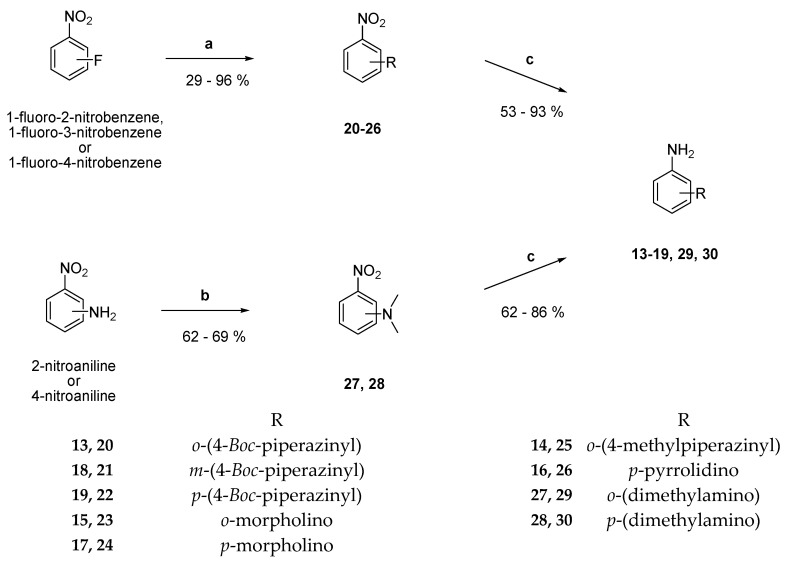
Preparation of aniline derivatives **13**–**19**, **29**, and **30**. Reagents and conditions: (a) anhydrous K_2_CO_3_, corresponding *N*-heterocycle, dry DMSO, 80 °C, for 72 h (compounds **20** and **22**–**26**), or anhydrous K_2_CO_3_, *N*-*Boc*-piperazine, dry DMSO, 120 °C, for 120 h (compound **21**); (b) (1) NaH, dry THF, rt, for 5 min; (2) methyl iodide, dry THF, rt, for 24 h (compounds **27** and **28**); (c) 15% (m/m) palladium on activated carbon, H_2_, dry methanol, rt, for 24 h.

Synthesis of the desired 2-, 3-, and 4-substituted derivatives of aniline **13**–**19** was started by treating 1-fluoro-2-nitrobenzene, 1-fluoro-3-nitrobenzene, or 1-fluoro-4-nitrobenzene, respectively, with the corresponding secondary amines *N*-*Boc*-piperazine (**20**–**22**), morpholine (**23**, **24**), *N*-methylpiperazine (**25**), or pyrrolidine (**26**) and potassium carbonate in dry dimethyl sulfate in the course of a nucleophilic aromatic substitution. Thereby, the nitrobenzyl-derivatives **20**–**26** were obtained [16]. To prepare the *N*,*N*-dimethyl-2-nitroaniline **27** and the *N*,*N*-dimethyl-4-nitroaniline **28**, 2- and 4-nitroaniline were treated with a 60% dispersion of NaH in mineral oil, followed by addition of methyl iodide in dry tetrahydrofuran (THF) [17]. Subsequent reduction of the nitro group of compounds **20**–**28** with palladium in dry methanol in an atmosphere of hydrogen gave the desired derivatives of aniline **13**–**19**, **29**, and **30** (Figure 3) [18]. Efficient reduction of the nitro group was detected in the ^1^H-NMR spectrum: the aromatic protons shifted to lower frequencies and an additional signal for amino protons appeared.

**Figure 4:**
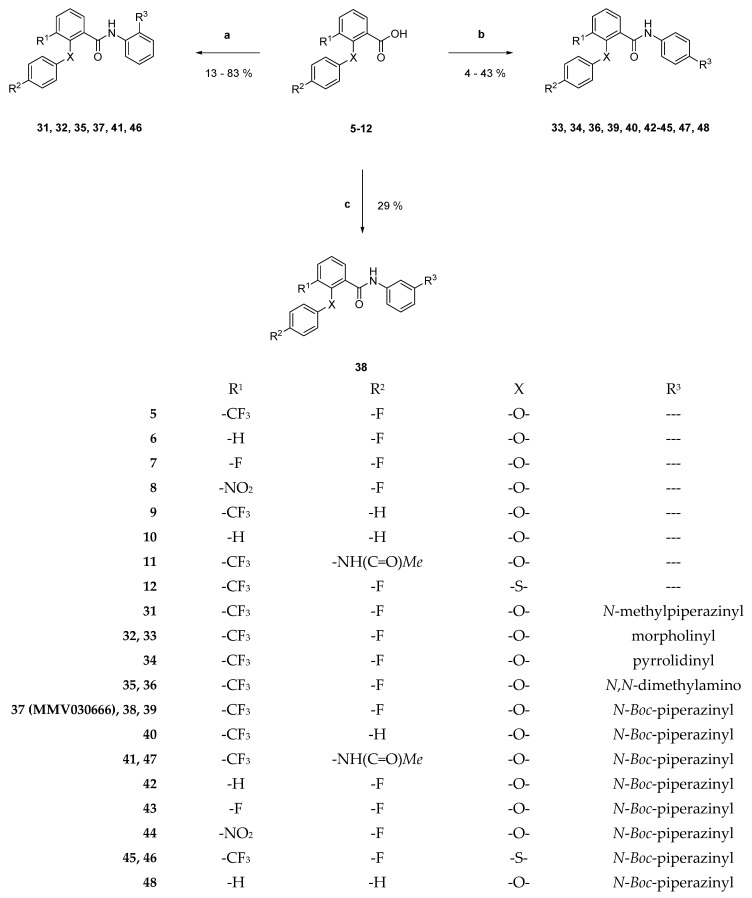
Preparation of compounds **31**–**48**. Reagents and conditions: (a–c) (1) corresponding aniline derivative, dry dichloromethane, 0 °C, for 5 min; (2) 2-chloro-*N*-methylpyridin-1-ium iodide, DIPEA, rt, for 24 h (compounds **31**–**45** and **48**) or 48 h (compounds **46** and **47**).

Amide bond formation between the benzoic acid and aniline derivatives was accomplished using a combination of 2-chloro-*N*-methylpyridin-1-ium iodide (Mukaiyama reagent) and diisopropylethylamine (DIPEA) in dry dichloromethane (Figure 4) [19]. Successful amide synthesis was detected via ^1^H-NMR spectroscopy, whereby the -NH_2_ signal disappeared and an amide hydrogen peak appeared at higher frequencies.

**Figure 5:**
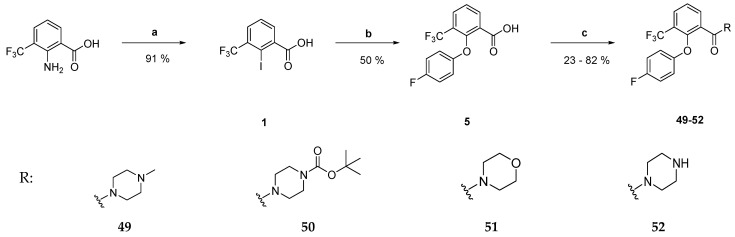
Preparation of compounds **49**–**52**. Reagents and conditions: (a) (1) H_2_SO_4_ 30%, DMSO, 0 °C, for 5 min; (2) NaNO_2_, rt, for 2 h; (3) KI, H_2_O, rt, for 1 h; (4) KI, H_2_O, rt, for 1 h; (b) 4-fluorophenol, Cu, CuI, DBU, dry pyridine, dry DMF, 160 °C, for 2 h; (c) (1) corresponding secondary amine, dry DMF, 0 °C, for 5 min; (2) potassium Oxyma-B, 0 °C, for 5 min; (3) EDC × HCl, rt, for 72 h (compounds **49**–**51**) or (1) *N*-*Boc*-piperazine, dry DMF, 0 °C, for 5 min; (2) potassium Oxyma-B, 0 °C, for 5 min; (3) EDC × HCl, rt, for 72 h; (4) dry dichloromethane, 0 °C, for 5 min; (5) trifluoroacetic acid, dry dichloromethane, rt, for 24 h (compound **52**).

The tertiary amides **49**, **50**, and **51** were prepared by coupling the carboxylic acid derivative **5** with the respective secondary amines *N*-methylpiperazine (**49**), morpholine (**51**), or *N*-*Boc*-piperazine (**50**). The coupling reagents Potassium Oxyma-B and 1-Ethyl-3-(3-dimethylaminopropyl)carbodiimide × HCl (EDC × HCl) ensured efficient amide bond formation [20]. The *N*-*Boc*-group of **50** was cleaved using trifluoroacetic acid in dichloromethane. Thereby, compound **52** was obtained (Figure 5) [21].

**Figure 6:**
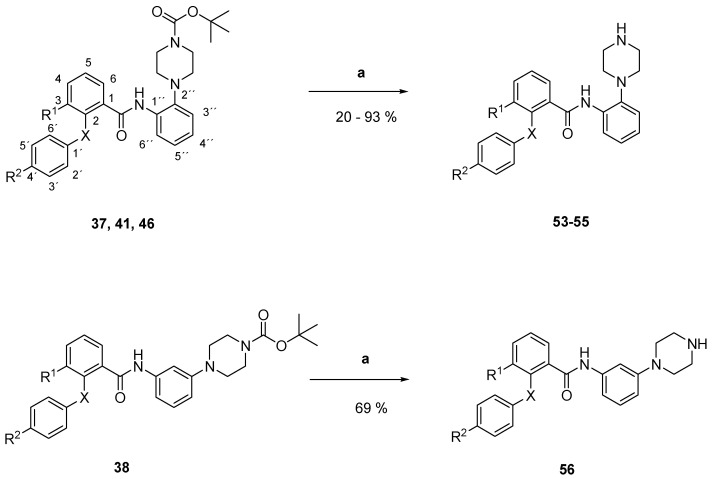

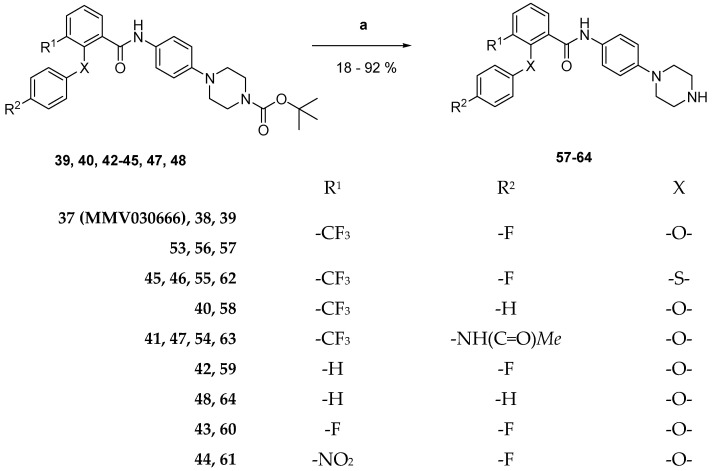
Preparation of compounds **53**–**64**. Reagents and conditions: (a) (1) dry dichloromethane, 0 °C, for 5 min; (2) trifluoroacetic acid, dry dichloromethane, rt, for 24 h.

In order to evaluate the importance of the bulky *N*-*tert*-Butoxy-group for the biological activity of compounds **37**–**48** the latter was again cleaved with trifluoroacetic acid in dry dichloromethane, yielding the mono substituted piperazine derivatives **53**–**64** (Figure 6) [21]. Successful cleavage was detected in the ^1^H-NMR spectra: the singlet peak of the *tert*-Butyl-group at 2.5 ppm disappeared and an -NH-peak appeared at higher frequencies.

**Figure 7:**
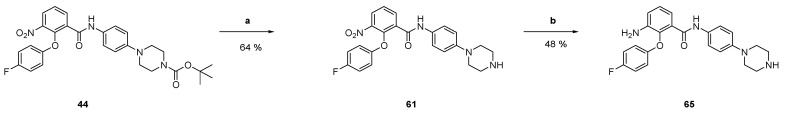
Preparation of compound **65**. Reagents and conditions: (a) (1) dry dichloromethane, 0 °C, for 5 min; (2) trifluoroacetic acid, dry dichloromethane, rt, for 24 h; (b) 15% (m/m) palladium on activated carbon, H_2_, dry methanol, rt, for 24 h.

The influence of an amino group compared to an electron-withdrawing nitro group on the anti-infective activity was assessed by preparing compound **65**. Therefore, the *N*-*Boc*-group of compound **44** was cleaved using trifluoroacetic acid in dry dichloromethane to obtain compound **61**, which was then followed by reduction of the nitro group with palladium in an atmosphere of hydrogen (Figure 7) [18,21].

**Figure 8:**
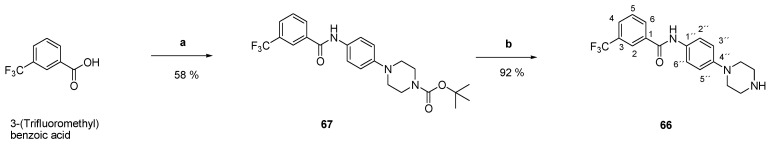
Preparation of compound **66**. Reagents and conditions: (a) aniline derivative **19**, dry dichloromethane, 0 °C, for 5 min; (2) 2-chloro-*N*-methylpyridin-1-ium iodide, DIPEA, rt, for 24 h; (b) (1) dry dichloromethane, 0 °C, for 5 min; (2) trifluoroacetic acid, dry dichloromethane, rt, for 24 h.

Moreover, the impact of the 2-aryloxy substituent on antiplasmodial activity was investigated. Therefore, compound **66** was prepared starting from 3-(trifluoromethyl)benzoic acid, which was subsequently coupled with the aniline derivative **19** with the help of 2-chloro-*N*-methylpyridin-1-ium iodide and DIPEA to obtain the amide **67**. Its *N*-*Boc*-group was cleaved using trifluoroacetic acid in dry dichloromethane (Figure 8) [19,21].

### 2.2. Biological Activity and Cytotoxicity

All newly synthesized compounds were tested for their antiplasmodial activity using the chloroquine-sensitive strain *Plasmodium falciparum* NF54. Cytotoxicity was determined using rat skeletal myofibroblasts (L-6 cells) as well as human hepatocarcinoma cells (HepG2 cells). As standards, chloroquine, podophyllotoxin, and doxorubicin were used. Results obtained are summarized in Table 1.

The tertiary amides **49**–**52** exhibit by far the lowest antiplasmodial activities (*Pf*NF54 IC_50_ = 23.2–81.1 µM), strongly indicating the importance of the amide hydrogen for the biological efficacy. All other newly prepared compounds are benzanilides with an unsubstituted amide hydrogen. The majority of them possess 4-fluorophenoxy and 3-trifluoromethyl groups in ring positions 2 and 3 of the benzamide core as well as a basic substituent at the aniline moiety. As was to be expected from previous studies, the ring position of the latter has an obvious impact on the antiplasmodial activity of compounds. The 3′-piperazinyl-substituted **56** exhibits the lowest activity against *Pf*NF54 (IC_50_ = 5.45 µM) compared to its *para-* and *ortho*-analogs **57** (*Pf*NF54 IC_50_ = 3.15 µM) and **53** (*Pf*NF54 IC_50_ = 1.04 µM). Further substitution of the piperazinyl ring by a 4-methyl group gave **31** (*Pf*NF54 IC_50_ = 14.0 µM), which is distinctly less active than **53**. Similarly, replacement of the piperazinyl by a dimethylamino (**36** (*Pf*NF54 IC_50_ = 7.58 µM) and **35** (*Pf*NF54 IC_50_ = 4.40 µM)) or a morpholino substituent (**33** (*Pf*NF54 IC_50_ = 6.81 µM) and **32** (*Pf*NF54 IC_50_ = 3.04 µM)) decreased the antiplasmodial activities in comparison to **57** and **53**, respectively. However, the 4-pyrrolidino analog **34** (*Pf*NF54 IC_50_ = 1.68 µM) is significantly more active than **57**. Likewise, changes in the substitution pattern of the benzamide core had a detectable effect on the antiplasmodial activity. Replacement of the 3-trifluoromethyl group of **57** gave its less active 3-fluoro **60** (*Pf*NF54 IC_50_ = 4.70 µM), 3-nitro **61** (*Pf*NF54 IC_50_ = 4.10 µM), and 3-amino **65** (*Pf*NF54 IC_50_ = 7.00 µM) analogs, whereas the 3-unsubstituted compound **59** (*Pf*NF54 IC_50_ = 2.04 µM) is more active. Replacement of the 2-(4-fluorophenoxy) substituent of **57** and **53** by a 2-(4-acetamidophenoxy) group gave compounds **63** (*Pf*NF54 IC_50_ = 15.7 µM) and **54** (*Pf*NF54 IC_50_ = 11.1 µM), which are among the least active of the benzanilides. The 2-phenoxy **58** (*Pf*NF54 IC_50_ = 3.81 µM) and its 2-unsubstituted analog **66** (*Pf*NF54 IC_50_ = 2.60 µM) show activity similar to **57** as well as the 3-unsubstituted 2-phenoxy derivative **64** (*Pf*NF54 IC_50_ = 2.33 µM). Significantly improved activities were observed for the 2-(4-fluorophenyl)sulfanyl derivatives **62** (*Pf*NF54 IC_50_ = 1.66 µM) and **55** (*Pf*NF54 IC_50_ = 0.69 µM) of **57** and **53**, respectively. Cytotoxicity against L-6 cells is for the most part consistent with values obtained against HepG2 cells. Compounds with high cytotoxicity in L-6 cells are also in most cases toxic to human cells. Among the more active compounds the 2′-morpholino substituted benzanilide **32** (*Pf*NF54 IC_50_ = 3.04 µM) does not show significant toxicity against neither rat skeletal myofibroblasts (IC_50_ = 161 µM) nor human hepatocarcinoma cells (IC_50_ = >100 µM). Its 4′-pyrrolidino analog **34** shows higher activity against *P. falciparum* NF54 (IC_50_ = 1.68 µM), very low L-6 (IC_50_ = 185 µM), and moderate HepG2 cytotoxicity (IC_50_ = 39.8 µM), resulting in the most promising compound out of this series of derivatives.

Due to initial studies and the fact that some of the Malaria Box compounds also exhibit antibacterial activity, selected benzanilides **55**–**62**, **64**, and **65**, as well as the tertiary amides **49**, **51**, and **52**, were further evaluated for their *in vitro* activity against a selection of pathogens from the ESKAPE panel (*Escherichia coli*, *Staphylococcus aureus*, *Klebsiella pneumoniae*, *Acinetobacter baumannii*, and *Pseudomonas aeruginosa*) as well as *Bacillus subtilis*, *Serratia marcescens*, and *Micrococcus luteus*. Minimal inhibitory concentration (MIC) and minimal bactericidal concentration (MBC) were determined following the latest EUCAST guidelines on antimicrobial susceptibility testing. Results obtained are summarized in Table 2, whereby the most promising ones are highlighted in green and moderate ones in yellow.

The analyzed tertiary amides **49**, **51**, and **52**, as well as the benzanilides **56**–**62**, **64**, and **65**, exhibit overall rather high MIC and MBC values of ≥128 µM against the Gram-negative strains *P. aeruginosa*, *E. coli*, *S. marcescens*, *A. baumannii*, and *K. pneumoniae*, which could very well be due to the low cell wall permeability of the compounds. A different picture emerges when looking at the Gram-positive strains, especially *M. luteus*. While removing the *N*-*Boc*-group results in a significant loss of antiplasmodial activity, the antibacterial properties improve. Regarding the 3′-piperazinylbenzanilide **56** and the 4′-piperazinylbenzanilide **57**, as well as the 2-(4-fluorophenoxy)-4′-(piperazin-1-yl)benzanilide **59**, its 3-fluorophenoxy analog **60**, and the 2-phenoxy-4′-(piperazin-1-yl)benzanilide **64**, promising antibacterial activity could be observed (MIC = 16–64 µg/mL; MBC = 16–64 µg/mL). The latter (**64**) also exhibits encouraging MIC and MBC values of 32 µg/mL and 64 µg/mL, respectively, against *S. aureus*. Excitingly, the 2-[(4-Fluorophenyl)sulfanyl]-*N*-[2-(piperazin-1-yl)phenyl]-3-(trifluoromethyl)benzamide **55** shows broad-spectrum antibacterial and bactericidal activity against Gram-negative *A. baumannii* and *E. coli* (MIC = 32–64 µg/mL; MBC = 64 µg/mL), as well as Gram-positive *M. luteus*, *B. subtilis*, and *S. aureus* (MIC = 16–64 µg/mL, MBC = 16–64 µg/mL).

### 2.3. Physicochemical and Pharmacokinetic Parameters

In addition to the anti-infective activity and cytotoxicity of compounds **31**–**36** and **49**–**66**, their log *p* and log D_7.4_ values were calculated *in silico*. Log *p* values ranged from 3.26 to 6.10 and log D_7.4_ values ranged from 1.96 to 6.10. Among the compounds with considerable anti-infective properties, the 2-(4-fluorophenoxy)-4′-(piperazin-1-yl)benzanilide **59** and the 2-(4-fluorophenoxy)-2′-(piperazin-1-yl)-3-(trifluoromethyl)benzanilide **53** exhibit the lowest log *p* (4.28–5.16) and log D_7.4_ (2.79–3.70) values. Furthermore, ligand efficiency, an important parameter in early drug development, was determined. Ligand efficiency is defined by the free binding energy of a compound divided by its number of heavy atoms (HA) [22]. The calculated values ranged from 0.178 to 0.306 kcal/mol/HA. Among the more active compounds, the benzanilide **59** again showed the highest LE value of 0.269 kcal/mol/HA. To complete the dataset for Lipinski’s rule of five, the number of hydrogen-bond donors and acceptors was determined. Compounds exhibit up to three HBDs and a maximum of five HBAs. With a molecular weight below 500 g/mol, all newly prepared benzanilides comply with Lipinski’s advice for the preparation of drug-like molecules.

Furthermore, passive permeability of compounds through semipermeable membranes, for example, the blood–brain-barrier, was determined via PAMPA and was detectable for all compounds except for **31**, **34**–**36**, and **63** due to insufficient solubility and/or excessive mass retention. Results are summarized in Table 3 above. Permeability is defined using caffeine (Pe = 8.00 × 10^−6^ cm/s) and hydrochlorothiazide (Pe = 0.09 × 10^−6^ cm/s) as standards. The diarylthioethers **55** and **62** with promising antiplasmodial activity also exhibit encouraging permeability of 4.41 and 6.51 × 10^−6^ cm/s, respectively. The benzanilide **59** with not only antiplasmodial but also detectable antibacterial activity showed the by far highest permeability of 14.23 × 10^−6^ cm/s.

CYPlebrity from the Kirchmair group at the University of Vienna is part of the New E-Resource for Drug Discovery (NERDD), a collection of machine learning models, and it was used to predict whether the newly synthesized compounds inhibit human Cytochrom P450 enzymes (CYP1A2, 2C9, 2C19, 2D6, and 3A4) essential for the phase I liver metabolism of xenobiotics and, if so, how severely [23]. Strong enzyme inhibition would result in an increased risk when combining the oral application of these compounds with other drugs. Results obtained are summarized in Table 4.

Compounds are overall predicted to have significant impact on CYP1A2, CYP2C9, and CYP2C19, with inhibition rates between 27 and 70%. As for CYP3A4, which is responsible for the metabolism of most xenobiotics, the overall predicted enzyme inhibition by the newly prepared compounds is considerably lower (31–46%). Promisingly, CYP2D6, the second most important phase I enzyme, is predicted to be distinctly less influenced by this series of benzamides (15–33%).

## 3. Materials and Methods

### 3.1. Instrumentation and Chemicals

Melting points were obtained using an Electrothermal IA 9200 melting point apparatus (Fisher Scientific, Birmingham, UK). IR spectra were acquired using a Bruker Alpha Platinum AT FTIR spectrometer (Bruker, Ettlingen, Germany) (preparation of KBr disks), and the frequencies are reported in cm^−1^. For HRMS, a Q Exactive Hybrid Quadrupole-Orbitrap mass spectrometer (Thermo Fisher Scientific, Waltham, MA, USA) run by Thermo Q Exactive 2.9 (Thermo Fisher Scientific, Waltham, MA, USA) and Thermo Xcalibur^TM^ Software Version 4.4 (Thermo Fisher Scientific, Waltham, MA, USA) and a Micromass Tofspec 3E spectrometer (MALDI) and a GCT-Premier, Waters, Milford, MA, USA (EI, 70 eV) were used. The structures of all newly synthesized derivatives were determined by one- and two-dimensional NMR spectroscopy using a Varian UnityInova 400 MHz or a Bruker Avance Neo 400 MHz spectrometer, 5 mm tubes, and TMS as internal standard. Shifts in ^1^H NMR (400 MHz) and ^13^C NMR (100 MHz) are reported in ppm. ^1^H- and ^13^C-resonances were assigned using ^1^H,^1^H- and ^1^H,^13^C-correlation spectra and are numbered as given in Figure 6 and Figure 8. Signal multiplicities are abbreviated as follows: br, broad; d, doublet; dd, doublet of doublets; m, multiplet; q, quartet; s, singlet; t, triplet; and td, triplet of doublets.

Materials: thin layer chromatography (TLC): TLC plates silica gel 60 F_254_ (Merck, Darmstadt, Germany); column chromatography (CC): silica gel 60 (Merck 70–230 mesh, pore diameter 60 Å), flash silica gel (VWR 230–400 mesh, pore diameter 60 Å or Merck 230–400 mesh, pore diameter 60 Å), and aluminum oxide basic (Merck); PAMPA: 96-well pre-coated Corning Gentest PAMPA plate system (Corning, Glendale, AZ, USA), 96-well UV-star Microplates (Greiner Bio-One, Kremsmünster, Austria), and a SpectraMax M3 UV plate-reader (Molecular Devices, San Jose, CA, USA). FTIR and HRMS as well as ^1^H-NMR and ^13^C-NMR spectra of compounds **31**–**36**, **47**, and **49**–**67** are available in the Supplementary Materials (Figures S1–S26).

### 3.2. Syntheses

#### 3.2.1. General Procedure for the Preparation of 2-iodobenzoic Acids **1**–**4** (Figure 2)

The corresponding anthranilic acid (6.00 mmol) was dissolved in DMSO (11 mL) and the solution was cooled to 0 °C in an ice bath. Upon adding 11 mL of 30% aq sulfuric acid (H_2_SO_4_), the reaction mixture was stirred at 0 °C for 5 min. After that, the ice bath was removed, and sodium nitrite (NaNO_2_) (13.28 mmol) was added. The reaction mixture was stirred at room temperature for 2 h. Subsequently, a solution of potassium iodide (KI) (10.92 mmol) in 5 mL of demineralized water was added dropwise with a syringe via a septum. The reaction mixture was stirred for another hour before adding a second portion of KI (6.00 mmol) dissolved in 3 mL of aqua demin. After stirring for 1 h at ambient temperature, 50 mL of ethyl acetate was added. The aqueous and organic phases were separated. The organic phase was washed with aqua demin and brine, dried over anhydrous sodium sulfate, filtered, and the solvent was evaporated in vacuo. The respective residues were purified by recrystallization from aqua demin.

2-Iodo-3-(trifluoromethyl)benzoic acid (**1**): Reaction of 2-amino-3-(trifluoromethyl)benzoic acid (2.11 g (10.33 mmol)) dissolved in DMSO (17 mL) and 30% aq H_2_SO_4_ (17 mL) with NaNO_2_ (1.54 g (22.35 mmol)) and KI (4.74 g (28.54 mmol)) in aqua deminaqua demin (17 mL) gave the raw benzoic acid derivative. Purification by recrystallization from aqua demin (10 mL) gave compound **1** as a brownish solid (2.97 g (91%)). m.P. 134 °C. NMR data were in accordance with the literature data [24].

2-Iodobenzoic acid (**2**): Reaction of anthranilic acid (831 mg (6.06 mmol)) dissolved in DMSO (11 mL) and 30% aq H_2_SO_4_ (11 mL) with NaNO_2_ (921 mg (13.35 mmol)) and KI (2.83 g (17.02 mmol)) in aqua demin (8 mL) gave the raw iodobenzoic acid. It was purified by recrystallization from aqua demin (7 mL), giving compound **2** as a brown solid (1.48 g (99%)). m.P. 160 °C. NMR data were in accordance with the literature data [25].

3-Fluoro-2-iodobenzoic acid (**3**): The reaction of 2-amino-3-fluorobenzoic acid (313 mg (2.02 mmol)) dissolved in DMSO (4 mL) and 30% aq H_2_SO_4_ (4 mL) with NaNO_2_ (309 mg (4.48 mmol)) and KI (939 mg (5.66 mmol)) in aqua demin (3 mL) yielded the raw product. It was purified by recrystallization from aqua demin (4 mL), giving compound **3** as a brown solid (245 mg (46%)). m.P. 153 °C. NMR data were in accordance with the literature data [26].

2-Iodo-3-nitrobenzoic acid (**4**): Reaction of 2-amino-3-nitrobenzoic acid (558 mg (3.06 mmol)) dissolved in DMSO (6 mL) and 30% aq H_2_SO_4_ (6 mL) with NaNO_2_ (460 mg (6.67 mmol)) and KI (1.42 g (8.55 mmol)) in aqua demin (4 mL) gave the raw product. Purification by recrystallization from aqua demin (5 mL) yielded compound **4** as a brown solid (799 mg (89%)). m.P. 207 °C. NMR data were in accordance with literature data [27].

#### 3.2.2. General Procedure for the Preparation of Diarylethers and **5**–**11** and Diarylthioether **12** (Figure 2)

The corresponding 2-iodobenzoic acid derivative **1**–**4** (4.00 mmol) was dissolved in dry DMF. The respective phenol or benzenethiol (4.20 mmol), copper (Cu) (0.53 mmol), copper (I) iodide (CuI) (0.18 mmol), DBU (12.00 mmol), and dry pyridine (0.80 mmol) were added in that order. The reaction mixture was refluxed at 160 °C for 2–48 h. After completion, the mixture was cooled to room temperature and acidified with 2*N* HCl to a pH of 1. Equal amounts of ice and dichloromethane were added. The organic and aqueous phases were separated. The aqueous phase was extracted three times with dichloromethane. The organic phases were combined, washed with aqua demin and brine, dried over anhydrous sodium sulfate, and filtered. The solvent was evaporated in vacuo giving the crude products, which were subsequently purified by column chromatography.

2-(4-Fluorophenoxy)-3-(trifluoromethyl)benzoic acid (**5**): Reaction of compound **1** (1499 mg (4.74 mmol)) with 4-fluorophenol (561 mg (4.98 mmol)), copper (40 mg (0.62 mmol)), copper (I) iodide (43 mg (0.23 mmol)), DBU (2167 mg (14.23 mmol)), and dry pyridine (75 mg (0.95 mmol)) in dry DMF (38 mL) gave the crude diarylether. It was purified by column chromatography (silica gel, CH_2_Cl_2_/*Me*OH/AcOH 149:1:1) followed by recrystallization from dichloromethane, yielding compound **5** as a colorless solid (711 mg (50%)). m.P. 143 °C. NMR data were in accordance with the literature data [12].

2-(4-Fluorophenoxy)benzoic acid (**6**): Reaction of compound **2** (688 mg (2.77 mmol)) with 4-fluorophenol (334 mg (2.98 mmol)), copper (31 mg (0.49 mmol)), copper (I) iodide (37 mg (0.19 mmol)), DBU (1267 mg (8.32 mmol)), and dry pyridine (44 mg (0.56 mmol)) in dry DMF (23 mL) gave the crude product. It was purified by column chromatography (flash silica gel, CH/(EtAc/EtOH/AcOH) 9:1 (3:1:0.08)), yielding compound **6** as a colorless amorphous solid (386 mg (60%)). NMR data were in accordance with the literature data [27].

3-Fluoro-2-(4-fluorophenoxy)benzoic acid (**7**): Reaction of compound **3** (595 mg (2.24 mmol)) with 4-fluorophenol (263 mg (2.35 mmol)), copper (19 mg (0.30 mmol)), copper (I) iodide (19 mg (0.10 mmol)), DBU (1023 mg (6.72 mmol)), and dry pyridine (36 mg (0.45 mmol)) in dry DMF (18 mL) gave the crude product. Purification by column chromatography (silica gel, CH_2_Cl_2_/*Me*OH/AcOH 149:1:1) yielded compound **7** as a yellow amorphous solid (240 mg (43%)). NMR data were in accordance with the literature data [13].

2-(4-Fluorophenoxy)-3-nitrobenzoic acid (**8**): Reaction of compound **4** (2464 mg (8.41 mmol)) with 4-fluorophenol (1000 mg (8.92 mmol)), copper (83 mg (1.31 mmol)), copper (I) iodide (76 mg (0.40 mmol)), DBU (3841 mg (25.23 mmol)), and dry pyridine (132 mg (1.67 mmol)) in dry DMF (68 mL) gave the crude product. It was purified by column chromatography (silica gel, CH_2_Cl_2_/*Me*OH/AcOH 149:1:1), yielding compound **8** as a light-orange amorphous solid (1515 mg (65%)). NMR data were in accordance with the literature data [13].

2-Phenoxy-3-(trifluoromethyl)benzoic acid (**9**): Reaction of compound **1** (627 mg (5.15 mmol)) with phenol (509 mg (5.41 mmol)), copper (49 mg (0.77 mmol)), copper (I) iodide (54 mg (0.28 mmol)), DBU (2353 mg (15.45 mmol)), and dry pyridine (71 mg (0.90 mmol)) in dry DMF yielded the crude product. It was purified by column chromatography (silica gel, CH_2_Cl_2_/propan-2-ol/NH_3_ cc. 8:9:2). The obtained salt was dissolved in aqua demin (10 mL) and acidified with 2*N* HCl to a pH of 1. The aqueous phase was extracted with dichloromethane. The organic phase was dried over anhydrous sodium sulfate, filtered, and the solvent evaporated in vacuo, giving compound **9** as a light-brown amorphous solid (538 mg (37%)). NMR data were in accordance with the literature data [12].

2-Phenoxybenzoic acid (**10**): Reaction of compound **2** (992 mg (4.00 mmol)) with phenol (395 mg (4.20 mmol)), copper (34 mg (0.53 mmol)), copper (I) iodide (34 mg (0.18 mmol)), DBU (1827 mg (12.00 mmol)), and dry pyridine (63 mg (0.80 mmol)) in dry DMF gave the crude product. Purification by column chromatography (silica gel, CH/EtAc/EtOH/AcOH 50:9:4:0.25) yielded compound **10** as an amorphous colorless solid (554 mg (65%)). NMR data were in accordance with the literature data [27].

2-(4-Acetamidophenoxy)-3-(trifluoromethyl)benzoic acid (**11**): Reaction of compound **1** (1273 mg (4.03 mmol)) with *N*-(4-hydroxyphenyl)acetamide (645 mg (4.27 mmol)), copper (35 mg (0.55 mmol)), copper (I) iodide (43 mg (0.25 mmol)), DBU (1827 mg (12.00 mmol)), and dry pyridine (63 mg (0.80 mmol)) in dry DMF (30 mL) for 48 h gave the crude diarylether. It was purified by column chromatography (silica gel, CH_2_Cl_2_/EtOH/AcOH 9:1:0.1), giving compound **11** as a pale-yellow amorphous solid (438 mg (32%)). NMR data were in accordance with the literature data [12].

2-[(4-Fluorophenyl)sulfanyl]-3-(trifluoromethyl)benzoic acid (**12**): Reaction of compound **1** (1901 mg (6.02 mmol)) with 4-fluorobenzene-1-thiol (806 mg (6.29 mmol)), copper (76 mg (1.20 mmol)), copper (I) iodide (60 mg (0.32 mmol)), DBU (2740 mg (17.99 mmol)), and dry pyridine (94 mg (1.19 mmol)) in dry DMF (48 mL) for 24 h yielded the crude product. It was purified by column chromatography (flash silica gel, CH_2_Cl_2_/*Me*OH/AcOH 149:1:1), giving compound **12** as a light-brown amorphous solid (199 mg (21%)). NMR data were in accordance with the literature data [13].

#### 3.2.3. General Procedure for the Preparation of (Nitrophenyl)piperazines **20**–**26** (Figure 3)

Anhydrous potassium carbonate (14.00 mmol) and the respective fluoro-nitrobenzene (7.00 mmol) were suspended in dry DMSO. The corresponding *N*-heterocycle (14.00 mmol) was added, and the reaction mixture was refluxed to 80–120 °C for 72–120 h. After completion, the mixture was cooled to 0 °C in an ice bath, diluted with ethyl acetate (30 mL), and acidified with 2*N* HCl to a pH of 1. The aqueous and organic phases were separated. The aqueous phase was extracted three times with ethyl acetate. The organic phases were combined, dried over anhydrous sodium sulfate, filtered, and the solvent evaporated in vacuo, yielding the crude products that were either purified by column chromatography or used without further purification.

*tert*-Butyl-4-(2-nitrophenyl)piperazine-1-carboxylate (**20**): Compound **20** was prepared by refluxing anhydrous potassium carbonate (1960 mg (14.20 mmol)), *N*-*Boc*-piperazine (2640 mg (14.20 mmol)), and 1-fluoro-2-nitrobenzene (1000 mg (7.20 mmol)) in dry DMSO (40 mL). The product was obtained as an orange oil (2073 mg (95%)), which was used without further purification. NMR data were in accordance with the literature data [18].

*tert*-Butyl-4-(3-nitrophenyl)piperazine-1-carboxylate (**21**): Refluxing anhydrous potassium carbonate (1940 mg (14.04 mmol)), *N*-*Boc*-piperazine (2609 mg (14.00 mmol)), and 1-fluoro-3-nitrobenzene (988 mg (7.00 mmol)) in dry DMSO (40 mL) for 120 h at 120 °C gave the crude product. It was purified by column chromatography (silica gel, CH/EtAc 4:1), yielding compound **21** as an orange amorphous solid (624 mg (29%)). NMR data were in accordance with the literature data [28].

*tert*-Butyl-4-(4-nitrophenyl)piperazine-1-carboxylate (**22**): Compound **22** was prepared by refluxing anhydrous potassium carbonate (1937 mg (14.02 mmol)), *N*-*Boc*-piperazine (2689 mg (14.44 mmol)), and 1-fluoro-4-nitrobenzene (988 mg (7.00 mmol)) in dry DMSO (40 mL). The product **22** was obtained as an orange solid (2065 mg, (96%)), which was used without further purification. NMR data were in accordance with the literature data [29].

4-(2-Nitrophenyl)morpholine (**23**): Refluxing a suspension of anhydrous potassium carbonate (967 mg (7.00 mmol)), morpholine (610 mg (7.00 mmol)), and 1-fluoro-2-nitrobenzene (494 mg (3.50 mmol)) in dry DMSO (20 mL) gave compound **23** as an orange oil (554 mg (76%)), which was used without further purification. NMR data were in accordance with the literature data [30].

1-(4-Nitrophenyl)pyrrolidine (**24**): Refluxing a suspension of anhydrous potassium carbonate (967 mg (7.00 mmol)), pyrrolidine (498 mg (7.00 mmol)), and 1-fluoro-4-nitrobenzene (494 mg (3.50 mmol)) in dry DMSO (20 mL) yielded compound **24** as an orange amorphous solid (636 mg (95%)), which was used without further purification. NMR data were in accordance with the literature data [31].

1-Methyl-4-(2-nitrophenyl)piperazine (**25**): Reaction of anhydrous potassium carbonate (989 mg (7.18 mmol), *N*-methyl-piperazine (701 mg (7.00 mmol)), and 1-fluoro-2-nitrobenzene (494 mg (3.50 mmol)) in dry DMSO (20 mL) gave compound **25** as an orange oil (635 mg (82%)), which was used without further purification. NMR data were in accordance with the literature data [32].

4-(4-Nitrophenyl)morpholine (**26**): Reaction of anhydrous potassium carbonate (967 mg (7.00 mmol)), morpholine (610 mg (7.00 mmol)), and 1-fluoro-4-nitrobenzene (494 mg (3.50 mmol)) in dry DMSO (20 mL) gave compound **26** as an orange amorphous solid (700 mg (96%)), which was used without further purification. NMR data were in accordance with the literature data [33].

#### 3.2.4. General Procedure for the Preparation of **27** and **28** (Figure 3)

A 60% dispersion of sodium hydride (NaH) in mineral oil was dissolved in dry THF. The respective nitroaniline was added slowly and the reaction mixture was stirred at room temperature for 5 min. Methyl iodide was dissolved in dry THF and added slowly with a syringe via a septum. The reaction mixture was stirred at room temperature overnight. After completion, aqua demin was added. The aqueous suspension was extracted twice with ethyl acetate. The organic phases were combined, dried over anhydrous sodium sulfate, filtered, and the solvent was evaporated in vacuo. The crude products were purified by column chromatography.

*N*,*N*-Dimethyl-2-nitroaniline (**27**): Reaction of NaH (60% dispersion in mineral oil, 370 mg (9.64 mmol)) with 2-nitroaniline (386 mg (2.79 mmol)) and methyl iodide (1976 mg (13.92 mmol)) in dry THF (20 mL) gave the crude product. It was purified by column chromatography (silica gel, CH/EtAc 9:1), yielding compound **27** as an orange amorphous solid (320 mg (69%)). NMR data were in accordance with the literature data [34].

*N*,*N*-Dimethyl-4-nitroaniline (**28**): Reaction of NaH (60% dispersion in mineral oil, 356 mg (9.30 mmol)) with 4-nitroaniline (400 mg (2.90 mmol)) and methyl iodide (1976 mg (13.92 mmol)) in dry THF (20 mL) gave the crude product. It was purified by column chromatography (silica gel, CH/EtAc 9:1), yielding compound **28** as an orange amorphous solid (299 mg (62%)). NMR data were in accordance with the literature data [31].

#### 3.2.5. General Procedure for the Reduction of Nitrobenzenes to Aniline Derivatives **13**–**19**, **29**, **30**, and **65** (Figure 3 and Figure 7)

The respective nitro-derivatives **20**–**28** (2.00 mmol) and 15% (m/m) palladium on activated carbon were dissolved in dry methanol (80–100 mL). Reduction of the nitro group was performed in an atmosphere of hydrogen using a parr apparatus at room temperature overnight. After completion, the reaction mixture was filtered and the solvent evaporated in vacuo. The crude products were either used without purification or purified by column chromatography.

*tert*-Butyl-4-(2-aminophenyl)piperazine-1-carboxylate (**13**): Reaction of compound **20** (3667 mg (11.93 mmol)) with PdC (560 mg) in dry methanol (100 mL) in an atmosphere of hydrogen yielded the crude product. It was purified by column chromatography (silica gel, CH_2_Cl_2_/*Me*OH 79:1), giving compound **13** as a light-brown amorphous solid (1754 mg (53%)). NMR data were in accordance with the literature data [18].

2-(4-Methylpiperazin-1-yl)aniline (**14**): Reaction of compound **25** (637 mg (2.88 mmol)) with PdC (116 mg) in dry methanol (100 mL) in an atmosphere of hydrogen yielded pure compound **14** as a colorless amorphous solid (457 mg (83%)). NMR data were in accordance with the literature data [32].

2-(4-Morpholin-4-yl)aniline (**15**): Reaction of compound **23** (552 mg (2.65 mmol)) with PdC (106 mg) in dry methanol (100 mL) in an atmosphere of hydrogen yielded pure compound **15** as a brown amorphous solid (331 mg (70%)). NMR data were in accordance with the literature data [35].

4-(Morpholin-4-yl)aniline (**16**): Reaction of compound **26** (697 mg (3.35 mmol)) with PdC (128 mg) in dry methanol (60 mL) in an atmosphere of hydrogen yielded pure compound **16** as a pale-pink amorphous solid (537 mg (90%)). NMR data were in accordance with the literature data [33].

4-(Pyrrolidin-1-yl)aniline (**17**): Reaction of compound **24** (637 mg (3.31 mmol)) with PdC (96 mg) in dry methanol (80 mL) in an atmosphere of hydrogen yielded pure compound **17** as a dark-red oil (456 mg (85%)). NMR data were in accordance with the literature data [31].

*tert*-Butyl-4-(3-aminophenyl)piperazine-1-carboxylate (**18**): Reaction of compound **21** (809 mg (2.63 mmol)) with PdC (125 mg) in dry methanol (100 mL) gave pure compound **18** as a dark-brown oil (657 mg (90%)). NMR data were in accordance with the literature data [36].

*tert*-Butyl-4-(4-aminophenyl)piperazine-1-carboxylate (**19**): Reaction of compound **22** (1982 mg (6.45 mmol)) with PdC (299 mg) in dry methanol (100 mL) gave pure compound **19** as a dark-red oil (1664 mg (93%)). NMR data were in accordance with the literature data [36].

*N*^1^,*N*^1^-Dimethylbenzene-1,2-diamine (**29**): Reaction of compound **27** (321 mg (1.93 mmol)) with PdC (60 mg) in dry methanol (100 mL) in an atmosphere of hydrogen yielded pure compound **29** as a dark-red oil (163 mg (62%)). NMR data were in accordance with the literature data [37].

*N*^1^,*N*^1^-Dimethylbenzene-1,4-diamine (**30**): Reaction of compound **28** (300 mg (1.81 mmol)) with PdC (49 mg) in dry methanol (80 mL) in an atmosphere of hydrogen yielded pure compound **30** as a pale-brown oil (212 mg (86%)). NMR data were in accordance with the literature data [31].

3-Amino-2-(4-fluorophenoxy)-*N*-[4-(piperazin-1-yl)phenyl]benzamide (**65**): Reaction of compound **61** (124 mg (0.28 mmol) with PdC (33 mg) in dry methanol (80 mL) yielded the crude product. It was purified by column chromatography (aluminum oxide basic, CH_2_Cl_2_/*Me*OH 29:1), yielding compound **65** as a pale-brown solid (57 mg (50%)). m.P. 150 °C; Rf = 0.163 (silica gel, CH_2_Cl_2_/*Me*OH 19:1); IR = 3374, 1651, 1517, 1498, 1472, 1321, 1235, 1198, 828, and 766; ^1^H NMR (CDCl_3_, 400 MHz) δ = 3.00–3.03 (m, 4H, N(CH_2_)_2_), 3.06–3.09 (m, 4H, N(CH_2_)_2_), 3.40 (br s, 2H, NH_2_), 6.83–6.87 (m, 2H, 3″-H, 5″-H), 6.87–6.90 (m, 2H, 2′-H, 6′-H), 6.95–6.99 (m, 3H, 3′-H, 4-H, 5′-H), 7.20 (t, *J* = 7.9 Hz, 1H, 5-H), 7.32–7.36 (m, 2H, 2″-H, 6″-H), 7.53 (dd, *J* = 7.8, 1.6 Hz, 1H, 6-H), and 8.69 (br s, 1H, NH); ^13^C NMR (CDCl_3_, 100 MHz) δ = 46.10 (N(CH_2_)_2_), 50.76 (N(CH_2_)_2_), 115.91 (d, *J* = 8.1 Hz, C-2′, C-6′), 116.58 (C-3″, C-5″), 116.69 (d, *J* = 22.7 Hz, C-3′, C-5′), 119.64 (C-4), 121.05 (C-6), 126.71 (C-5), 128.91 (C-1), 130.34 (C-1″), 138.04 (C-2), 140.04 (C-3), 148.91 (C-4″), 152.29 (d, *J* = 2.4 Hz, C-1′), 158.51 (d, *J* = 242 Hz, C-4′), and 162.65 (C=O); HRMS (ESI+) callculated for C_23_H_24_FN_4_O_2_^+^ [M+H^+^]: 407.1879 found: 407.1872.

#### 3.2.6. General Procedure for the Preparation of Tertiary Amides **49**–**51** (Figure 5)

The benzoic acid derivative **5** (1.00 mmol) and the respective secondary aliphatic amine (1.00 mmol) were dissolved in dry DMF (20 mL) and cooled in an ice bath to 0 °C. Potassium Oxyma-B (1.00 mmol) was added and the reaction mixture was stirred at 0 °C for 5 min. After that, EDC × HCl (1.00 mmol) was added and the reaction mixture was stirred at room temperature for 72 h. After completion, 20 mL of 2*N* NaOH was added. The aqueous suspension was extracted three times with dichloromethane. The organic phases were combined, washed three times with aqua demin, dried over anhydrous sodium sulfate, and filtered. The solvent was evaporated in vacuo yielding the crude products, which were then purified by column chromatography.

[2-(4-Fluorophenoxy)-3-(trifluoromethyl)phenyl](4-methylpiperazin-1-yl)methanon (**49**): Reaction of compound **5** (305 mg (1.02 mol)) with *N*-methylpiperazine (100 mg (1.00 mmol)), potassium Oxyma-B (234 mg (1.05 mmol), and EDC × HCl (2.14 mg (1.12 mmol)) in dry DMF (20 mL) gave the crude product. It was purified by column chromatography (silica gel, CH_2_Cl_2_/*Me*OH 29:1), yielding compound **49** as a yellow oil (153 mg (40%)). Rf = 0.313 (silica gel, CH_2_Cl_2_/*Me*OH 29:1); IR = 3439, 1641, 1502, 1449, 1327, 1295, 1220, 1138, 1001, 823, and 778; ^1^H NMR (CDCl_3_, 400 MHz) δ = 2.13–2.43 (m, 4H, 2 NCH_2_), 2.28 (s, 3H, CH_3_), 3.15–3.21 (m, 1H, NCH), 3.25 (t, *J* = 5.1 Hz, 2H, NCH_2_), 3.67–3.73 (m, 1H, NCH), 6.78–6.82 (m, 2H, 2′-H, 6′-H), 6.92–6.98 (m, 2H, 3′-H, 5′-H), 7.39 (t, *J* = 7.7, Hz, 1H, 5-H), 7.59 (dd, *J* = 7.7, 1.6 Hz, 1H, 6-H), and 7.77 (dd, *J* = 7.7, 1.6 Hz, 1H, 4-H); ^13^C NMR (CDCl_3_, 100 MHz) δ = 41.37 (CH_3_), 46.97 (NCH_2_), 46.75 (NCH_2_), 54.29 (NCH_2_), 54.84 (NCH_2_), 115.97 (d, *J* = 23.5 Hz, C-3′, C-5′), 117.44 (d, *J* = 8.2 Hz, C-2′, C-6′), 122.78 (q, *J* = 273 Hz, CF_3_), 124.74 (q, *J* = 31.5 Hz, C-3), 125.39 (C-5), 128.46 (q, *J* = 4.9 Hz, C-4), 131.54 (C-1), 133.40 (C-6), 149.28 (q, *J* = 1.9 Hz, C-2), 153.67 (d, *J* = 2.5 Hz, C-1′), 158.43 (d, *J* = 242 Hz, C-4′), and 164.86 (C=O); HRMS (ESI+) calculated for C_19_H_19_F_4_N_2_O_2_^+^ [M+H]^+^: 383.1383; found: 383.1373.

*tert*-Butyl-4-[2-(4-fluorophenoxy)-3-(trifluoromethyl)benzoyl]piperazine-1-carboxylate (**50**). Reaction of compound **5** (242 mg (0.81 mmol)) with *N*-*Boc*-piperazine (156 mg (0.84 mmol)), potassium Oxyma-B (187 mg (0.84 mmol)), and EDC × HCl (159 mg (0.83 mmol)) in dry DMF (16 mL) gave the crude product. It was purified by column chromatography (flash silica gel, CH_2_Cl_2_/acetonitrile 7:1), yielding compound **50** as a pale yellow solid (87 mg (23%)). m.P. 107 °C; Rf = 0.325 (silica gel, CH_2_Cl_2_/acetonitrile 7:1); IR = 3441, 1691, 1649, 1502, 1452, 1367, 1328, 1252, 1219, 1160, 1014, and 776; ^1^H NMR (CDCl_3_, 400 MHz) δ = 1.46 (s, 9H, (CH_3_)_3_), 3.18–3.26 (m, 4H, 2 NCH_2_), 3.29–3.36 (m, 2H, NCH_2_), 3.47–3.63 (m, 2H, NCH_2_), 6.77–6.80 (m, 2H, 2′-H, 6′-H), 6.92–6.98 (m, 2H, 3′-H, 5′-H), 7.41 (t, *J* = 7.8 Hz, 1H, 5-H), 7.61 (dd, *J* = 7.7, 1.6 Hz, 1H, 6-H), and 7.79 (dd, *J* = 7.9, 1.6 Hz, 1H, 4-H); ^13^C NMR (CDCl_3_, 100 MHz) δ = 28.33 ((CH_3_)_3_), 41.44 (NCH_2_), 43.27 ((NCH_2_)_2_), 46.69 (NCH_2_), 80.46 (C*Me*_3_), 116.04 (d, *J* = 23.6 Hz, C-3′, C-5′), 117.19 (d, *J* = 8.3 Hz, C-2′, C-6′), 122.69 (q, *J* = 273 Hz, CF_3_), 124.85 (q, *J* = 31.7 Hz, C-3), 125.65 (C-5), 128.73 (q, *J* = 4.9 Hz, C-4), 131.41 (C-1), 133.37 (C-6), 149.04 (br, C-2), 153.69 (d, *J* = 2.5 Hz, C-1′), 154.37 (N(C=O)O), 158.42 (d, *J* = 242 Hz, C-4′), and 165.18 ((C=O)N); HRMS (ESI+) calculated for C_23_H_25_F_4_N_2_O_4_^+^ [M+H]^+^: 469.1750; found: 469.1742; calculated for C_19_H_17_F_4_N_2_O_4_^+^ [M+H-C_4_H_8_]^+^: 413.1119; found: 413.1115.

[2-(4-Fluorophenoxy)-3-(trifluoromethyl)phenyl](morpholin-4-yl)methanon (**51**): Reaction of compound **5** (54 mg (0.18 mmol)) with morpholine (15 mg (0.17 mmol), potassium Oxyma-B (43 mg (0.19 mmol), and EDC × HCl (33 mg (0.17 mmol)) in dry DMF (5 mL) gave the crude product. It was purified by column chromatography (silica gel, CH_2_Cl_2_/acetonitrile 7:1), yielding compound **51** as a colorless solid (17 mg (27%)). m.P. 108 °C; Rf = 0.450 (silica gel, CH_2_Cl_2_/acetonitrile 7:1); IR = 3440, 2919, 2850, 1642, 1503, 1451, 1328, 1277, 1249, 1210, 1188, 1158, 1129, 906, 878, 845, 827, 787, and 733; ^1^H NMR (CDCl_3_, 400 MHz) δ = 3.19–3.33 (m, 3H, NCH, NCH_2_), 3.49–3.67 (m, 5H, NCH, O(CH_2_)_2_), 6.78–6.83 (m, 2H, 2′-H, 6′-H), 6.94–6.99 (m, 2H, 3′-H, 5′-H), 7.41 (t, *J* = 7.8 Hz, 1H, 5-H), 7.62 (dd, *J* = 7.8, 1.7 Hz, 1H, 6-H), and 7.79 (dd, *J* = 7.8, 1.7 Hz, 1H, 4-H); ^13^C NMR (CDCl_3_, 100 MHz) δ = 41.88 (NCH_2_), 47.20 (NCH_2_), 66.47 (OCH_2_), 66.53 (OCH_2_), 116.06 (d, *J* = 23.6 Hz, C-3′, C-5′), 117.20 (d, *J* = 8.3 Hz, C-2′, C-6′), 122.71 (q, *J* = 273 Hz, CF_3_), 124.83 (q, *J* = 31.6 Hz, C-3), 125.63 (C-5), 128.72 (q, *J* = 4.9 Hz, C-4), 131.25 (C-1), 133.48 (C-6), 149.06 (q, *J* = 1.9 Hz, C-2), 153.70 (d, *J* = 2.5 Hz, C-1′), 158.45 (d, *J* = 242 Hz, C-4′), and 165.08 (C=O); HRMS (ESI+) calculated for C_18_H_16_F_4_NO_3_^+^ [M+H]^+^: 370.1066; found: 370.1059.

#### 3.2.7. General Procedure for the Preparation of Benzamides **31**–**48** and **67** (Figure 4 and Figure 8)

The respective carboxylic acids **5**–**12** (1.00 mmol) and amines **13**–**19**, **29**, and **30** (1.00 mmol) were dissolved in dry dichloromethane (30 mL) and cooled in an ice bath to 0 °C. 2-Chloro-*N*-methylpyridin-1-ium iodide (1.75 mmol) and diisopropylethylamine (DIPEA) (5.00 mmol) were added. The reaction mixture was stirred at room temperature for 24–28 h. After completion, 50 mL of saturated aq NH_4_Cl was added. The aqueous and organic phases were separated. The aqueous phase was extracted twice with ethyl acetate. The organic phases were combined, washed with 8% aq NaHCO_3_ and brine, dried over anhydrous sodium sulfate, and filtered. The solvent was evaporated in vacuo yielding the crude products, which were purified by column chromatography and/or recrystallization.

2-(4-Fluorophenoxy)-*N*-[2-(4-methylpiperazin-1-yl)phenyl]-3-(trifluoromethyl)benzamide (**31**): Reaction of the carboxylic acid **5** (219 mg (0.73 mmol)) with the amine **14** (136 mg (0.71 mmol)), 2-chloro-*N*-methylpyridin-1-ium iodide (333 mg (1.30 mmol)), and DIPEA (452 mg (3.50 mmol)) in dry dichloromethane (30 mL) gave the crude product. It was purified by column chromatography (flash silica gel, CH/EtOH 1:1), yielding compound **31** as a colorless solid (114 mg (34%)). m.P. 192 °C; Rf = 0.338 (silica gel, CH_2_Cl_2_/*Me*OH 39:1); IR = 3287, 2795, 1668, 1591, 1499, 1450, 1370, 1311, 1243, 1218, 1163, 1128, 1009, 916, 836, 775, and 690; ^1^H NMR (CDCl_3_, 400 MHz) δ = 2.35 (s, 3H, NCH_3_), 2.65 (br, 4H, N(CH_2_)_2_), 2.91 (t, *J* = 4.3 Hz, 4H, N(CH_2_)_2_), 6.68–6.72 (m, 2H, 2′-H, 6′-H), 6.83–6.87 (m, 2H, 3′-H, 5′-H), 7.06 (td, *J* = 7.6, 1.7 Hz, 1H, 4″-H), 7.11 (td, *J* = 7.7, 1.7 Hz, 1H, 5″-H), 7.17 (dd, *J* = 7.6, 1.7 Hz, 1H, 3″-H), 7.53 (t, *J* = 7.8 Hz, 1H, 5-H), 7.90 (dd, *J* = 7.7, 1.7 Hz, 1H, 4-H), 8.23 (dd, *J* = 7.9, 1.7 Hz, 1H, 6-H), 8.30 (dd, *J* = 7.9, 1.7 Hz, 1H, 6″-H), and 9.84 (s, 1H, NH); ^13^C NMR (CDCl_3_, 100 MHz) δ = 46.07 (NCH_3_), 52.18 (N(CH_2_)_2_), 55.63 (N(CH_2_)_2_), 116.23 (d, *J* = 23.8 Hz, C-3′, C-5′), 116.38 (d, *J* = 8.4 Hz, C-2′, C-6′), 119.32 (C-6″), 120.58 (C-3″), 122.73 (q, *J* = 274 Hz, CF_3_), 124.28 (C-4″), 125.33 (q, *J* = 31.8 Hz, C-3), 125.56 (C-5″), 126.11 (C-5), 130.25 (q, *J* = 4.6 Hz, C-4), 132.00 (C-1), 133.27 (C-1″), 135.40 (C-6), 141.17 (C-2″), 149.79 (q, *J* = 1.8 Hz, C-2), 154.26 (d, *J* = 2.3 Hz, C-1′), 158.29 (d, *J* = 242 Hz, C-4′), and 161.55 (C=O); HRMS (EI+) calculated for C_25_H_23_F_4_N_3_O_2_: 473.1726; found: 473.1759.

2-(4-Fluorophenoxy)-*N*-[2-(morpholin-4-yl)phenyl]-3-(trifluoromethyl)benzamide (**32**): Reaction of the carboxylic acid **5** (225 mg (0.75 mmol)) with the amine **15** (127 mg (0.71 mmol)), 2-chloro-*N*-methylpyridin-1-ium iodide (333 mg (1.30 mmol)), and DIPEA (452 mg (3.50 mmol)) in dry dichloromethane (30 mL) gave the crude product. It was purified by column chromatography (flash silica gel, CH/EtAc 9:1), yielding compound **32** as a colorless solid (82 mg (25%)). m.P. 147 °C; Rf = 0.175 (silica gel, CH/EtAc 9:1); IR = 3316, 2970, 2826, 1678, 1591, 1521, 1499, 1445, 1324, 1219, 1162, 1121, 936, 824, 785, 764, and 717; ^1^H NMR (CDCl_3_, 400 MHz) δ = 2.88 (t, *J* = 4.3 Hz, 4H, N(CH_2_)_2_), 3.92 (t, *J* = 4.5 Hz, 4H, O(CH_2_)_2_), 6.68–6.72 (m, 2H, 2′-H, 6′-H), 6.84–6.88 (m, 2H, 3′-H, 5′-H), 7.10 (td, *J* = 7.6, 1.8 Hz, 1H, 4″-H), 7.14 (td, *J* = 7.6, 1.8 Hz, 1H, 5″-H), 7.18 (dd, *J* = 7.6, 1.8 Hz, 1H, 3″-H), 7.53 (t, *J* = 7.8 Hz, 1H, 5-H), 7.90 (dd, *J* = 7.7, 1.7 Hz, 1H, 4-H), 8.23 (dd, *J* = 7.9, 1.7 Hz, 1H, 6-H), 8.30 (br d, *J* = 7.8 Hz, 1H, 6″-H), and 9.82 (s, 1H, NH); ^13^C NMR (CDCl_3_, 100 MHz) δ =52.69 (N(CH_2_)_2_), 67.35 (O(CH_2_)_2_), 116.29 (d, *J* = 23.8 Hz, C-3′, C-5′), 116.44 (d, *J* = 6.9 Hz, C-2′, C-6′), 119.84 (C-6″), 120.54 (C-3″), 122.69 (q, *J* = 274 Hz, CF_3_), 124.51 (C-4″), 125.35 (q, *J* = 31.5 Hz, C-3), 125.91 (C-5″), 126.18 (C-5), 130.44 (q, *J* = 5.4 Hz, C-4), 131.90 (C-1), 133.20 (C-1″), 135.35 (C-6), 140.85 (C-2″), 149.82 (q, *J* = 1.9 Hz, C-2), 154.17 (d, *J* = 2.3 Hz, C-1′), 158.33 (d, *J* = 242 Hz, C-4′), and 161.76 (C=O); HRMS (EI+) calculated for C_24_H_20_F_4_N_2_O_3_: 460.1410; found: 460.1412.

2-(4-Fluorophenoxy)-*N*-[4-(morpholin-4-yl)phenyl]-3-(trifluoromethyl)benzamide (**33**): Reaction of the carboxylic acid **5** (301 mg (1.00 mmol)) with the amine **16** (180 mg (1.01 mmol)), 2-chloro-*N*-methylpyridin-1-ium iodide (500 mg (1.96 mmol)), and DIPEA (646 mg (5.00 mmol)) in dry dichloromethane (30 mL) gave the crude product. It was purified by column chromatography (flash silica gel, CH_2_Cl_2_/acetonitrile 6:1), yielding compound **33** as a colorless solid (115 mg (25%)). m.P. 210 °C; Rf = 0.438 (silica gel, CH_2_Cl_2_/acetonitrile 6:1); IR = 3302, 1650, 1597, 1520, 1501, 1452, 1317, 1218, 1124, 925, 824, and 776; ^1^H NMR (CDCl_3_, 400 MHz) δ = 3.09–3.12 (m, 4H, N(CH_2_)_2_), 3.83–3.86 (m, 4H, O(CH_2_)_2_), 6.75–6.79 (m, 2H, 2′-H, 6′-H), 6.82 (d, *J* = 8.8 Hz, 2H, 3″-H, 5″-H), 6.91–6.96 (m, 2H, 3′-H, 5′-H), 7.24 (d, *J* = 8.7 Hz, 2H, 2″-H, 6″-H), 7.53 (t, *J* = 7.8 Hz, 1H, 5-H), 7.88 (dd, *J* = 7.8, 1.6 Hz, 1H, 4-H), 8.29 (dd, *J* = 7.9, 1.6 Hz, 1H, 6-H), and 8.36 (s, 1H, NH); ^13^C NMR (CDCl_3_, 100 MHz) δ = 49.47 (N(CH_2_)_2_), 66.84 (O(CH_2_)_2_), 116.05 (C-3″, C-5″), 116.17 (d, *J* = 8.1 Hz, C-2′, C-6′), 116.53 (d, *J* = 23.8 Hz, C-3′, C-5′), 121.76 (C-2″, C-6″), 122.69 (q, *J* = 273 Hz, CF_3_), 125.18 (q, *J* = 31.6 Hz, C-3), 126.27 (C-5), 129.64 (C-1″), 130.49 (q, *J* = 4.9 Hz, C-4), 130.92 (C-1), 135.90 (C-6), 148.73 (C-4″), 149.39 (q, *J* = 1.9 Hz, C-2), 154.02 (d, *J* = 2.5 Hz, C-1′), 158.48 (d, *J* = 242 Hz, C-4′), and 161.40 (C=O); HRMS (ESI+) calculated for C_24_H_21_F_4_N_2_O_3_^+^ [M+H^+^]: 461.1488; found: 461.1491.

2-(4-Fluorophenoxy)-*N*-[4-(pyrrolidin-4-yl)phenyl]-3-(trifluoromethyl)benzamide (**34**): Reaction of the carboxylic acid **5** (307 mg (1.02 mmol)) with the amine **17** (167 mg (1.03 mmol), 2-chloro-*N*-methylpyridin-1-ium iodide (470 mg (1.84 mmol)), and DIPEA (646 mg (5.00 mmol)) in dry dichloromethane (30 mL) gave the crude product. It was purified by column chromatography (flash silica gel, CH/EtAc 4:1), yielding compound **34** as a pale-yellow solid (86 mg (19%)). m.P. 177 °C; Rf = 0.313 (silica gel, CH_2_Cl_2_/EtAc 4:1); IR = 1645, 1522, 1499, 1448, 1372, 1316, 1248, 1212, 1116, 837, 807, 771, and 686; ^1^H NMR (CDCl_3_, 400 MHz) δ = 1.96–2.00 (m, 4H, (CH_2_)_2_), 3.22–3.26 (m, 4H, N(CH_2_)_2_), 6.44–6.47 (m, 2H, 3″-H, 5″-H), 6.75–6.79 (m, 2H, 2′-H, 6′-H), 6.91–6.96 (m, 2H, 3′-H, 5′-H), 7.11–7.15 (m, 2H, 2″-H, 6″-H), 7.51 (t, *J* = 7.8 Hz, 1H, 5-H), 7.87 (dd, *J* = 7.8, 1.7 Hz, 1H, 4-H), 8.27 (s, 1H, NH), and 8.29 (dd, *J* = 7.8, 1.7 Hz, 1H, 6-H); ^13^C NMR (CDCl_3_, 100 MHz) δ = 25.44 ((CH_2_)_2_), 47.71 (N(CH_2_)_2_), 111.55 (C-3″, C-5″), 116.22 (d, *J* = 8.3 Hz, C-2′, C-6′), 116.47 (d, *J* = 23.7 Hz, C-3′, C-5′), 122.47 (C-2″, C-6″), 122.76 (q, *J* = 273 Hz, CF_3_), 125.09 (q, *J* = 31.6 Hz, C-3), 125.53 (C-1″), 126.16 (C-5), 130.19 (q, *J* = 4.9 Hz, C-4), 131.26 (C-1), 135.88 (C-6), 145.85 (C-4″), 149.38 (q, *J* = 1.8 Hz, C-2), 154.08 (d, *J* = 2.4 Hz, C-1′), 158.45 (d, *J* = 242 Hz, C-4′), and 161.26 (C=O); HRMS (ESI+) calculated for C_24_H_21_F_4_N_2_O_2_^+^ [M+H^+^]: 445.1523; found: 445.1522.

*N*-[2-(Dimethylamino)phenyl]-2-(4-fluorophenoxy)-3-(trifluoromethyl)benzamide (**35**): reaction of the carboxylic acid **5** (242 mg (0.84 mmol)) with the amine **29** (116 mg (0.85 mmol)), 2-chloro-*N*-methylpyridin-1-ium iodide (400 mg (1.57 mmol)), and DIPEA (517 mg (4.00 mmol)) in dry dichloromethane (30 mL) gave the crude product. It was purified by column chromatography (flash silica gel, CH/EtAc 9:1), yielding compound **35** as a colorless solid (144 mg (41%)). m.P. 126 °C; Rf = 0.200 (silica gel, CH/EtAc 9:1); IR = 3322, 1664, 1592, 1502, 1449, 1325, 1213, 1182, 1133, 941, 825, 785, 767, 748, and 688; ^1^H NMR (CDCl_3_, 400 MHz) δ = 2.58 (s, 6H, N(CH_3_)_2_), 6.73–6.76 (m, 2H, 2′-H, 6′-H), 6.85–6.90 (m, 2H, 3′-H, 5′-H), 7.05 (td, *J* = 7.4, 1.7 Hz, 1H, 4″-H), 7.08 (td, *J* = 7.4, 1.8 Hz, 1H, 5″-H), 7.16 (dd, *J* = 7.3, 2.1 Hz, 1H, 3″-H), 7.52 (t, *J* = 7.8 Hz, 1H, 5-H), 7.88 (dd, *J* = 7.8, 1.8 Hz, 1H, 4-H), 8.28 (dd, *J* = 7.6, 2.0 Hz, 1H, 6″-H), 8.31 (dd, *J* = 7.8, 1.8 Hz, 1H, 6-H), and 9.79 (s, 1H, NH); ^13^C NMR (CDCl_3_, 100 MHz) δ =44.95 (N(CH_3_)_2_), 116.09 (d, *J* = 23.6 Hz, C-3′, C-5′), 116.77 (d, *J* = 8.2 Hz, C-2′, C-6′), 119.83 (C-6″), 120.02 (C-3″), 122.76 (q, *J* = 274 Hz, CF_3_), 124.33 (C-4″), 125.02 (C-5″), 125.06 (q, *J* = 31.7 Hz, C-3), 125.96 (C-5), 130.57 (q, *J* = 5.0 Hz, C-4), 131.56 (C-1), 133.23 (C-1″), 135.66 (C-6), 143.24 (C-2″), 150.02 (q, *J* = 1.8 Hz, C-2), 154.28 (d, *J* = 1.7 Hz, C-1′), 158.36 (d, *J* = 242 Hz, C-4′), and 161.68 (C=O); HRMS (EI+) calculated for C_22_H_18_F_4_N_2_O_2_: 418.1304; found: 418.1309.

*N*-[4-(Dimethylamino)phenyl]-2-(4-fluorophenoxy)-3-(trifluoromethyl)benzamide (**36**): Reaction of the carboxylic acid **5** (435 mg (1.45 mmol)) with the amine **30** (212 mg (1.56 mmol)), 2-chloro-*N*-methylpyridin-1-ium iodide (737 mg (2.88 mmol)), and DIPEA (1008 mg (7.80 mmol)) in dry dichloromethane (40 mL) gave the crude product. It was purified by column chromatography (flash silica gel, CH_2_Cl_2_/*Me*OH 149:1), yielding compound **36** as a pale-yellow solid (127 mg (21%)). m.P. 160 °C; Rf = 0.313 (silica gel, CH_2_Cl_2_/*Me*OH 149:1); IR = 1645, 1499, 1451, 1316, 1247, 1215, 1186, 1140, 945, 814, 776, and 675; ^1^H NMR (CDCl_3_, 400 MHz) δ = 2.91 (s, 6H, N(CH_3_)_2_), 6.61–6.66 (m, 2H, 2″-H, 6″-H), 6.75–6.80 (m, 2H, 2′-H, 6′-H), 6.91–6.96 (m, 2H, 3″-H, 5″-H), 7.14–7.18 (m, 2H, 3′-H, 5′-H), 7.51 (t, *J* = 7.8 Hz, 1H, 5-H), 7.87 (dd, *J* = 7.8, 1.7 Hz, 1H, 4-H), and 8.27–8.30 (m, 2H, 6-H, NH); ^13^C NMR (CDCl_3_, 100 MHz) δ = 40.72 (N(CH_3_)_2_), 112.74 (C-3″, C-5″), 116.20 (d, *J* = 8.3 Hz, C-2′, C-6′), 116.49 (d, *J* = 23.7 Hz, C-3′, C-5′), 122.17 (C-2″, C-6″), 122.73 (q, *J* = 273 Hz, CF_3_), 125.11 (q, *J* = 31.6 Hz, C-3), 126.19 (C-5), 126.81 (C-1″), 130.29 (q, *J* = 4.9 Hz, C-4), 131.15 (C-1), 135.89 (C-6), 148.39 (C-4″), 149.38 (q, *J* = 1.9 Hz, C-2), 154.06 (d, *J* = 2.5 Hz, C-1′), 158.46 (d, *J* = 242 Hz, C-4′), and 161.33 (C=O); HRMS (ESI+) calculated for C_22_H_19_F_4_N_2_O_2_^+^ [M+H^+^]: 419.1377; found: 419.1369.

*tert*-Butyl-4-{2-[2-(4-fluorophenoxy)-3-(trifluoromethyl)benzamido]phenyl}pipera-zine-1-carboxylate (**37** (**MMV030666**)): Reaction of the carboxylic acid **5** (210 mg (0.70 mmol)) with the amine **13** (194 mg (0.70 mmol)), 2-chloro-*N*-methylpyridin-1-ium iodide (316 mg (1.24 mmol)), and DIPEA (452 mg (3.50 mmol)) in dry dichloromethane (30 mL) gave the crude product. It was purified by column chromatography (silica gel, CH_2_Cl_2_/*Me*OH 99:1), yielding compound **37** as a pale-yellow amorphous solid (51 mg (13%)). NMR data were in accordance with the literature data [12].

*tert*-Butyl-4-{3-[2-(4-fluorophenoxy)-3-(trifluoromethyl)benzamido]phenyl}piperazine-1-carboxylate (**38**): Reaction of the carboxylic acid **5** (329 mg (1.10 mmol)) with the amine **18** (302 mg (1.09 mmol)), 2-chloro-*N*-methylpyridin-1-ium iodide (486 mg (1.90 mmol)), and DIPEA (698 mg (5.40 mmol)) in dry dichloromethane (35 mL) gave the crude product. It was purified by column chromatography (silica gel, CH/EtAc 2:1), yielding compound **38** as a pale-yellow amorphous solid (177 mg (29%)). NMR data were in accordance with the literature data [12].

*tert*-Butyl-4-{4-[2-(4-fluorophenoxy)-3-(trifluoromethyl)benzamido]phenyl}piperazine-1-carboxylate (**39**): Reaction of the carboxylic acid **5** (305 mg (1.02 mmol)) with the amine **19** (280 mg (1.01 mmol)), 2-chloro-*N*-methylpyridin-1-ium iodide (452 mg (1.77 mmol)), and DIPEA (646 mg (5.00 mmol)) in dry dichloromethane (30 mL) gave the crude product. It was purified by column chromatography (silica gel, CH/EtAc 3:1), yielding compound **39** as a pale-yellow amorphous solid (23 mg (4%)). NMR data were in accordance with the literature data [12].

*tert*-Butyl-4-{4-[2-phenoxy-3-(trifluoromethyl)benzamido]phenyl}piperazine-1-carboxylate (**40**): Reaction of the carboxylic acid **9** (264 mg (0.94 mmol)) with the amine **19** (274 mg (0.99 mmol)), 2-chloro-*N*-methylpyridin-1-ium iodide (438 mg (1.71 mmol)), and DIPEA (607 mg (4.70 mmol)) in dry dichloromethane (30 mL) gave the crude product. It was purified by column chromatography (flash silica gel, CH/EtAc 2:1), yielding compound **40** as a pale-yellow amorphous solid (153 mg (30%)). NMR data were in accordance with the literature data [12].

*tert*-Butyl-4-{2-[2-(4-acetamidophenoxy)-3-(trifluoromethyl)benzamido]phenyl}piperazine-1-carboxylate (**41**): Reaction of the carboxylic acid **11** (708 mg (2.09 mmol)) with the amine **13** (579 mg (2.09 mmol)), 2-chloro-*N*-methylpyridin-1-ium iodide (933 mg (3.65 mmol)), and DIPEA (1349 mg (10.44 mmol)) in dry dichloromethane (100 mL) gave the crude product. It was purified by column chromatography (flash silica gel, CH_2_Cl_2_/*Me*OH 29:1), yielding compound **41** as a colorless amorphous solid (1038 mg (83%)). NMR data were in accordance with the literature data [12].

*tert*-Butyl-4-{4-[2-(4-fluorophenoxy)benzamido]phenyl}piperazine-1-carboxylate (**42**): Reaction of the carboxylic acid **6** (240 mg (1.03 mmol)) with the amine **19** (281 mg (1.01.mmol)), 2-chloro-*N*-methylpyridin-1-ium iodide (449 mg (1.76 mmol)), and DIPEA (646 mg (5.00 mmol)) in dry dichloromethane (30 mL) gave the crude product. It was purified by column chromatography (silica gel, CH/EtAc 2:1), yielding compound **42** as a pale-brown amorphous solid (134 mg (27%)). NMR data were in accordance with the literature data [13].

*tert*-Butyl-4-{4-[3-fluoro-2-(4-fluorophenoxy)benzamido]phenyl}piperazine-1-carboxylate (**43**): Reaction of the carboxylic acid **7** (106 mg (0.42 mmol)) with the amine **19** (119 mg (0.43 mmol)), 2-chloro-*N*-methylpyridin-1-ium iodide (191 mg (0.75 mmol)), and DIPEA (274 mg (2.12 mmol)) in dry dichloromethane (13 mL) gave the crude product. It was purified by column chromatography (silica gel, CH_2_Cl_2_/EtAc 9:1), yielding compound **43** as a pale-brown amorphous solid (92 mg (43%)). NMR data were in accordance with the literature data [13].

*tert*-Butyl-4-{4-[2-(4-fluorophenoxy)-3-nitrobenzamido]phenyl}piperazine-1-carboxylate (**44**): Reaction of the carboxylic acid **8** (562 mg (2.03 mmol)) with the amine **19** (557 mg (2.01 mmol)), 2-chloro-*N*-methylpyridin-1-ium iodide (898 mg (3.51 mmol)), and DIPEA (1292 mg (10.00 mmol)) in dry dichloromethane (60 mL) gave the crude product. It was purified by column chromatography (silica gel, CH_2_Cl_2_/acetonitrile 12:1), yielding compound **44** as a yellow amorphous solid (421 mg (39%)). NMR data were in accordance with the literature data [13].

*tert*-Butyl-4-(4-{2-[(4-fluorophenyl)sulfanyl]-3-(trifluoromethyl)benzamido}phenyl)piperazine-1-carboxylate (**45**): Reaction of the carboxylic acid **12** (212 mg (0.67 mmol)) with the amine **19** (190 mg (0.69 mmol)), 2-chloro-*N*-methylpyridin-1-ium iodide (300 mg (1.17 mmol)), and DIPEA (433 mg (3.35 mmol)) in dry dichloromethane (20 mL) gave the crude product. It was purified by column chromatography (silica gel, CH_2_Cl_2_/*Me*OH 79:1), yielding compound **45** as a pale-brown amorphous solid (66 mg (17%)). NMR data were in accordance with the literature data [13].

*tert*-Butyl-4-(2-{2-[(4-fluorophenyl)sulfanyl]-3-(trifluoromethyl)benzamido}phenyl) piperazine-1-carboxylate (**46**): Reaction of the carboxylic acid **12** (235 mg (0.74 mmol)) with the amine **13** (212 mg (0.76 mmol)), 2-chloro-*N*-methylpyridin-1-ium iodide (339 mg (1.33 mmol)), and DIPEA (479 mg (3.70 mmol)) in dry dichloromethane (22 mL) for 48 h gave the crude product. It was purified by column chromatography (silica gel, CH/EtAc 4:1), yielding compound **46** as a colorless amorphous solid (189 mg (44%)). NMR data were in accordance with the literature data [13].

*tert*-Butyl-4-{4-[2-(4-acetamidophenoxy)-3-(trifluoromethyl)benzamido]phenyl} piperazine-1-carboxylate (**47**): Reaction of the carboxylic acid **11** (339 mg (1.00 mmol)) with the amine **19** (282 mg (1.02 mmol), 2-chloro-*N*-methylpyridin-1-ium iodide (455 mg (1.78 mmol)), and DIPEA (646 mg (5.00 mmol)) in dry dichloromethane (30 mL) for 48 h gave the crude product. It was purified by column chromatography (silica gel, CH_2_Cl_2_/acetonitrile 2:1), yielding compound **47** as a colorless solid (82 mg (14%)). m.P. 233 °C; Rf = 0.238 (silica gel, CH_2_Cl_2_/*Me*OH 29:1); IR = 1654, 1604, 1514, 1448, 1406, 1331, 1247, 1139, 925, 821, and 769; ^1^H NMR (CDCl_3_, 400 MHz) δ = 1.48 (s, 9H, (CH_3_)_3_), 2.12 (s, 3H, CH_3_), 3.05–3.08 (m, 4H, N(CH_2_)_2_), 3.54–3.57 (m, 4H, N(CH_2_)_2_), 6.76 (d, *J* = 8.4 Hz, 2H, 2′-H, 6′-H), 6.82 (d, *J* = 8.5 Hz, 2H, 3″-H, 5″-H), 7.09 (s, 1H, NH), 7.24–7.27 (m, 2H, 2″-H, 6″-H), 7.39 (d, *J* = 8.4 Hz, 2H, 3′-H, 5′-H), 7.52 (t, *J* = 7.7 Hz, 1H, 5-H), 7.88 (d, *J* = 7.7 Hz, 1H, 4-H), 8.31 (d, *J* = 7.7 Hz, 1H, 6-H), and 8.47 (s, 1H, NH); ^13^C NMR (CDCl_3_, 100 MHz) δ = 24.43 (CH_3_), 28.42 ((CH_3_)_3_), 43.61 (N(CH_2_)_2_), 49.56 (N(CH_2_)_2_), 79.92 (C*Me*_3_), 115.30 (C-2′, C-6′), 116.99 (C-3″, C-5″), 121.42 (C-3′, C-5′), 121.80 (C-2″, C-6″), 122.70 (q, *J* = 273 Hz, CF_3_), 125.27 (q, *J* = 31.8 Hz, C-3), 126.19 (C-5), 129.91 (C-1″), 130.52 (q, *J* = 4.8 Hz, C-4), 130.85 (C-1), 133.51 (C-4′), 135.87 (C-6), 148.64 (C-4″), 149.35 (q, *J* = 1.3 Hz, C-2), 154.42 (C-1′), 154.69 (N(C=O)O), 161.45 (ArC=O), and 168.04 (CH_3_C=O); HRMS (ESI+) calculated for C_31_H_34_F_3_N_4_O_5_^+^ [M+H^+^]: 599.2476; found: 599.2464.

*tert*-Butyl-4-[4-(2-phenoxybenzamido)phenyl]piperazine-1-carboxylate (**48**): Reaction of compound **10** (291 mg (1.36 mmol)) with the amine **19** (378 mg (1.36 mmol)), 2-chloro-*N*-methylpyridin-1-ium iodide (608 mg (2.38 mmol)), and DIPEA (879 mg (6.80 mmol)) in dry dichloromethane (40 mL) gave the crude product. It was purified by column chromatography (silica gel, CH/EtAc 2.5:1), yielding compound **48** as a colorless amorphous solid (262 mg (40%)). NMR data were in accordance with the literature data [13].

*tert*-Butyl-4-{4-[3-(trifluoromethyl)benzamido]phenyl}piperazine-1-carboxylate (**67**): Reaction of 3-(trifluoromethyl)benzoic acid (194 mg (1.02 mmol)) with the amine **19** (280 mg (1.01 mmol)), 2-chloro-*N*-methylpyridin-1-ium iodide (447 mg (1.75 mmol)), and DIPEA (646 mg (5.00 mmol)) in dry dichloromethane (30 mL) gave the crude product. It was purified by column chromatography (silica gel, CH/EtAc 2:1), yielding compound **67** as a pale-brown solid (264 mg (58%)). m.P. 172 °C; Rf = 0.213 (silica gel, CH/EtAc 2:1); IR = 1643, 1514, 1315, 1229, 1115, 904, 811, and 695; ^1^H NMR (CDCl_3_, 400 MHz) δ = 1.49 (s, 9H, (CH_3_)_3_), 3.09–3.12 (m, 4H, N(CH_2_)_2_), 3.57–3.60 (m, 4H, N(CH_2_)_2_), 6.91–6.95 (m, 2H, 3″-H, 5″-H), 7.51–7.56 (m, 2H, 2″-H, 6″-H), 7.61 (br t, *J* = 7.8 Hz, 1H, 5-H), 7.79 (br d, *J* = 7.8 Hz, 1H, 4-H), 7.87 (br s, 1H, NH), 8.05 (br d, *J* = 7.8 Hz, 1H, 6-H), and 8.12 (br s, 1H, 2-H); ^13^C NMR (CDCl_3_, 100 MHz) δ = 28.42 ((CH_3_)_3_), 43.51 (N(CH_2_)_2_), 49.63 (N(CH_2_)_2_), 79.96 (C*Me*_3_), 117.13 (C-3″, C-5″), 121.78, 121.89 (C-2″, C-6″), 123.67 (q, *J* = 272 Hz, CF_3_), 123.97 (q, *J* = 3.8 Hz, C-2), 128.22 (q, *J* = 3.8 Hz, C-4), 129.37 (C-5), 130.18, 130.25 (C-1″), 130.32 (C-6), 131.26 (q, *J* = 32.9 Hz, C-3), 135.88 (C-1), 148.69, 148.71 (C-4″), 154.70 (N(C=O)O), and 164.10 (C=O). HRMS (ESI+) calculated for C_23_H_27_F_3_N_3_O_3_^+^ [M+H^+^]: 450.1999; found: 450.1991.

#### 3.2.8. General Procedure for the Preparation of N-aryl-piperazines **52**–**64** and **66** (Figure 6 and Figure 8)

The respective *N*-*Boc*-piperazine derivatives **37**–**48** and **67** (1.00 mmol) were dissolved in dry dichloromethane (10 mL) and cooled to 0 °C. A solution of trifluoroacetic acid (6.00–12.00 mmol) in dry dichloromethane (3 mL) was added via a dropping funnel. The reaction mixture was stirred at room temperature for 24–48 h. After that, excess solvent and trifluoroacetic acid were evaporated in vacuo. The residue was suspended in a solution of potassium carbonate (6.00 mmol) in aqua demin (12 mL). The aqueous phase was extracted five times with a 3:1 mixture of dichloromethane and propan-2-ol. The organic phases were combined, dried over anhydrous sodium sulfate, filtered, and the solvent was evaporated in vacuo, yielding the desired *N*-aryl-piperazines which were either obtained as pure compounds or had to be purified by column chromatography.

[2-(4-Fluorophenoxy)-3-(trifluoromethyl)phenyl](piperazin-1-yl)methanon (**52**): Reaction of compound **50** (104 mg (0.22 mmol)) with trifluoroacetic acid (753 mg (6.60 mmol)) in dry dichloromethane (10 mL) gave the protonated form of **52**. Work-up with an aqueous solution of potassium carbonate (628 mg (4.54 mol)) followed by column chromatography yielded compound **52** as a colorless oil (66 mg (82%)). Rf = 0.288 (silica gel, CH_2_Cl_2_//EtOH 39:1); IR = 3441, 1636, 1503, 1449, 1327, 1249, 1220, 1136, 823, and 778; ^1^H NMR (CDCl_3_) δ = 2.66–2.86 (m, 4H, 2 NCH_2_), 3.11–3.17 (m, 1H, NCH), 3.19–3.22 (m, 2H, NCH_2_), 3.62–3.67 (m, 1H, NCH), 6.79–6.82 (m, 2H, 2′-H, 6′-H), 6.93–6.98 (m, 2H, 3′-H, 5′-H), 7.39 (t, *J* = 7.8 Hz, 1H, 5-H), 7.60 (dd, *J* = 7.7, 1.7 Hz, 1H, 6-H), and 7.77 (dd, *J* = 7.8, 1.7 Hz, 1H, 4-H); ^13^C NMR (CDCl_3_) δ = 42.60 (NCH_2_), 45.54 (NCH_2_), 46.12 (NCH_2_), 48.09 (NCH_2_), 115.97 (d, *J* = 23.6 Hz, C-3′, C-5′), 117.34 (d, *J* = 8.2 Hz, C-2′, C-6′), 122.78 (q, *J* = 273 Hz, CF_3_), 124.73 (q, *J* = 31.5 Hz, C-3), 125.47 (C-5), 128.45 (q, *J* = 4.9 Hz, C-4), 131.67 (C-1), 133.43 (C-6), 149.11 (q, *J* = 1.9 Hz, C-2), 153.71 (d, *J* = 2.5 Hz, C-1′), 158.42 (d, *J* = 241 Hz, C-4′), and 165.00 (C=O); HRMS (ESI+) calculated for C_18_H_17_F_4_N_2_O_2_^+^ [M+H]^+^: 369.1226; found: 369.1218.

2-(4-Fluorophenoxy)-*N*-[2-(piperazin-1-yl)phenyl]-3-(trifluoromethyl)benzamide **(53**): Reaction of compound **37** (398 mg (0.70 mmol)) with trifluoroacetic acid (2390 mg (21 mmol)) in dry dichloromethane (9 mL) gave the protonated product. Work-up with an aqueous solution of potassium carbonate (1940 mg (14 mmol)) and subsequent column chromatography (flash silica gel, CH_2_Cl_2_/*Me*OH 19:1) yielded compound **53** as a colorless solid (64 mg (20%)). m.P. 117 °C; Rf = 0.200 (silica gel, CH_2_Cl_2_/*Me*OH 19:1); IR = 3423, 1676, 1592, 1501, 1448, 1326, 1219, 1138, 780, and 689; ^1^H NMR (CDCl_3_, 400 MHz) δ = 2.85 (t, *J* = 4.7 Hz, 4H, N(CH_2_)_2_), 3.09 (t, *J* = 4.8 Hz, 4H, N(CH_2_)_2_), 6.69–6.73 (m, 2H, 2′-H, 6′-H), 6.83–6.87 (m, 2H, 3′-H, 5′-H), 7.06 (td, *J* = 7.4, 1.5 Hz, 1H, 4″-H), 7.08 (td, *J* = 7.6, 1.7 Hz, 1H, 5″-H), 7.17 (dd, *J* = 7.2, 1.7 Hz, 1H, 3″-H), 7.52 (t, *J* = 7.9 Hz, 1H, 5-H), 7.89 (dd, *J* = 7.9, 1.7 Hz, 1H, 4-H), 8.21 (dd, *J* = 7.6, 1.7 Hz, 1H, 6-H), 8.30 (dd, *J* = 7.9, 1.3 Hz, 1H, 6″-H), and 9.78 (s, 1H, NH); ^13^C NMR (CDCl_3_, 100 MHz) δ = 46.63 (N(CH_2_)_2_), 53.46 (N(CH_2_)_2_), 116.22 (d, *J* = 23.4 Hz, C-3′, C-5′), 116.40 (d, *J* = 8.3 Hz, C-2′, C-6′), 119.42 (C-6″), 120.63 (C-3″), 122.71 (q, *J* = 273 Hz, CF_3_), 124.32 (C-4″), 125.27 (q, *J* = 31.6 Hz, C-3), 125.48 (C-5″), 126.08 (C-5), 130.28 (q, *J* = 4.9 Hz, C-4), 132.00 (C-1), 133.19 (C-1″), 135.32 (C-6), 141.67 (C-2″), 149.81 (q, *J* = 1.7 Hz, C-2), 154.23 (d, *J* = 2.7 Hz, C-1′), 158.28 (d, *J* = 242 Hz, C-4′), and 161.68 (C=O); HRMS (EI+) calculated for C_24_H_21_F_4_N_3_O_2_: 459.1570; found: 459.1567.

2-(4-Acetamidophenoxy)-*N*-[2-(piperazin-1-yl)phenyl]-3-(trifluoromethyl) benzamide (**54**): Reaction of compound **41** (306 mg (0.51 mmol)) with trifluoroacetic acid (1710 mg (15.00 mmol)) in dry dichloromethane (9 mL) gave the protonated form of **54**. Work-up with a solution of potassium carbonate (1394 mg (10.09 mmol)) in aqua demin gave the crude product. It was purified by column chromatography (silica gel, CHCl_3_/*Me*OH 19:1), yielding compound **54** as a colorless oil (117 mg (46%)). Rf = 0.200 (silica gel, CH_2_Cl_2_/EtAc/*Me*OH 1:1:1); IR = 3424, 1669, 1506, 1448, 1320, 1233, 1136, and 759; ^1^H NMR (CDCl_3_, 400 MHz) δ = 2.08 (s, 3H, CH_3_), 2.83–2.85 (m, 4H, N(CH_2_)_2_), 3.07–3.10 (m, 4H, N(CH_2_)_2_), 6.70 (d, *J* = 9.0 Hz, 2H, 2′-H, 6′-H), 7.03–7.11 (m, 2H, 4″-H, 5″-H), 7.15–7.18 (m, 2H, 3″-H, NH), 7.30 (d, *J* = 9.0 Hz, 2H, 3′-H, 5′-H), 7.51 (t, *J* = 7.8 Hz, 1H, 5-H), 7.88 (dd, *J* = 7.9, 1.7 Hz, 1H, 4-H), 8.23 (dd, *J* = 7.8, 1.7 Hz, 1H, 6-H), 8.31 (dd, *J* = 7.5, 2.0 Hz, 1H, 6″-H), and 9.86 (s, 1H, NH); ^13^C NMR (CDCl_3_, 100 MHz) δ = 24.36 (CH_3_), 46.62 (N(CH_2_)_2_), 53.47 (N(CH_2_)_2_), 115.61 (C-2′, C-6′), 119.51 (C-6″), 120.67 (C-3″), 121.22 (C-3′, C-5′), 122.73 (q, *J* = 273 Hz, CF_3_), 124.32 (C-4″), 125.39 (q, *J* = 31.6 Hz, C-3), 125.43 (C-5″), 126.00 (C-5), 130.30 (q, *J* = 4.8 Hz, C-4), 132.01 (C-1), 133.21 (C-4′), 133.27 (C-1″), 135.32 (C-6), 141.78 (C-2″), 149.77 (q, *J* = 1.7 Hz, C-2), 154.71 (C-1′), 161.73 (ArC=O), and 168.02 (CH_3_C=O); HRMS (ESI+) calculated for C_26_H_26_F_3_N_4_O_3_^+^ [M+H]^+^: 499.1957; found: 499.1966.

2-[(4-Fluorophenyl)sulfanyl]-*N*-[2-(piperazin-1-yl)phenyl]-3-(trifluoromethyl)benz-amide (**55**): Reaction of compound **46** (102 mg (0.18 mmol)) with trifluoroacetic acid (592 mg (5.19 mmol)) in dry dichloromethane (5 mL) gave the protonated form of **55**. Work-up with a solution of potassium carbonate (480 mg (3.47 mmol)) in aqua demin yielded pure compound **55** as a yellow oil (78 mg (93%)). Rf = 0.375 (silica gel, CH_2_Cl_2_/*Me*OH 19:1); IR = 1670, 1589, 1511, 1488, 1444, 1306, 1227, 1122, 813, 742, and 685; ^1^H NMR (CDCl_3_, 400 MHz) δ = 2.83–2.85 (m, 4H, N(CH_2_)_2_), 2.94–2.96 (m, 4H, N(CH_2_)_2_), 6.75–6.78 (m, 2H, 3′-H, 5′-H), 7.07–7.09 (m, 2H, 2′-H, 6′-H), 7.11 (t, *J* = 7.8 Hz, 1H, 4″-H), 7.18 (t, *J* = 7.8 Hz, 1H, 5″-H), 7.20 (d, *J* = 7.8 Hz, 1H, 3″-H), 7.58 (t, *J* = 7.8 Hz, 1H, 5-H), 7.76 (d, *J* = 7.8 Hz, 1H, 6-H), 7.89 (d, *J* = 7.8 Hz, 1H, 4-H), 8.33 (d, *J* = 7.8 Hz, 1H, 6″-H), and 9.10 (s, 1H, NH); ^13^C NMR (CDCl_3_, 100 MHz) δ = 46.51 (N(CH_2_)_2_), 53.40 (N(CH_2_)_2_), 116.13 (d, *J* = 22.2 Hz, C-3′, C-5′), 119.05 (C-6″), 121.00 (C-3″), 123.29 (q, *J* = 274 Hz, CF_3_), 124.27 (C-4″), 125.79 (C-5″), 128.39 (q, *J* = 5.6 Hz, C-4), 129.32 (C-5), 130.77 (d, *J* = 3.2 Hz, C-1′), 131.26 (C-2), 132.50 (d, *J* = 8.2 Hz, C-2′, C-6′), 132.80 (C-6), 133.41 (C-1″), 134.19 (q, *J* = 29.5 Hz, C-3), 141.43 (C-2″), 144.40 (C-1), 161.96 (d, *J* = 248 Hz, C-4′), and 164.80 (C=O); HRMS (ESI+) calculated for C_24_H_22_F_4_N_3_OS^+^ [M+H^+^]: 476.1414; found: 476.1404.

2-(4-Fluorophenoxy)-*N*-[3-(piperazin-1-yl)phenyl]-3-(trifluoromethyl)benzamide (**56**): Reaction of compound **38** (99 mg (0.22 mmol)) with trifluoroacetic acid (301 mg (2.64 mmol)) in dry dichloromethane (6 mL)) gave the protonated form of **56**. Work-up with a solution of potassium carbonate (186 mg (1.35 mmol)) in aqua demin yielded pure compound **56** as a pale-yellow solid (70 mg (69%)). m.P. 153 °C; Rf = 0.200 (silica gel, CH/EtAc 2:1); IR = 3418, 1661, 1607, 1501, 1450, 1310, 1221, 1136, 833, 778, and 688; ^1^H NMR (CDCl3, 400 MHz) δ = 3.00–3.03 (m, 4H, N(CH_2_)_2_), 3.11–3.14 (m, 4H, N(CH_2_)_2_), 6.66–6.71 (m, 2H, 4″-H‚ 6″-H), 6.75–6.79 (m, 2H, 2′-H, 6′-H), 6.91–6.95 (m, 2H, 3′-H, 5′-H), 7.12 (t, *J* = 2.2 Hz, 1H, 2″-H), 7.15 (t, *J* = 8.1 Hz, 1H, 5″-H), 7.54 (t, *J* = 7.9 Hz, 1H, 5-H), 7.90 (dd, *J* = 7.9, 1.7 Hz, 1H, 4-H), 8.29 (dd, *J* = 7.9, 1.7 Hz, 1H, 6-H), and 8.43 (br s, 1H, NH); ^13^C NMR (CDCl_3_, 100 MHz) δ = 45.92 ((NCH_2_)_2_), 49.87 ((NCH_2_)_2_), 108.01 (C-2″), 111.43 (C-6″), 112.60 (C-4″), 116.18 (d, *J* = 8.3 Hz, C-2′, C-6′), 116.57 (d, *J* = 23.8 Hz, C-3′, C-5′), 122.67 (q, *J* = 273 Hz, CF_3_), 125.23 (q, *J* = 31.8 Hz, C-3), 126.33 (C-5), 129.46 (C-5″), 130.61 (q, *J* = 5.0 Hz, C-4), 130.94 (C-1), 135.90 (C-6), 138.03 (C-1″), 149.40 (q, *J* = 1.8 Hz, C-2), 152.30 (C-3″), 154.03 (d, *J* = 2.5 Hz, C-1′), 158.50 (d, *J* = 242 Hz, C-4′), and 161.57 (C=O); HRMS (ESI+) calculated for C_24_H_22_F_4_N_3_O_2_^+^ [M+H]^+^: 460.1648; found: 460.1638.

2-(4-Fluorophenoxy)-*N*-[4-(piperazin-1-yl)phenyl]-3-(trifluoromethyl)benzamide (**57**): Reaction of compound **39** (602 mg (1.08 mmol)) with trifluoroacetic acid (1477 mg (12.96 mmol)) in dry dichloromethane (15 mL) gave the protonated form of **57**. Work-up with a solution of potassium carbonate (909 mg (6.58 mmol)) in aqua demin yielded the crude product. It was purified by column chromatography (silica gel, CH/EtAc 1.5:1) followed by extraction of the organic phases with aq NaHCO_3_, giving compound **57** as a pale-yellow solid (89 mg (18%)). m.P. 204 °C; Rf = 0.138 (silica gel, EtAc/*Me*OH 1:1); IR = 3424, 1663, 1595, 1516, 1501, 1449, 1317, 1238, 1214, 1170, 1131, 820, 778, and 687; ^1^H NMR (CDCl_3_, 400 MHz) δ = 3.00–3.04 (m, 4H, N(CH_2_)_2_), 3.07–3.11 (m, 4H, N(CH_2_)_2_), 6.75–6.79 (m, 2H, 2′-H, 6′-H), 6.83 (d, *J* = 8.9 Hz, 2H, 3″-H, 5″-H), 6.91–6.96 (m, 2H, 3′-H, 5′-H), 7.22 (d, *J* = 8.9 Hz, 2H, 2″-H, 6″-H), 7.52 (t, *J* = 7.8 Hz, 1H, 5-H), 7.88 (dd, *J* = 7.8, 1.7 Hz, 1H, 4-H), 8.29 (dd, *J* = 7.9, 1.7 Hz, 1H, 6-H), and 8.36 (br s, 1H, NH); ^13^C NMR (CDCl_3_, 100 MHz) δ = 46.08 ((NCH_2_)_2_), 50.52 ((NCH_2_)_2_), 116.17 (d, *J* = 8.3 Hz, C-2′, C-6′), 116.40 (C-3″, C-5″), 116.52 (d, *J* = 24.0 Hz, C-3′, C-5′), 121.72 (C-2″, C-6″), 122.70 (q, *J* = 273 Hz, CF_3_), 125.15 (q, *J* = 31.7 Hz, C-3), 126.24 (C-5), 129.33 (C-1″), 130.43 (q, *J* = 5.0 Hz, C-4), 130.97 (C-1), 135.89 (C-6), 149.29 (C-4″), 149.39 (q, *J* = 1.9 Hz, C-2), 154.02 (d, *J* = 2.5 Hz, C-1′), 158.47 (d, *J* = 242 Hz, C-4′), and 161.37 (C=O); HRMS (ESI+) calculated for C_24_H_22_F_4_N_3_O_2_^+^ [M+H]^+^: 460.1648; found: 460.1638.

2-Phenoxy-*N*-[4-(piperazin-1-yl)phenyl]-3-(trifluoromethyl)benzamide (**58**): Reaction of compound **40** (59 mg (0.11 mmol)) with trifluoroacetic acid (376 mg (3.30 mmol)) in dry dichloromethane (10 mL) for 48 h gave the protonated form of **58**. Work-up with a solution of potassium carbonate (365 mg (2.64 mmol)) in aqua demin gave pure compound **58** as a pale-yellow solid (45 mg (92%)). m.P. 149 °C; Rf = 0.163 (silica gel, CH_2_Cl_2_/*Me*OH 19:1); IR = 3418, 1660, 1516, 1449, 1316, 1237, 1137, and 751; ^1^H NMR (CDCl_3_, 400 MHz) δ = 3.02–3.05 (m, 4H, N(CH_2_)_2_), 3.09–3.12 (m, 4H, N(CH_2_)_2_), 6.80–6.83 (m, 4H, 2′-H, 3″-H, 5″-H, 6′-H), 7.03 (t, *J* = 7.4 Hz, 1H, 4′-H), 7.18–7.27 (m, 4H, 2″-H, 3′-H, 5′-H, 6″-H), 7.52 (t, *J* = 7.9 Hz, 1H, 5-H), 7.89 (dd, *J* = 7.9, 1.6 Hz, 1H, 4-H), 8.33 (dd, *J* = 7.9, 1.6 Hz, 1H, 6-H), and 8.47 (s, 1H, NH); ^13^C NMR (CDCl_3_, 100 MHz) δ = 45.81 (N(CH_2_)_2_), 50.24 (N(CH_2_)_2_), 114.95 (C-2′, C-6′), 116.53 (C-3″, C-5″), 121.88 (C-2″, C-6″), 122.74 (q, *J* = 273 Hz, CF_3_), 123.32 (C-4′), 125.27 (q, *J* = 31.7 Hz, C-3), 126.12 (C-5), 129.67 (C-1″), 130.00 (C-3′, C-5′), 130.48 (q, *J* = 4.9 Hz, C-4), 130.88 (C-1), 135.95 (C-6), 149.03 (C-4″), 149.31 (q, *J* = 1.7 Hz, C-2), 158.05 (C-1′), and 161.43 (C=O); HRMS (ESI+) calculated for C_24_H_23_F_3_N_3_O_2_^+^ [M+H]^+^: 442.1742; found: 442.1745.

2-(4-Fluorophenoxy)-*N*-[4-(piperazin-1-yl)phenyl]benzamide (**59**): Reaction of compound **42** (50 mg (0.10 mmol)) with trifluoroacetic acid (279 mg (2.45 mmol)) in dry dichloromethane (13 mL)) gave the protonated form of **59**. Work-up with a solution of potassium carbonate (88 mg (0.64 mmol)) in aqua demin yielded pure compound **59** as a pale-brown solid (29 mg (73%)). m.P. 175 °C; Rf = 0.175 (silica gel, CH_2_Cl_2_/*Me*OH 19:1); IR = 3373, 2941, 1650, 1591, 1517, 1501, 1476, 1450, 1320, 1239, 1207, 1128, 1092, 822, 780, and 655; ^1^H NMR (CDCl_3_, 400 MHz) δ = 3.01–3.13 (m, 8H, 2 N(CH_2_)_2_), 6.83 (dd, *J* = 8.1, 1.1 Hz, 1H, 3-H), 6.88–6.92 (m, 2H, 3″-H, 5″-H), 7.06–7.13 (m, 4H, 2′-H, 3′-H, 5′-H, 6′-H), 7.25 (td, *J* = 8.0, 1.1 Hz, 1H, 5-H), 7.41 (td, *J* = 8.0, 1.7 Hz, 1H, 4-H), 7.48–7.53 (m, 2H, 2″-H, 6″-H), 8.32 (dd, *J* = 8.0, 1.7 Hz, 1H, 6-H), and 9.40 (s, 1H, NH); ^13^C NMR (CDCl_3_, 100 MHz) δ = 46.08 (N(CH_2_)_2_), 50.83 (N(CH_2_)_2_), 116.70 (C-3″, C-5″), 116.94 (d, *J* = 23.6 Hz, C-3′, C-5′), 117.86 (C-3), 121.12 (d, *J* = 8.4 Hz, C-2′, C-6′), 121.69 (C-2″, C-6″), 124.00 (C-5), 124.21 (C-1), 130.64 (C-1″), 132.52 (C-6), 132.92 (C-4), 148.93 (C-4″), 151.13 (d, *J* = 2.7 Hz, C-1′), 155.42 (C-2), 159.64 (d, *J* = 244 Hz, C-4′), and 162.31 (C=O); HRMS (ESI+) calculated for C_23_H_23_FN_3_O_2_^+^ [M+H]^+^: 392.1774; found: 392.1775.

3-Fluoro-2-(4-fluorophenoxy)-*N*-[4-(piperazin-1-yl)phenyl]benzamide (**60**): Reaction of compound **43** (34 mg (0.07 mmol)) with trifluoroacetic acid (229 mg (2.01 mmol)) in dry dichloromethane (8 mL) gave the protonated form of **60**. Work-up with a solution of potassium carbonate (185 mg (1.34 mmol)) yielded compound **60** as a pale yellow solid (24 mg (84%)). m.P. 180 °C; Rf = 0.175 (silica gel, CH_2_Cl_2_/*Me*OH 19:1); IR = 3370, 1654, 1586, 1502, 1461, 1321, 1270, 1236, 1213, 1184, 1129, 880, 823, 795, and 765; ^1^H NMR (CDCl_3_, 400 MHz) δ = 3.01–3.04 (m, 4H, N(CH_2_)_2_), 3.08–3.11 (m, 4H, N(CH_2_)_2_), 6.87 (d, *J* = 8.9 Hz, 2H, 3″-H, 5″-H), 6.93–6.97 (m, 2H, 2′-H, 6′-H), 6.99–7.04 (m, 2H, 3′-H, 5′-H), 7.29–7.37 (m, 2H, 4-H, 5-H), 7.40 (d, *J* = 8.9 Hz, 2H, 2″-H, 6″-H), 8.04–8.07 (m, 1H, 6-H), and 9.00 (s, 1H, NH); ^13^C NMR (CDCl_3_, 100 MHz) δ = 46.02 (N(CH_2_)_2_), 50.63 (N(CH_2_)_2_), 116.59 (d, *J* = 23.6 Hz, C-3′, C-5′), 116.59 (C-3″, C-5″), 116.73 (d, *J* = 8.3 Hz, C-2′, C-6′), 120.20 (d, *J* = 18.5 Hz, C-4), 121.54 (C-2″, C-6″), 126.24 (d, *J* = 7.6 Hz, C-5), 127.09 (d, *J* = 3.3 Hz, C-6), 129.45 (C-1), 130.05 (C-1″), 140.40 (d, *J* = 13.1 Hz, C-2), 149.11 (C-4″), 153.00 (t, *J* = 2.2 Hz, C-1′), 154.99 (d, *J* = 252 Hz, C-3), 158.85 (d, *J* = 243 Hz, C-4′), and 161.13 (d, *J* = 3.2 Hz, C=O); HRMS (ESI+) calculated for C_23_H_22_F_2_N_3_O_2_^+^ [M+H^+^]: 410.1675; found: 410.1670.

2-(4-Fluorophenoxy)-3-nitro-*N*-[4-(piperazin-1-yl)phenyl]benzamide (**61**): Reaction of compound **44** (155 mg (0.29 mmol)) with trifluoroacetic acid (791 mg (6.94 mmol)) in dry dichloromethane (16 mL) gave the protonated form of **61**. Work-up with a solution of potassium carbonate (239 mg (1.73 mmol)) in aqua demin yielded pure compound **61** as a yellow solid (81 mg (64%)). m.P. 224 °C; Rf = 0.188 (silica gel, CH_2_Cl_2_/*Me*OH 19:1); IR = 3416, 2828, 1651, 1603, 1528, 1499, 1446, 1358, 1237, 1185, 883, 818, and 773; ^1^H NMR (DMSO-d_6_, 400 MHz) δ = 2.80–2.83 (m, 4H, N(CH_2_)_2_), 2.96–2.99 (m, 4H, N(CH_2_)_2_), 6.83 (d, *J* = 8.8 Hz, 2H, 3″-H, 5″-H), 6.84–6.88 (m, 2H, 2′-H, 6′-H), 7.07–7.12 (m, 2H, 3′-H, 5′-H), 7.29 (d, *J* = 8.8 Hz, 2H, 2″-H, 6″-H), 7.61 (t, *J* = 7.9 Hz, 1H, 5-H), 7.95 (dd, *J* = 7.7, 1.3 Hz, 1H, 6-H), 8.19 (dd, J = 8.1, 1.3 Hz, 1H, 4-H), and 10.25 (s, 1H, NH); ^13^C NMR (DMSO-d_6_, 100 MHz) δ = 45.51 (N(CH_2_)_2_), 49.57 (N(CH_2_)_2_), 115.35 (C-3″, C-5″), 116.09 (d, *J* = 23.6 Hz, C-2′, C-6′), 117.35 (d, *J* = 8.4 Hz, C-3′, C-5′), 120.85 (C-2″, C-6″), 126.34 (C-5), 126.70 (C-4), 130.08 (C-1″), 133.88 (C-1), 134.25 (C-6), 143.43 (C-3), 143.98 (C-2), 148.36 (C-4″), 153.61 (d, *J* = 2.2 Hz, C-1′), 157.68 (d, *J* = 239 Hz, C-4′), and 161.61 (C=O); HRMS (ESI+) calculated for C_23_H_22_FN_4_O_4_^+^ [M+H]^+^: 437.1625; found: 437.1627.

2-[(4-Fluorophenyl)sulfanyl]-*N*-[4-(piperazin-1-yl)phenyl]-3-(trifluoromethyl)benz-amide (**62**): Reaction of compound **45** (35 mg (0.06 mmol)) with trifluoroacetic acid (213 mg (1.87 mmol)) in dry dichloromethane (5 mL) gave the protonated form of **62**. Work-up with a solution of potassium carbonate (175 mg (1.27 mmol)) in aqua demin yielded pure compound **62** as a colorless solid (25 mg (97%)). m.P. 214 °C; Rf = 0.138 (silica gel CH_2_Cl_2_/*Me*OH 19:1); IR = 1646, 1517, 1309, 1217, 1122, 817, and 679; ^1^H NMR (CDCl_3_, 400 MHz) δ = 3.03–3.06 (m, 4H, N(CH_2_)_2_), 3.11–3.14 (m, 4H, N(CH_2_)_2_), 6.82–6.90 (m, 4H, 3′-H, 3″-H, 5′-H, 5″-H), 7.06–7.11 (m, 2H, 2′-H, 6′-H), 7.26–7.29 (m, 2H, 2″-H, 6″-H), 7.58 (t, *J* = 7.9 Hz, 1H, 5-H), 7.72 (br s, 1H, NH), 7.83 (dd, *J* = 7.8, 1.4 Hz, 1H, 6-H), and 7.88 (dd, *J* = 8.0, 1.4 Hz, 1H, 4-H); ^13^C NMR (CDCl_3_, 100 MHz) δ = 46.03 (N(CH_2_)_2_), 50.55 (N(CH_2_)_2_), 116.36 (d, *J* = 22.3 Hz, C-3′, C-5′), 116.53 (C-3″, C-5″), 120.95 (C-2″, C-6″), 123.26 (q, *J* = 274 Hz, CF_3_), 128.50 (q, *J* = 5.6 Hz, C-4), 129.48 (C-5), 129.80 (C-1″), 130.36 (C-2), 130.92 (d, *J* = 3.5 Hz, C-1′), 131.68 (d, *J* = 8.2 Hz, C-2′, C-6′), 133.65 (C-6), 134.28 (q, *J* = 29.6 Hz, C-3), 143.89 (C-1), 149.06 (C-4″), 161.94 (d, *J* = 248 Hz, C-4′), and 164.45 (C=O); HRMS (ESI+) calculated for C_24_H_22_F_4_N_3_OS^+^ [M+H^+^]: 476.1414; found: 476.1404.

2-(4-Acetamidophenoxy)-*N*-[4-(piperazin-1-yl)phenyl]-3-(trifluoromethyl)benzamide (**63**): Reaction of compound **47** (35 mg (0.06 mmol)) with trifluoroacetic acid (200 mg (1.75 mmol)) in dry dichloromethane (6 mL) gave the protonated form of **63**. Work-up with a solution of potassium carbonate (162 mg (1.17 mmol)) in aqua demin yielded pure compound **63** as a pale-yellow solid (23 mg (79%)). m.P. 213 °C; Rf = 0.125 (silica gel, CH_2_Cl_2_/*Me*OH 19:1); IR = 1656, 1503, 1445, 1231, 1126, and 821; ^1^H NMR (MeOD, 400 MHz) δ = 2.09 (s, 3H, CH_3_), 2.97–3.00 (m, 4H, N(CH_2_)_2_), 3.09–3.12 (m, 4H, N(CH_2_)_2_), 6.78 (d, *J* = 8.5 Hz, 2H, 2′-H, 6′-H), 6.87 (d, *J* = 8.5 Hz, 2H, 3″-H, 5″-H), 7.16 (d, *J* = 8.5 Hz, 2H, 2″-H, 6″-H), 7.40 (d, *J* = 8.5 Hz, 2H, 3′-H, 5′-H), 7.54 (t, *J* = 7.9 Hz, 1H, 5-H), 7.89 (dd, *J* = 7.9, 1.6 Hz, 1H, 6-H), and 7.94 (dd, *J* = 7.9, 1.6 Hz, 1H, 4-H); ^13^C NMR (MeOD, 100 MHz) δ = 23.96 (CH_3_), 46.72 (N(CH_2_)_2_), 51.56 (N(CH_2_)_2_), 117.84 (C-2′, C-6′), 117.87 (C-3″, C-5″), 122.14 (q, *J* = 272 Hz, CF_3_), 122.79 (C-3′, C-5′), 123.56 (C-2″, C-6″), 126.04 (q, *J* = 30.0 Hz, C-3), 126.76 (C-5), 130.55 (q, *J* = 4.9 Hz, C-4), 131.89 (C-1″), 133.87 (C-1), 135.40 (C-4′), 135.57 (C-6), 150.70 (C-4″), 152.09 (q, *J* = 1.8 Hz, C-2), 156.21 (C-1′), 165.82 (ArC=O), and 171.64 (CH_3_C=O); HRMS (ESI+) calculated for C_26_H_26_F_3_N_4_O_3_^+^ [M+H^+^]: 499.1952; found: 499.1941.

2-Phenoxy-*N*-[4-(piperazin-1-yl)phenyl]benzamide (**64**): Reaction of **48** (200 mg (0.42 mmol)) with trifluoroacetic acid (1437 mg (12.60 mmol)) in dry dichloromethane (8 mL) gave the protonated form of **64**. Work-up with a solution of potassium carbonate (1160 mg (8.40 mmol)) in aqua demin yielded the crude product. It was purified by column chromatography (aluminum oxide basic, CH_2_Cl_2_/EtAc/*Me*OH 37:2:1), giving compound **64** as a pale-yellow solid (50 mg (31%)). m.P. 173 °C; Rf = 0.275 (silica gel, CH_2_Cl_2_//EtAc/*Me*OH 37:2:1); IR = 3371, 1650, 1593, 1516, 1489, 1449, 1320, 1214, 796, and 751; ^1^H NMR (CDCl_3_, 400 MHz) δ = 3.02–3.06 (m, 4H, N(CH_2_)_2_), 3.09–3.13 (m, 4H, N(CH_2_)_2_), 6.86–6.92 (m, 3H, 3-H, 3″-H, 5″-H), 7.11 (d, *J* = 7.7 Hz, 2H, 2′-H, 6′-H), 7.20–7.27 (m, 2H, 4′-H, 5-H), 7.38–7.44 (m, 3H, 3′-H, 4-H, 5′-H), 7.48–7.52 (m, 2H, 2″-H, 6″-H), 8.33 (dd, *J* = 7.9, 1.7 Hz, 1H, 6-H), and 9.49 (s, 1H, NH); ^13^C NMR (CDCl_3_, 100 MHz) δ = 46.02 (N(CH_2_)_2_), 50.75 (N(CH_2_)_2_), 116.72 (C-3″, C-5″), 118.46 (C-3), 119.46 (C-2′, C-6′), 121.65 (C-2″, C-6″), 123.95 (C-5), 124.31 (C-1), 124.83 (C-4′), 130.27 (C-3′, C-5′), 130.77 (C-1″), 132.42 (C-6), 132.86 (C-4), 148.78 (C-4″), 155.13 (C-2), 155.40 (C-1′), and 162.37 (C=O); HRMS (ESI+) calculated for C_23_H_24_N_3_O_2_^+^ [M+H^+^]: 374.1863 found: 374.1858.

*N*-[4-(Piperazin-1-yl)phenyl]-3-(trifluoromethyl)benzamide (**66**): Reaction of compound **67** (149 mg (0.33 mmol)) with trifluoroacetic acid (1137 mg (9.97 mmol)) in dry dichloromethane (6 mL) gave the protonated form of **66**. Work-up with a solution of potassium carbonate (924 mg (6.69 mmol)) in aqua demin yielded pure compound **66** as a yellow solid (107 mg (92%)). m.P. 147 °C; Rf = 0.138 (silica gel, CH_2_Cl_2_/*Me*OH 19:1); IR = 1641, 1520, 1330, 1256, 1228, 1114, 1071, 938, 905, 811, 790, and 695; ^1^H NMR (DMSO-d_6_, 400 MHz) δ = 2.48 (br, 4H, N(CH_2_)_2_), 3.01–3.04 (m, 4H, N(CH_2_)_2_), 6.93 (d, *J* = 8.5 Hz, 2H, 3″-H, 5″-H), 7.61 (d, *J* = 8.5 Hz, 2H, 2″-H, 6″-H), 7.78 (t, *J* = 7.8 Hz, 1H, 5-H), 7.95 (br d, *J* = 7.8 Hz, 1H, 4-H), 8.26 (br d, *J* = 7.8 Hz, 1H, 6-H), 8.29 (br s, 1H, 2-H), and 10.28 (s, 1H, NH); ^13^C NMR (DMSO-d_6_, 100 MHz) δ = 45.59 (N(CH_2_)_2_), 49.68 (N(CH_2_)_2_), 115.34 (C-3″, C-5″), 121.64 (C-2″, C-6″), 124.00 (q, *J* = 273 Hz, CF_3_), 124.06 (q, *J* = 3.9 Hz, C-2), 127.84 (q, *J* = 3.8 Hz, C-4), 129.06 (q, *J* = 32.0 Hz, C-3), 129.63 (C-5), 130.38 (C-1″), 131.65 (C-6), 135.95 (C-1), 148.44 (C-4″), and 163.31 (C=O); HRMS (ESI+) calculated for C_18_H_19_F_3_N_3_O^+^ [M+H^+^]: 350.1475 found: 350.1468.

### 3.3. Biological Tests

#### 3.3.1. *In Vitro* Microplate Assay Against *P. falciparum* NF54

The *in vitro* activity of compounds against erythrocytic stages of the drug-sensitive NF54 strain of *P. falciparum*, originating from Thailand, was determined using a ^3^H-hypoxanthine incorporation assay [38,39,40]. Compounds were dissolved in DMSO at 10 mg/mL and further diluted in medium before adding to parasite cultures that were incubated in RPMI 1640 medium without hypoxanthine, supplemented with HEPES (5.94 g/L), NaHCO_3_ (2.1 g/L), neomycin (100 U/mL), AlbumaxR (5 g/L), and washed human red blood cells A+ at 2.5% hematocrit (0.3% parasitemia). Serial drug dilutions of eleven 3-fold dilution steps covering a range from 100 to 0.002 µg/mL were prepared. The 96-well plates were incubated in a humidified atmosphere at 37 °C, 4% CO_2_, 3% O_2_, and 93% N_2_. After 48 h of incubation time, 0.05 mL of ^3^H-hypoxanthine (=0.5 µCi) was added to each well of the plate. The plates were incubated for a further 24 h under the same conditions. Plates were then harvested using a BetaplateTM cell harvester (Wallac, Zurich, Switzerland). Red blood cells were transferred onto a glass fiber filter and then washed with distilled aqua demin. The dried filters were inserted into a plastic foil with 10 mL of scintillation fluid and counted in a BetaplateTM liquid scintillation counter (Wallac, Zurich, Switzerland). IC_50_ values were calculated from sigmoidal inhibition curves by linear regression using Microsoft Excel [41]. Chloroquine (Sigma C6628, St. Louis, MO, USA) was used as control. Results are presented in Table 1.

#### 3.3.2. *In Vitro* Microplate Assay Against a Selection of ESKAPE Pathogens (Commissioned Work Performed by the Antimicrobial Screening Facility at the University of Warwick, UK)

Minimum inhibitory concentrations (MIC values) were obtained using the microbroth dilution method as stated in the EUCAST guidelines. Tests were conducted on cation adjusted Mueller–Hinton broth (caMHB) or agar (caMHA). These were prepared and stored following the manufacturer’s guidelines. Biological organisms were prepared via growth on either Luria–Bertani (LB) or cation adjusted Mueller–Hinton agar plates. These were grown for 24 h at 37 °C, except for *Micrococcus luteus* where MICs took longer to grow and as a consequence were read after 48 h once a clear positive control could be observed. For the MIC experiment, the bacteria were prepared using the McFarland 0.5 standard and diluted 1 in 100 or 1 in 10 in caMHB following EUCAST and CSLI guidelines for the preparation of bacteria. Testing of the compounds using the microbroth dilution method took place in 96-well plates. Serial 2-fold dilutions of compounds in caMHB covering a range from 256 µg/mL to 0.25 µg/mL were prepared. Bacteria preparations (McFarland) were added to the compound dilutions, further halving the compound concentrations. The plate was then incubated for 18 h at 37 °C. Controls for the MIC experiments included a bacterial growth (positive) control of bacteria and caMHB only and a media only (negative) control. After incubation, the plates were read, whereby the positive control of bacteria was visible and the negative control was clear of contamination. The last clear well, where there is no growth of bacteria, was counted as the MIC value. For the minimum bactericidal concentrations (MBC values) an aliquot from each test well (including controls) was pipetted onto caMHA plates and incubated at 37 °C for 24 h. The lowest concentration without any bacterial growth was counted as the MBC value. The following pathogens were used: *Pseudomonas aeruginosa* NCTC 13437, *Escherichia coli* NCTC 13353, *Bacillus subtilis* 168a, *Serratia marcescens* NCTC 9940, *Micrococcus luteus* ATCC 10240, *Staphylococcus aureus* JE2 USA 300, *Acinetobacter baumannii* ATCC 19606, and *Klebsiella pneumoniae* ATCC 700603. A concentration range of 256 µg/mL to 0.25 µg/mL of a combination of ciprofloxacin and meropenem against E. coli 25922 was used as an antibiotic control to ensure correct dilution and performance of the assay. Results are presented in Table 2.

#### 3.3.3. Resazurin-Based *In Vitro* Cytotoxicity with L-6 Cells

The cytotoxicity assays were performed using 96-well microtiter plates, each well containing 4000 L-6 cells (a primary cell line derived from rat skeletal myofibroblasts, ATCC CRL-1458TM) in 0.1 mL of RPMI 1640 medium supplemented with 1% glutamine (200 mM) and 10% fetal bovine serum [42,43]. Serial drug dilutions of eleven 3-fold dilution steps covering a range from 100 to 0.002 µg/mL were prepared. After 70 h of incubation, the plates were inspected under an inverted microscope to assure growth of the controls and sterile conditions. Then, 0.01 mL resazurin solution (resazurin, 12.5 mg in 100 mL double-distilled aqua demin) was added to each well and the plates were incubated for another 2 h. The plates were read with a Spectramax Gemini XS microplate fluorometer (Molecular Devices Cooperation, Sunnyvale, CA, USA) using an excitation wavelength of 536 nm and an emission wavelength of 588 nm. IC_50_ values were calculated by linear regression from the sigmoidal dose–inhibition curves using SoftmaxPro software Version 8.2.1 (Molecular Devices Cooperation, Sunnyvale, CA, USA) [41]. Podophyllotoxin (Sigma P4405) was used as control. Results are presented in Table 1.

#### 3.3.4. Resazurin-Based *In Vitro* Cytotoxicity with HepG2 Cells (Commissioned Work Performed by Bienta, Kyiv, Ukraine)

The HepG2 cells were washed with DPBS (Dulbecco’s PBS without calcium and magnesium, Gibco, Cat #21600-044) and trypsinized with trypsin solution in DPBS. Trypsinization was stopped by adding culture medium, and after being stained with Trypan Blue (SORS Ukraine) cells were counted using a counting chamber (Hausser Scientific, Cat #3500). The cell suspension was placed in a falcon tube containing plating medium DMEM/High glucose with 1% fetal bovine serum, 100 units/mL penicillin, and 100 µg/mL streptomycin. Cells at a density of 250,000 cells/mL were seeded in sterile 384-well plates (Greiner Bio-One, Cat #781091) in a volume of 5000 cells per well and incubated at 37 °C and 5% CO_2_. Serial dilutions of ten 3-fold dilution steps of test and reference compounds covering a range from 100.0 to 0.005 µM were added to the wells, followed by incubation at 37 °C and 5% CO_2_ for 48 h. Then, resazurin (50 µM final concentration) was added and plates were incubated for another 3 h. The plates were read with a SpectraMax microplate reader (Molecular Devices Cooperation, Sunnyvale, CA, USA)) using an extinction wavelength of 555 nm and an emission wavelength of 570 nm. IC_50_ values were calculated using GraphPad Prism 9.0 charting software [44,45,46]. Doxorubicin (Sigma Aldrich D1515) was used as control. Results are presented in Table 1.

#### 3.3.5. Parallel Artificial Membrane Permeability Assay (PAMPA)

With the high-throughput PAMPA the newly synthesized compounds were tested for their passive permeability through cell membranes without the influence of efflux pumps or transporter proteins. The assay was performed using a Corning^®^ Gentest^TM^ Precoated PAMPA Plate System (Corning, Glendale, AZ, USA) with 96-well polystyrene plates. The bottom of the acceptor plate consists of a porous membrane, whereby the pores are lined with a lipid–oil–lipid triple layer. Stock solutions of each test compound at 10 mM were prepared in DMSO or methanol and diluted with phosphate-buffered saline (PBS at a pH of 7.4) to a final concentration of 200 µM. Hydrochlorothiazide (Pe = 0.9 nm/s) and caffeine (Pe = 80 nm/s) were used as standards. The donor plate (bottom plate) was filled with the compound solutions, whereby all compounds were tested in quadruplicates. Each well of the acceptor plate (top plate) was filled with PBS buffer. Donor and acceptor plates were combined and incubated at room temperature for 5 h. After that, the plates were separated and 150 µL of each well of both plates were transferred to 96-well UV plates (Greiner Bio-One). Absorption at different wavelengths covering a range from 200 to 300 nm was measured using a SpectraMax M3 UV plate reader. By measuring serial dilutions of five dilution steps covering a range from 200 to 12.5 µM, calibration curves were prepared for each compound. The plates were analyzed at the wavelength where the R2 value of the calibration curve was higher than 0.99 [47]. Effective permeability, Pe, of each test compound was calculated using the following Equations (1)–(3) and are presented in Table 3:

(1) $$Pe=\frac{-ln[1-\frac{c_{A}\left(t\right)}{c_{equ}}]}{S\times \left(\frac{1}{V_{D}}+\frac{1}{V_{A}}\right)\times t}$$

where

*Pe*—effective permeability;

*S*—filter area (0.3 cm^2^);

*V_D_*—donor well volume (0.3 mL);

*V_A_*—acceptor well volume (0.2 mL);

*t*—incubation time (18,000 s);

*c_A_*(*t*)—acceptor well compound concentration at time *t*;

*c_equ_*—equilibrium concentration.

(2) $$c_{equ}=\frac{{[c}_{D}\left(t\right)\times V_{D}+c_{A}\left(t\right)\times V_{A}]}{\left(V_{D}+V_{A}\right)}$$

where

*V_D_*—donor well volume (0.3 mL);

*V_A_*—acceptor well volume (0.2 mL);

*c_A_*(*t*)*—*acceptor well compound concentration at time t;

*c_D_*(*t*)*—*donor well compound concentration at time t.

Recovery of compounds from donor and acceptor wells (mass retention) was calculated as shown in the equation below. Data were only accepted when recovery exceeded 70%. The equation is as follows:

(3) $$R=1-\frac{\left[c_{D}\left(t\right)\times V_{D}+c_{A}\left(t\right)\times V_{A}\right]}{\left(c_{0}\times V_{D}\right)}$$

where

*R*—mass retention (%);

*V_D_*—donor well volume (0.3 mL);

*V_A_*—acceptor well volume (0.2 mL);

*c_A_*(*t*)—acceptor well compound concentration at time t;

*c_D_*(*t*)—donor well compound concentration at time t;

*c*_0_—initial donor well compound concentration (200 µM).

### 3.4. Determination of In Silico ADME Parameters

#### Ligand Efficiency (LE)

Ligand efficiency was calculated as shown in the following Equation (4). Results are presented in Table 3 [22]:

(4) $$LE=\frac{1.37}{HA}\times {pIC}_{50}$$

where

LE—ligand efficiency;

HA—number of heavy atoms;

pIC_50_—negative logarithm of IC_50_.

## 4. Conclusions

**Figure 9:**
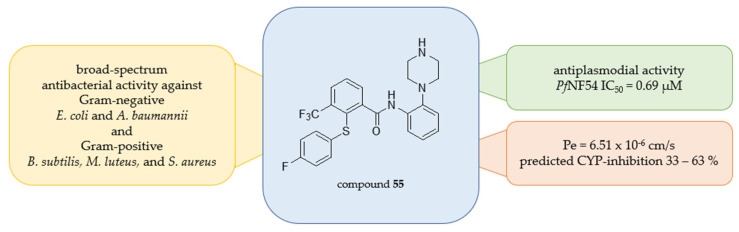
Highlights of the most promising compound **55**.

This paper deals with the preparation of a series of derivatives of MMV’s Malaria Box compound **MMV030666** (**37**), a 2-(4-fluorophenoxy)-3-(trifluoromethyl)benzanilide with a 2′-(4-Boc-piperazinyl) group. When the anilide moiety was replaced by tertiary amides the antiplasmodial activity dropped heavily. Cleavage of the Boc group led to decreased antiplasmodial activity and increased cytotoxicity, whereas its replacement with a pyrrolidino substituent gave the most promising antiplasmodial compound **34** of the new series, showing good antiplasmodial activity (*P. falciparum* NF54 IC_50_ = 1.68 µM) and low cytotoxicity (L-6 IC_50_ = 185 µM, HepG2 IC_50_ = 39.8 µM). The 2-phenoxy-*N*-(4′-piperazinyl)benzanilide **64** exhibited activity against Gram-positive bacteria *M. luteus* and *S. aureus* (MIC = 32 µg/mL, MBC = 64 µg/mL). The more toxic 2-[(4-fluorophenyl)sulfanyl]-*N*-(2′-piperazinyl)-3-(trifluoromethyl)benzanilide **55** showed the highest antiplasmodial properties (*Pf*NF54: IC_50_ = 0.692 µM) and broad-spectrum antibacterial and bactericidal activity against Gram-negative *A. baumannii* and *E. coli* (MIC = 32–64 µg/mL; MBC = 64 µg/mL) as well as Gram-positive *M. luteus*, *B. subtilis*, and *S. aureus* (MIC = 16–64 µg/mL, MBC = 16–64 µg/mL). Furthermore, it exhibits encouraging permeability of 6.51 × 10^−6^ cm/s and is predicted to moderately inhibit the most important CYP enzymes (Figure 9). Subsequent testing of the most promising compounds against multi-resistant bacterial strains is planned in future.

## Figures and Tables

**Table 1:** Activities of compounds **31**–**36** and **49**–**60** against *P. falciparum* NF54 ^a,b^, L-6 cells ^a^, and HepG2 cells, expressed as IC_50_ (µM). The most promising results are highlighted in green.

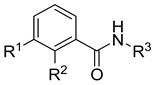								
Cp.	R^1^	R^2^	R^3^	*P.f.*NF54 IC_50_ (µM)	S.I. = IC_50_ (Cyt. L-6)/ IC_50_ (*P.f.*NF54)	Cytotoxicity L-6 Cells IC_50_ (µM)	S.I. = IC_50_ (Cyt. HepG2)/IC_50_ (*P.f.*NF54)	Cytotoxicity HepG2 Cells IC_50_ (µM)
**31**	CF_3_	4F-phenoxy	2-(4-*Me*piperazinyl)phenyl	14.0	8.59	120	n.d.	n.d.
**32**	CF_3_	4F-phenoxy	2-morpholinophenyl	3.04	53.0	161	>32.9	>100
**33**	CF_3_	4F-phenoxy	4-morpholinophenyl	6.81	28.7	195	5.18	35.3
**34**	CF_3_	4F-phenoxy	4-pyrrolidinophenyl	1.68	110	185	23.7	39.8
**35**	CF_3_	4F-phenoxy	2-(dimethylamino)phenyl	4.40	5.70	25.1	n.d.	n.d.
**36**	CF_3_	4F-phenoxy	4-(dimethylamino)phenyl	7.58	31.5	239	>13.2	>100
**49**	CF_3_	4F-phenoxy	4-*Me*piperazinyl	55.2	2.48	137	>1.81	>100
**50**	CF_3_	4F-phenoxy	4-*Boc*-piperazinyl	51.8	0.80	41.4	0.68	35.2
**51**	CF_3_	4F-phenoxy	morpholino	81.1	1.89	154	>1.23	>100
**52**	CF_3_	4F-phenoxy	piperazinyl	23.2	5.85	136	>4.32	>100
**53**	CF_3_	4F-phenoxy	2-piperazinylphenyl	1.04	13.0	13.5	3.94	4.10
**54**	CF_3_	4-*Me*(CO)NHphenoxy	2-piperazinylphenyl	11.1	1.19	13.2	n.d.	n.d.
**55**	CF_3_	4F-Ph-S-	2-piperazinylphenyl	0.69	10.2	7.06	6.65	4.60
**56**	CF_3_	4F-phenoxy	3-piperazinylphenyl	5.45	6.27	34.2	2.33	12.7
**57**	CF_3_	4F-phenoxy	4-piperazinylphenyl	3.15	11.9	37.3	3.30	10.4
**58**	CF_3_	phenoxy	4-piperazinylphenyl	3.81	6.79	25.9	1.52	5.80
**59**	H	4F-phenoxy	4-piperazinylphenyl	2.04	12.5	25.4	19.1	38.9
**60**	F	4F-phenoxy	4-piperazinylphenyl	4.70	8.68	40.8	5.57	26.2
**61**	NO_2_	4F-phenoxy	4-piperazinylphenyl	4.10	10.7	43.8	7.27	29.8
**62**	CF_3_	4F-Ph-S-	4-piperazinylphenyl	1.66	7.88	13.1	5.59	9.30
**63**	CF_3_	4-*Me*(CO)NHphenoxy	4-piperazinylphenyl	15.7	7.24	113	n.d.	n.d.
**64**	H	phenoxy	4-piperazinylphenyl	2.33	17.4	40.6	5.28	12.3
**65**	NH_2_	4F-phenoxy	4-piperazinylphenyl	7.00	22.4	157	>14.3	>100
**66**	CF_3_	H	4-piperazinylphenyl	2.60	16.0	41.5	4.55	11.8
**CQ**				0.009	9672	90.92		
**POD**						0.012		
**DOX**								0.300

CQ = chloroquine; POD = podophyllotoxin; DOX = doxorubicin; ^a^ IC_50_ values represent the average of four determinations (two determinations in two independent experiments); ^b^, sensitive to chloroquine; n.d., not determined.

**Table 2:** MIC and MBC data ^a^ of compounds **49**, **51**, **52**, **55**–**62**, **64**, and **65** against 8 of the WHO priority pathogens, expressed as µg/mL. The most promising results are highlighted in green, moderate ones in yellow.

Cp.	*P. aeruginosa* NCTC 13437		*E. coli* NCTC 13353		*S. marcescens* NCTC 9940		*A. baumannii* ATCC 19606		*K. pneumoniae* ATCC 700603		*B. subtilis* 168a		*M. luteus* ATCC 10240		*S. aureus* JE2 USA 300	
	MIC	MBC	MIC	MBC	MIC	MBC	MIC	MBC	MIC	MBC	MIC	MBC	MIC	MBC	MIC	MBC
**49**	256	>256	256	>256	>256	>256	256	>256	>256	>256	256	>256	256	256	>256	>256
**51**	>256	>256	>256	>256	>256	>256	256	256	>256	>256	256	>256	256	128	>256	>256
**52**	>256	>256	>256	>256	>256	>256	256	>256	>256	>256	>256	>256	256	256	>256	>256
**55**	256	>256	64	64	256	>256	32	64	>256	>256	32	64	16	16	64	64
**56**	256	>256	256	>256	256	>256	128	256	>256	>256	128	256	64	64	256	256
**57**	>256	>256	>256	>256	>256	>256	128	256	128	128	128	256	64	128	128	128
**58**	>256	>256	>256	>256	>256	>256	256	>256	256	256	128	256	128	128	128	128
**59**	>256	>256	256	>256	>256	>256	256	256	128	128	128	128	64	64	128	128
**60**	>256	>256	>256	>256	>256	>256	>256	>256	>256	>256	256	256	64	128	>256	>256
**61**	>256	>256	>256	>256	>256	>256	256	>256	256	>256	256	>256	128	128	128	>256
**62**	>256	>256	>256	>256	>256	>256	256	>256	256	256	128	256	128	128	128	128
**64**	>256	>256	>256	>256	>256	>256	256	>256	256	>256	128	256	32	64	32	64
**65**	>256	>256	>256	>256	>256	>256	>256	>256	>256	>256	>256	>256	>256	>256	256	256

^a^ MIC and MBC values represent the average of two determinations.

**Table 3:** Key physicochemical properties and passive permeability of compounds **31**–**36** and **49**–**66**. HBD, HBA, log *p*, and log D_7.4_ as well as ligand efficiency (LE) values were calculated *in silico*, whilst passive permeability (Pe) was determined experimentally.

Compound	Rule of Five for Drug-Likeness				log D_7.4_ ^a^	LE *P.f.*NF54 (kcal/mol/HA)	Pe ^b^ (10^−6^ cm/s)
	MW (g/mol)	HBD ^a^	HBA ^a^	log *p* ^a^			
**31**	473.46	1	3	5.54	5.09	0.196	n.d.
**32**	460.42	1	3	5.48	5.48	0.229	6.63
**33**	460.42	1	3	5.48	5.48	0.215	3.92
**34**	444.15	1	2	6.10	6.10	0.247	n.d.
**35**	418.38	1	2	5.69	5.69	0.245	n.d.
**36**	418.38	1	2	5.69	5.69	0.234	n.d.
**49**	382.35	0	2	3.64	3.56	0.216	8.50
**50**	468.44	0	2	4.54	4.54	0.178	1.82
**51**	369.31	0	2	3.57	3.57	0.216	10.36
**52**	368.33	1	2	3.26	3.26	0.244	8.35
**53**	459.44	2	3	5.16	3.70	0.243	6.39
**54**	498.50	3	4	4.25	2.80	0.189	0.26
**55**	475.50	2	3	5.85	4.39	0.256	6.51
**56**	459.44	2	3	5.16	3.68	0.219	7.03
**57**	459.44	2	3	5.16	3.67	0.228	6.89
**58**	441.45	2	3	5.02	3.53	0.232	4.98
**59**	391.44	2	3	4.28	2.79	0.269	14.23
**60**	409.43	2	3	4.43	2.93	0.243	11.68
**61**	436.44	2	5	4.22	2.73	0.231	8.36
**62**	475.50	2	3	5.85	4.36	0.240	4.41
**63**	498.50	3	4	4.25	2.76	0.188	n.d.
**64**	373.45	2	3	4.14	2.65	0.276	7.67
**65**	406.45	3	4	3.45	1.96	0.235	5.76
**66**	349.35	2	3	3.52	2.03	0.306	3.91
hydrochlorothiazide							0.09
caffeine							8.00

^a^ HBD, HBA, log *p*, and log D_7.4_ values were calculated using the ChemAxon software JChem for Excel 14.9.1500.912 (2014); ^b^, determined by PAMPA; n.d., could not be determined.

**Table 4:** *In silico* predicted Cytochrom P450 inhibition of compounds **31**–**36** and **49**–**66** expressed in percent.

Compound	CYP1A2	CYP2C9	CYP2C19	CYP2D6	CYP3A4
**31**	43	51	62	25	38
**32**	38	65	64	17	39
**33**	29	54	56	16	40
**34**	33	54	59	22	43
**35**	56	53	65	23	40
**36**	55	60	70	26	42
**49**	31	42	50	19	37
**50**	32	52	58	16	41
**51**	27	48	57	15	36
**52**	34	50	52	25	40
**53**	46	56	56	32	41
**54**	41	57	55	28	38
**55**	41	58	63	33	44
**56**	48	57	53	29	46
**57**	35	51	48	25	42
**58**	35	51	49	27	35
**59**	37	56	59	27	39
**60**	35	50	49	24	41
**61**	34	49	52	20	41
**62**	32	55	58	29	42
**63**	32	46	43	21	37
**64**	36	51	59	28	33
**65**	34	50	49	25	42
**66**	35	53	47	25	31

## Data Availability

Data is contained within the article and Supplementary Materials.

## Supplementary Materials

The following supporting information can be downloaded at: https://www.mdpi.com/article/10.3390/ph18071004/s1, Figures S1–S26: FTIR-, HRMS-, ^1^H- and ^13^C-NMR spectra for compounds **31**–**36**, **47** and **49**–**67**.

## Abbreviations

The following abbreviations are used in this manuscript:

AcOH	acetic acid
ACT	artemisinin-based combination therapy
ADME	absorption, distribution, metabolism, excretion
Boc	butyloxycarbonyl
br	broad
*c*_0_	initial donor well compound concentration
*c_A_*(*t*)	acceptor well compound concentration at time t
caMHA	cation adjusted Mueller–Hinton agar
caMHB	cation adjusted Mueller–Hinton broh
*c_D_*(*t*)	donor well compound concentration at time t
*c_equ_*	equilibrium concentration
CH	cyclohexane
CH_2_Cl_2_	dichloromethane
CO_2_	carbon dioxide
CQ	chloroquine
Cu	copper
CuI	copper iodide
CYP450	cytochrom P450 enzymes
d	doublet
DBU	1,8-diazabicyclo[5.4.0]undec-7-ene
dd	doublet of doublets
DIPEA	diisopropylethylamine
DMF	dimethylformamide
DMSO	dimethyl sulfoxide
DOX	doxorubicin
DPBS	Dulbecco’s phosphate-buffered saline
EDC × HCl	1-ethyl-3-(3-dimethylaminopropyl)carbodiimide hydrochloride
EtAc	ethyl acetate
EtOH	ethanol
EUCAST	European Committee on Antimicrobial Susceptibility Testing
H_2_	hydrogen
H_2_O	aqua demin
H_2_SO_4_	sulfuric acid
HA	heavy atoms
HCl	hydrogen chloride
HEPES	4-(2-hydroxyethyl)-1-piperazineethanesulfonic acid
HepG2-cells	human hepatocarcinoma cells
HRMS	mass spectrometry
IC_50_	half maximal inhibitory concentration
IR	infrared spectrometry
K_2_CO_3_	potassium carbonate
KI	potassium iodide
L6-cells	rat skeletal myofibroblasts
LE	ligand efficiency
m	multiplet
m.p.	melting point
MBC	minimal bactericidal concentration
*Me*OH	methanol
MHz	mega hertz
MIC	minimal inhibitory concentration
MMV	Medicines for Malaria Venture
NADPH	nicotinamide adenine dinucleotide phosphate
NaH	sodium hydride
NaHCO_3_	sodium hydrogen carbonate
NaNO_2_	sodium nitrite
NaOH	sodium hydroxide
NH_3_	ammonia
NH_4_Cl	ammonium chloride
NMR	nuclear magnetic resonance
O_2_	dioxygen
*P.f.*	*Plasmodium falciparum*
PAMPA	parallel artificial membrane permeability assay
PBS	phosphate-buffered saline
PdC	palladium on activated carbon
*Pe*	permeability
pH	potential of hydrogen
pIC_50_	negative logarithm of IC_50_
POD	podophyllotoxin
q	quartet
R	mass retention
RPMI 1640	cell culture medium for mammalian cells
rt	room temperature
s	singlet
*S*	filter area
S.I.	selectivity index
t	triplet
*t*	incubation time
td	triplet of doublets
THF	tetrahydrofuran
TLC	thin layer chromatography
TMS	tetramethyl silane
*V_A_*	acceptor well volume
*V_D_*	donor well volume
WHO	World Health Organization
