# Supplementary material for: Anti-Infective Properties, Cytotoxicity, and In Silico ADME Parameters of Novel 4′-(Piperazin-1-yl)benzanilides
Source: Pharmaceuticals (Basel). 2025 Jul 3;18(7):1004. doi: 10.3390/ph18071004 (PMC12298624; doi:10.3390/ph18071004)
Supplement: Supplementary file 1 [file pharmaceuticals-18-01004-s001.zip › pharmaceuticals-3674061-supplementary.pdf]

Supplementary Information

Anti-infective properties, cytotoxicity and *in silico* ADME parameters of novel 4´-(Piperazin-1-yl)benzanilides

Theresa Hermann [1]*, Sarah Harzl [1], Robin Wallner [1], Elke Prettner [†], Eva-Maria Pferschy-Wenzig [2], Monica Cal [3,4], Pascal Mäser [3,4] and Robert Weis [1]

[1] Pharmaceutical Chemistry, Institute of Pharmaceutical Sciences, University of Graz, Schubertstraße 1, 8010 Graz, Austria; theresa.hermann@uni-graz.at, (T.H.), harzl.sarah@gmail.com (S.H), wallner.rob@gmail.com (R.W.), robert.weis@uni-graz.at (R.W.)

[2] Pharmacognosy, Institute of Pharmaceutical Sciences, University of Graz, Beethovenstraße 8, 8010 Graz, Austria; eva-maria.wenzig@uni-graz.at (E.-M.P.-W.)

[3] Swiss Tropical and Public Health Institute, Kreuzstraße 2, Allschwil, CH-4123 Basel, Switzerland, monica.cal@swisstph.ch (M.C.); pascal.maeser@swisstph.ch (P.M.)

[4] Faculty of Science, University of Basel, Petersplatz 1, , CH-4003 Basel, Switzerland

* Correspondence: theresa.hermann@uni-graz.at; Tel.: +43-316-380-5381; Fax: +43-316-380-9846

FTIR, HRMS, ^{1}H and ^{13}C NMR spectra of compounds 31-36, 47, 49-67

Figure S1. FTIR and HRMS spectra, 1H NMR at 400 MHz and 13C NMR at 100 MHz spectra for compound **31** ($CDCl_3$)

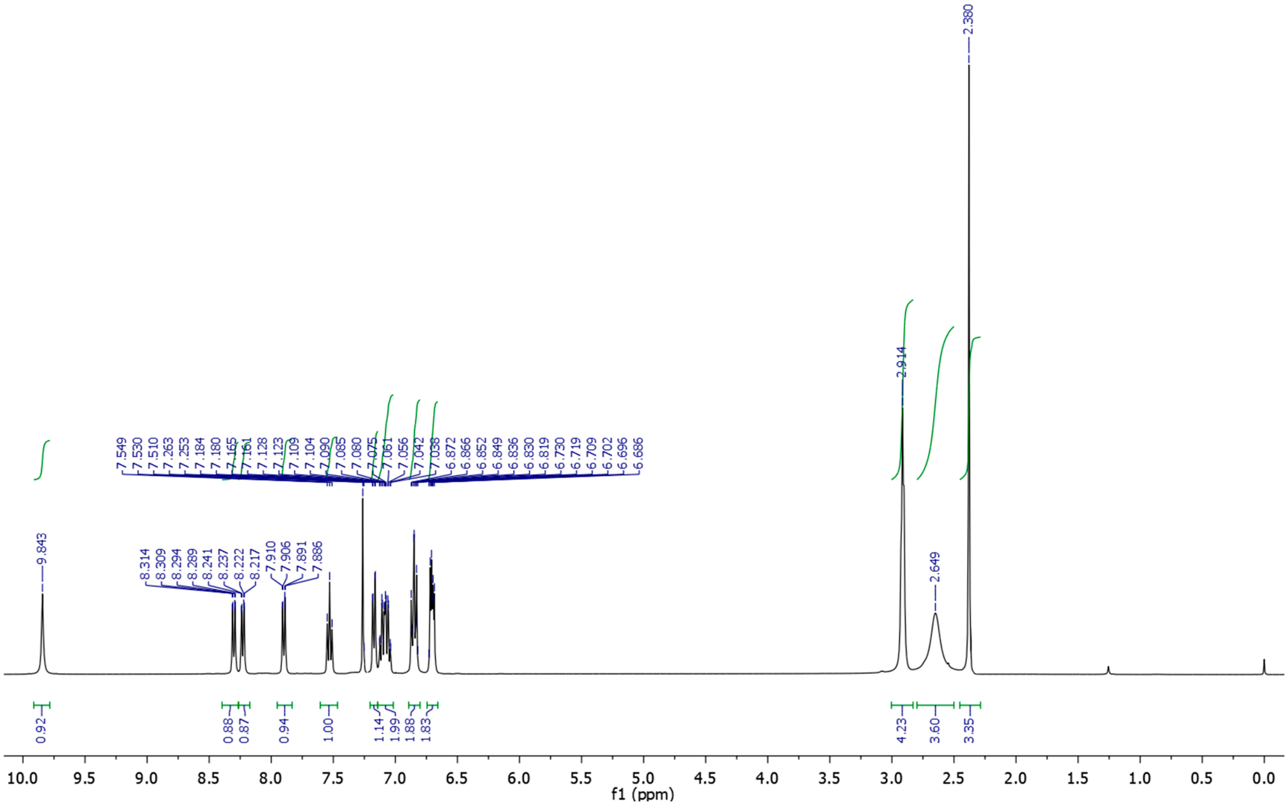

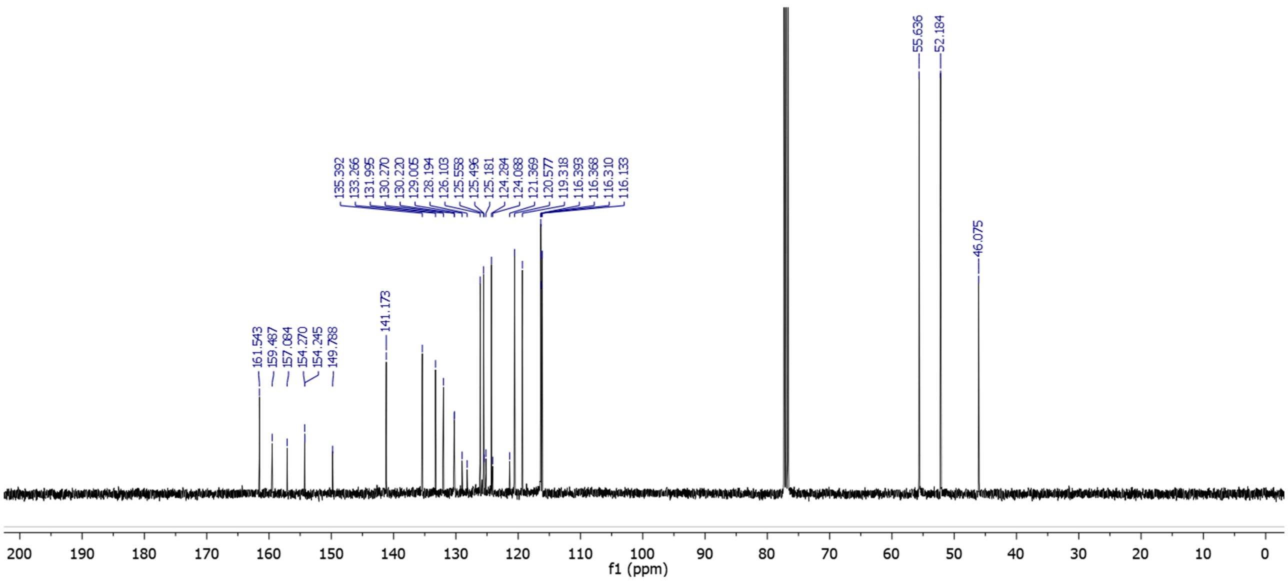

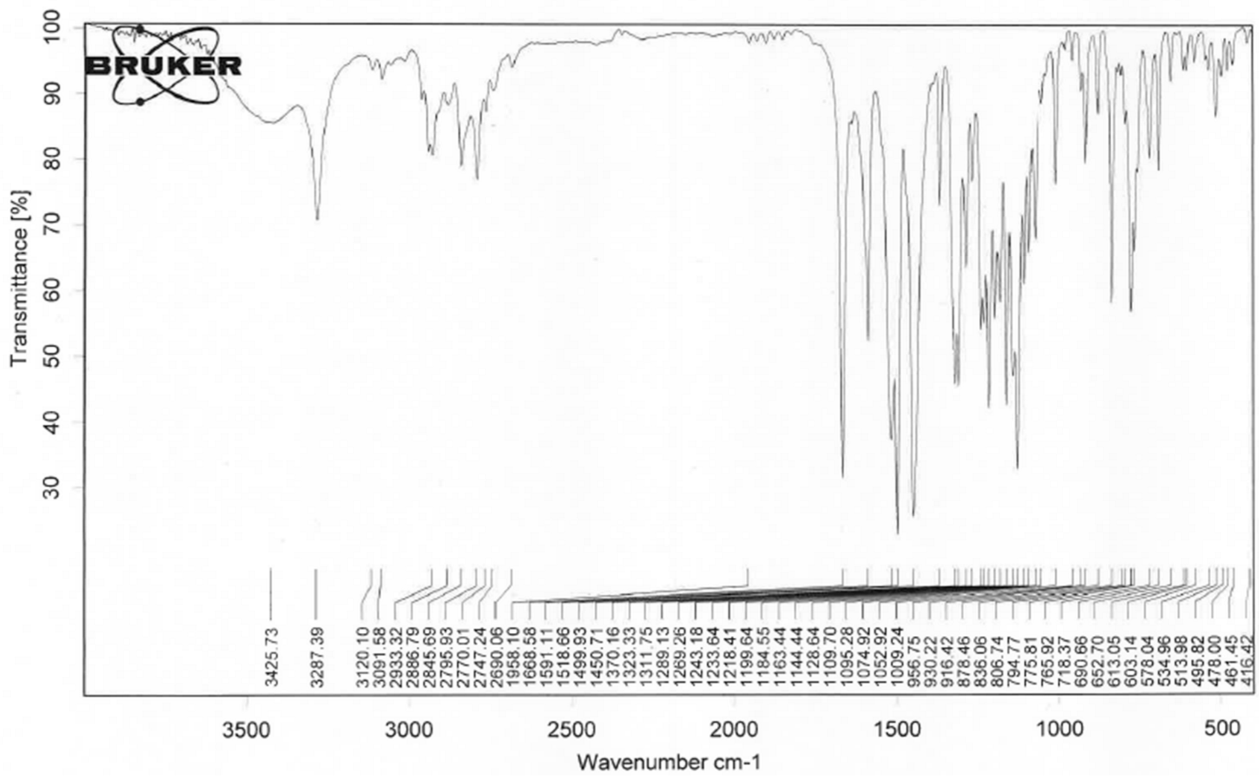
BRUKER
Transmittance [%]
100
90
80
70
60
50
40
30
3425.73
3287.39
3120.10
3091.58
2933.32
2886.79
2845.69
2795.93
2770.01
2747.24
2690.06
1958.10
1668.58
1591.11
1518.66
1499.93
1450.71
1370.16
1323.33
1311.75
1289.13
1269.26
1243.18
1233.64
1218.41
1199.64
1184.55
1163.44
1144.44
1128.64
1109.70
1095.28
1074.92
1052.92
1009.24
956.75
930.22
916.42
878.46
836.06
806.74
794.77
775.81
765.92
718.37
690.66
652.70
613.05
603.14
578.04
534.96
513.98
495.82
478.00
461.45
416.42
3500
3000
2500
2000
1500
1000
500
Wavenumber cm-1

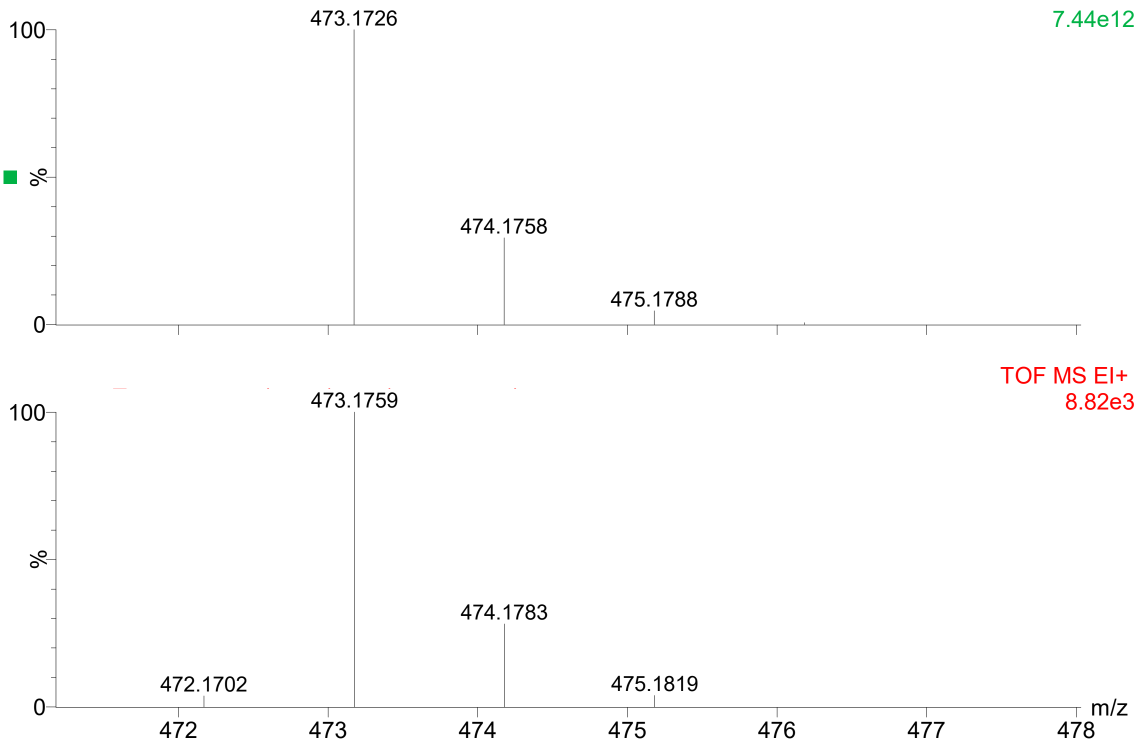
473.1726
7.44e12
100
%
474.1758
475.1788
0
TOF MS EI+
8.82e3
473.1759
100
%
474.1783
472.1702
475.1819
0
472
473
474
475
476
477
478
m/z

Figure S2. FTIR and HRMS spectra, 1H NMR at 400 MHz and 13C NMR at 100 MHz spectra for compound **32** ($CDCl_3$)

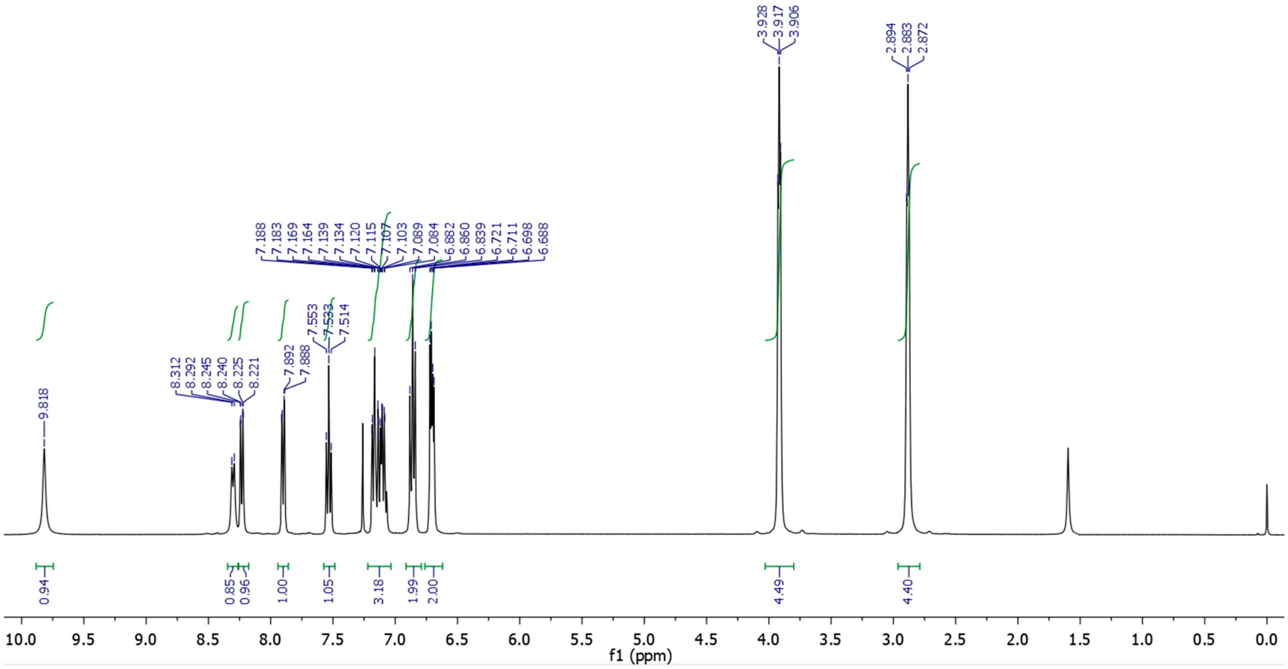

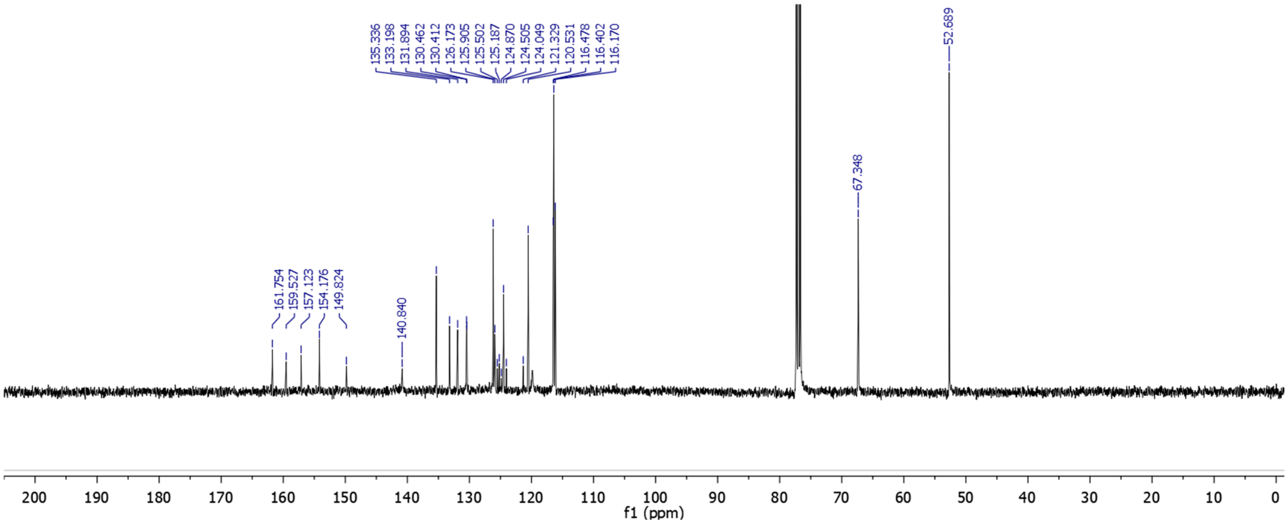

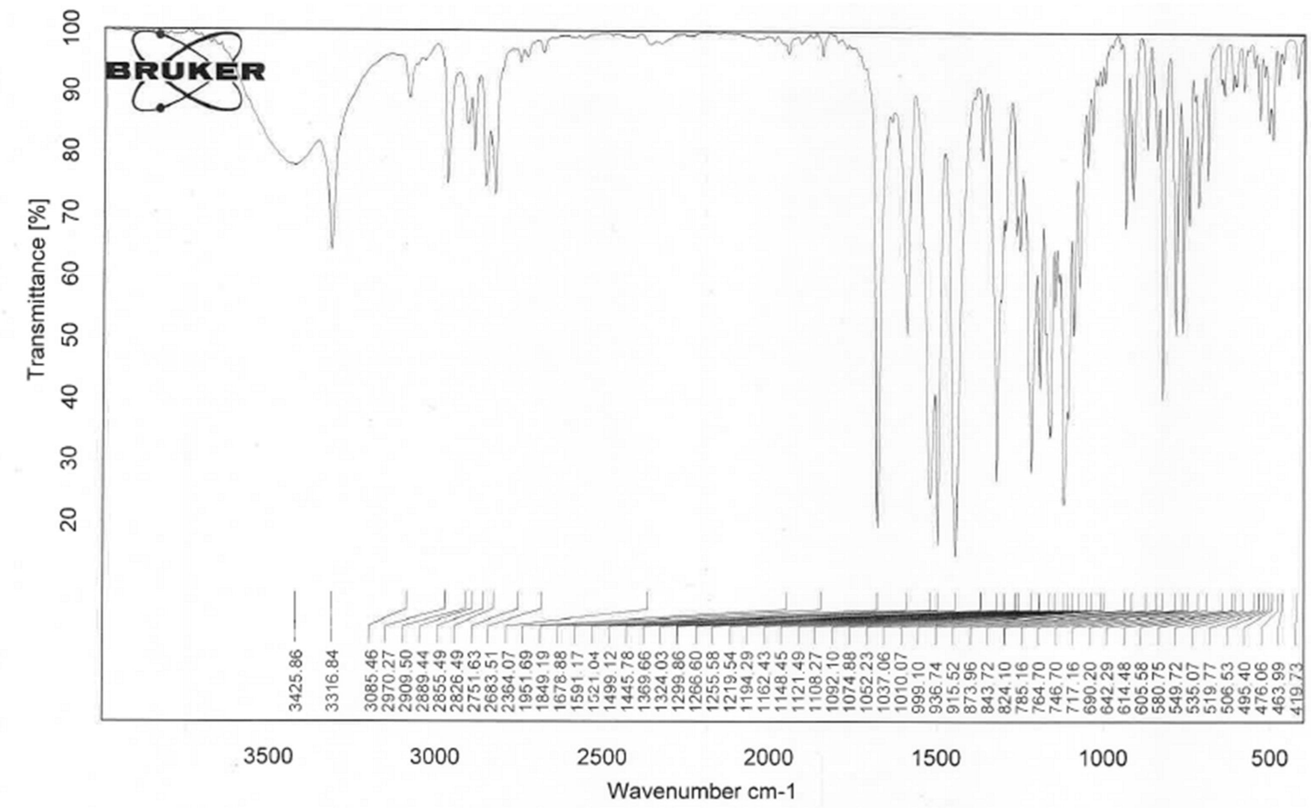

BRUKER
Transmittance [%]
Wavenumber cm-1
3425.86
3316.84
3085.46
2970.27
2909.50
2889.44
2855.49
2826.49
2751.63
2683.51
2364.07
1951.69
1849.19
1678.88
1591.17
1521.04
1499.12
1445.78
1369.66
1324.03
1299.86
1266.60
1255.58
1219.54
1194.29
1162.43
1148.45
1121.49
1108.27
1092.10
1074.88
1052.23
1037.06
1010.07
999.10
936.74
915.52
873.96
843.72
824.10
785.16
764.70
746.70
717.16
690.20
642.29
614.48
605.58
580.75
549.72
535.07
519.77
506.53
495.40
476.06
463.99
419.73

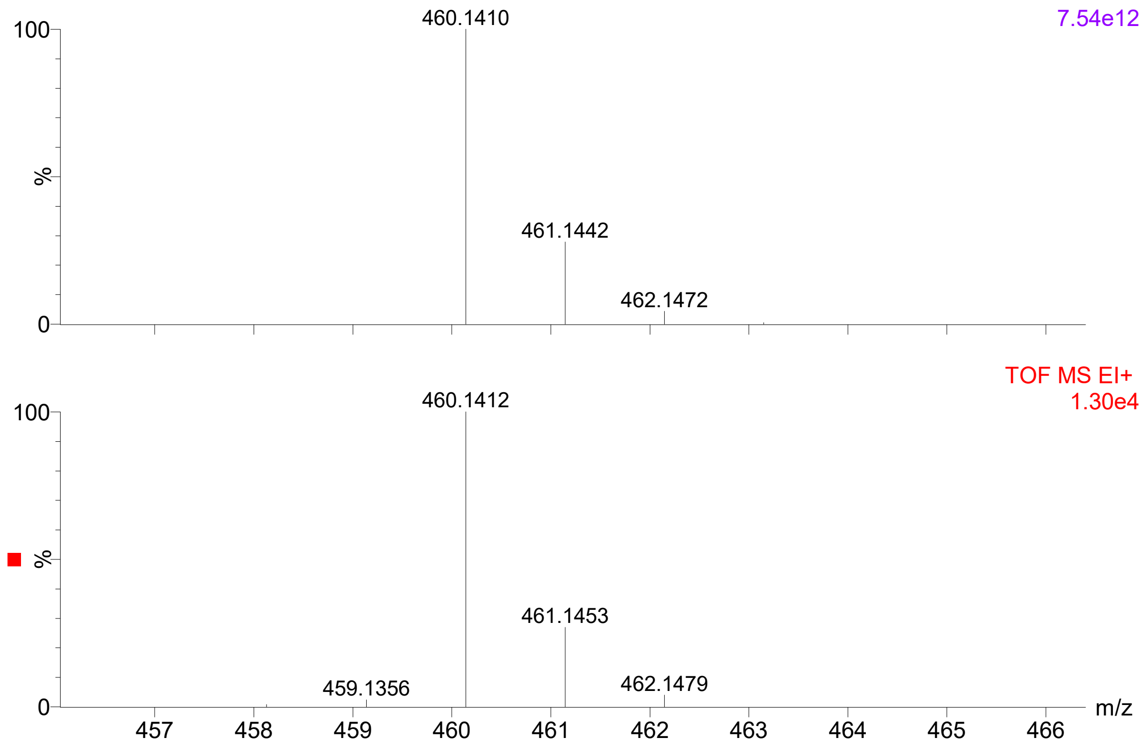

7.54e12
460.1410
461.1442
462.1472
TOF MS EI+
1.30e4
460.1412
461.1453
462.1479
459.1356
%
m/z

Figure S3. FTIR and HRMS spectra, 1H NMR at 400 MHz and 13C NMR at 100 MHz spectra for compound **33** ($CDCl_3$)

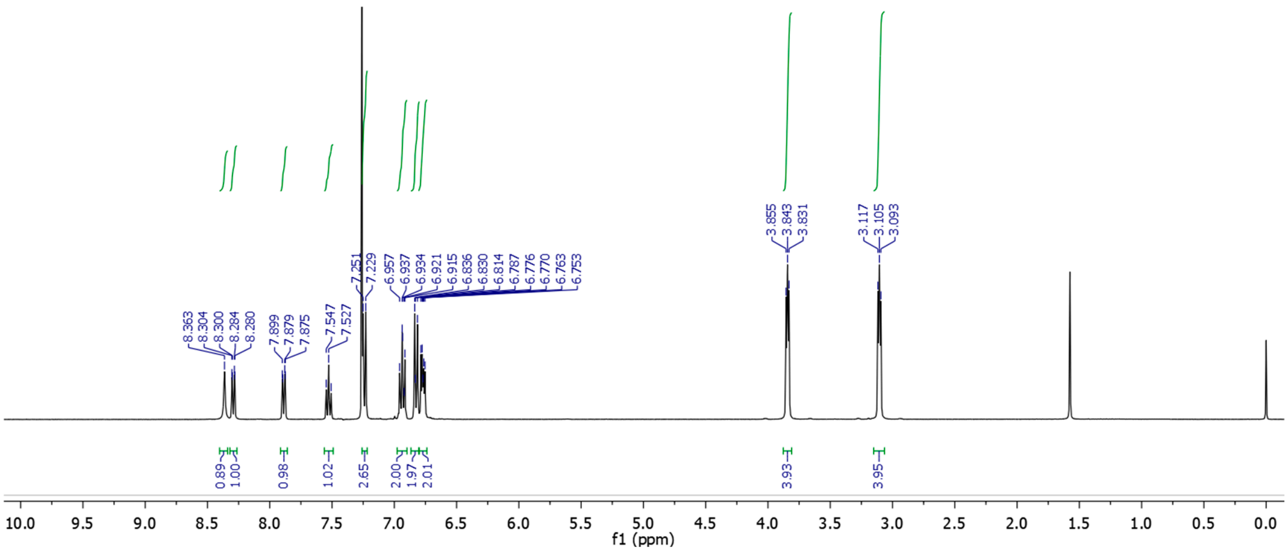

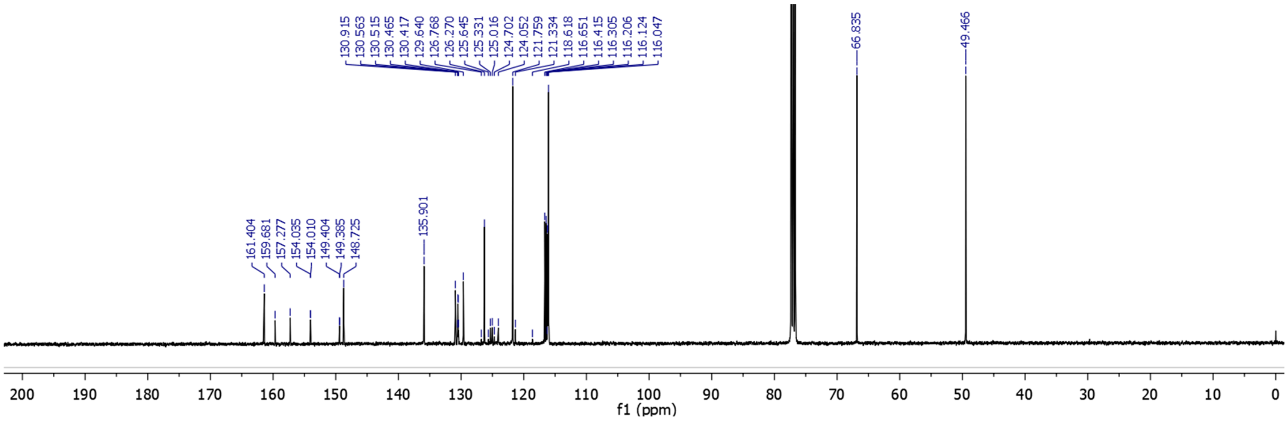

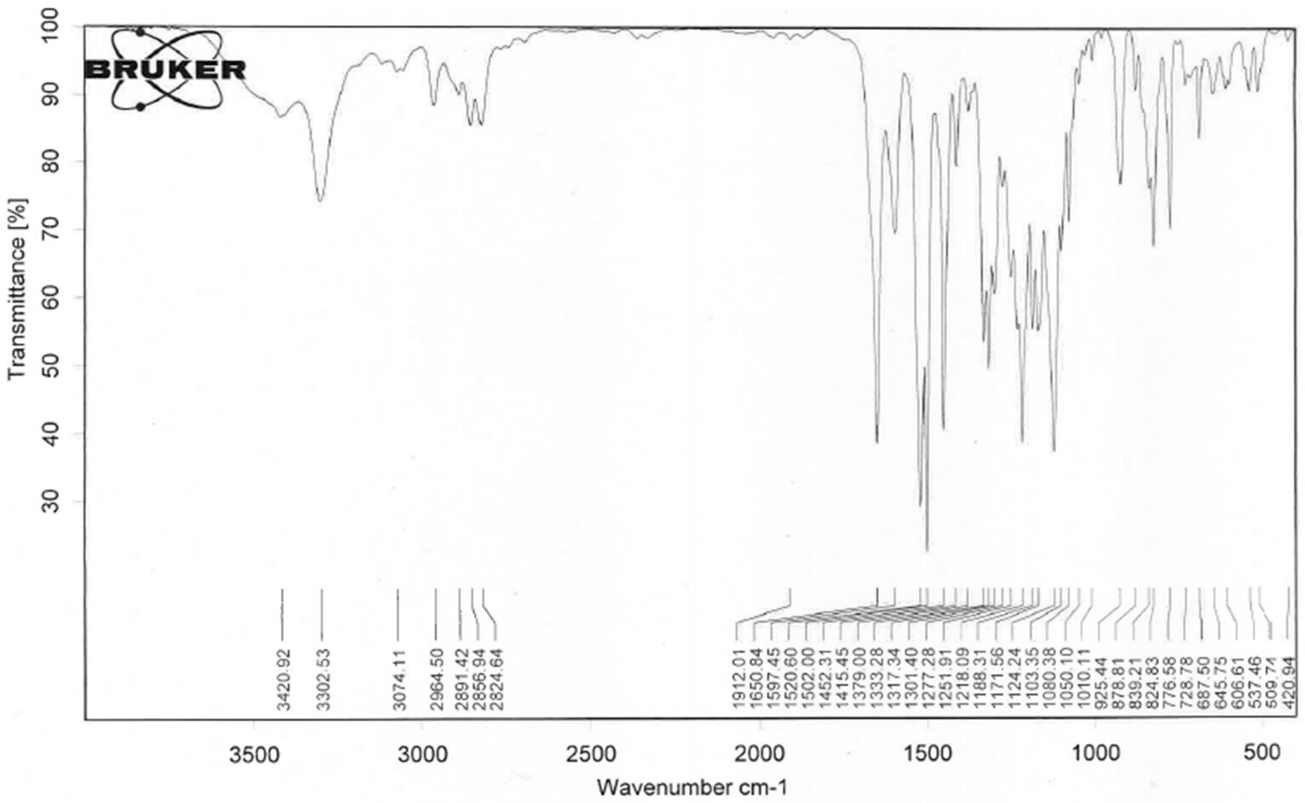
BRUKER
Transmittance [%]
Wavenumber cm-1
3420.92
3302.53
3074.11
2964.50
2891.42
2856.94
2824.64
1912.01
1650.84
1597.45
1520.60
1502.00
1452.31
1415.45
1379.00
1333.28
1317.34
1301.40
1277.28
1251.91
1218.09
1188.31
1171.56
1124.24
1103.35
1080.38
1050.10
1010.11
925.44
878.81
839.21
824.83
776.58
728.78
687.50
645.75
606.61
537.46
509.74
420.94

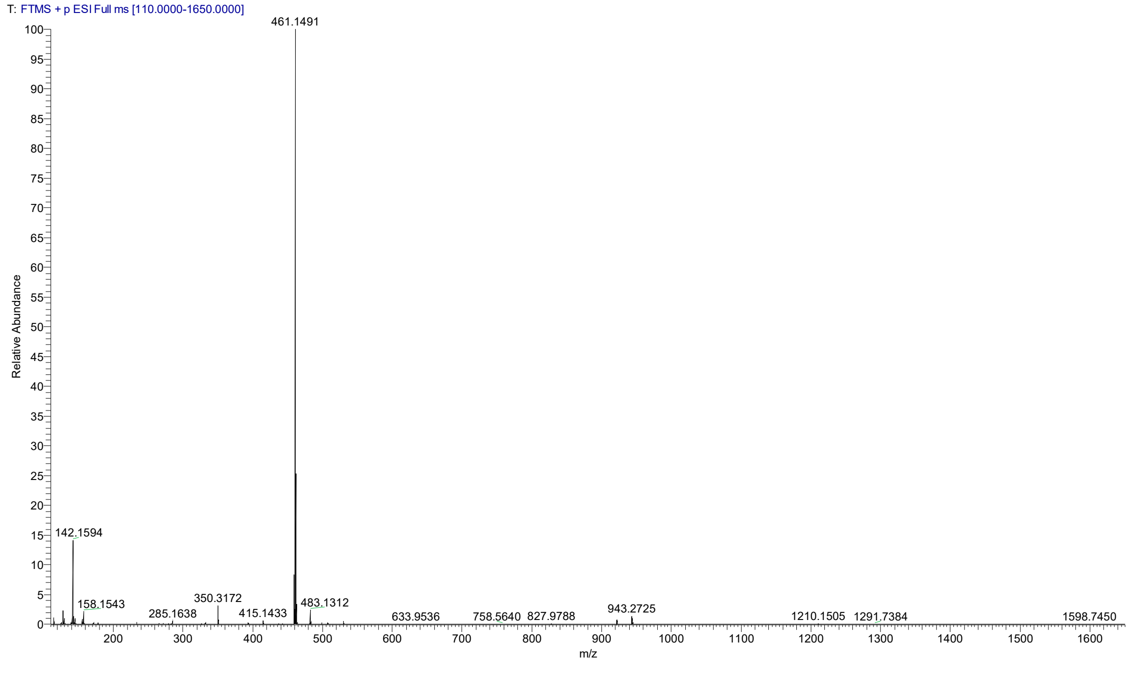
T: FTMS + p ESI Full ms [110.0000-1650.0000]
Relative Abundance
m/z
461.1491
142.1594
158.1543
285.1638
350.3172
415.1433
483.1312
633.9536
758.5640
827.9788
943.2725
1210.1505
1291.7384
1598.7450

Figure S4. FTIR and HRMS spectra, 1H NMR at 400 MHz and 13C NMR at 100 MHz spectra for compound **34** ($CDCl_3$)

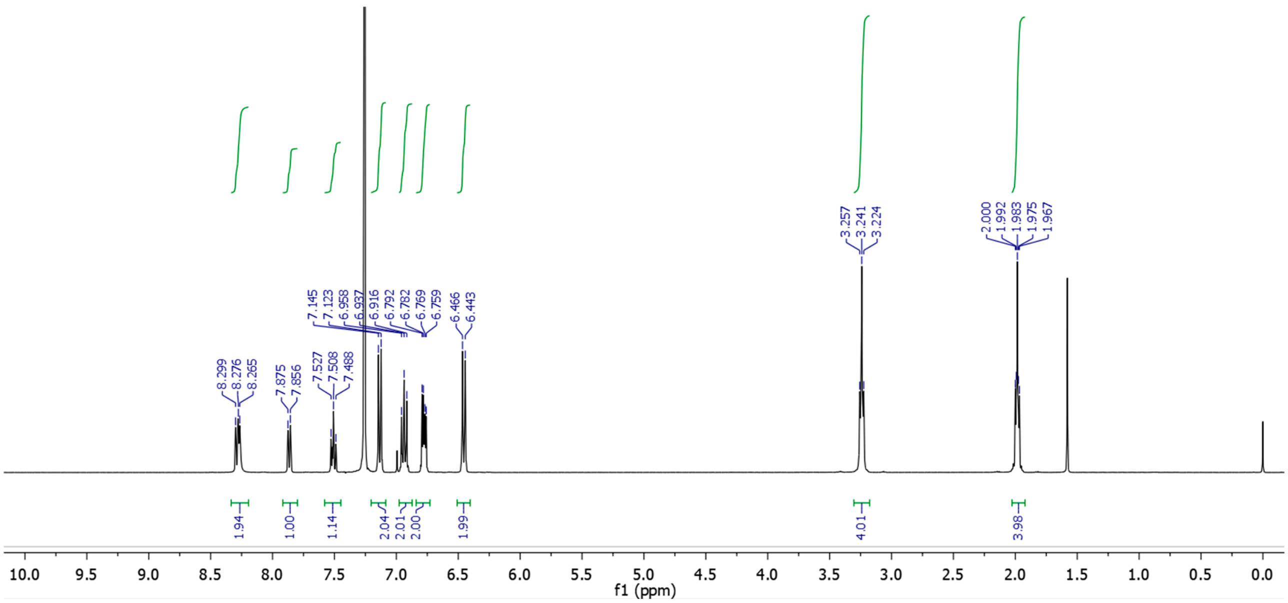

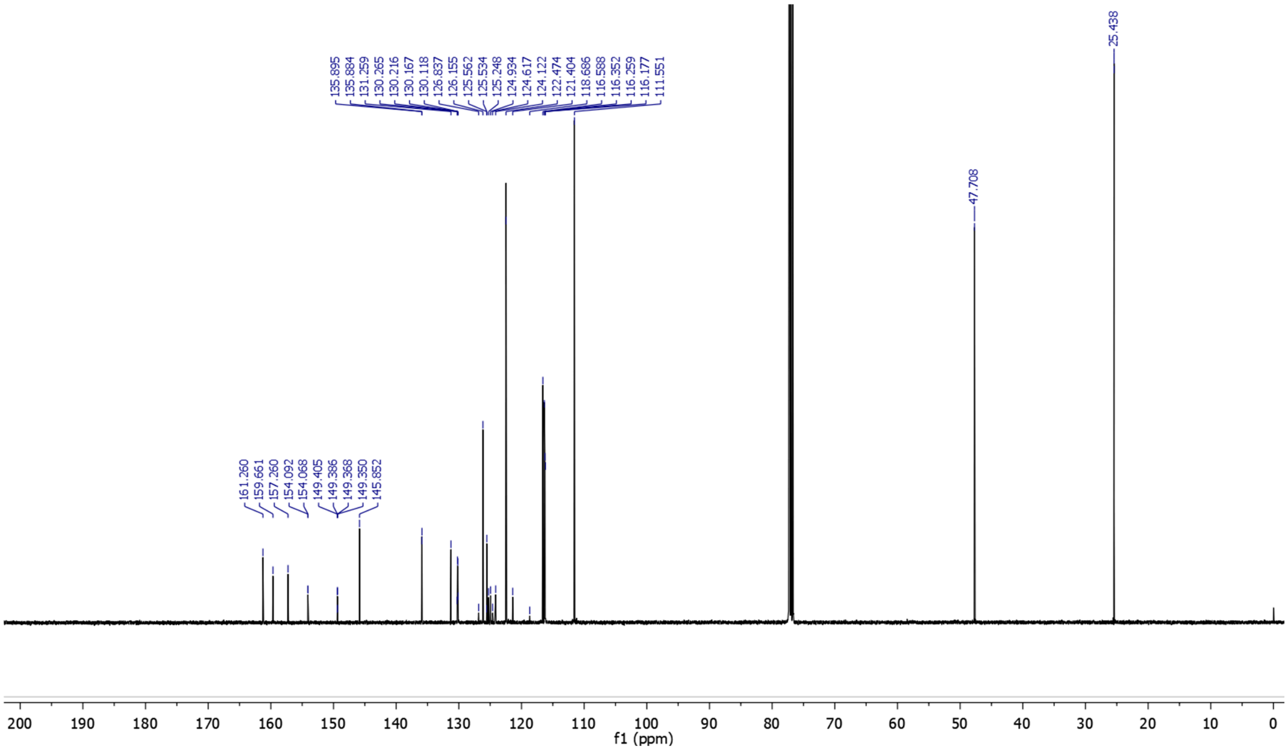

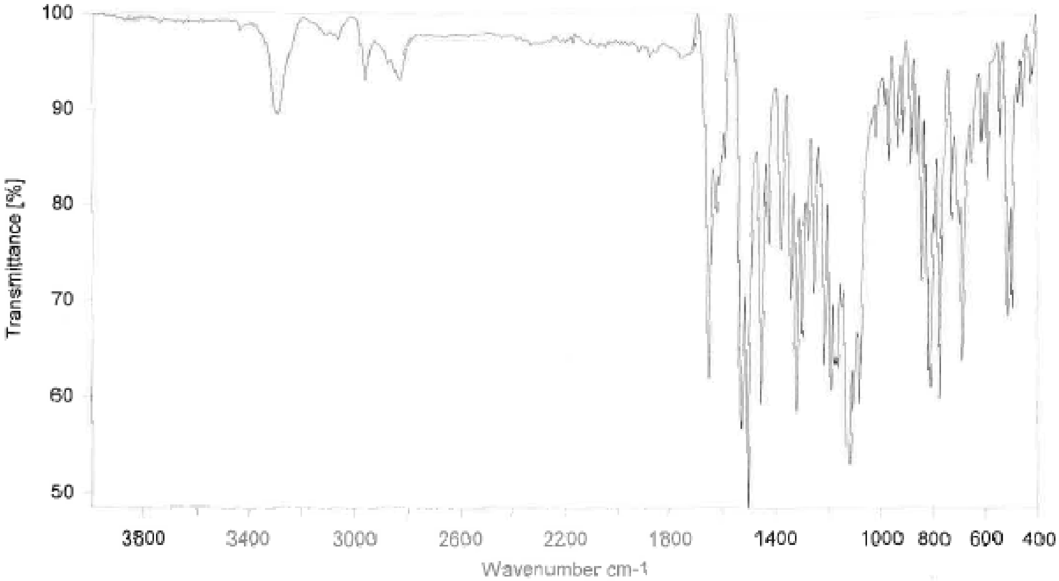

Wavelength	Absolute Intensity	Relative Intensity	Width	if Line < Shoulder	
3282.9515	0.894	0.106	65.5952	20.496403	0
3055.3915	0.973	0.024	82.9650	4.618019	0
2954.5821	0.931	0.044	21.9950	8.018835	0
2827.0901	0.930	0.067	88.9877	12.916131	0
2322.7373	0.967	0.013	121.1734	2.614077	0
1868.4075	0.954	0.016	37.1934	2.999278	0
1744.7135	0.953	0.046	2073.8810	5.288213	0
1645.4077	0.617	0.383	19.1932	74.289825	0
1616.0752	0.791	0.080	63.1891	9.032324	0
1603.1204	0.819	0.030	114.0981	2.010479	0
1582.7494	0.848	0.054	80.3201	5.170928	0
1522.4729	0.566	0.216	66.4724	27.299412	0
1499.7510	0.484	0.516	46.6851	100.024681	0
1448.4888	0.591	0.280	15.6322	51.313316	0
1421.0394	0.757	0.104	8.2528	16.907667	0
1372.6698	0.752	0.166	18.8685	31.547213	0
1336.5447	0.697	0.130	8.6668	22.351416	0
1316.3063	0.583	0.309	14.8066	55.840973	0
1294.2945	0.661	0.145	12.7433	24.315962	0
1272.1818	0.763	0.067	135.4290	7.956750	0
1248.2921	0.707	0.160	15.3734	30.003365	0
1212.9439	0.630	0.190	15.9082	33.398857	0
1184.3017	0.607	0.156	230.3227	21.594978	0
1160.4415	0.629	0.082	17.3824	13.358731	0
1146.0333	0.694	0.021	10.6278	3.251748	0
1116.0430	0.530	0.414	126.3108	76.247696	0
1101.6388	0.586	0.046	6.5444	8.298297	0
1075.5658	0.592	0.082	7.3777	14.677866	0
1011.1255	0.870	0.041	7.2168	6.632605	0
977.6670	0.903	0.024	5.8295	3.913334	0
964.5718	0.845	0.098	9.0941	17.263029	0
929.2992	0.859	0.103	14.3465	18.763721	0
909.5557	0.874	0.081	7.1270	14.815574	0
877.5612	0.839	0.122	8.9284	22.183083	0
855.8779	0.853	0.079	9.3247	12.157379	0
837.4151	0.721	0.181	8.8000	32.599022	0
807.9437	0.610	0.268	22.0332	46.571229	0
790.4782	0.770	0.041	117.7714	4.098952	0
771.7197	0.597	0.382	13.7739	72.844910	0
726.5155	0.785	0.121	43.5523	17.032948	0
699.9796	0.780	0.038	61.0903	4.070791	0
686.3652	0.636	0.315	19.2417	59.498516	0

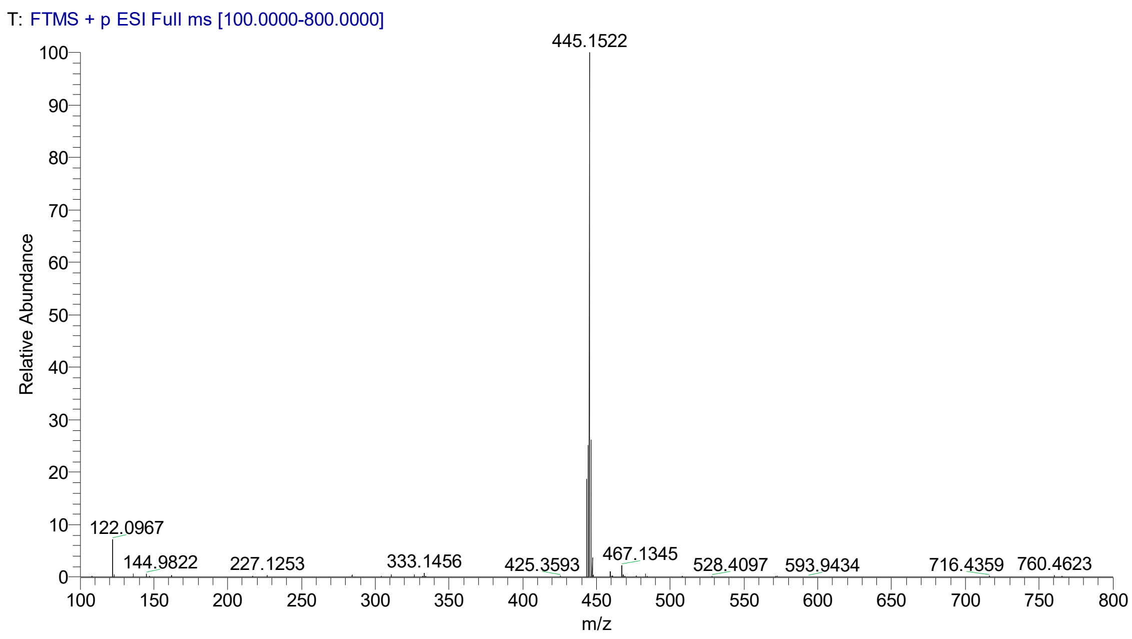
T: FTMS + p ESI Full ms [100.0000-800.0000]
445.1522
122.0967
144.9822
227.1253
333.1456
425.3593
467.1345
528.4097
593.9434
716.4359
760.4623
Relative Abundance
m/z

Figure S5. FTIR and HRMS spectra, 1H NMR at 400 MHz and 13C NMR at 100 MHz spectra for compound **35** ($CDCl_3$)

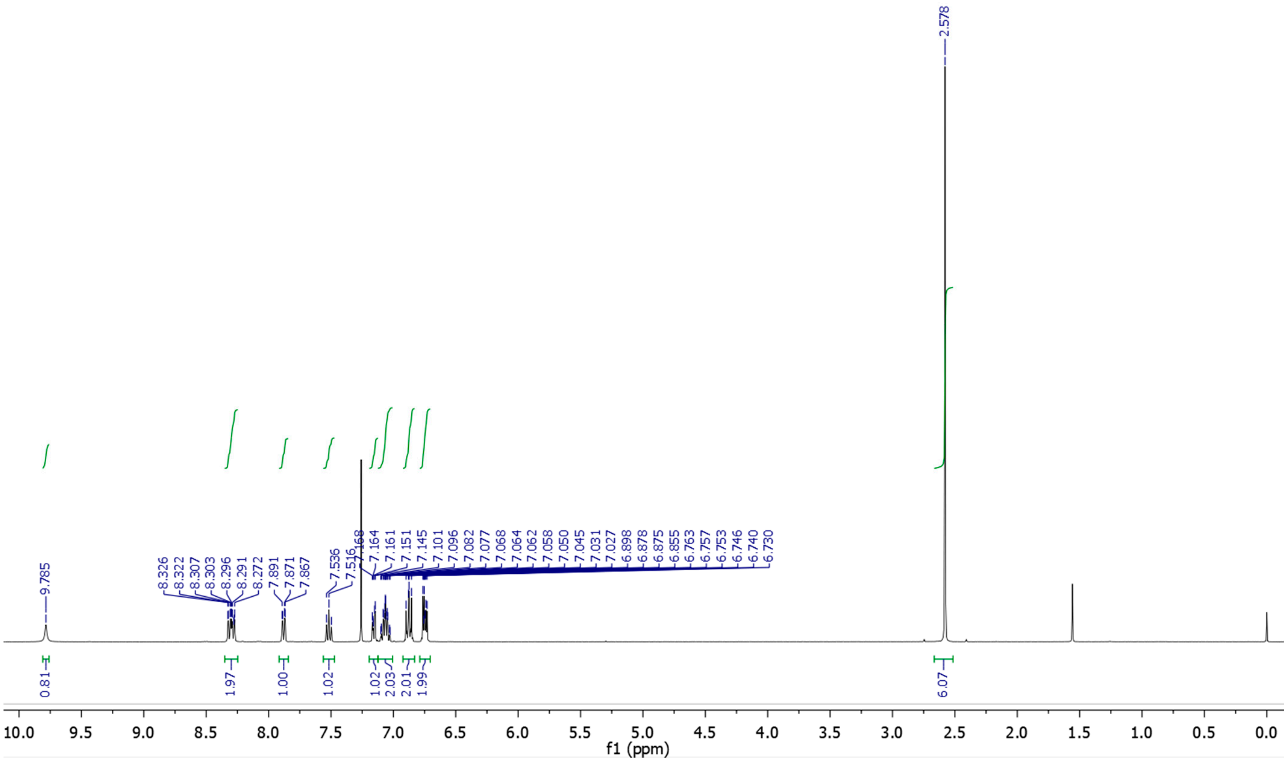

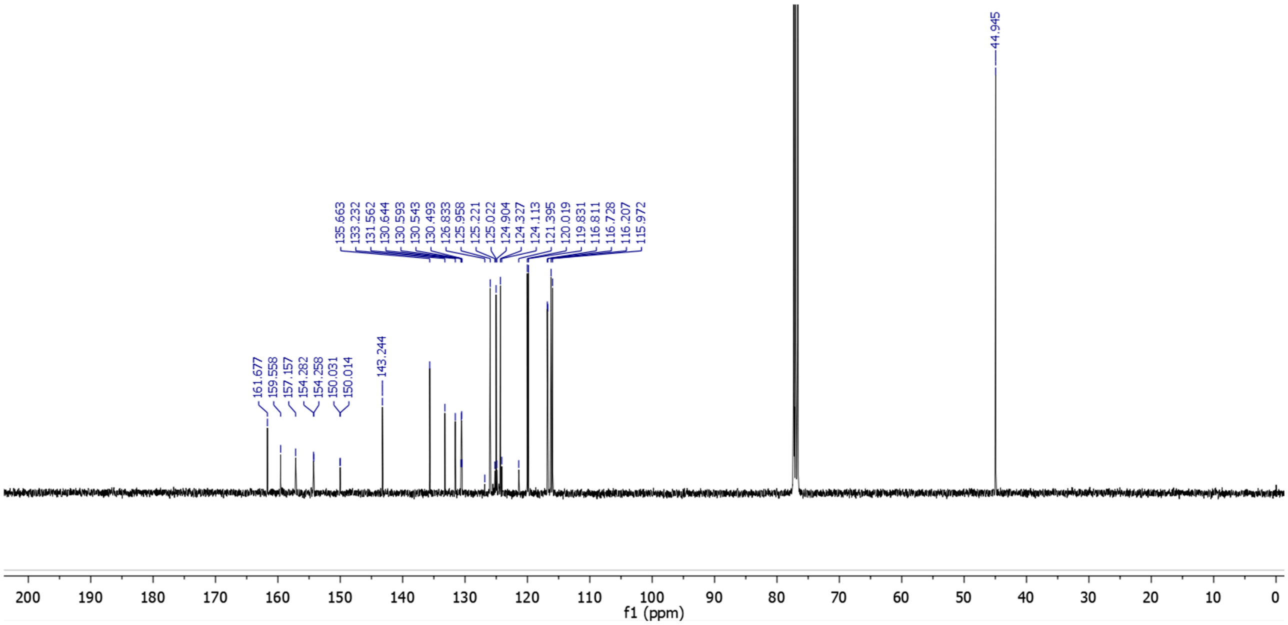

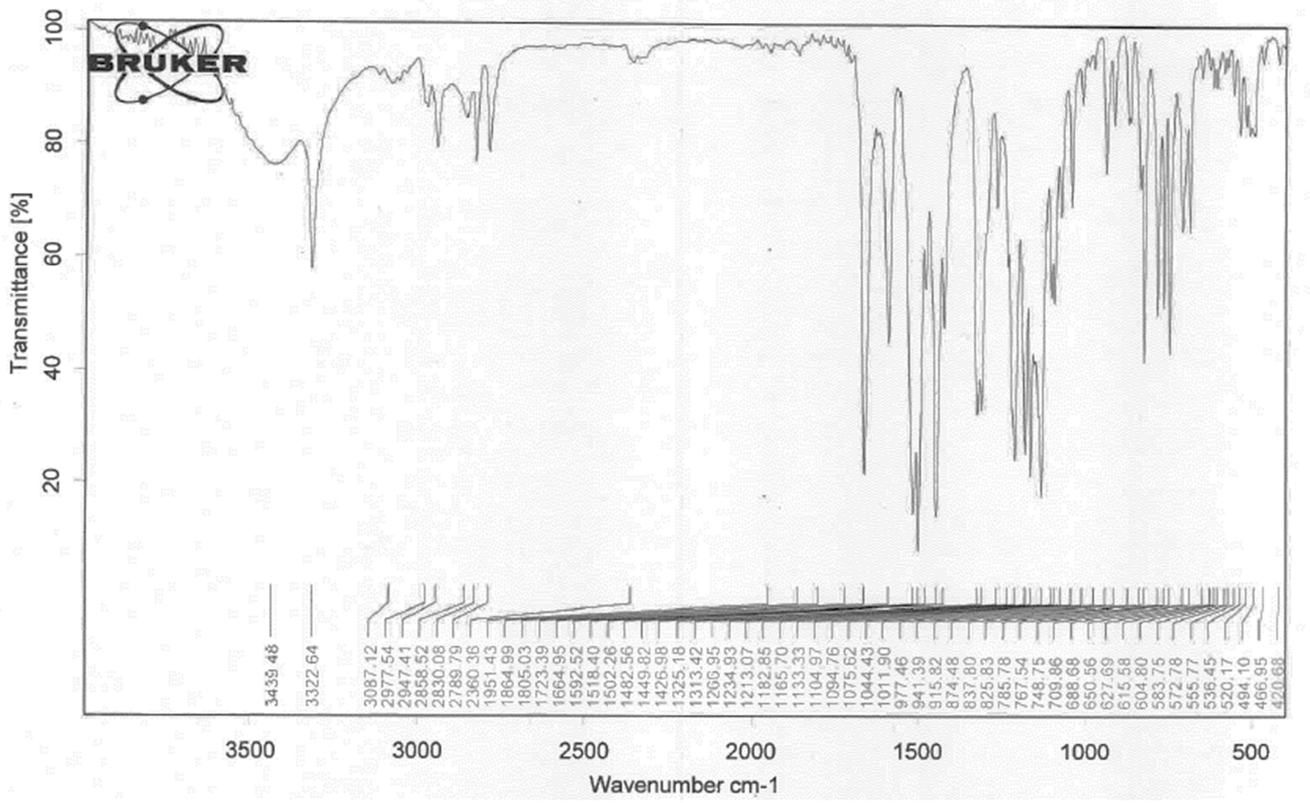

BRUKER
Transmittance [%]
Wavenumber cm-1

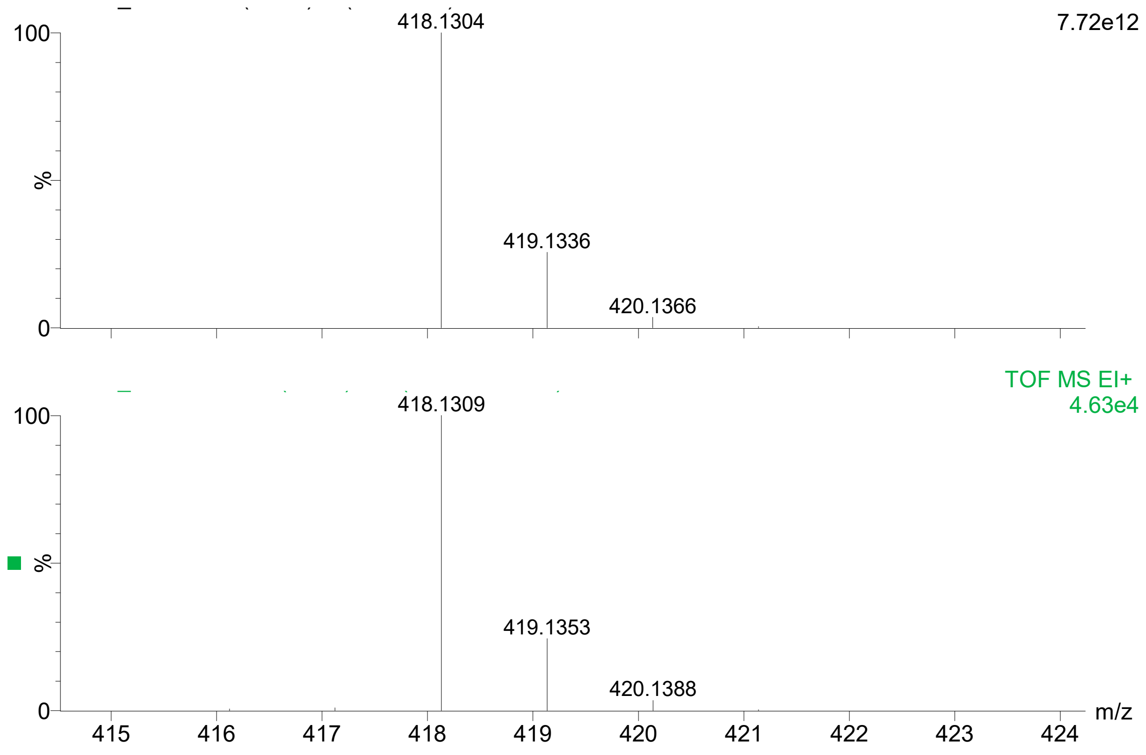

418.1304
7.72e12
419.1336
420.1366
%
TOF MS EI+
4.63e4
418.1309
419.1353
420.1388
m/z

Figure S6. FTIR and HRMS spectra, 1H NMR at 400 MHz and 13C NMR at 100 MHz spectra for compound **36** ($CDCl_3$)

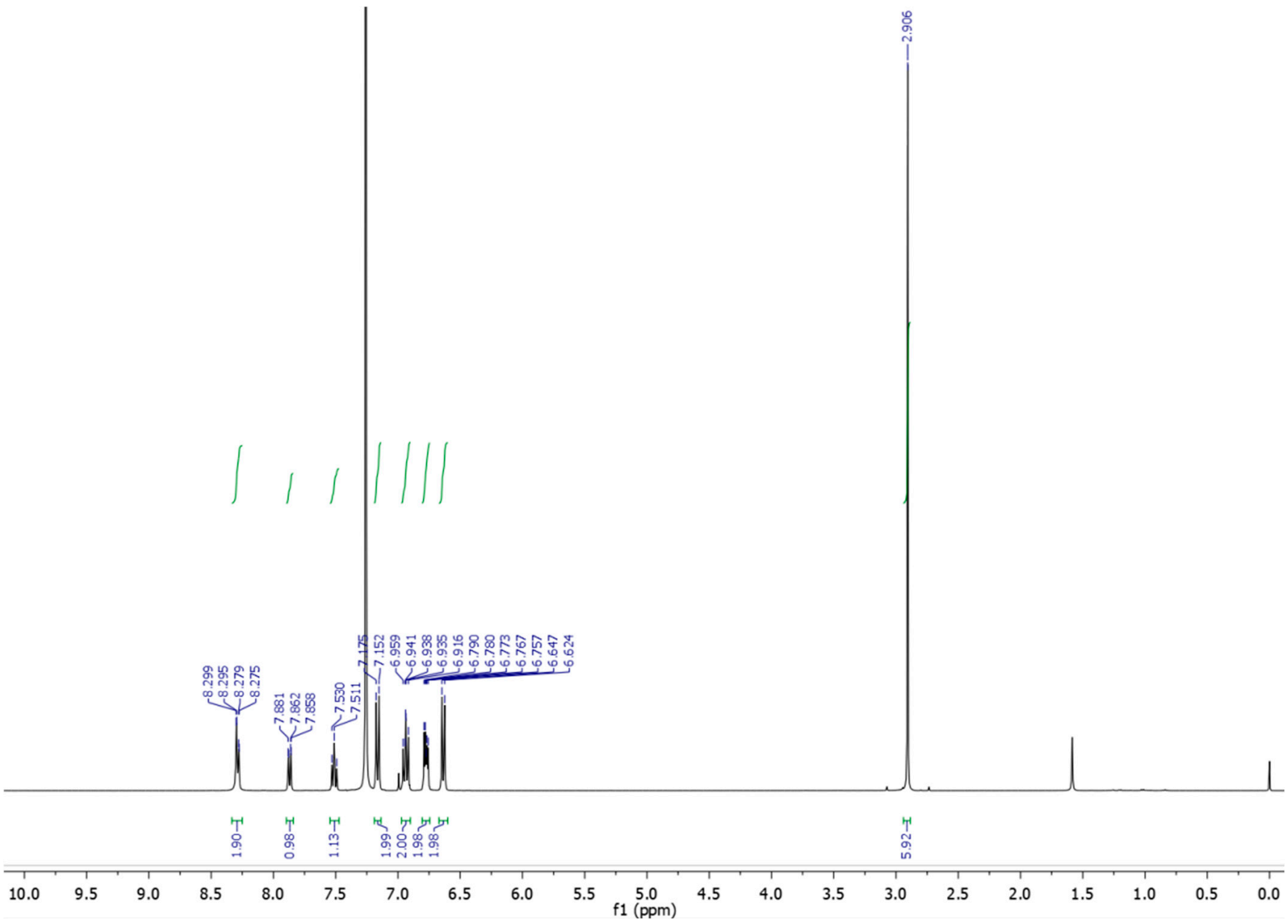

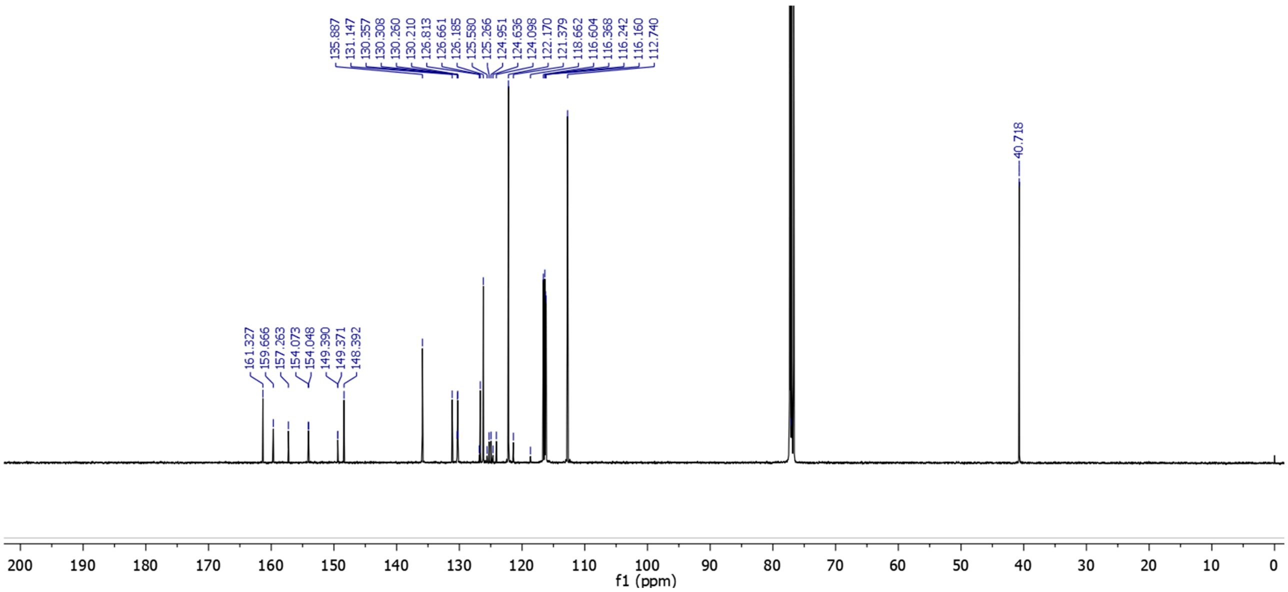

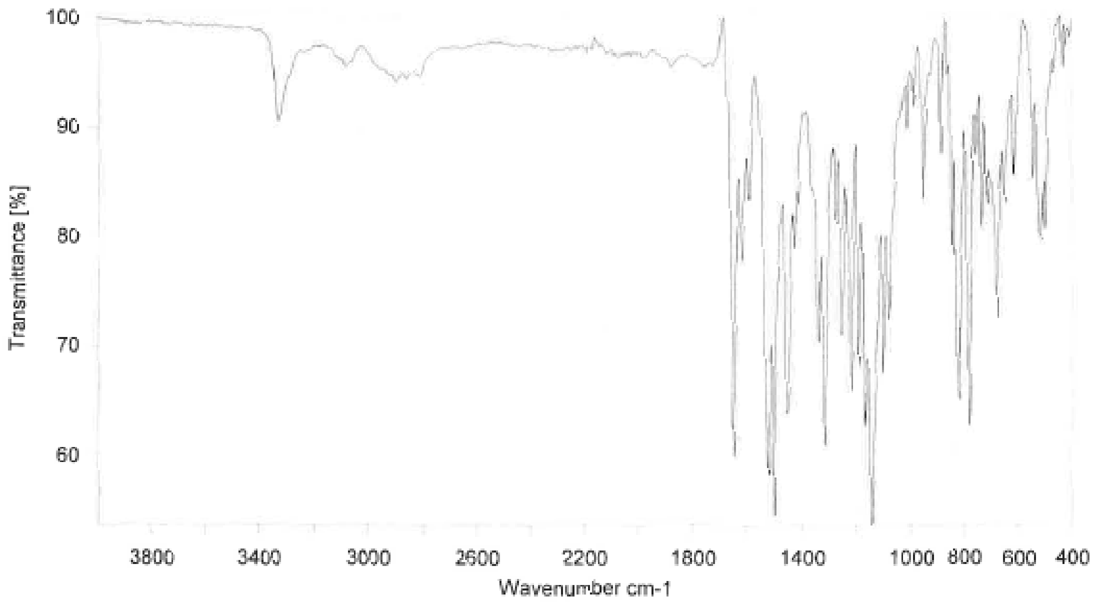

Wavelength	Absolute Intensity	Relative Intensity	Width	if Line < Shoulder	
3323.2387	0.906	0.094	62.9833	29.143198	0
3078.0070	0.956	0.019	70.9859	3.998926	0
2894.1264	0.943	0.038	198.9616	7.331126	0
2191.2637	0.969	0.013	339.7721	2.126271	0
1876.4050	0.956	0.037	1040.3827	5.686818	0
1749.5007	0.957	0.027	206.6704	2.311072	0
1645.5627	0.599	0.350	17.4118	74.598869	0
1616.2497	0.777	0.092	11.7989	16.256824	0
1588.0505	0.834	0.061	68.0626	8.430769	0
1518.1099	0.582	0.121	13.8099	25.720284	0
1499.3906	0.541	0.377	44.6242	79.919533	0
1451.1702	0.637	0.299	20.6055	41.460327	0
1422.7715	0.788	0.044	107.0398	6.887565	0
1410.8229	0.836	0.022	85.6824	2.742943	0
1335.1051	0.703	0.103	12.1268	17.249237	0
1316.2449	0.609	0.295	21.3212	59.091457	0
1270.4167	0.811	0.065	8.9917	12.802934	0
1247.1172	0.719	0.145	14.7784	28.933779	0
1215.3936	0.658	0.226	16.4139	48.033031	0
1186.1484	0.680	0.165	10.9494	28.685741	0
1162.8792	0.628	0.095	7514.5458	13.147407	0
1140.6051	0.537	0.467	59.1713	99.217125	0
1096.4917	0.675	0.134	9.6343	27.046856	0
1076.2035	0.724	0.085	9.9138	16.439852	0
1007.4306	0.898	0.046	6.8207	8.754515	0
981.0700	0.918	0.041	8.0023	7.339881	0
945.8288	0.833	0.146	14.6942	30.123526	0
878.8722	0.875	0.118	8.8844	22.849716	0
856.5395	0.949	0.024	1053.8411	2.252773	0
838.4546	0.783	0.114	8.5841	18.946892	0
814.6279	0.651	0.276	19.4568	53.015396	0
776.9079	0.626	0.375	18.9094	80.143806	0
751.1729	0.875	0.046	7.6844	8.208587	0
733.2945	0.809	0.111	7.6720	20.964605	0
713.3846	0.831	0.053	544.2907	6.649864	0
675.9416	0.726	0.220	31.2903	43.705891	0
648.8084	0.832	0.057	7.2463	10.162563	0
[illegible]	[illegible]				

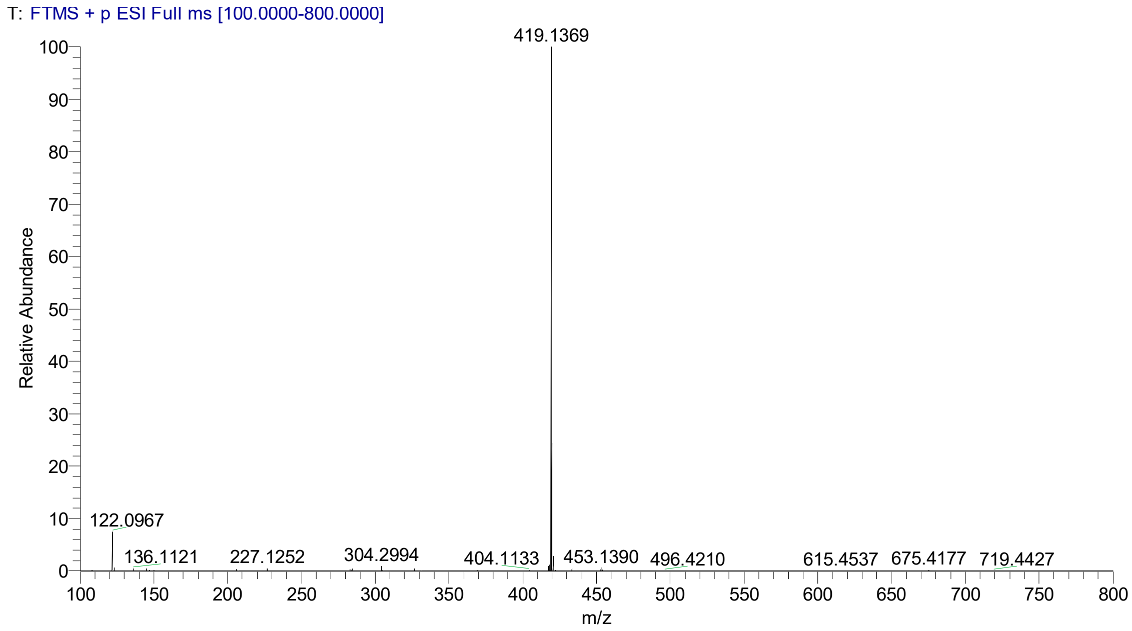
T: FTMS + p ESI Full ms [100.0000-800.0000]
419.1369
122.0967
136.1121
227.1252
304.2994
404.1133
453.1390
496.4210
615.4537
675.4177
719.4427
Relative Abundance
m/z

Figure S7. FTIR and HRMS spectra, 1H NMR at 400 MHz and 13C NMR at 100 MHz spectra for compound **47** ($CDCl_3$)

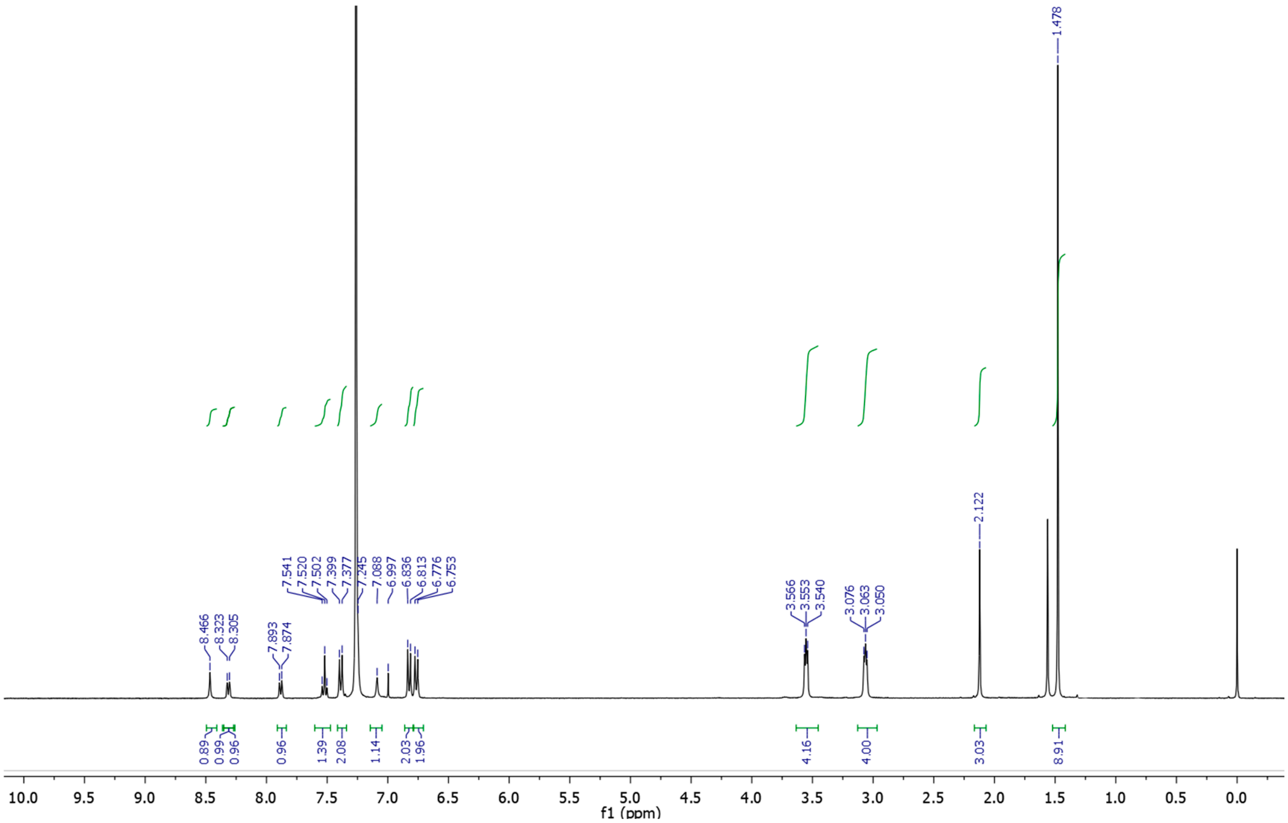

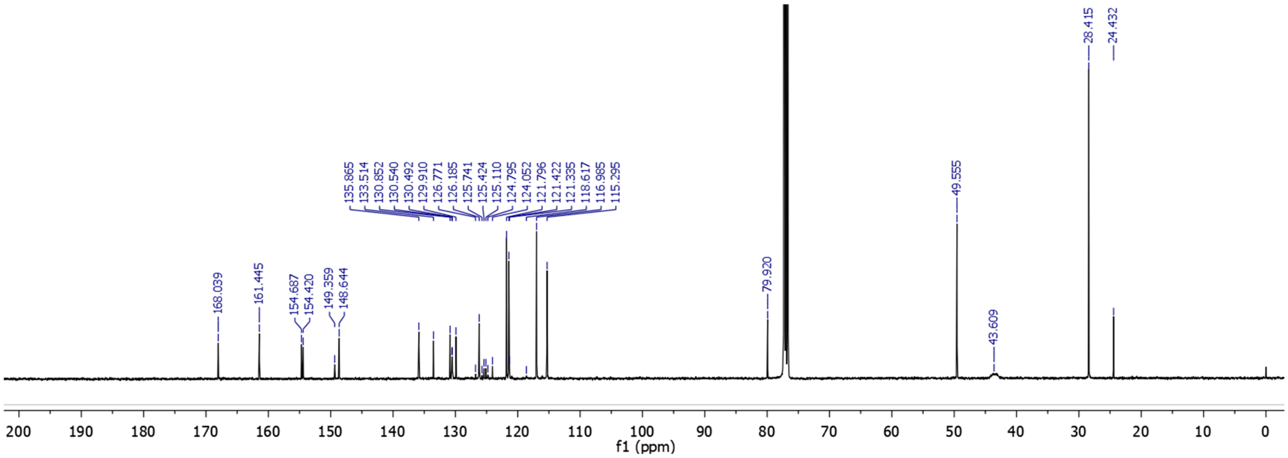

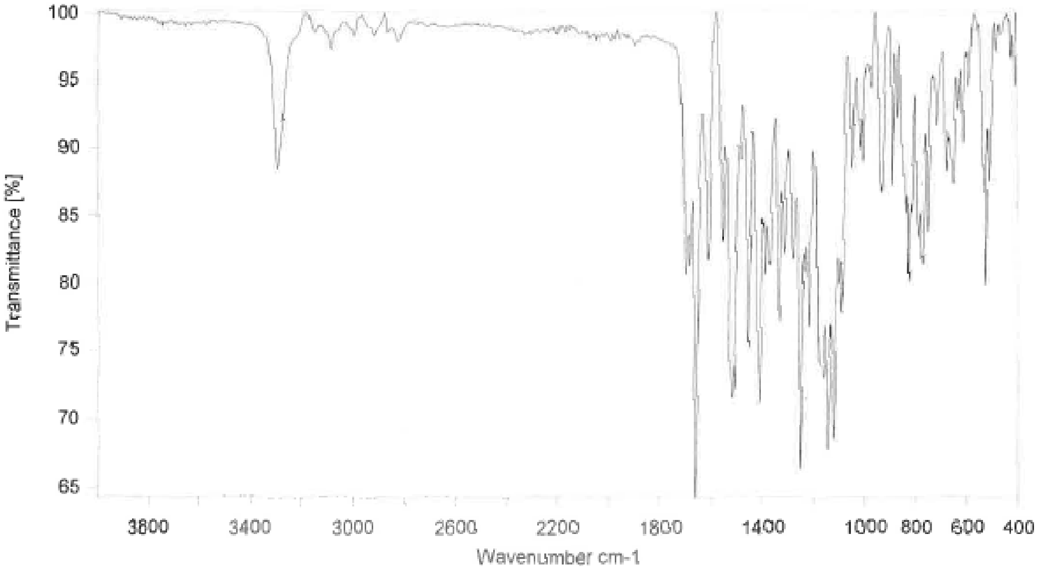

Wavelength	Absolute Intensity	Relative Intensity	Width	if Line < Shoulder	
3292.5946	0.884	0.116	49.7772	32.466015	0
3077.3538	0.973	0.027	49.7155	7.408878	0
2987.5810	0.982	0.012	32.0129	2.967676	0
2913.8034	0.983	0.017	47.7519	4.605954	0
2821.5604	0.979	0.018	40.3868	4.200316	0
2193.0930	0.984	0.008	14.3846	2.036808	0
1979.0906	0.979	0.009	21.0378	2.492479	0
1693.5939	0.807	0.058	26.1491	15.266386	0
1676.7296	0.812	0.036	26.7183	7.181674	0
1654.9034	0.642	0.358	18.9091	99.985603	0
1604.0028	0.816	0.137	19.9586	30.617662	0
1547.3284	0.831	0.105	249.3927	20.013756	0
1514.8788	0.715	0.250	33.6290	60.649254	0
1503.9511	0.721	0.038	29.1144	5.408106	0
1476.5456	0.892	0.025	31.4906	3.293617	0
1448.1411	0.752	0.170	14.6768	44.846920	0
1406.1239	0.711	0.232	23.1924	58.986645	0
1386.1368	0.806	0.054	8.9034	11.824996	0
1363.6462	0.814	0.067	4.7250	8.100092	0
1331.7205	0.771	0.145	13.2815	35.682434	0
1308.9693	0.821	0.059	11.7834	13.404267	0
1275.9826	0.817	0.060	15.9307	12.963675	0
1247.9321	0.663	0.337	48.6483	94.212570	0
1229.0634	0.808	0.018	6.2559	4.082520	0
1216.5871	0.768	0.076	8.6904	16.745605	0
1160.2348	0.729	0.064	206.2408	11.308853	0
1139.3154	0.677	0.244	73.1879	61.861923	0
1119.2612	0.685	0.099	10.9495	24.128151	0
1097.1372	0.799	0.019	4.8407	4.984043	0
1086.2191	0.778	0.061	9.1792	11.860849	0
1044.5586	0.885	0.088	16.1848	23.262064	0
1012.1706	0.899	0.033	26.2192	6.465026	0
1000.0495	0.890	0.078	25.7485	17.378578	0
962.5349	0.944	0.041	108.7796	5.260566	0
925.6887	0.868	0.127	19.8711	33.762939	0
882.8399	0.872	0.113	7.4613	29.044367	0
865.3222	0.922	0.050	9.3650	13.187500	0
833.7475	0.851	0.020	32.1936	4.127461	0
821.2613	0.800	0.199	37.9668	55.532303	0
783.3175	0.834	0.041	84.6367	5.264094	0
769.9366	0.814	0.133	32.7125	34.970161	0

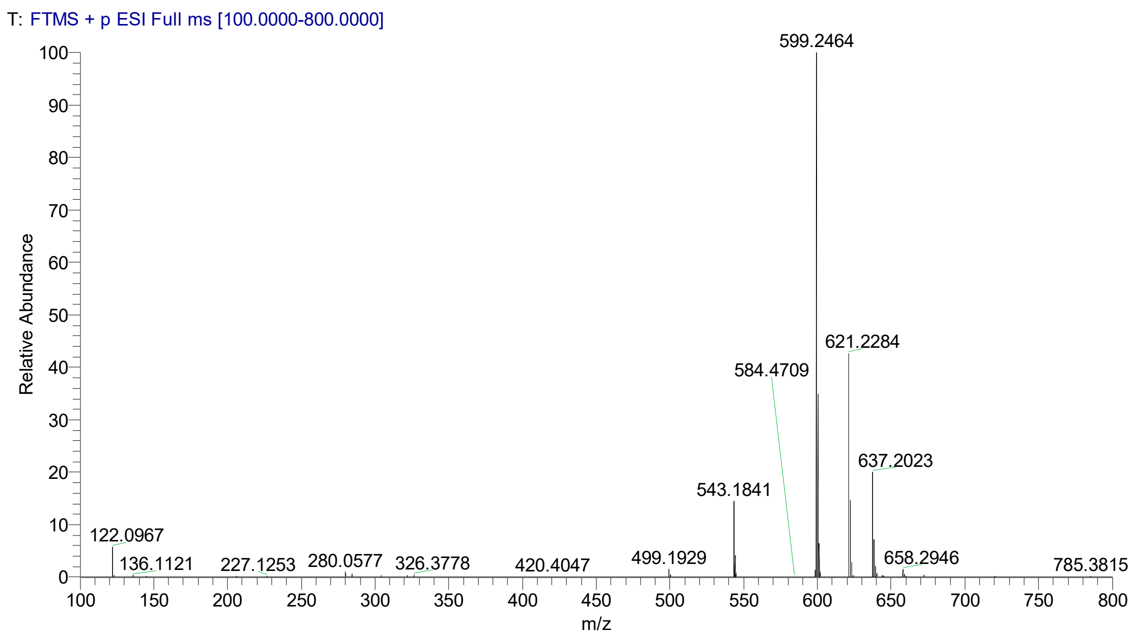
T: FTMS + p ESI Full ms [100.0000-800.0000]
599.2464
621.2284
584.4709
637.2023
543.1841
122.0967
136.1121
227.1253
280.0577
326.3778
420.4047
499.1929
658.2946
785.3815
Relative Abundance
m/z

Figure S8. FTIR and HRMS spectra, 1H NMR at 400 MHz and 13C NMR at 100 MHz spectra for compound **49** ($CDCl_3$)

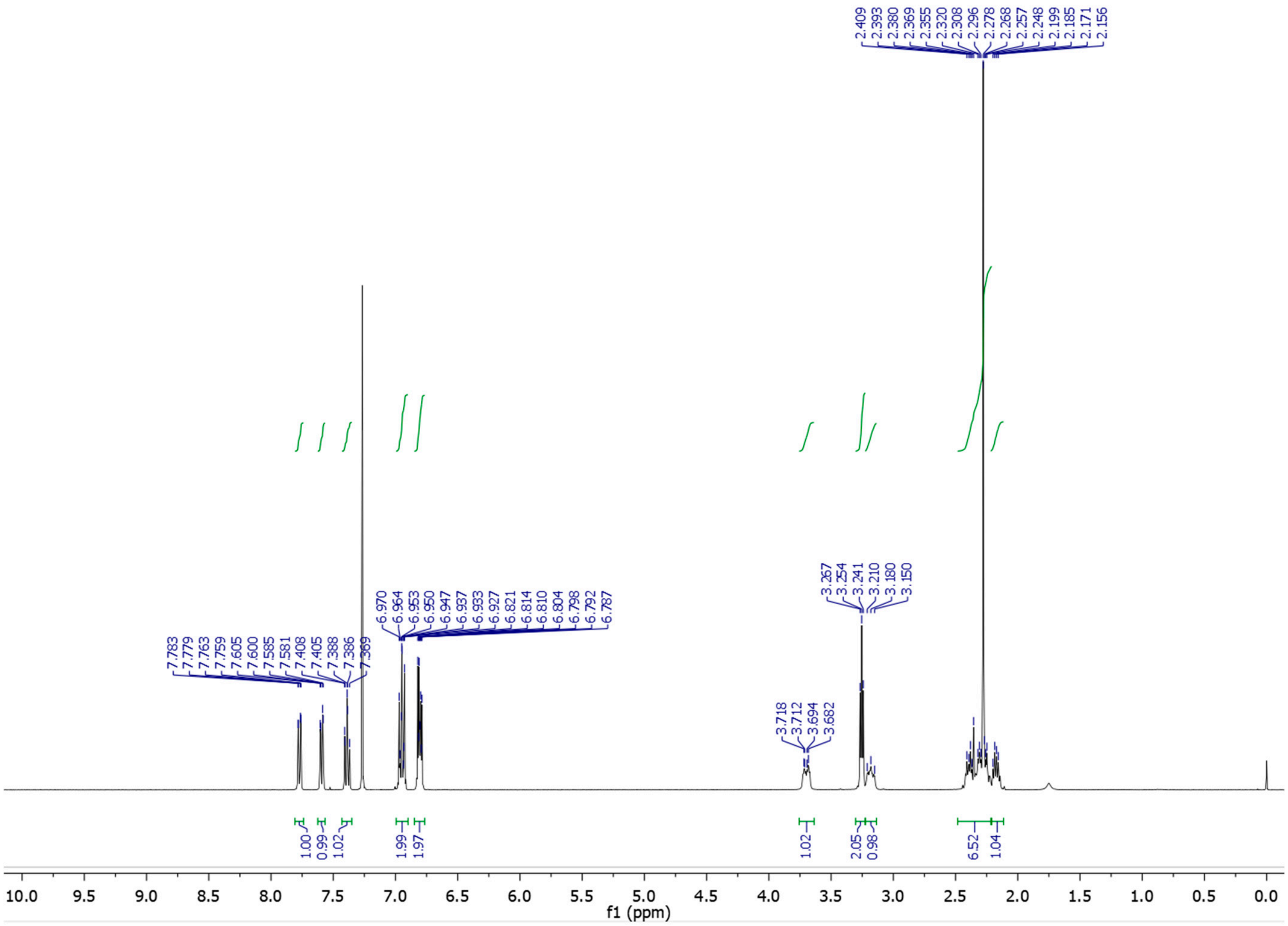

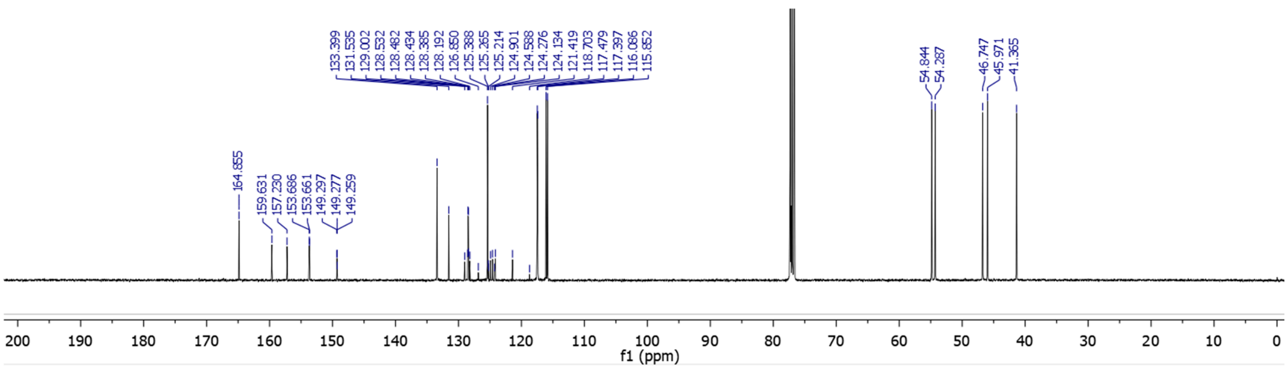

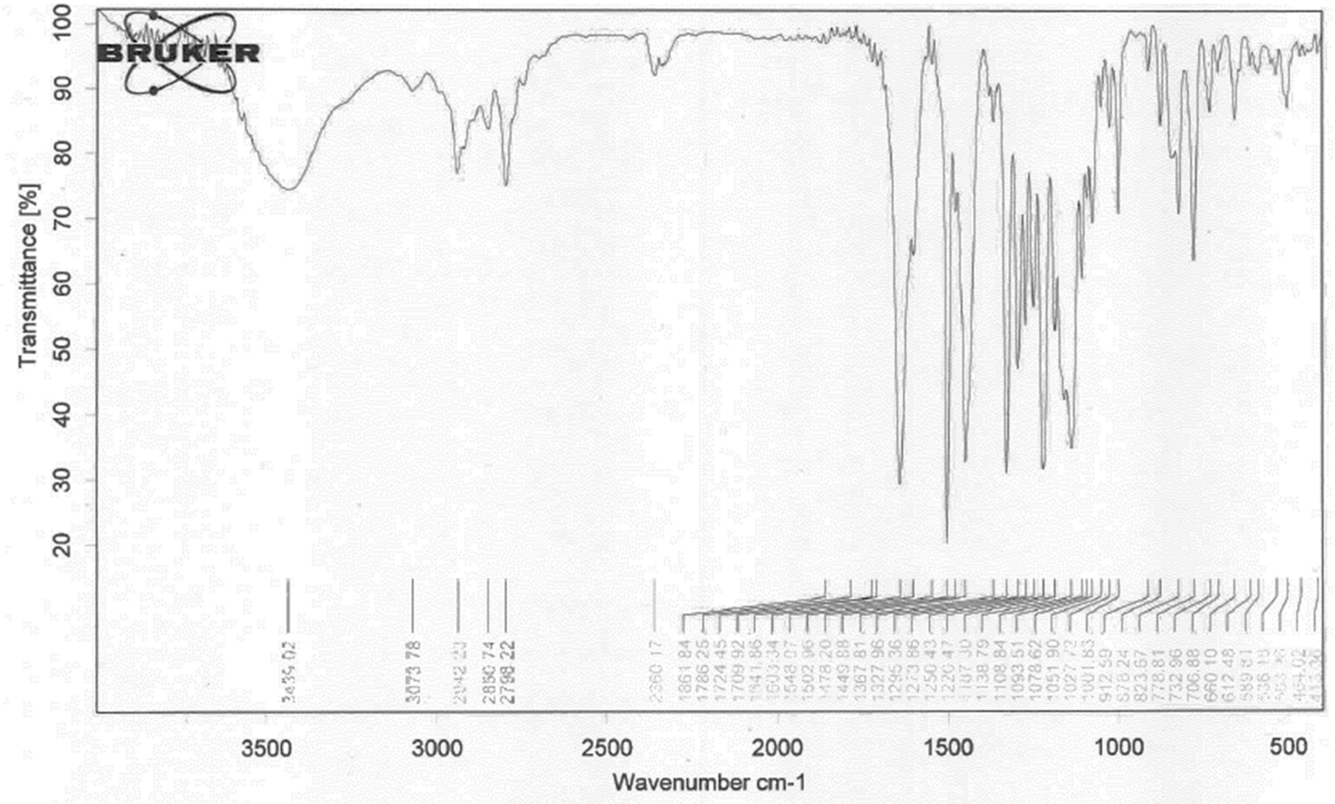
BRUKER
Transmittance [%]
Wavenumber cm-1
3435.02
3073.78
2942.20
2850.74
2798.22
2360.17
1861.84
1786.25
1724.45
1709.92
1641.86
1603.34
1548.07
1502.96
1478.20
1449.88
1367.81
1327.96
1295.36
1273.86
1250.43
1226.47
1187.30
1138.79
1108.84
1093.51
1078.62
1051.90
1027.72
1001.83
912.59
878.24
823.67
778.81
732.96
706.88
660.10
612.48
464.02

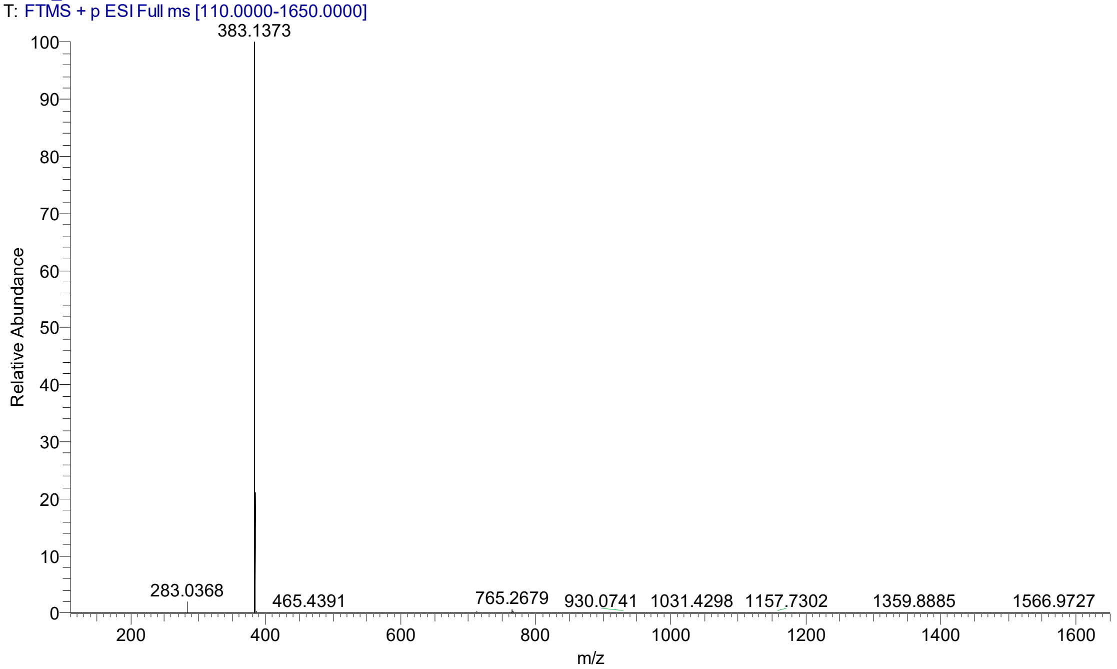
T: FTMS + p ESI Full ms [110.0000-1650.0000]
383.1373
Relative Abundance
283.0368
465.4391
765.2679
930.0741
1031.4298
1157.7302
1359.8885
1566.9727
m/z

Figure S9. FTIR and HRMS spectra, 1H NMR at 400 MHz and 13C NMR at 100 MHz spectra for compound **50** ($CDCl_3$)

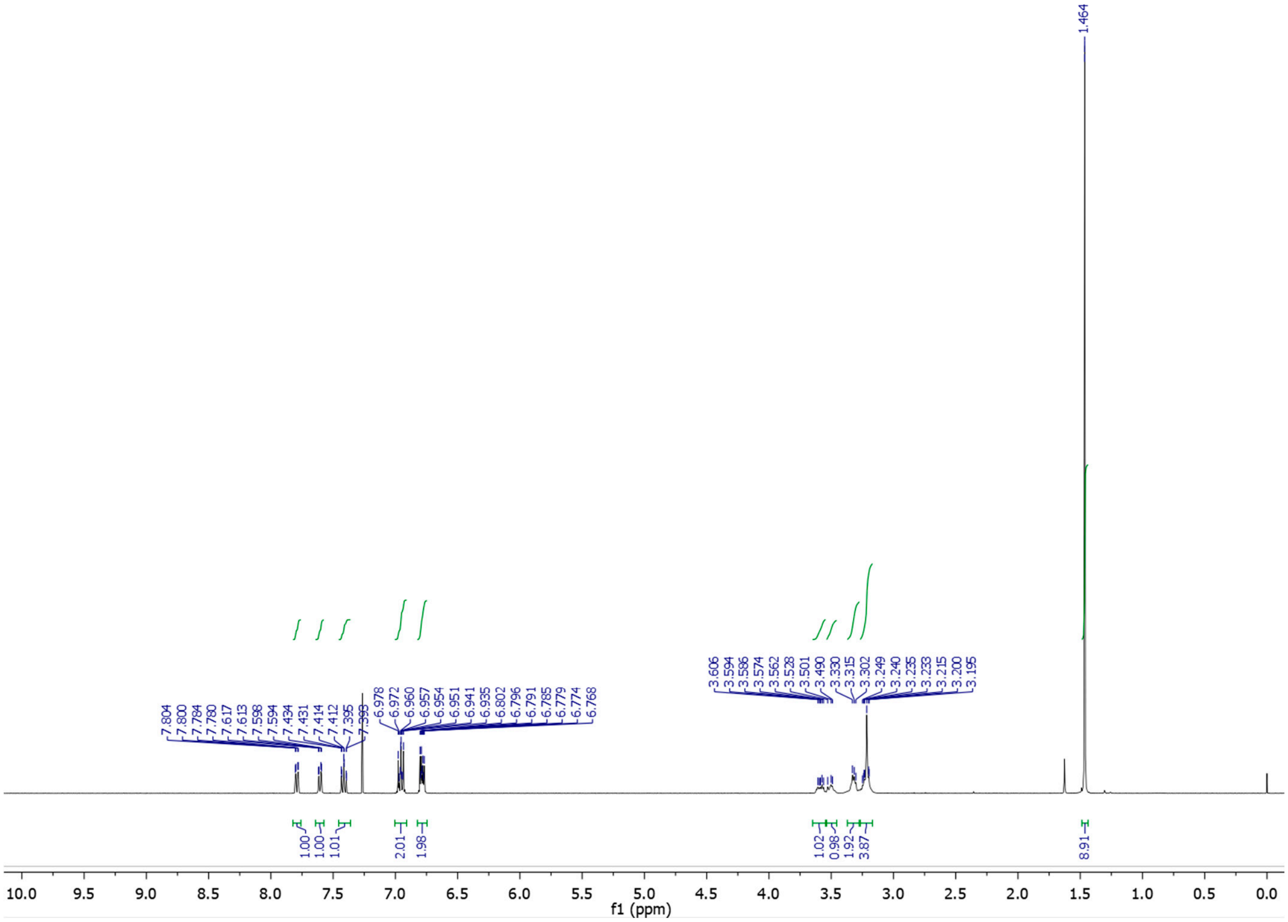

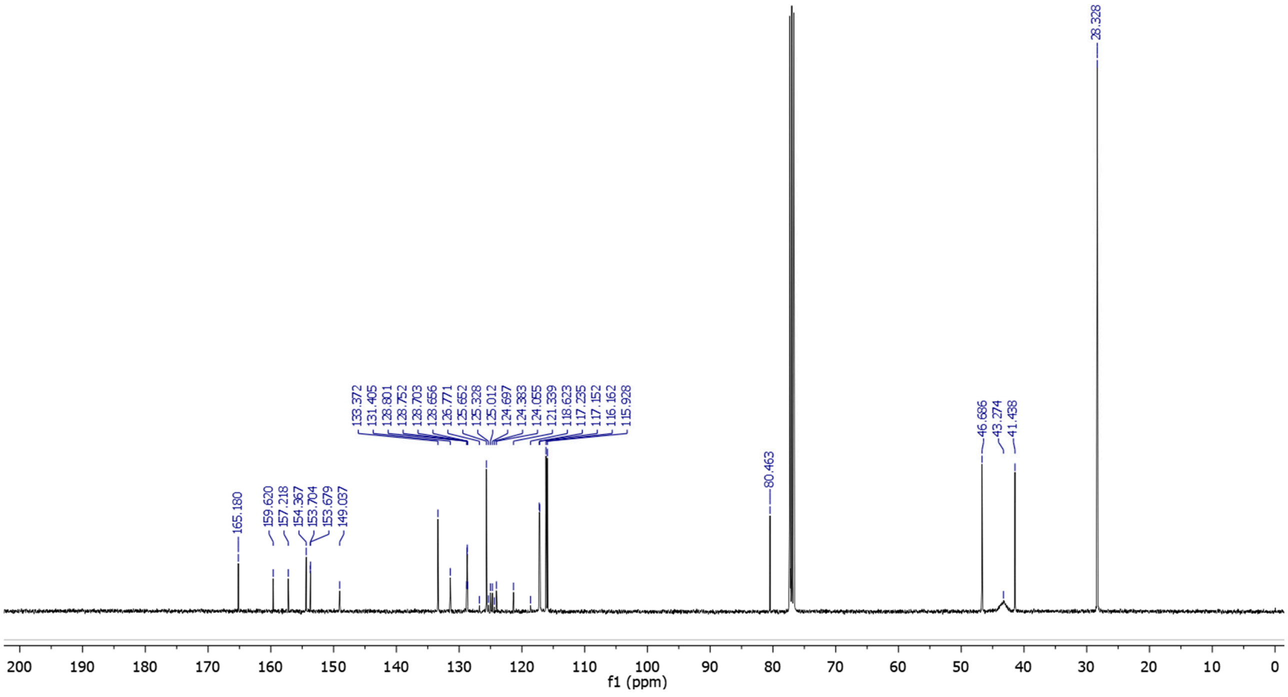

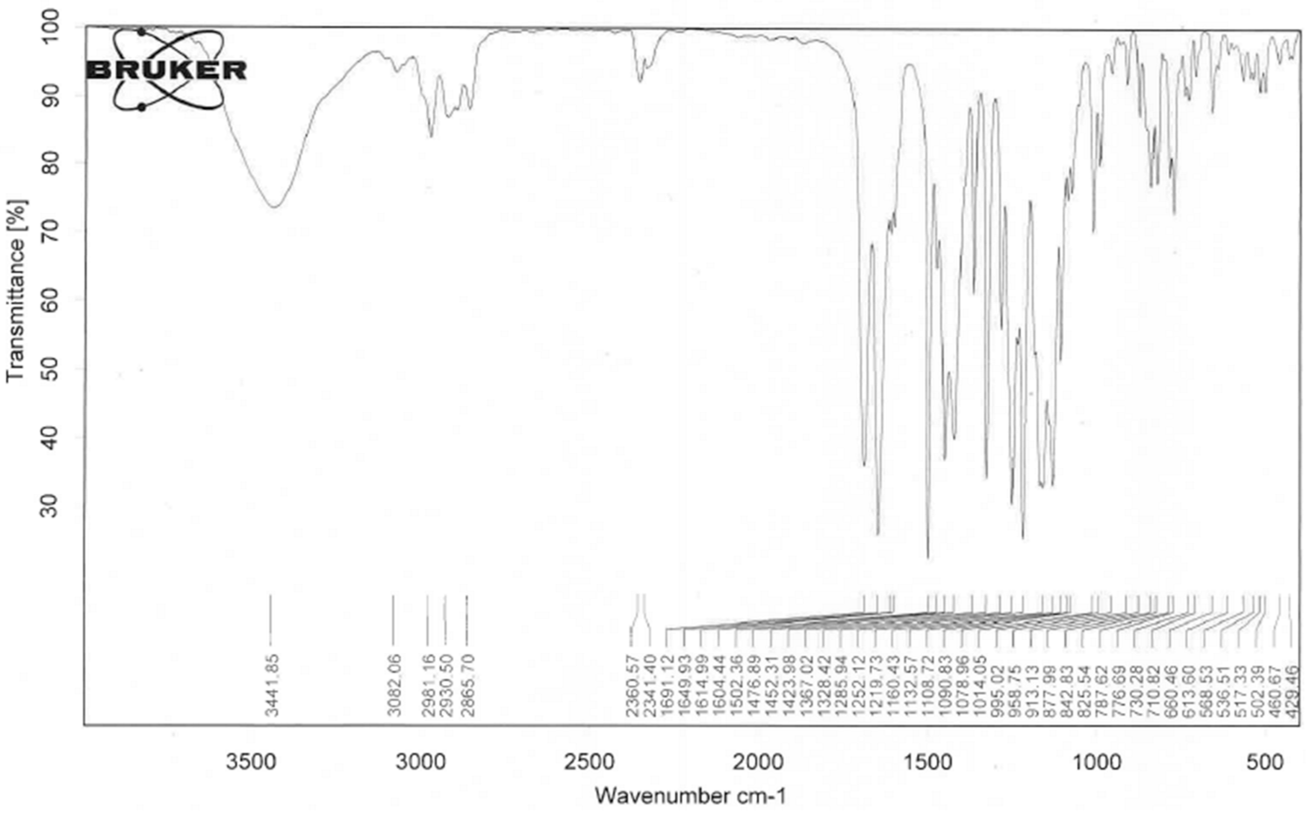
BRUKER
Transmittance [%]
Wavenumber cm-1
3441.85
3082.06
2981.16
2930.50
2865.70
2360.57
2341.40
1691.12
1649.93
1614.99
1604.44
1502.36
1476.89
1452.31
1423.98
1367.02
1328.42
1285.94
1252.12
1219.73
1160.43
1132.57
1108.72
1090.83
1078.96
1014.05
995.02
958.75
913.13
877.99
842.83
825.54
787.62
776.69
730.28
710.82
660.46
613.60
568.53
536.51
517.33
502.39
460.67
429.46

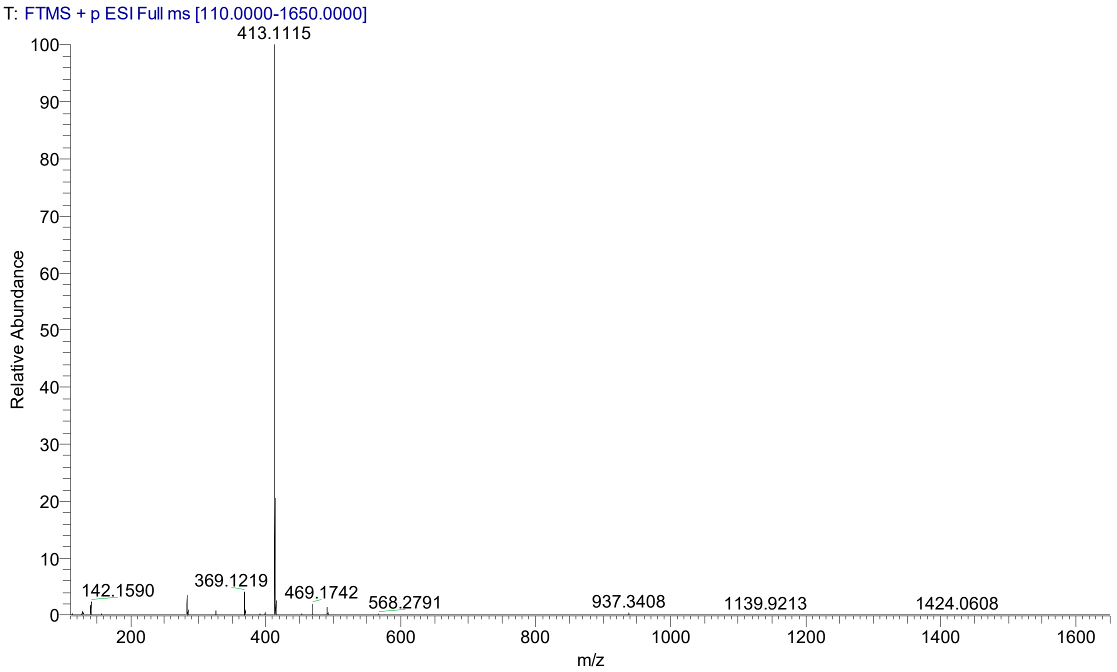
T: FTMS + p ESI Full ms [110.0000-1650.0000]
413.1115
Relative Abundance
142.1590
369.1219
469.1742
568.2791
937.3408
1139.9213
1424.0608
m/z

Figure S10. FTIR and HRMS spectra, 1H NMR at 400 MHz and 13C NMR at 100 MHz spectra for compound **51** ($CDCl_3$)

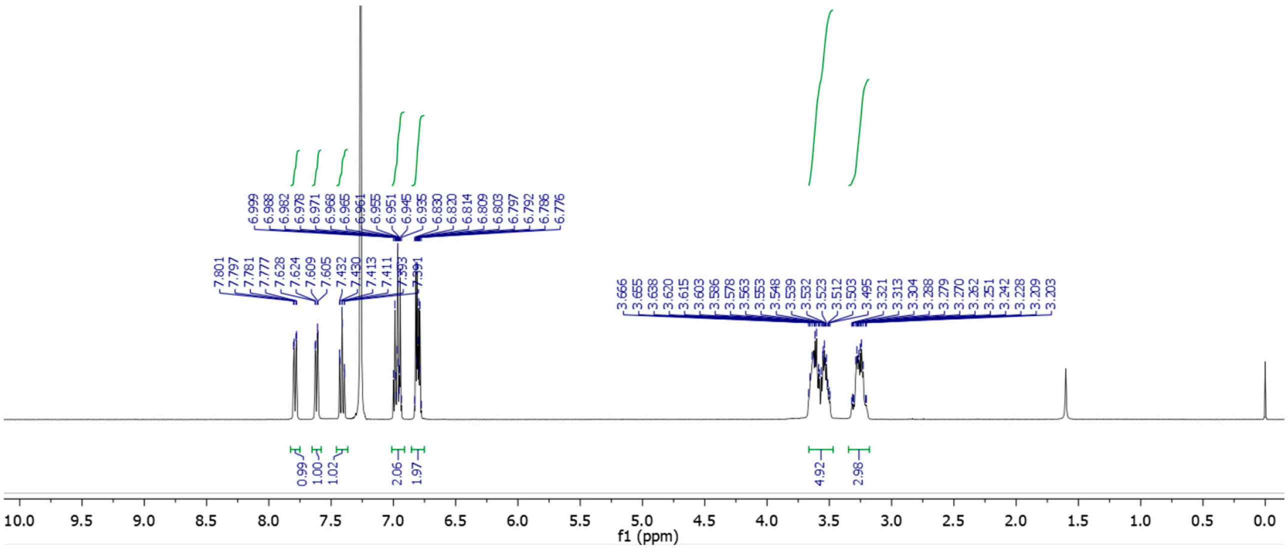

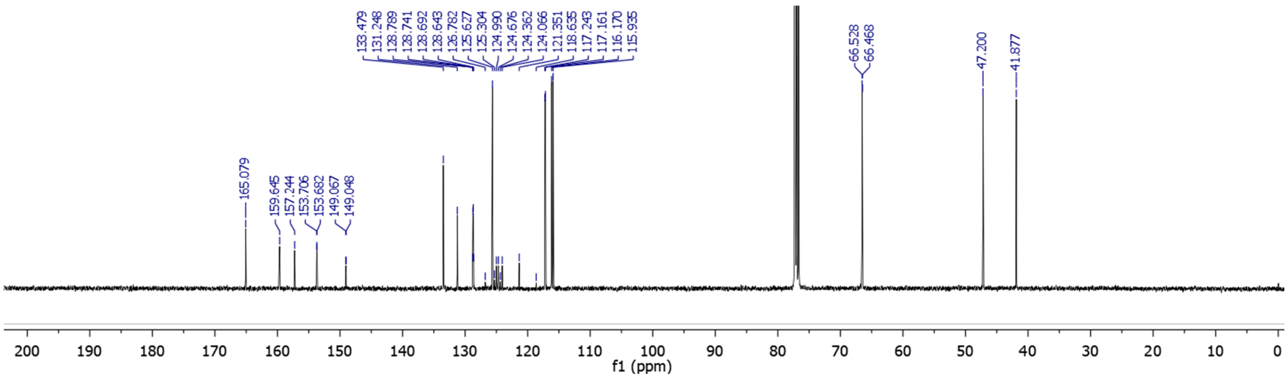

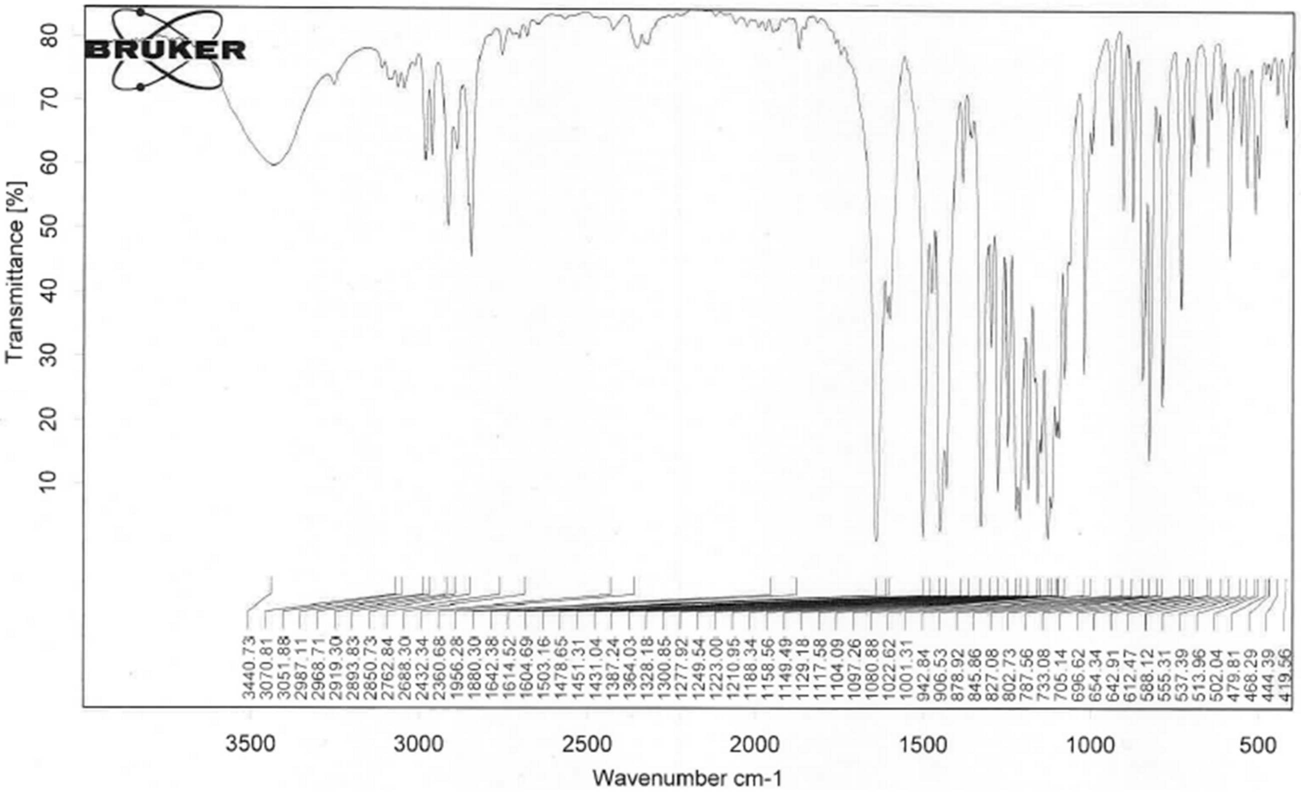
BRUKER
Transmittance [%]
Wavenumber cm-1
3440.73
3070.81
3051.88
2987.11
2968.71
2919.30
2893.83
2850.73
2762.84
2688.30
2432.34
2360.68
1956.28
1880.30
1642.38
1614.52
1604.69
1503.16
1478.65
1451.31
1431.04
1387.24
1364.03
1328.18
1300.85
1277.92
1249.54
1223.00
1210.95
1188.34
1158.56
1149.49
1129.18
1117.58
1104.09
1097.26
1080.88
1022.62
1001.31
942.84
906.53
878.92
845.86
827.08
802.73
787.56
733.08
705.14
696.62
654.34
642.91
612.47
588.12
555.31
537.39
513.96
502.04
479.81
468.29
444.39
419.56

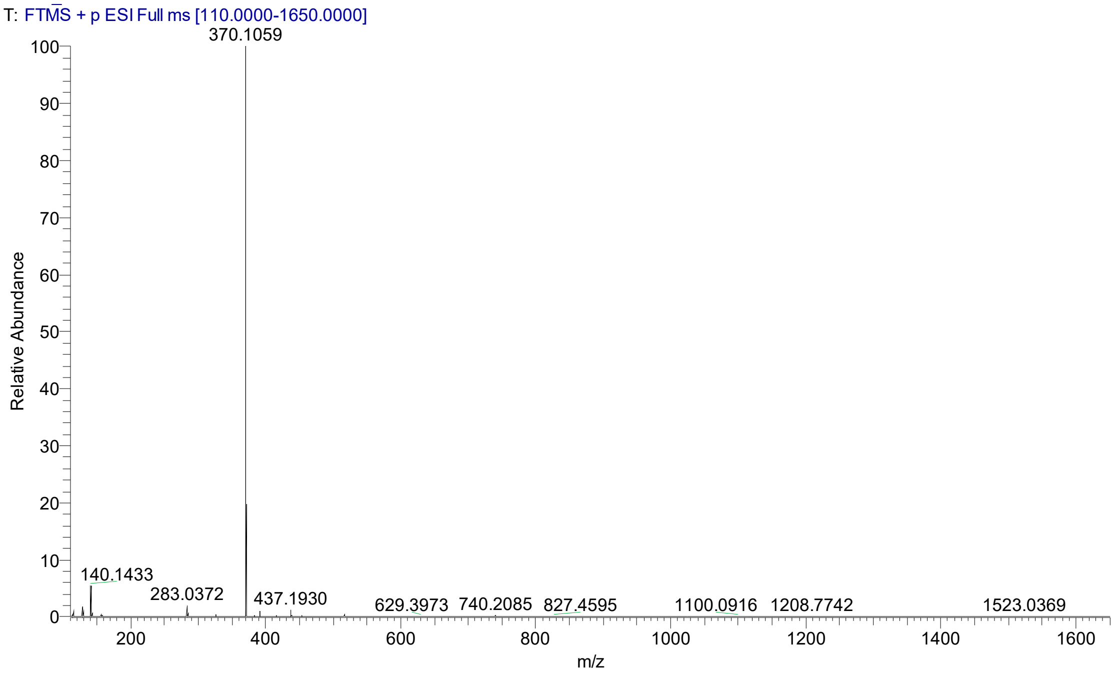
T: FTMS + p ESI Full ms [110.0000-1650.0000]
370.1059
140.1433
283.0372
437.1930
629.3973
740.2085
827.4595
1100.0916
1208.7742
1523.0369
Relative Abundance
m/z

Figure S11. FTIR and HRMS spectra, 1H NMR at 400 MHz and 13C NMR at 100 MHz spectra for compound **52** ($CDCl_3$)

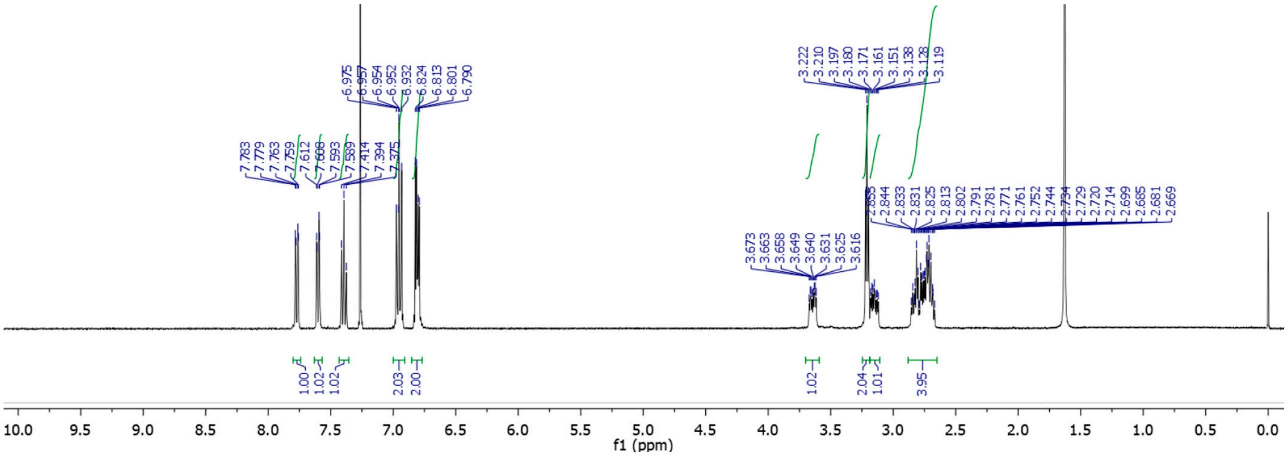

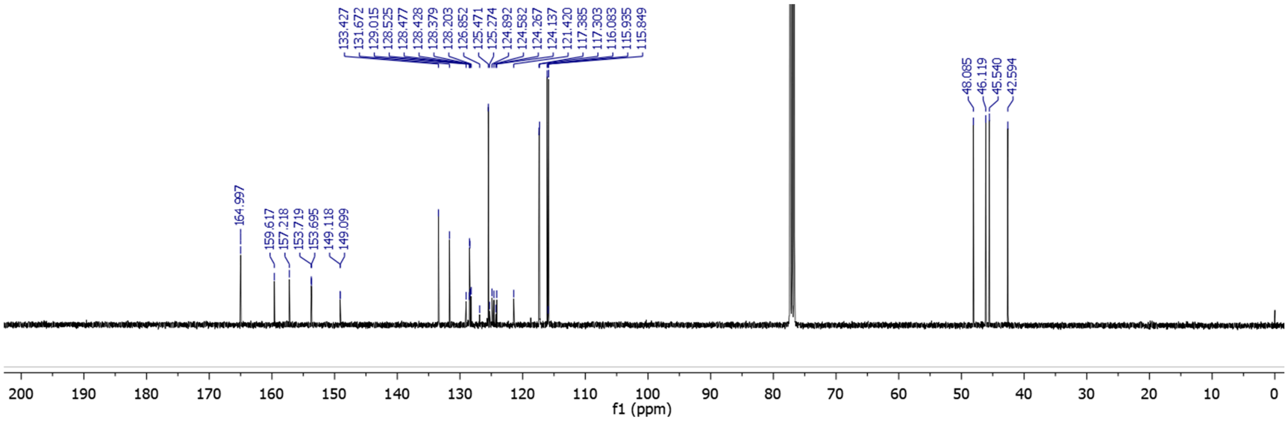

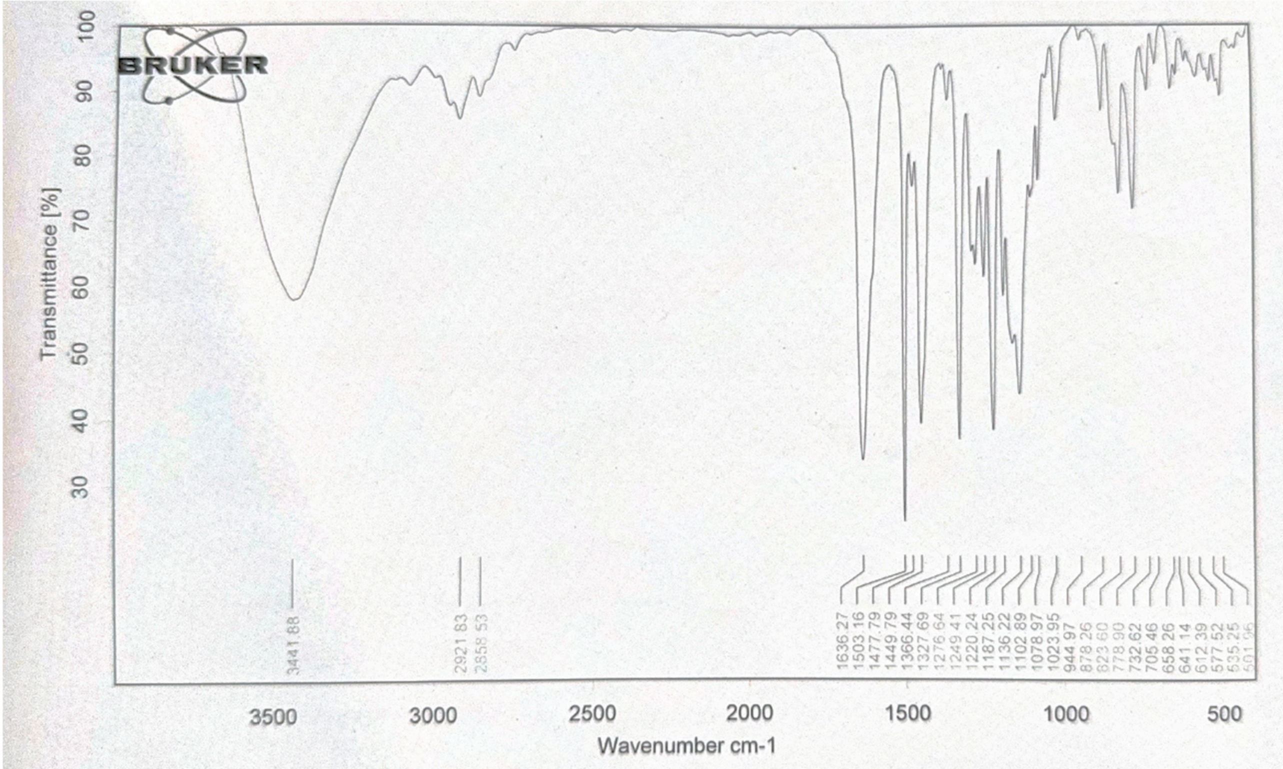
BRUKER
Transmittance [%]
Wavenumber cm-1
3441.88
2921.83
2858.53
1636.27
1503.16
1477.79
1449.79
1366.44
1327.69
1276.64
1249.41
1220.24
1187.25
1136.22
1102.89
1078.97
1023.95
944.97
878.26
823.60
778.90
732.62
705.46
658.26
641.14
612.39
577.52
535.25

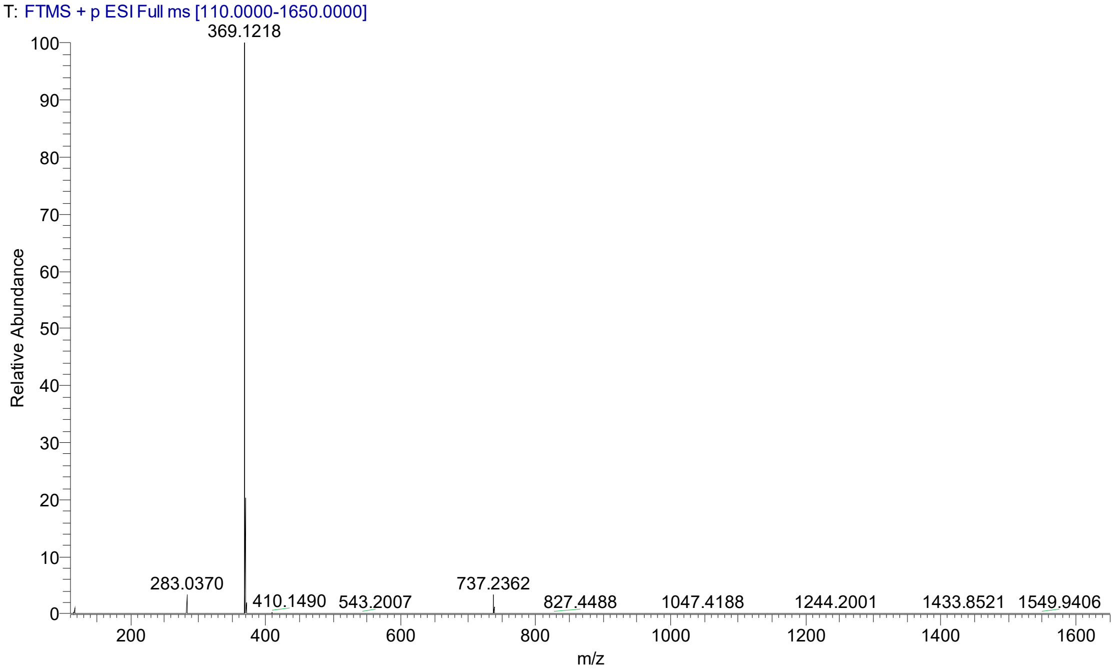
T: FTMS + p ESI Full ms [110.0000-1650.0000]
369.1218
283.0370
410.1490
543.2007
737.2362
827.4488
1047.4188
1244.2001
1433.8521
1549.9406
Relative Abundance
m/z

Figure S12. FTIR and HRMS spectra, 1H NMR at 400 MHz and 13C NMR at 100 MHz spectra for compound **53** ($CDCl_3$)

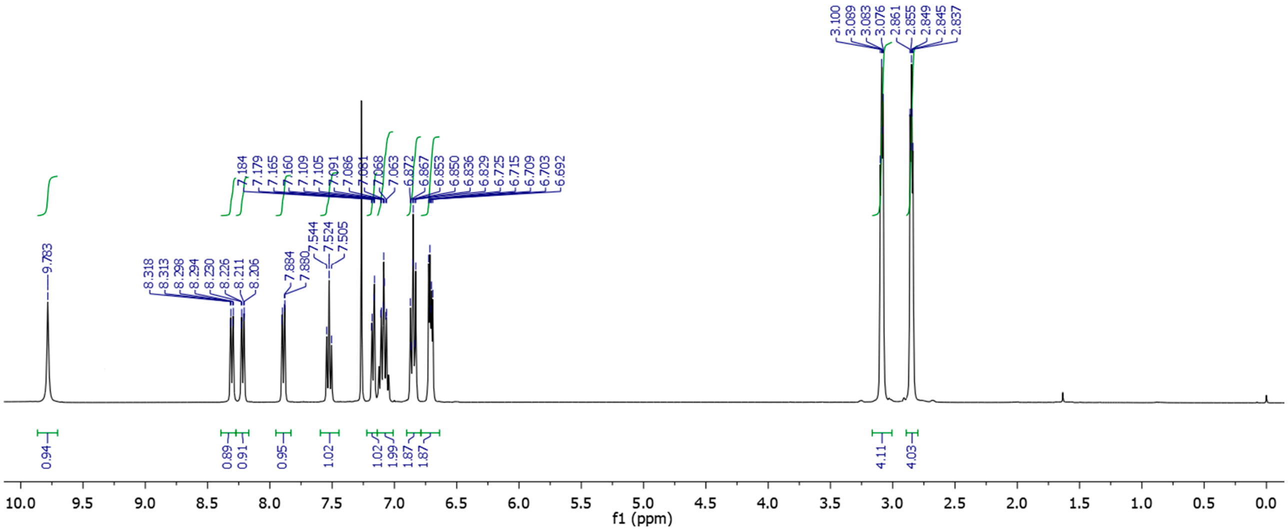

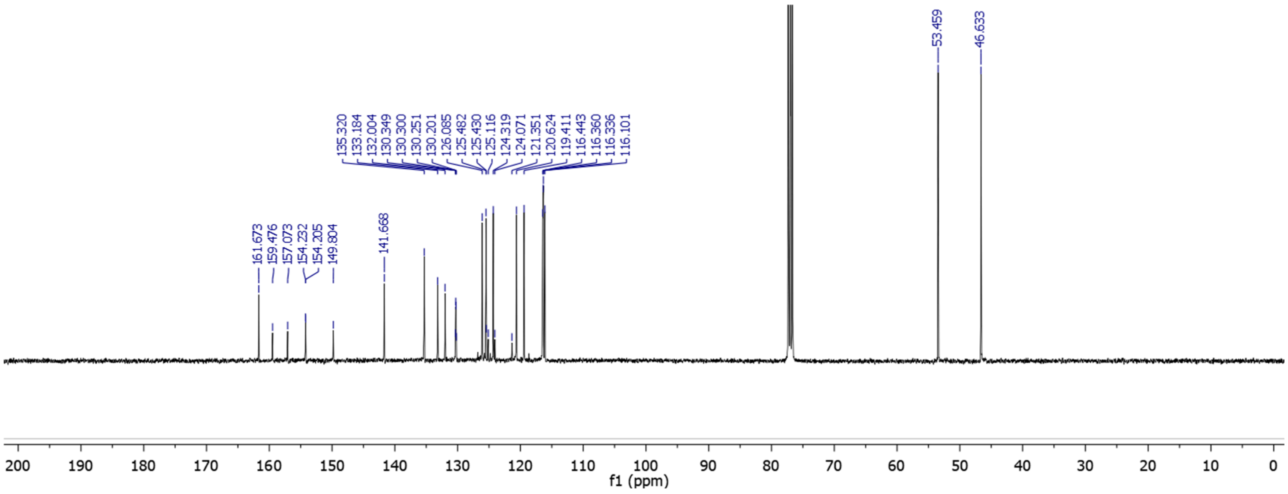

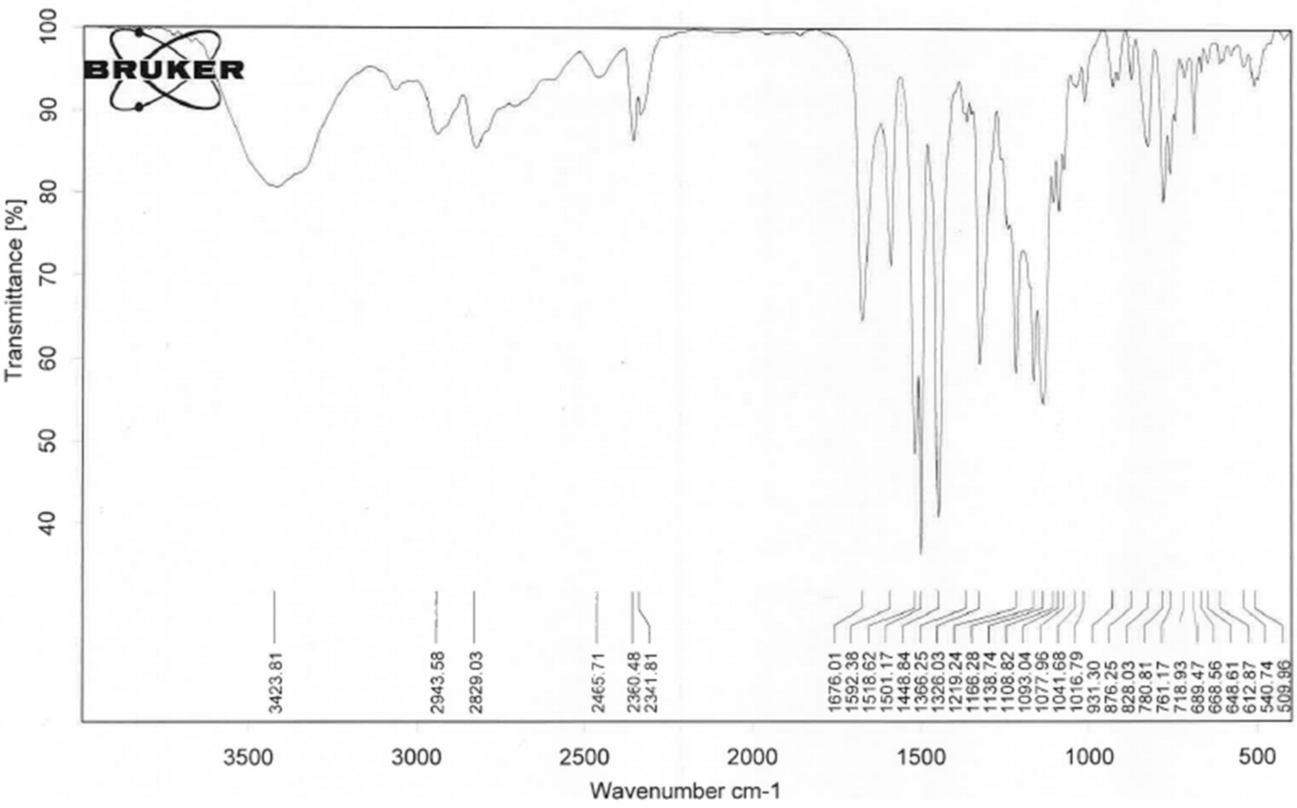
BRUKER
Transmittance [%]
100
90
80
70
60
50
40
3423.81
2943.58
2829.03
2465.71
2360.48
2341.81
1676.01
1592.38
1518.62
1501.17
1448.84
1366.25
1326.03
1219.24
1166.28
1138.74
1108.82
1093.04
1077.96
1041.68
1016.79
931.30
876.25
828.03
780.81
761.17
718.93
689.47
668.56
648.61
612.87
540.74
509.96
3500
3000
2500
2000
1500
1000
500
Wavenumber cm-1

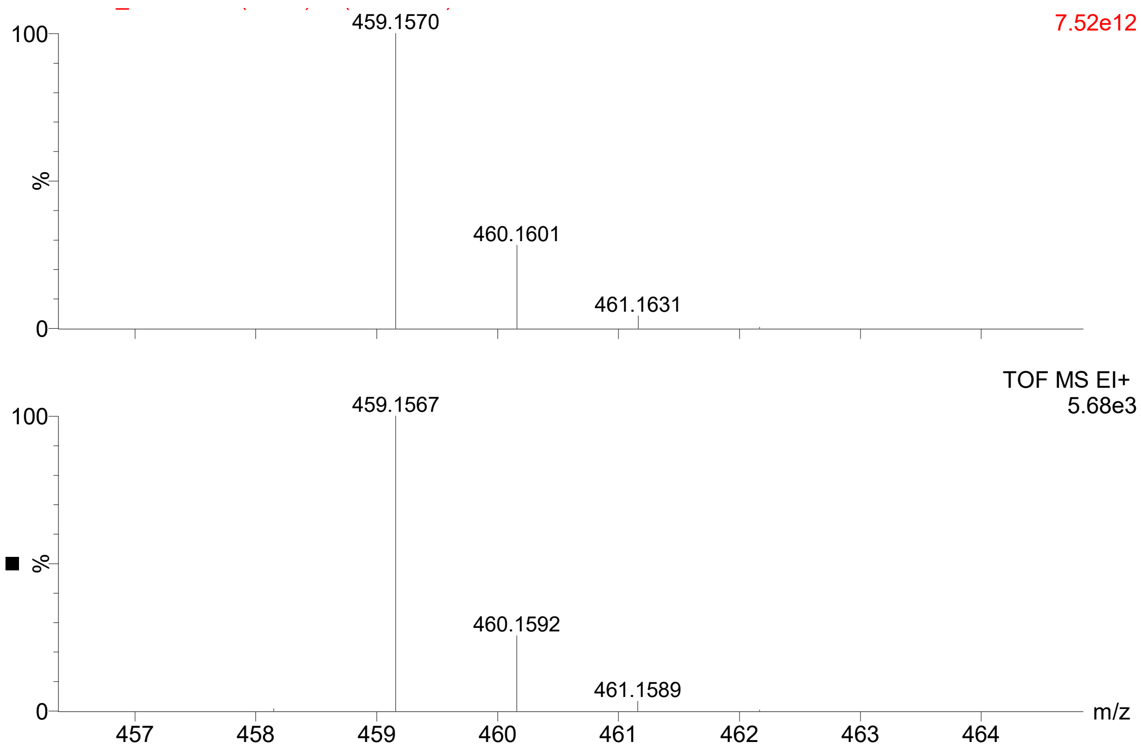
459.1570
7.52e12
100
%
460.1601
461.1631
0
TOF MS EI+
5.68e3
459.1567
100
%
460.1592
461.1589
0
m/z
457
458
459
460
461
462
463
464

Figure S13. FTIR and HRMS spectra, 1H NMR at 400 MHz and 13C NMR at 100 MHz spectra for compound **54** ($CDCl_3$)

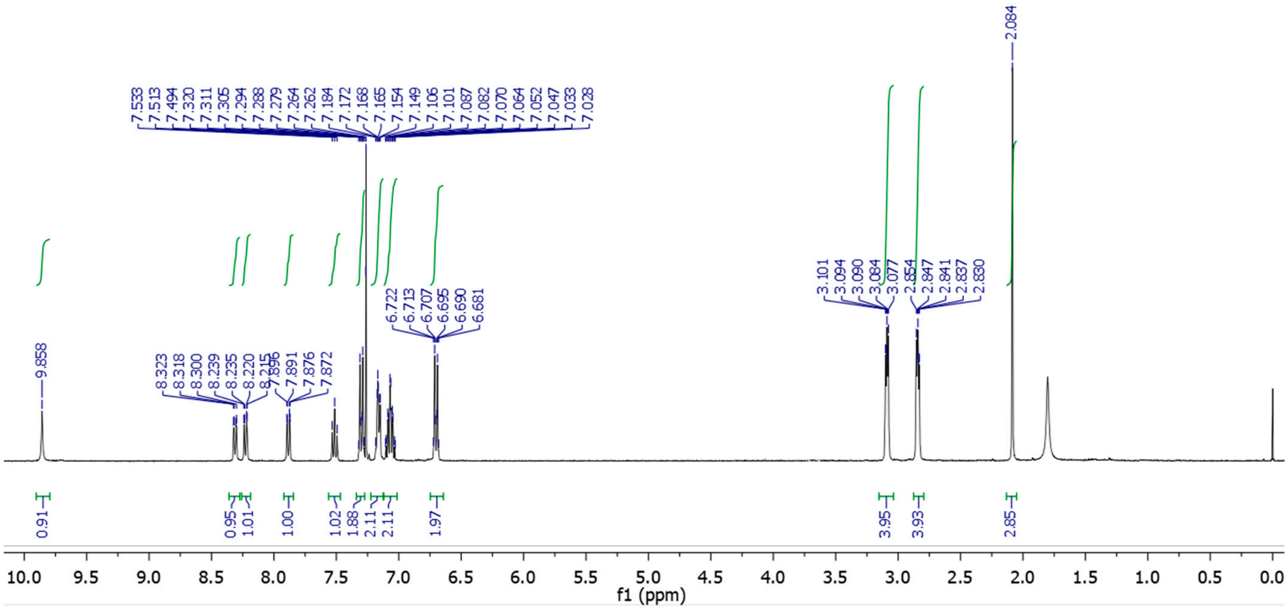

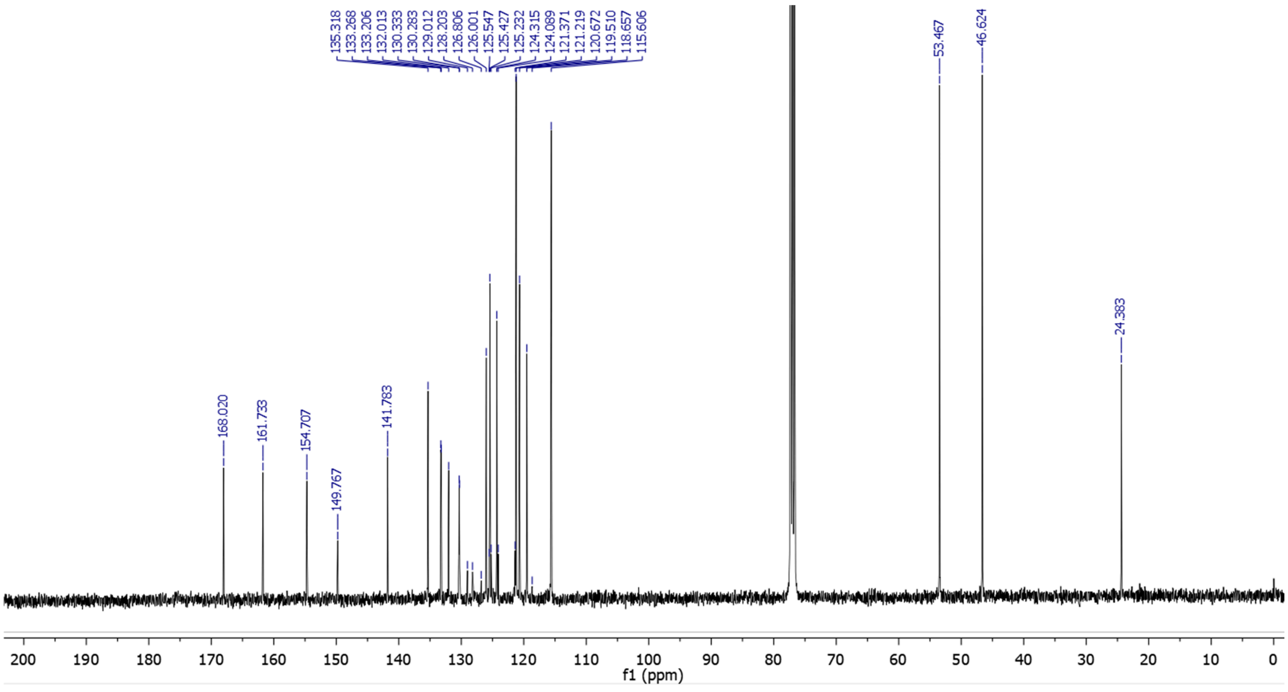

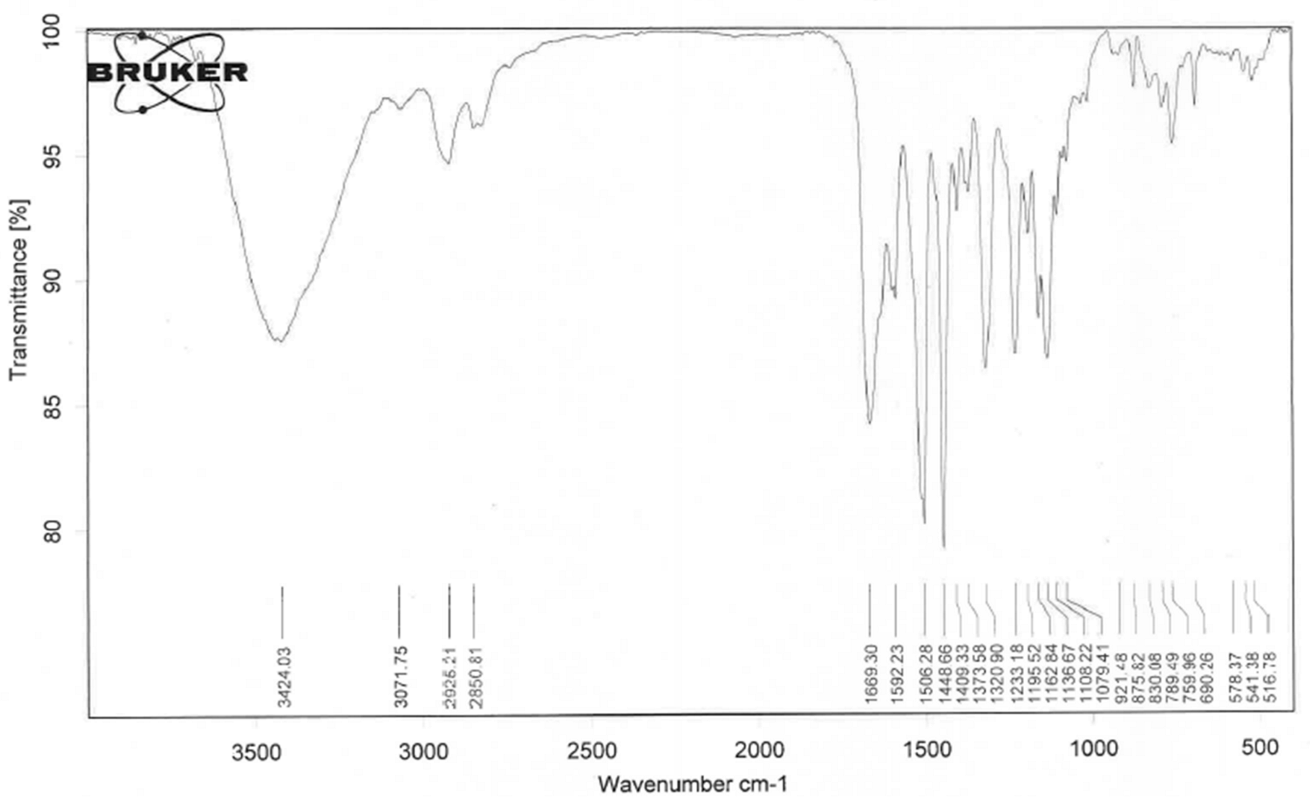
BRUKER
Transmittance [%]
Wavenumber cm-1
100
95
90
85
80
3500
3000
2500
2000
1500
1000
500
3424.03
3071.75
2926.21
2850.81
1669.30
1592.23
1506.28
1448.66
1409.33
1373.58
1320.90
1233.18
1195.52
1162.84
1136.67
1108.22
1079.41
921.48
875.82
830.08
789.49
759.96
690.26
578.37
541.38
516.18

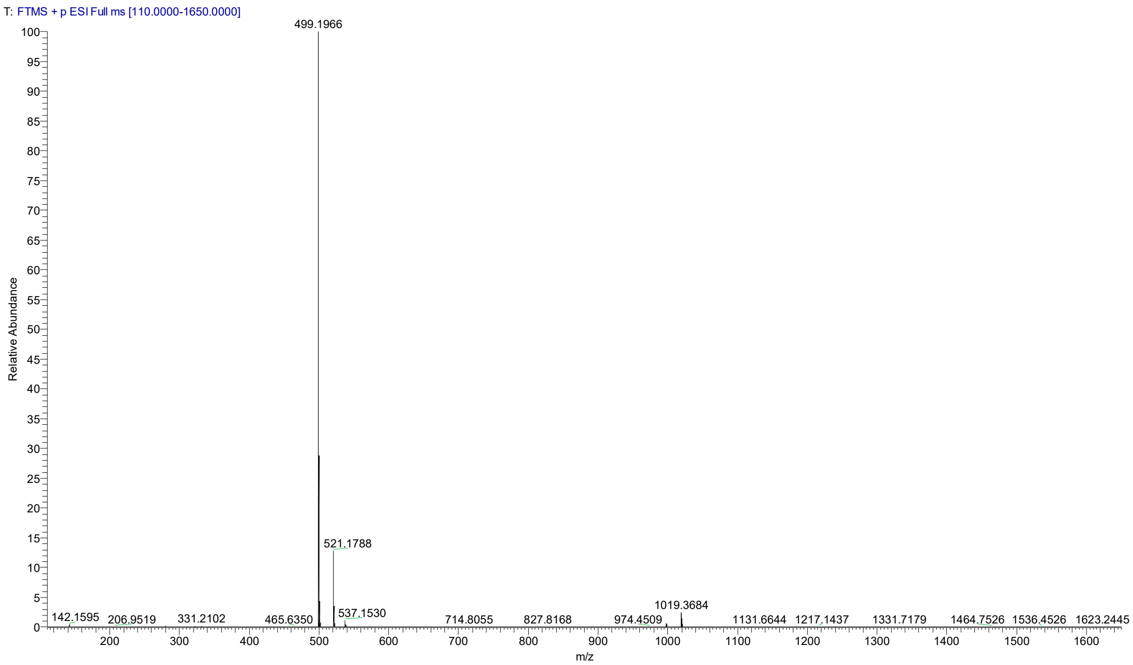
T: FTMS + p ESI Full ms [110.0000-1650.0000]
Relative Abundance
m/z
499.1966
521.1788
537.1530
1019.3684
142.1595
206.9519
331.2102
465.6350
714.8055
827.8168
974.4509
1131.6644
1217.1437
1331.7179
1464.7526
1536.4526
1623.2445

Figure S14. FTIR and HRMS spectra, 1H NMR at 400 MHz and 13C NMR at 100 MHz spectra for compound **55** ($CDCl_3$)

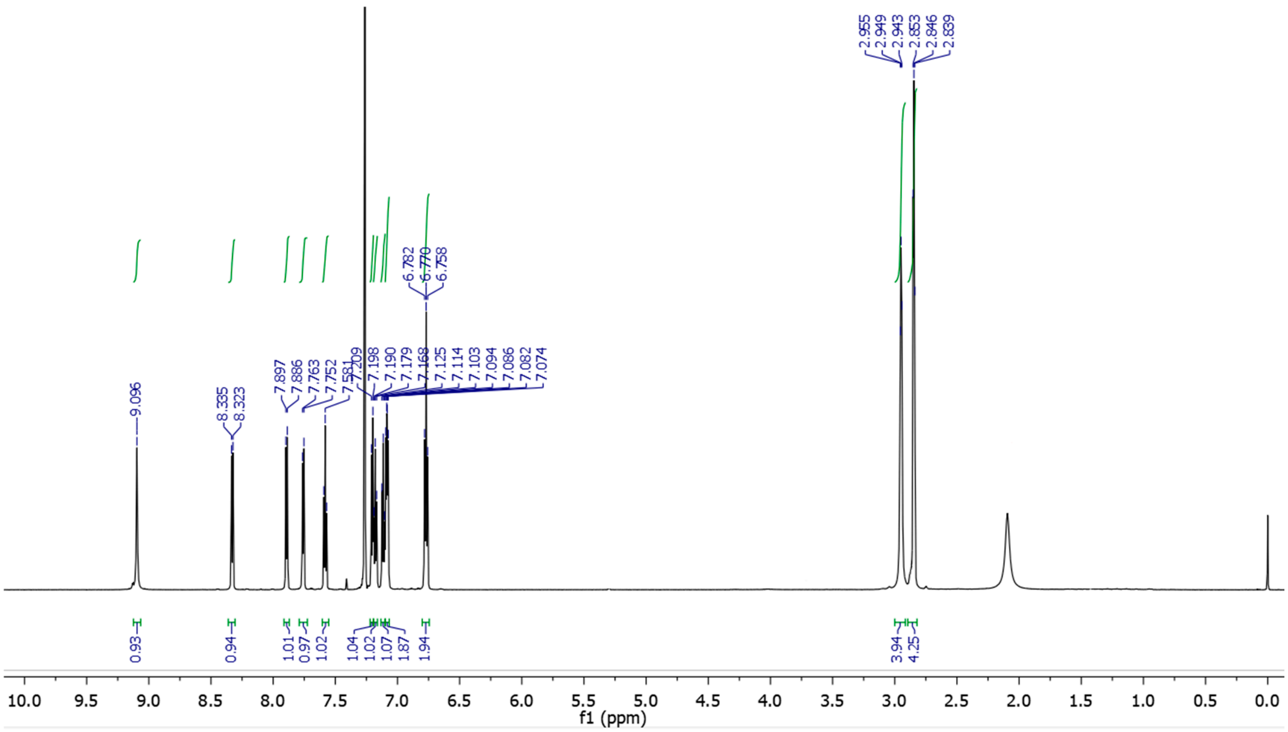

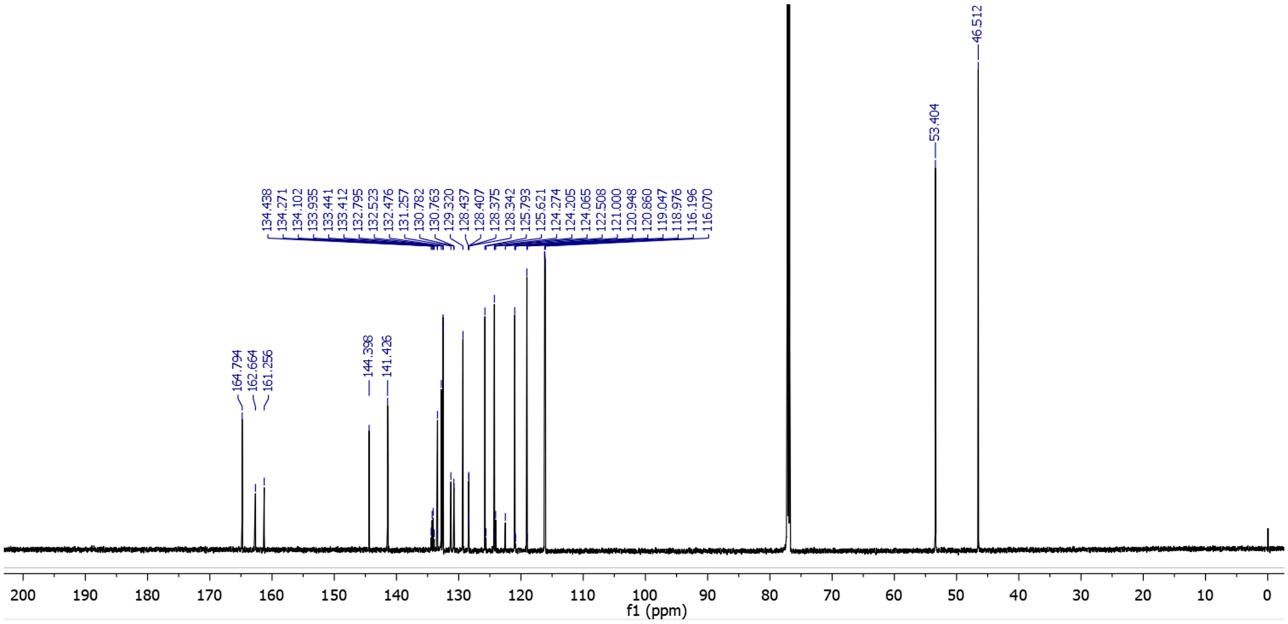

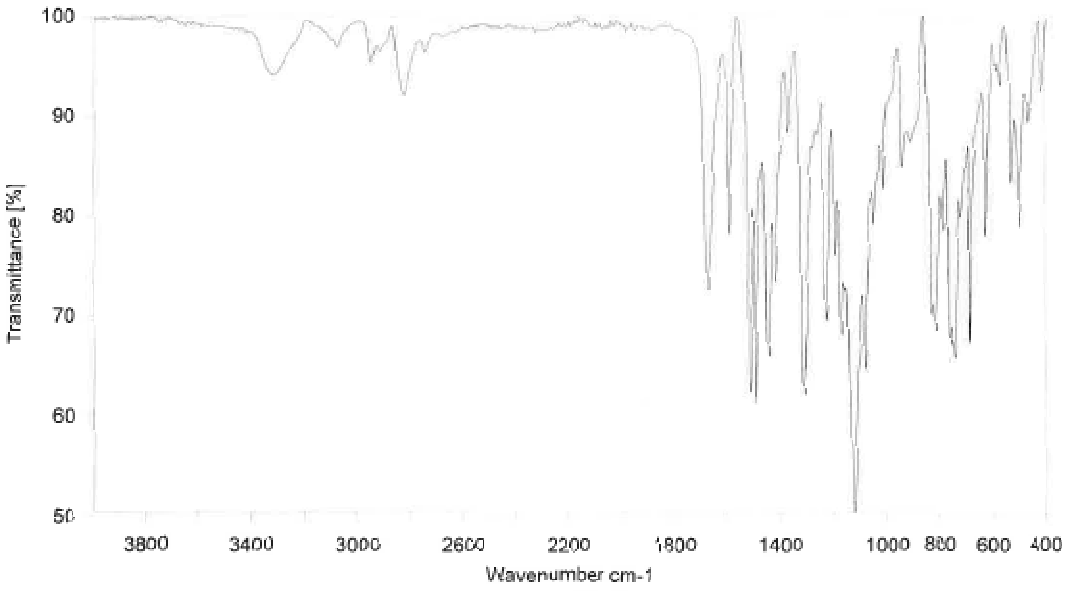

Wavelength	Absolute Intensity	Relative Intensity	Width	if Line <	Shoulder
3311.0731	0.942	0.057	113.7331	11.366195	0
3067.3487	0.970	0.026	59.9439	5.207452	0
2944.5178	0.956	0.037	67.4377	7.126399	0
2822.9912	0.922	0.078	51.5408	15.809667	0
2742.5715	0.965	0.012	17.7949	2.200915	0
1670.0936	0.727	0.273	39.6607	55.009659	0
1589.8431	0.781	0.198	14.4403	36.451733	0
1511.4236	0.626	0.188	16.0417	37.198109	0
1488.8307	0.613	0.361	14.5819	71.624779	0
1444.1051	0.661	0.214	23.0225	39.089172	0
1417.4185	0.736	0.081	116.3471	11.706748	0
1370.7309	0.884	0.071	14.3820	12.397522	0
1306.5807	0.623	0.323	27.7608	58.950386	0
1227.4569	0.697	0.205	18.6859	38.657734	0
1193.7693	0.761	0.081	41238.1829	11.238408	0
1170.8311	0.681	0.074	2388.3417	11.071179	0
1122.0434	0.505	0.496	106.3772	99.968468	0
1079.8922	0.649	0.088	11.2585	15.087232	0
1047.5442	0.792	0.041	8.3543	6.846390	0
1012.8664	0.828	0.051	8.0804	8.762295	0
936.3423	0.851	0.124	60.5892	23.943026	0
825.7378	0.704	0.047	215.7964	2.060911	0
813.6645	0.685	0.237	849.0277	34.769386	0
786.6876	0.788	0.050	62.9901	6.637544	0
757.9041	0.672	0.035	29.3230	3.632041	0
742.8677	0.659	0.341	57.8995	68.321602	0
719.4502	0.798	0.030	257.5156	3.836282	0
685.5920	0.674	0.201	9.6558	39.625198	0
626.4414	0.781	0.150	13.0748	29.249987	0

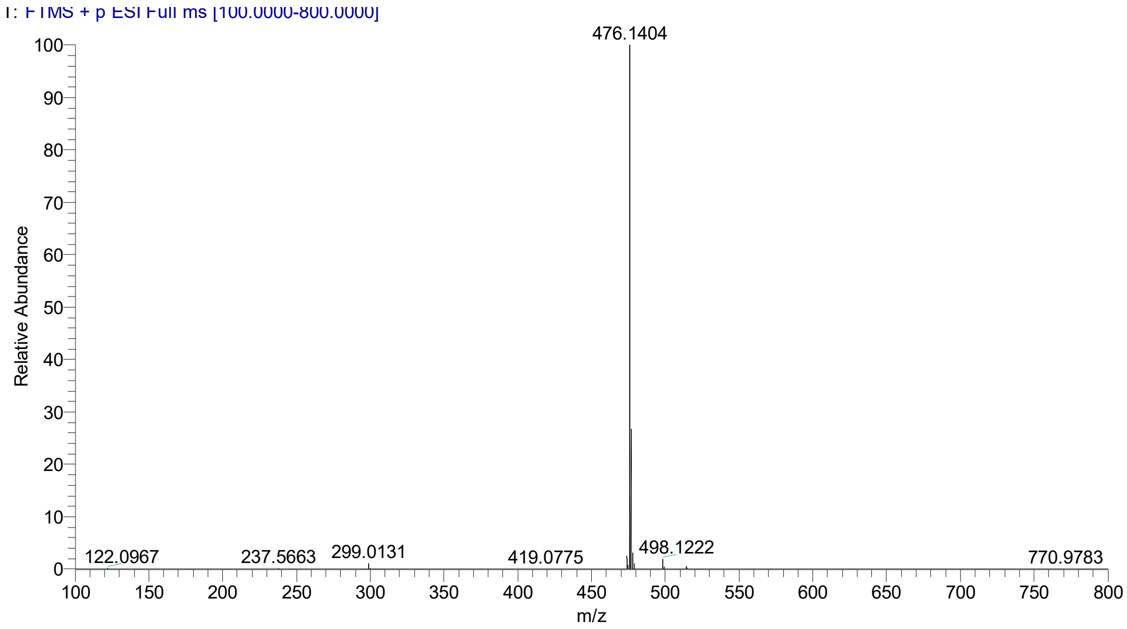

T: FTMS + p ESI Full ms [100.0000-800.0000]
476.1404
Relative Abundance
m/z
122.0967
237.5663
299.0131
419.0775
498.1222
770.9783
0
10
20
30
40
50
60
70
80
90
100
100
150
200
250
300
350
400
450
500
550
600
650
700
750
800

Figure S15. FTIR and HRMS spectra, 1H NMR at 400 MHz and 13C NMR at 100 MHz spectra for compound **56** ($CDCl_3$)

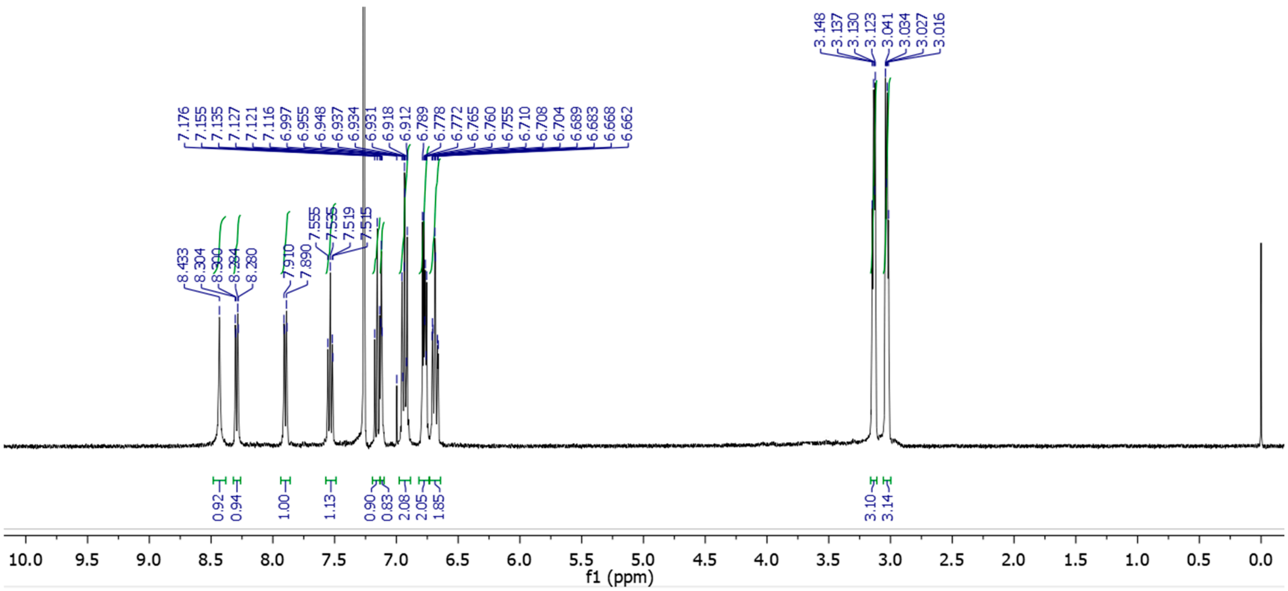

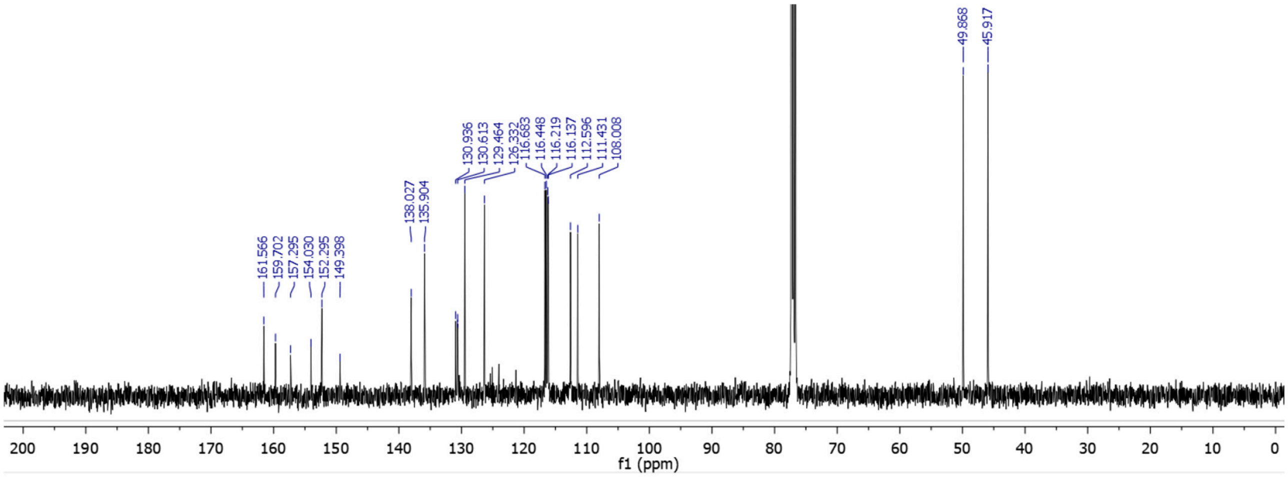

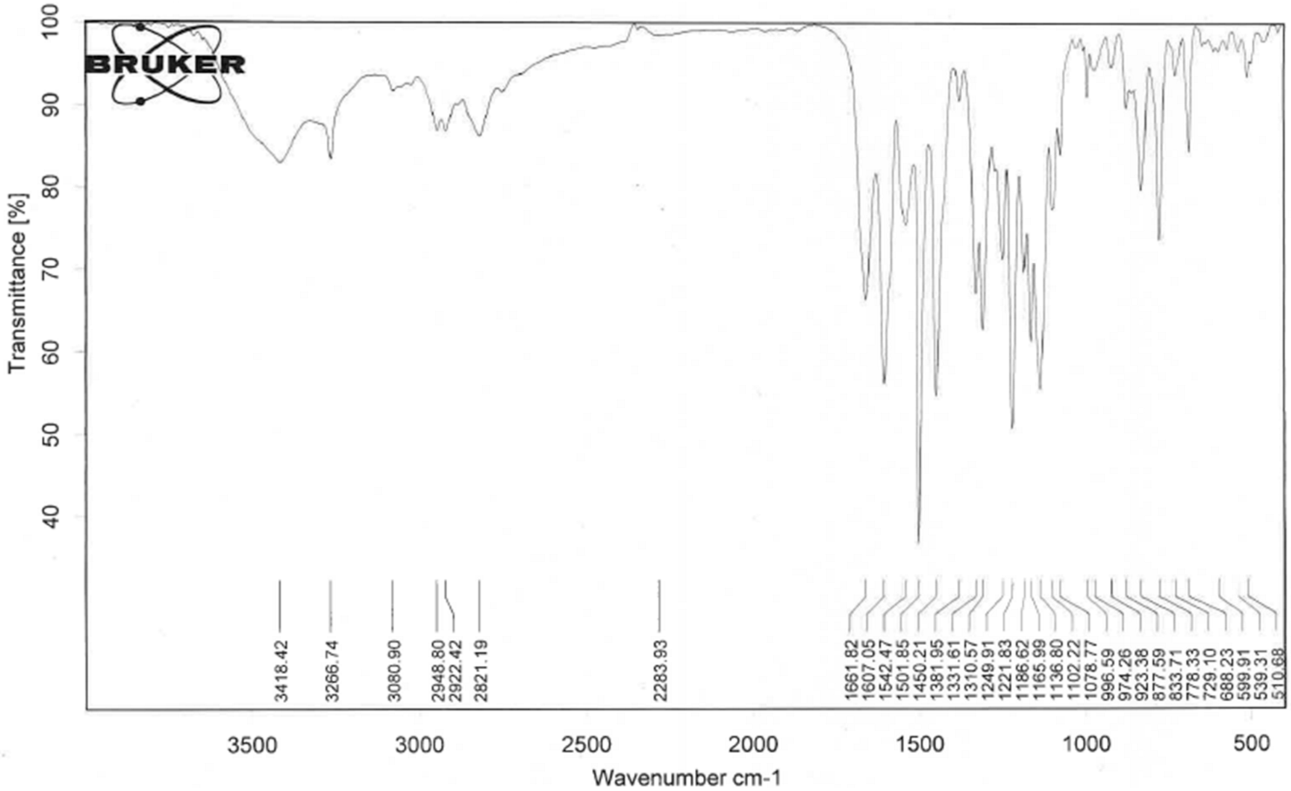

BRUKER
Transmittance [%]
Wavenumber cm-1
3418.42
3266.74
3080.90
2948.80
2922.42
2821.19
2283.93
1661.82
1607.05
1542.47
1501.85
1450.21
1381.95
1331.61
1310.57
1249.91
1221.83
1186.62
1165.99
1136.80
1102.22
1078.77
996.59
974.26
923.38
877.59
833.71
778.33
729.10
688.23
599.91
539.31
510.68

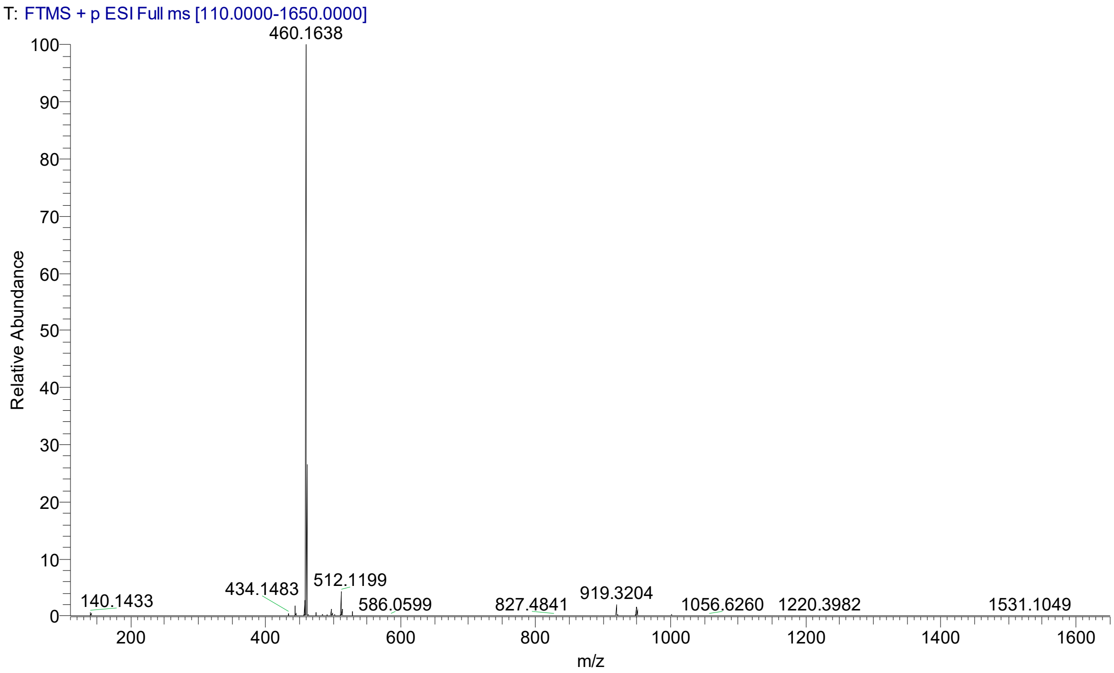

T: FTMS + p ESI Full ms [110.0000-1650.0000]
Relative Abundance
m/z
460.1638
140.1433
434.1483
512.1199
586.0599
827.4841
919.3204
1056.6260
1220.3982
1531.1049

Figure S16. FTIR and HRMS spectra, 1H NMR at 400 MHz and 13C NMR at 100 MHz spectra for compound **57** ($CDCl_3$)

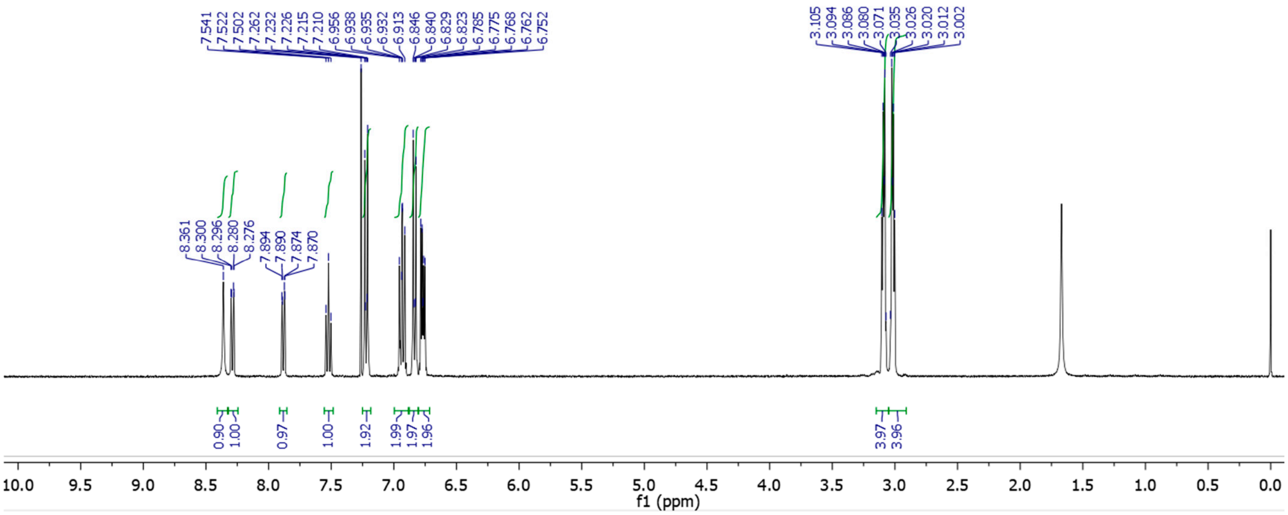

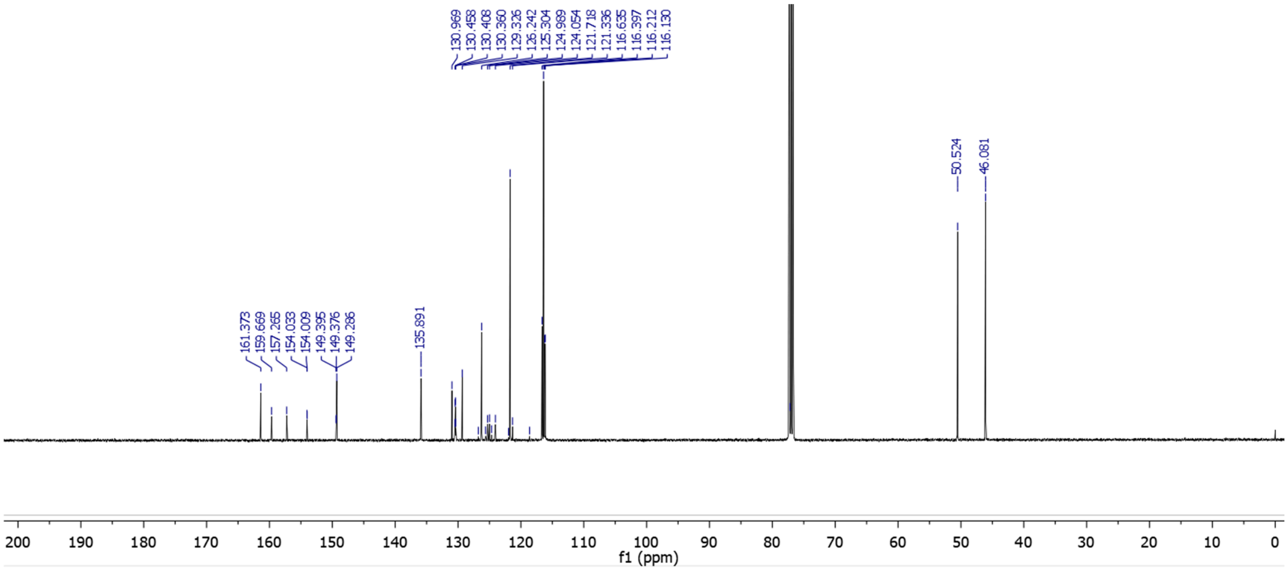

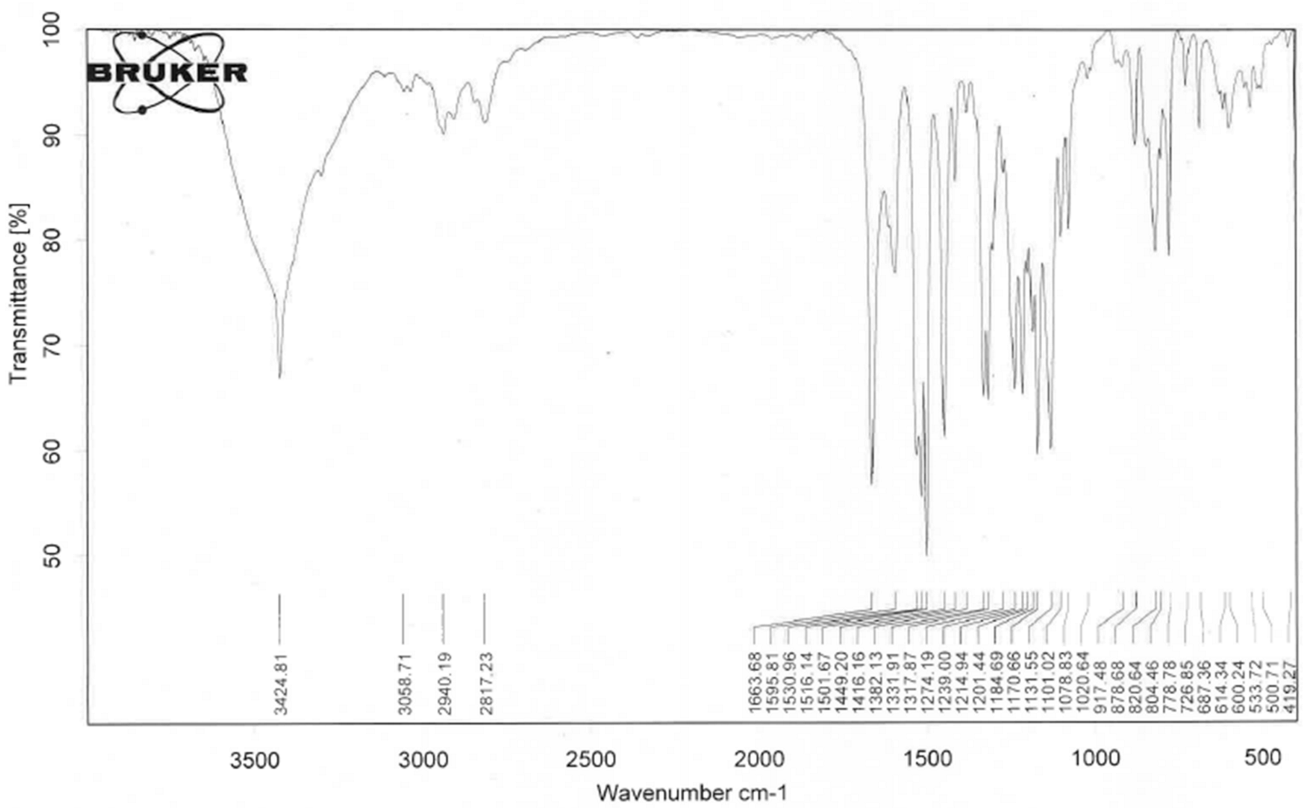
BRUKER
Transmittance [%]
100
90
80
70
60
50
3424.81
3058.71
2940.19
2817.23
1663.68
1595.81
1530.96
1516.14
1501.67
1449.20
1416.16
1382.13
1331.91
1317.87
1274.19
1239.00
1214.94
1201.44
1184.69
1170.66
1131.55
1101.02
1078.83
1020.64
917.48
878.68
820.64
804.46
778.78
726.85
687.36
614.34
600.24
533.72
500.71
419.27
3500
3000
2500
2000
1500
1000
500
Wavenumber cm-1

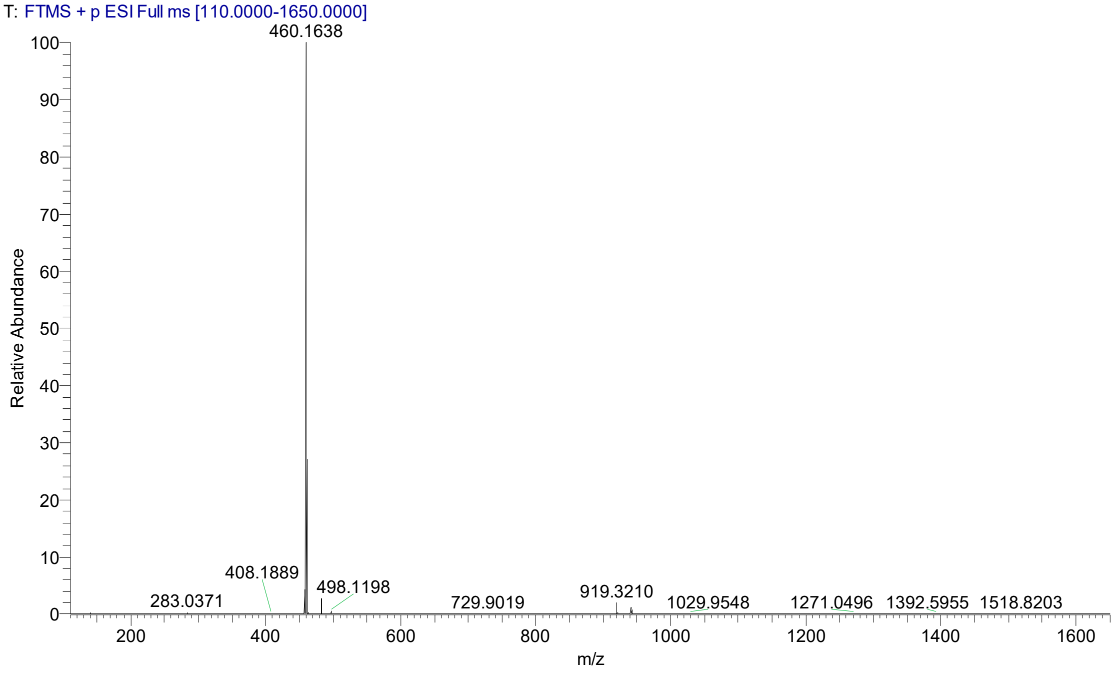
T: FTMS + p ESI Full ms [110.0000-1650.0000]
460.1638
408.1889
283.0371
498.1198
729.9019
919.3210
1029.9548
1271.0496
1392.5955
1518.8203
Relative Abundance
100
90
80
70
60
50
40
30
20
10
0
200
400
600
800
1000
1200
1400
1600
m/z

Figure S17. FTIR and HRMS spectra, 1H NMR at 400 MHz and 13C NMR at 100 MHz spectra for compound **58** ($CDCl_3$)

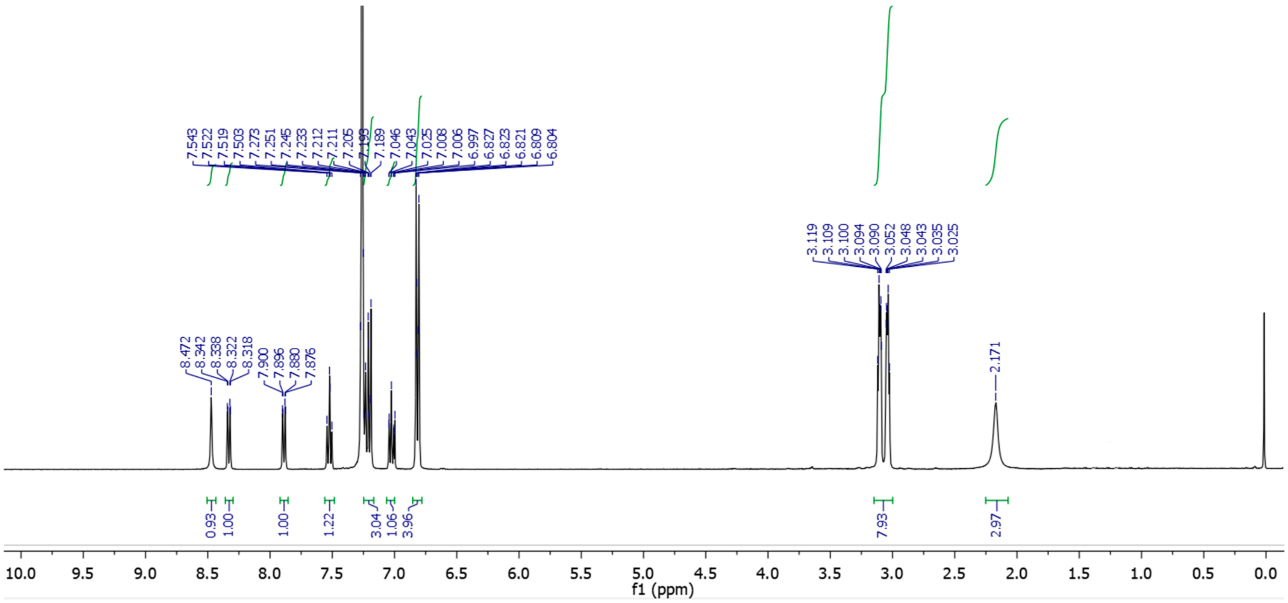

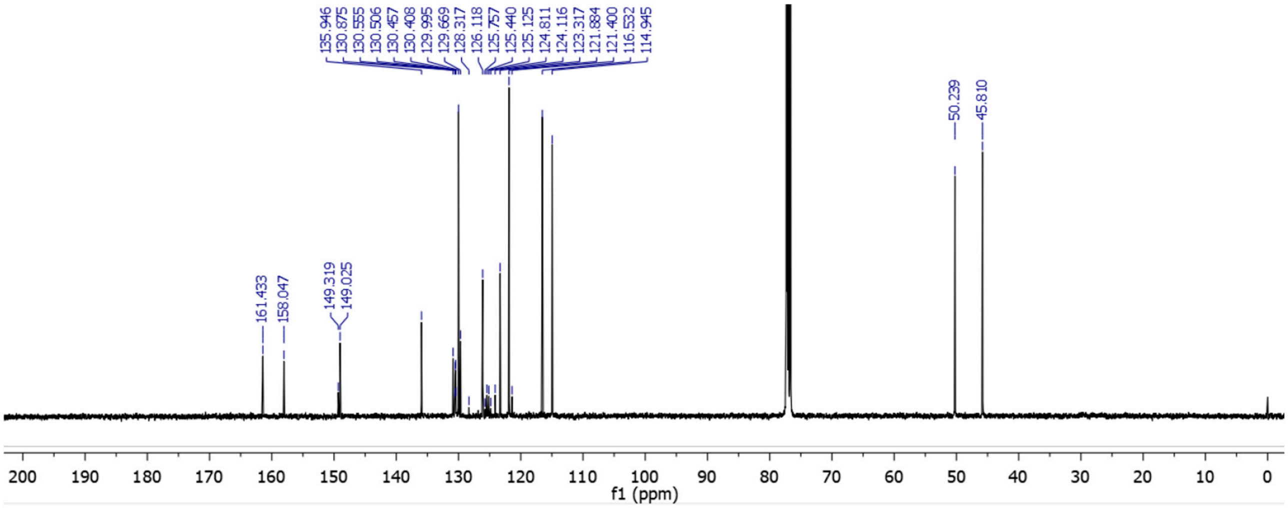

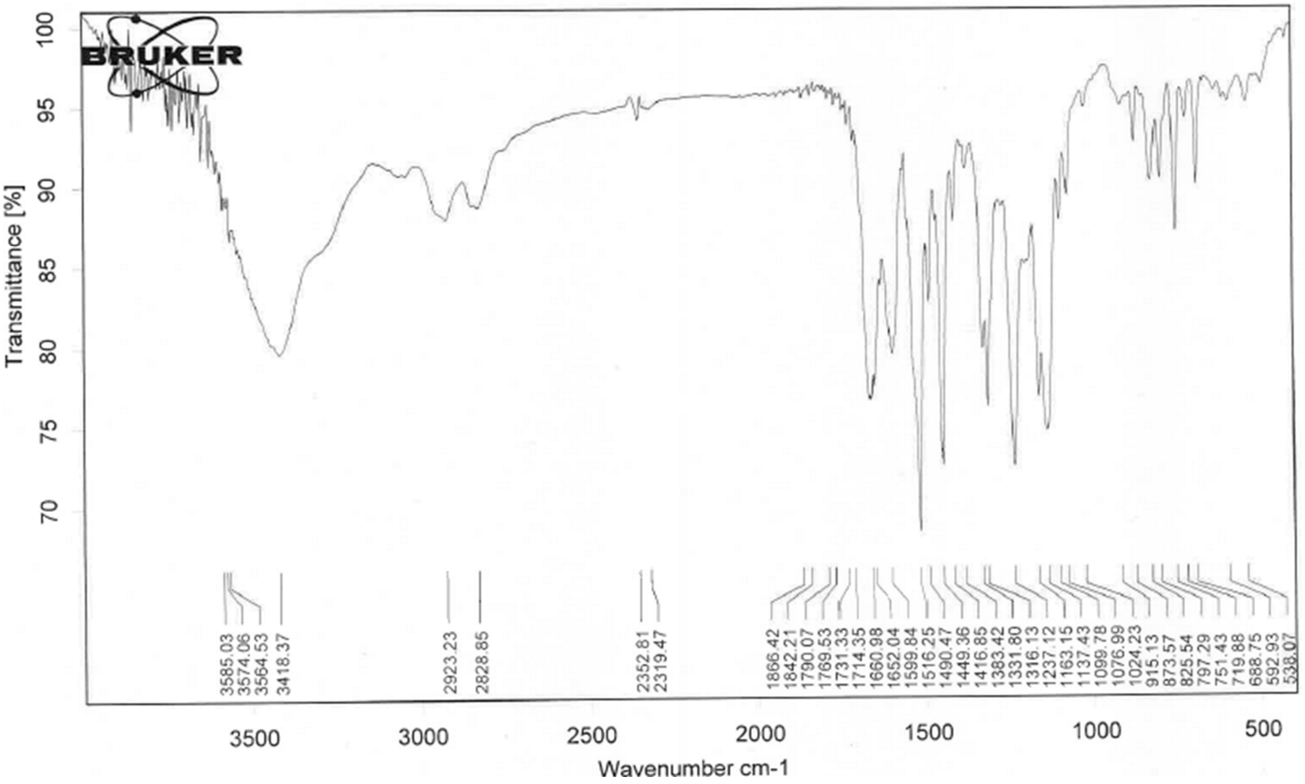
BRUKER
Transmittance [%]
100
95
90
85
80
75
70
3585.03
3574.06
3564.53
3418.37
2923.23
2828.85
2352.81
2319.47
1866.42
1842.21
1790.07
1769.53
1731.33
1714.35
1660.98
1652.04
1599.84
1516.25
1490.47
1449.36
1416.85
1383.42
1331.80
1316.13
1237.12
1163.15
1137.43
1099.78
1076.99
1024.23
915.13
873.57
825.54
797.29
751.43
719.88
688.75
592.93
538.07
3500
3000
2500
2000
1500
1000
500
Wavenumber cm-1

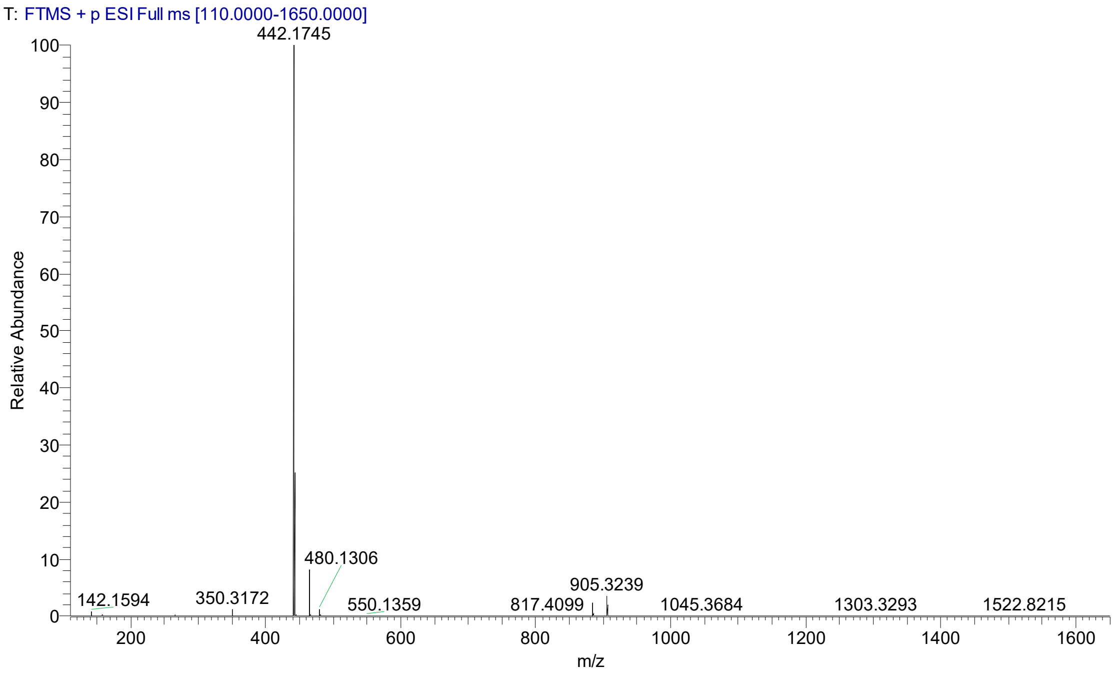
T: FTMS + p ESI Full ms [110.0000-1650.0000]
442.1745
Relative Abundance
480.1306
905.3239
142.1594
350.3172
550.1359
817.4099
1045.3684
1303.3293
1522.8215
m/z

Figure S18. FTIR and HRMS spectra, 1H NMR at 400 MHz and 13C NMR at 100 MHz spectra for compound **59** ($CDCl_3$)

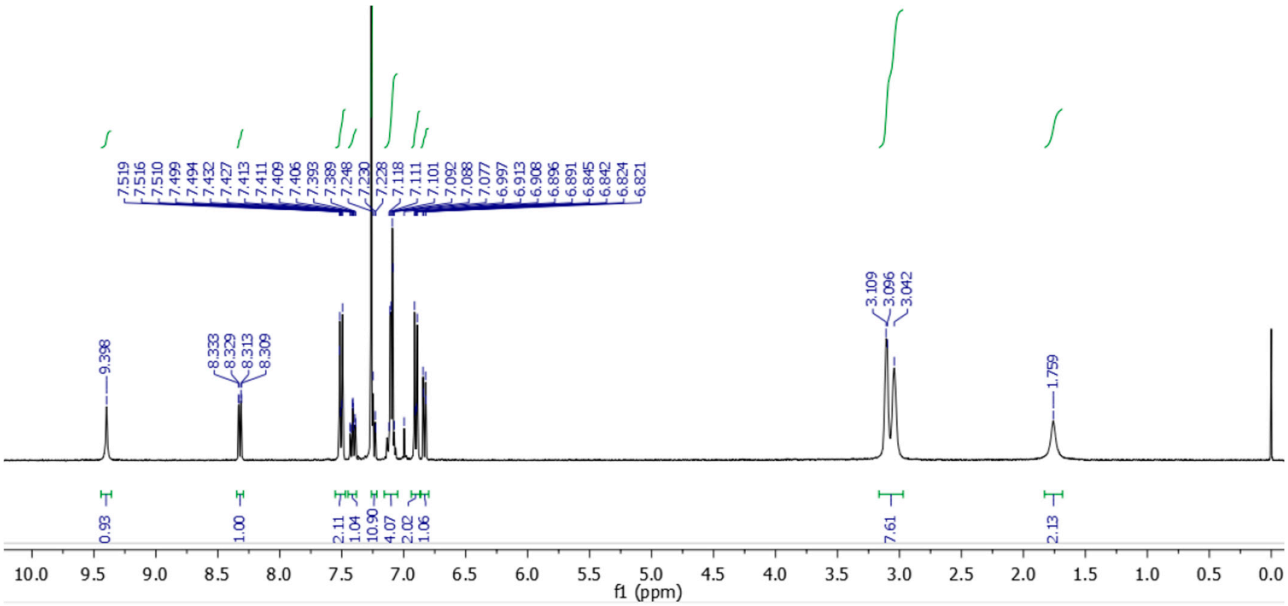

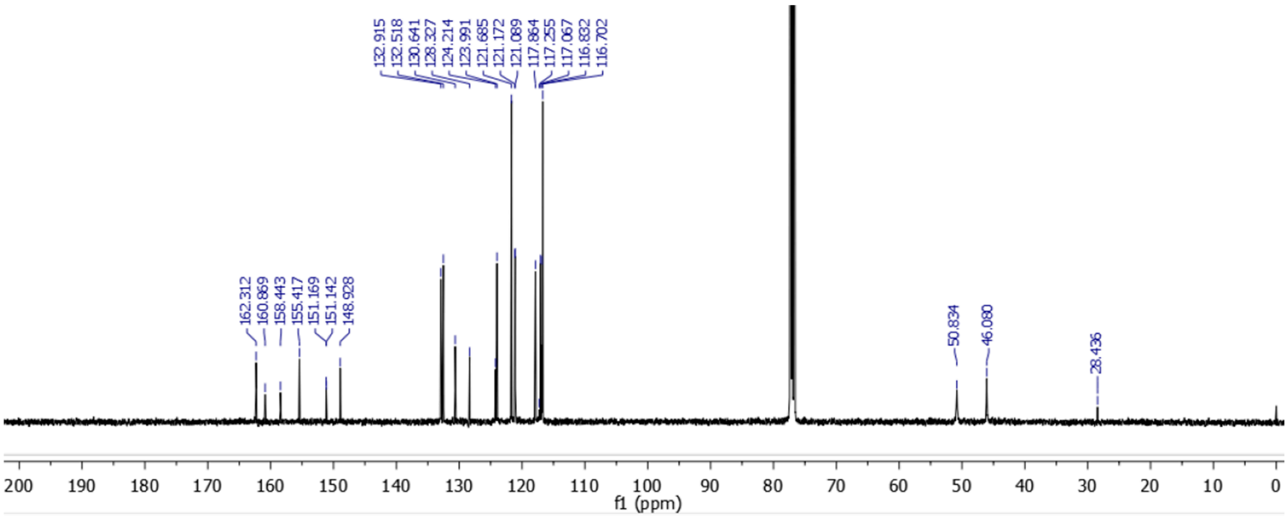

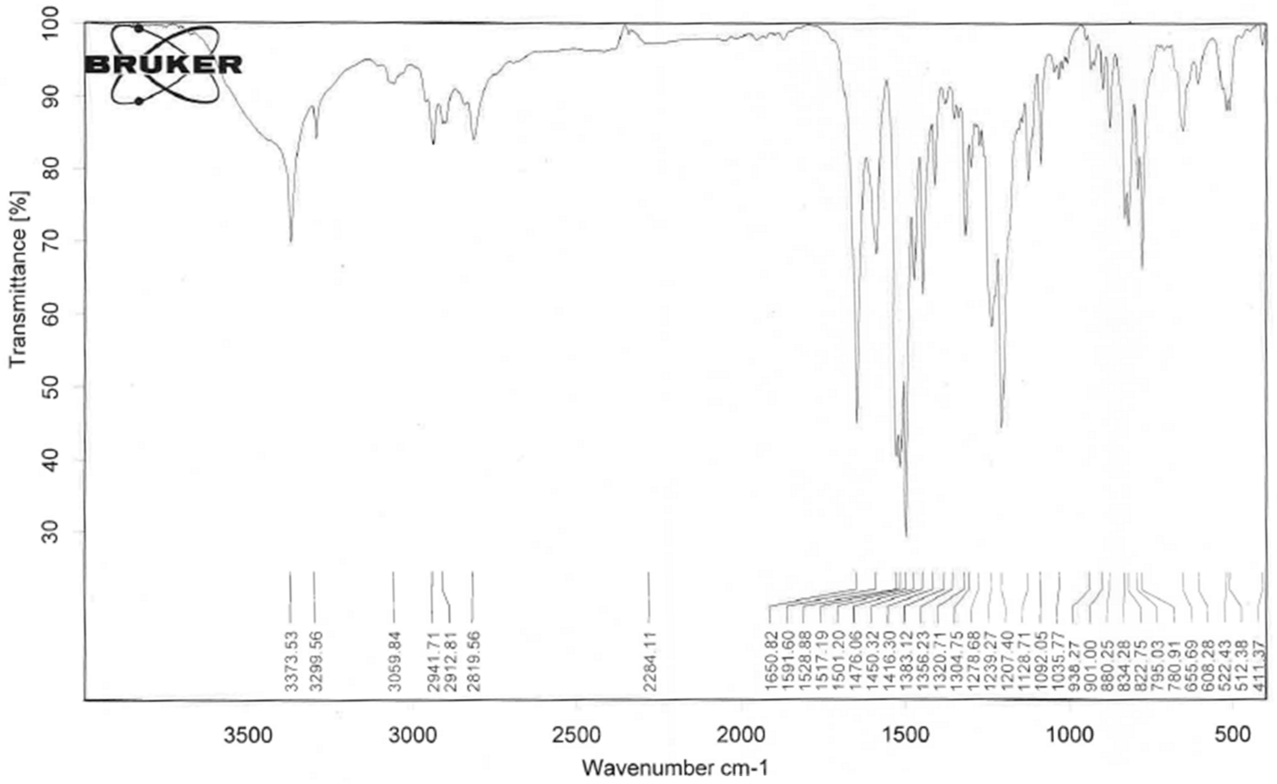

BRUKER
Transmittance [%]
Wavenumber cm-1
3373.53
3299.56
3059.84
2941.71
2912.81
2819.56
2284.11
1650.82
1591.60
1528.88
1517.19
1501.20
1476.06
1450.32
1416.30
1383.12
1356.23
1320.71
1304.75
1278.68
1239.27
1207.40
1128.71
1092.05
1035.77
938.27
901.00
880.25
834.28
822.75
795.03
780.91
655.69
608.28
522.43
512.38
411.37

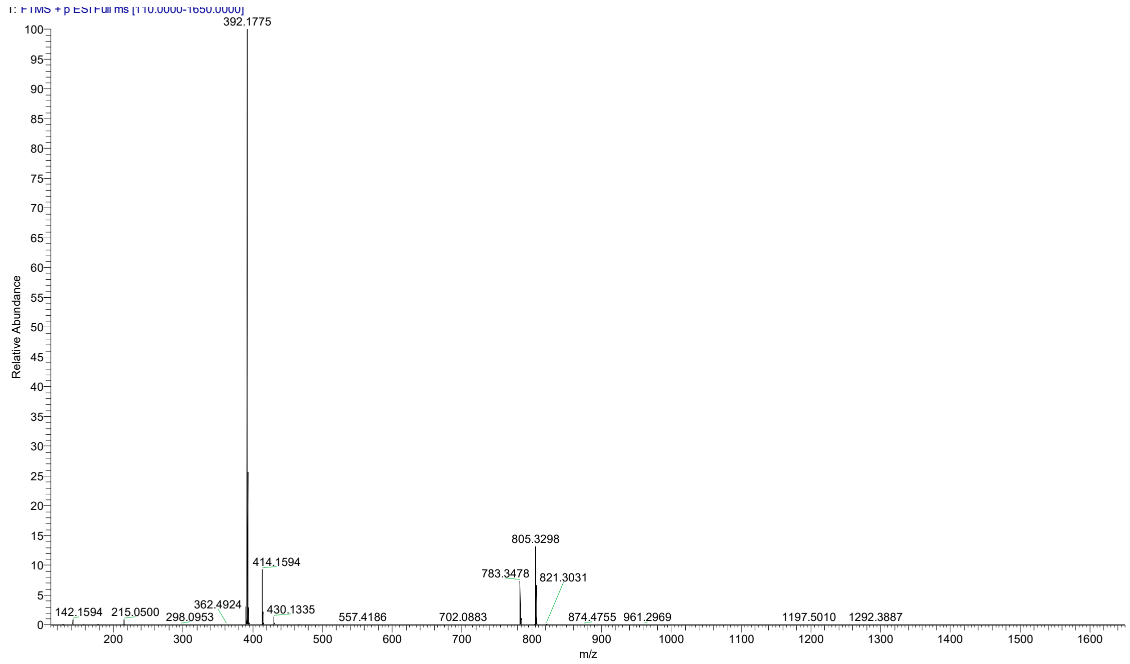

T: FTMS + p ESI Full ms [110.0000-1650.0000]
Relative Abundance
m/z
392.1775
805.3298
414.1594
783.3478
821.3031
362.4924
430.1335
142.1594
215.0500
298.0953
557.4186
702.0883
874.4755
961.2969
1197.5010
1292.3887

Figure S19. FTIR and HRMS spectra, 1H NMR at 400 MHz and 13C NMR at 100 MHz spectra for compound **60** ($CDCl_3$)

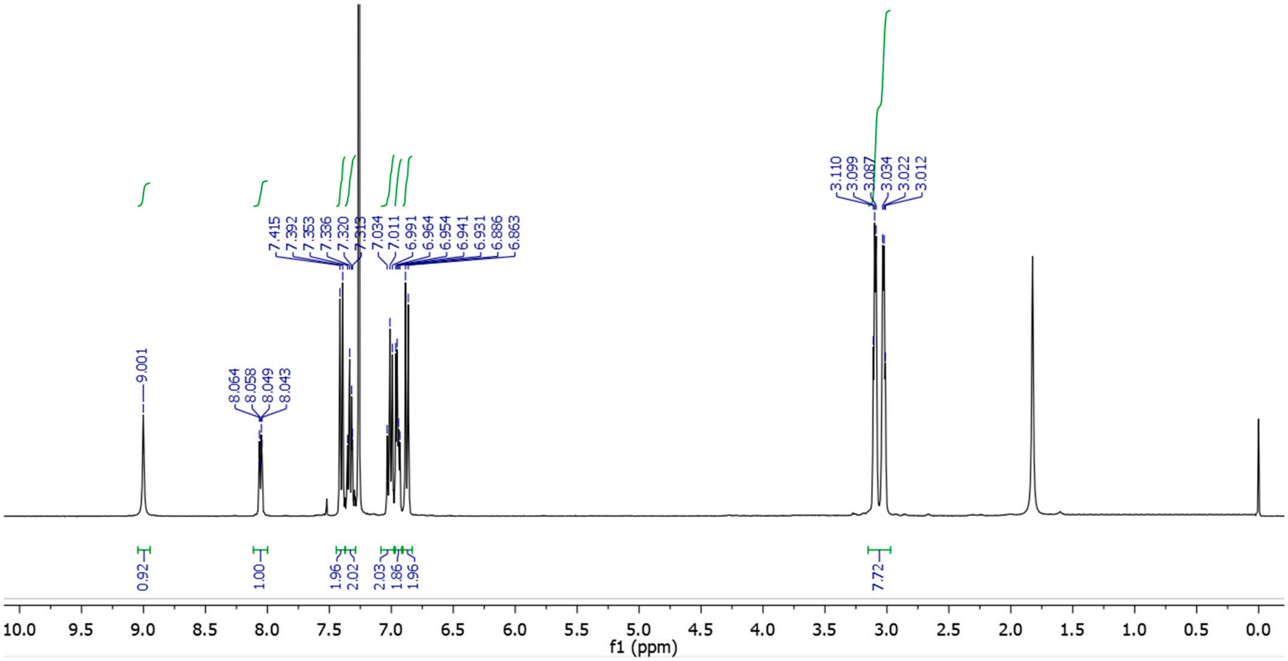

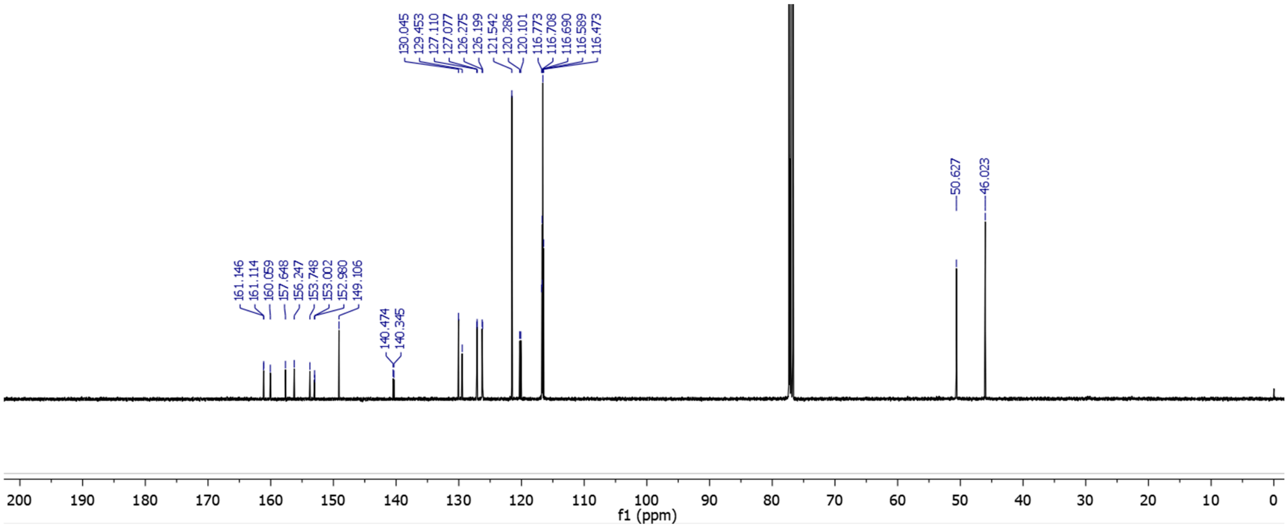

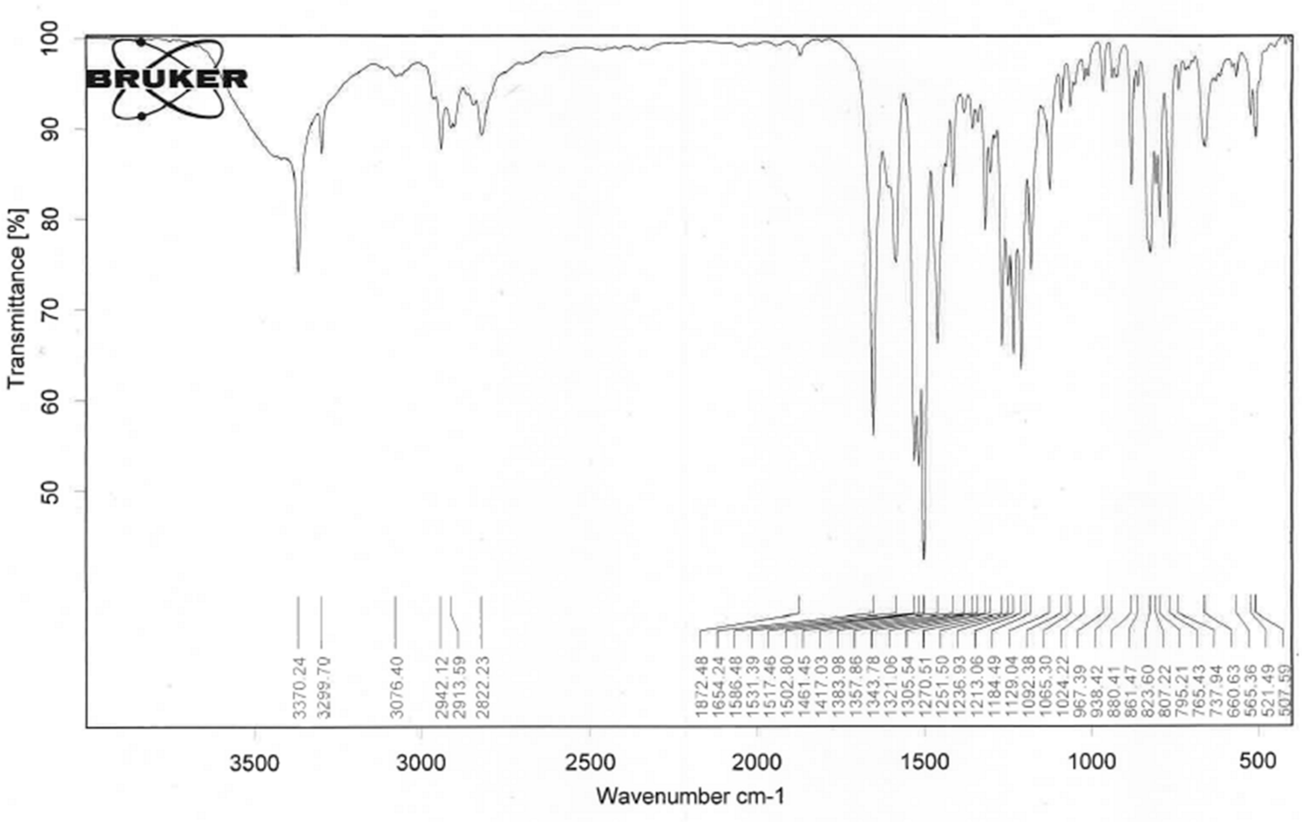
BRUKER
Transmittance [%]
Wavenumber cm-1
3370.24
3299.70
3076.40
2942.12
2913.59
2822.23
1872.48
1654.24
1586.48
1531.39
1517.46
1502.80
1461.45
1417.03
1383.98
1357.86
1343.78
1321.06
1305.54
1270.51
1251.50
1236.93
1213.06
1184.49
1129.04
1092.38
1065.30
1024.22
967.39
938.42
880.41
861.47
823.60
807.22
795.21
765.43
737.94
660.63
565.36
521.49
507.59

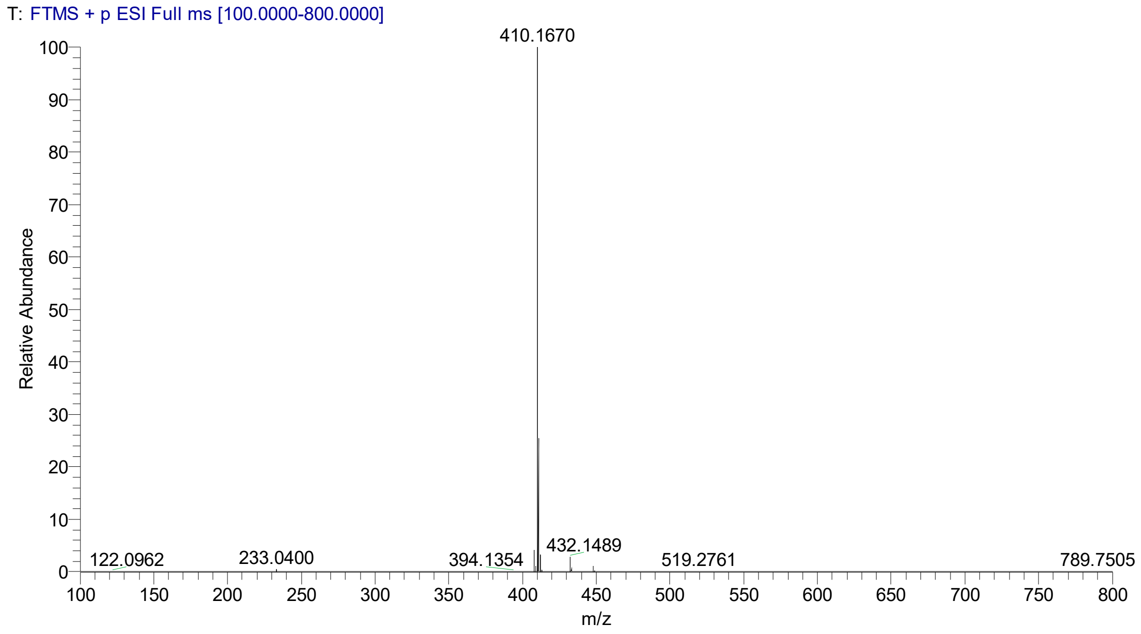
T: FTMS + p ESI Full ms [100.0000-800.0000]
410.1670
122.0962
233.0400
394.1354
432.1489
519.2761
789.7505
Relative Abundance
m/z

Figure S20. FTIR and HRMS spectra, 1H NMR at 400 MHz and 13C NMR at 100 MHz spectra for compound **61** (DMSO-d6)

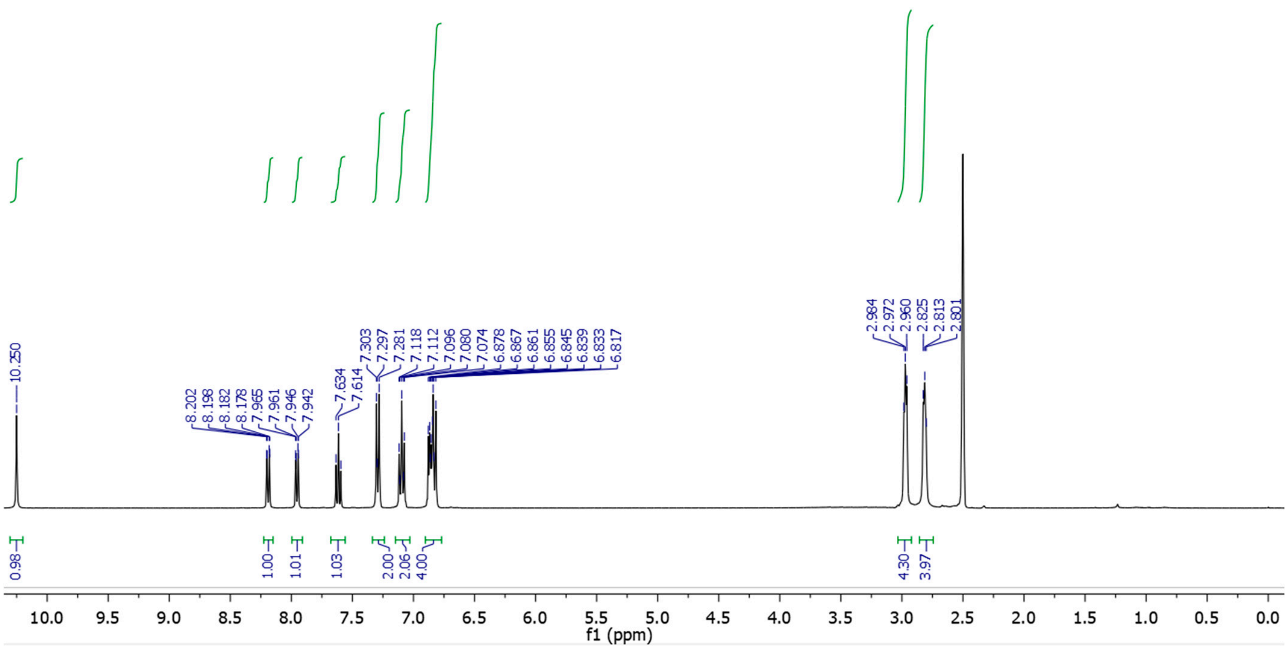

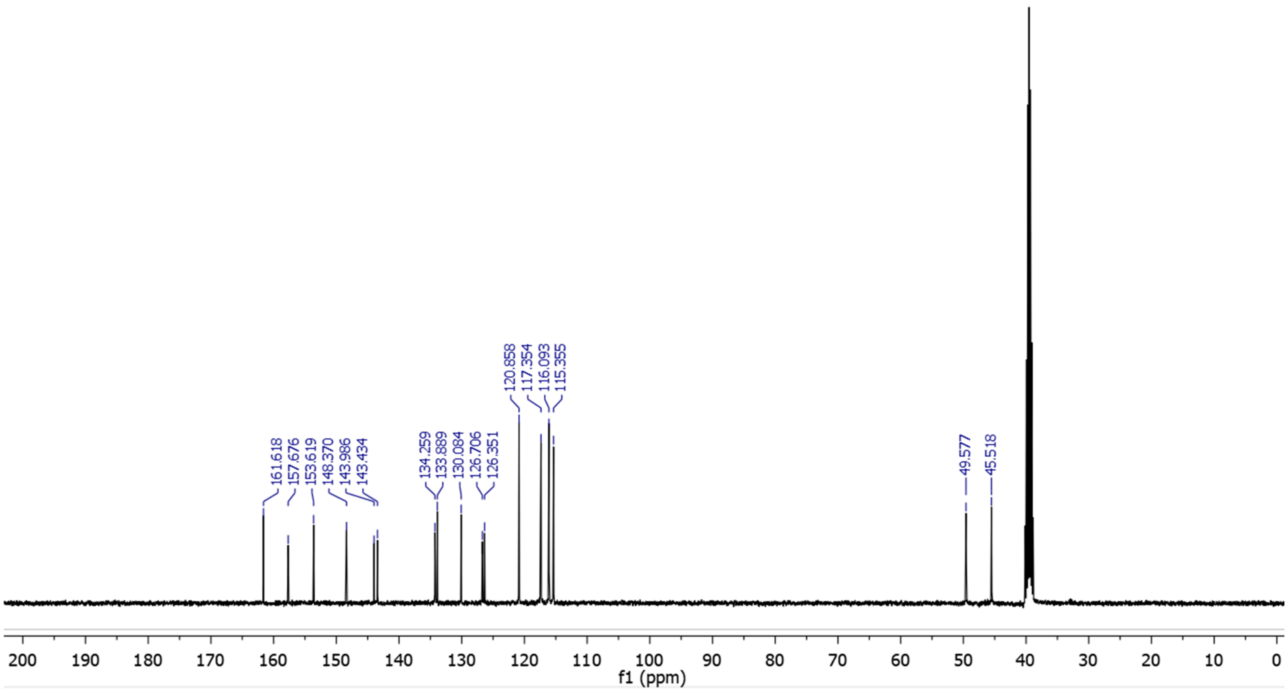

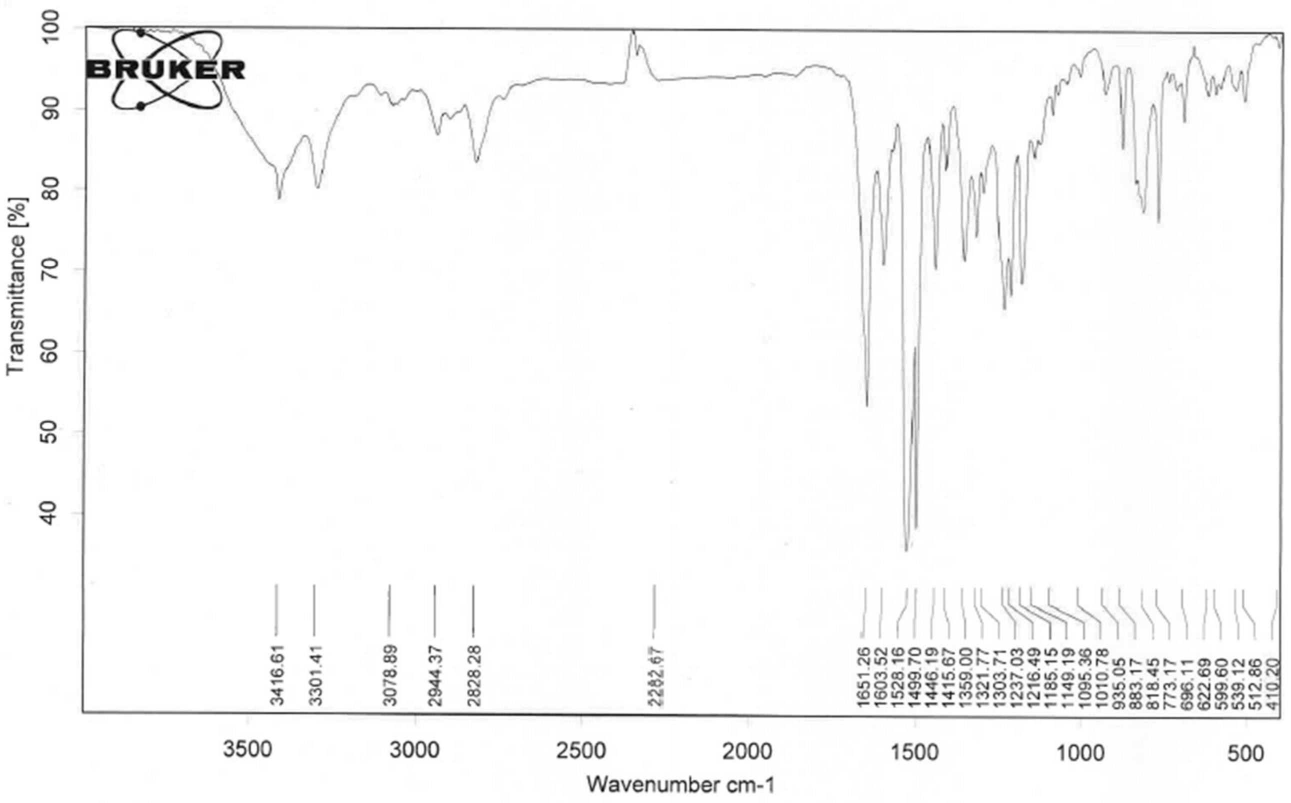
BRUKER
Transmittance [%]
100
90
80
70
60
50
40
3416.61
3301.41
3078.89
2944.37
2828.28
2282.67
1651.26
1603.52
1528.16
1499.70
1446.19
1415.67
1359.00
1321.77
1303.71
1237.03
1216.49
1185.15
1149.19
1095.36
1010.78
935.05
883.17
818.45
773.17
696.11
622.69
599.60
539.12
512.86
410.20
3500
3000
2500
2000
1500
1000
500
Wavenumber cm-1

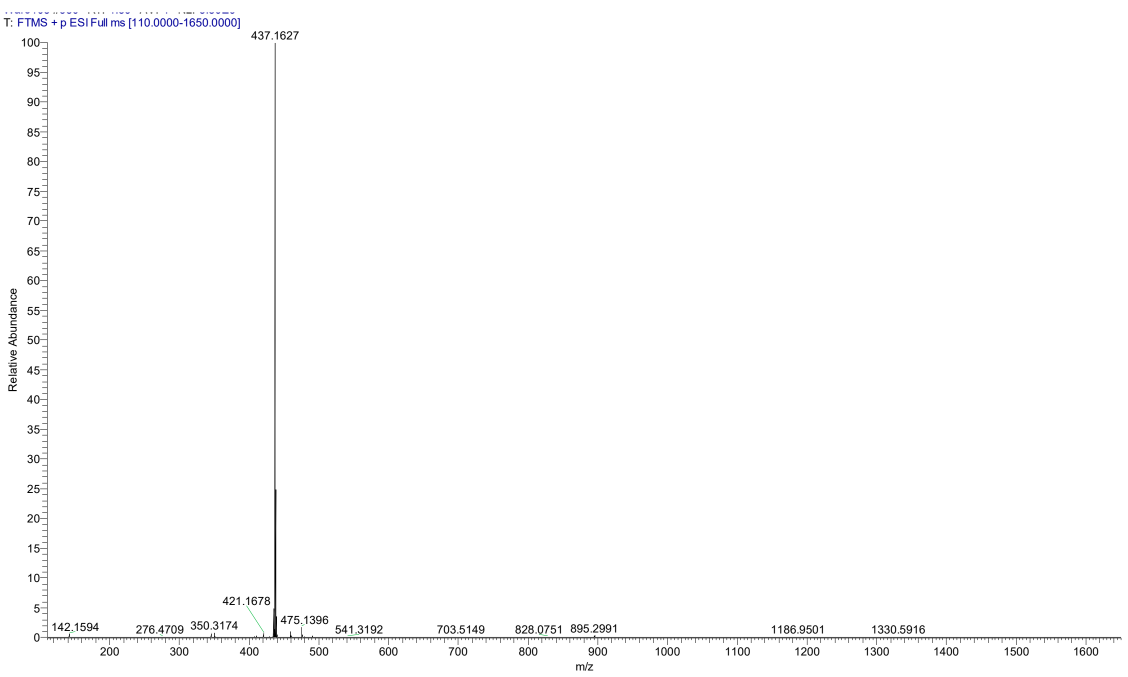
T: FTMS + p ESI Full ms [110.0000-1650.0000]
437.1627
Relative Abundance
421.1678
475.1396
142.1594
276.4709
350.3174
541.3192
703.5149
828.0751
895.2991
1186.9501
1330.5916
m/z

Figure S21. FTIR and HRMS spectra, 1H NMR at 400 MHz and 13C NMR at 100 MHz spectra for compound **62** ($CDCl_3$)

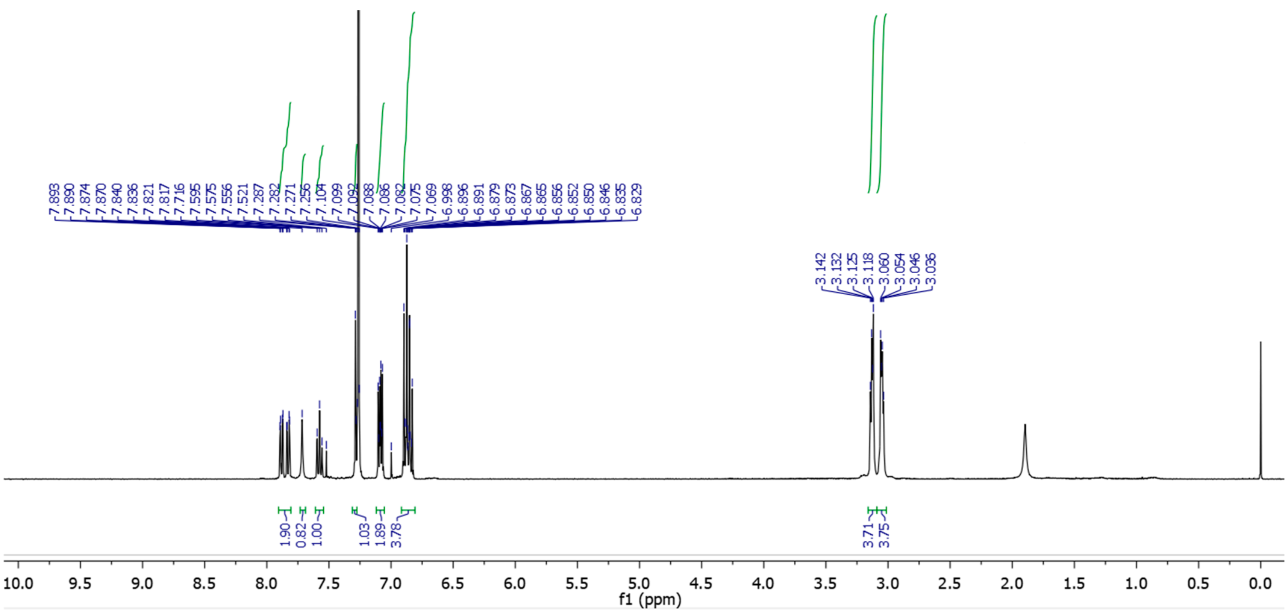

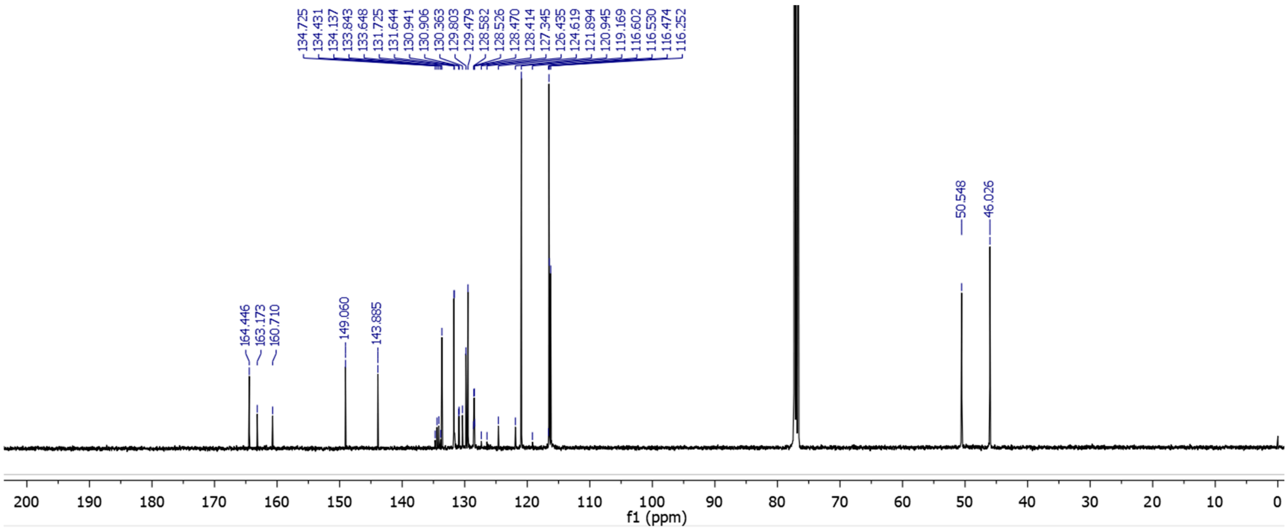

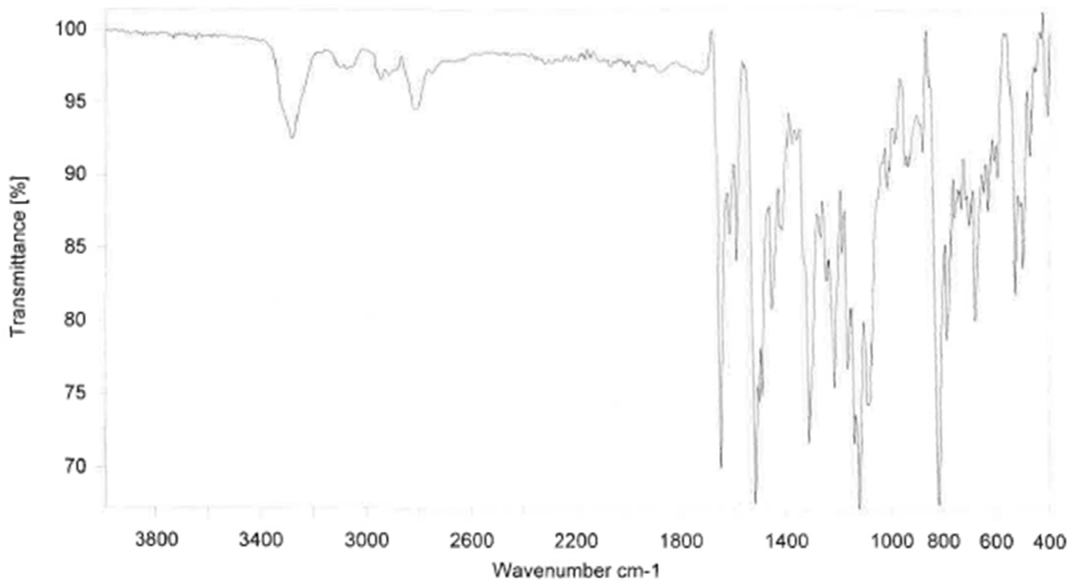

Wavelength	Absolute Intensity	Relative Intensity	Width	if Line < Shoulder	
3281.7683	0.925	0.075	101.8851	21.978777	0
3077.5172	0.973	0.013	81.0634	3.824243	0
2949.4362	0.966	0.019	86.2112	4.652929	0
2814.1617	0.945	0.044	68.0161	12.182627	0
2322.9821	0.977	0.009	99.9861	2.343201	0
1976.9615	0.971	0.010	13.8214	2.579468	0
1875.9364	0.971	0.008	47.2248	2.324031	0
1717.0455	0.970	0.029	933.9489	5.061743	0
1646.4987	0.700	0.294	20.6485	81.593781	0
1615.5451	0.859	0.037	10.7321	8.696502	0
1588.6403	0.841	0.087	11.9953	19.803675	0
1517.1217	0.676	0.292	50.9264	78.289009	0
1501.0660	0.745	0.019	31.1116	3.427743	0
1489.1298	0.749	0.043	6.0040	10.083630	0
1453.3557	0.809	0.082	15.9361	20.743944	0
1413.9697	0.863	0.051	107.0250	8.535467	0
1378.6903	0.922	0.018	1013.6092	3.044170	0
1309.8915	0.718	0.204	28.8383	50.731705	0
1269.2611	0.858	0.022	76.0922	4.709969	0
1244.8404	0.828	0.027	1095.4332	4.538485	0
1217.5445	0.755	0.134	27.5504	37.946453	0
1186.2889	0.847	0.036	8.4240	8.805311	0
1164.8833	0.768	0.065	1011.2477	12.636461	0
1142.2767	0.717	0.026	8.8530	5.796220	0
1122.2531	0.672	0.334	107.1177	95.944305	0
1088.6348	0.743	0.070	23.1172	16.968037	0
1015.1260	0.892	0.032	10.8624	8.231207	0
988.7246	0.922	0.022	91.5810	3.203931	0
944.9611	0.907	0.019	739.1836	2.429859	0
933.7005	0.906	0.072	39.5228	17.922680	0
878.9064	0.916	0.063	54.3861	7.766436	0
817.6384	0.675	0.327	36.9986	95.111725	0
784.4414	0.788	0.070	13.3198	19.408484	0
758.5799	0.872	0.030	11.9530	7.962374	0
733.8025	0.877	0.027	127.8350	4.185768	0
702.6255	0.866	0.033	14.0554	7.241991	0
679.7692	0.801	0.128	17.4636	32.894600	0
648.4646	0.889	0.016	6.8978	3.948472	0
632.4305	0.876	0.044	11.2440	9.710068	0
612.2285	0.911	0.014	6.7256	3.295664	0
597.6915	0.900	0.061	60.7966	7.891520	0
531.5248	0.820	0.184	48.8142	52.700611	0

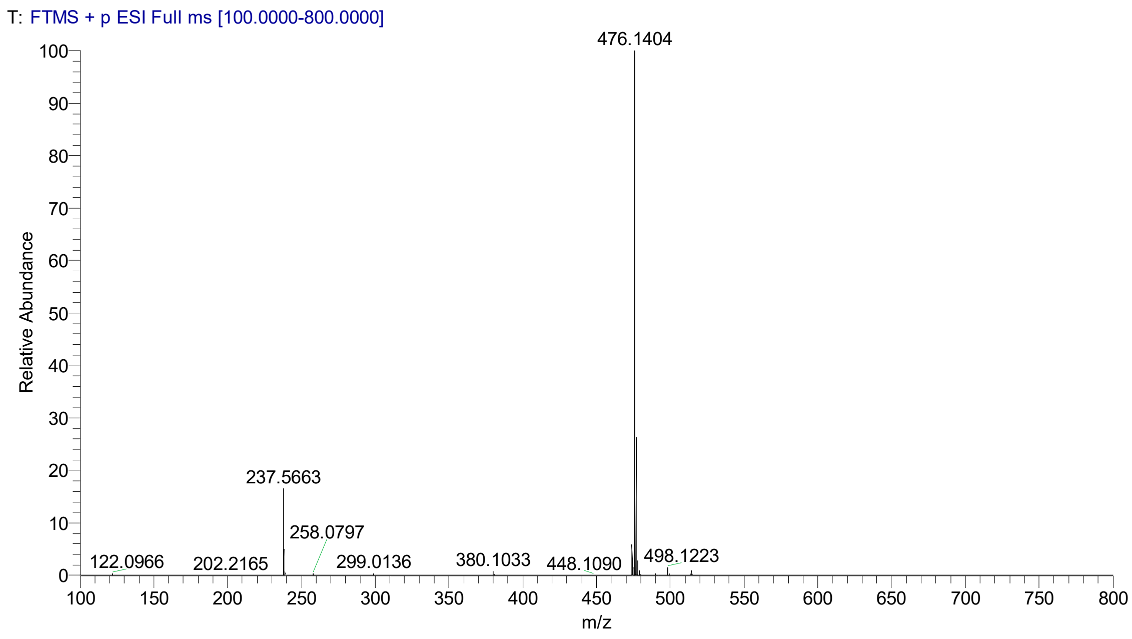
T: FTMS + p ESI Full ms [100.0000-800.0000]
476.1404
237.5663
258.0797
122.0966
202.2165
299.0136
380.1033
448.1090
498.1223
Relative Abundance
m/z

Figure S22. FTIR and HRMS spectra, 1H NMR at 400 MHz and 13C NMR at 100 MHz spectra for compound **63** (CD_3OD)

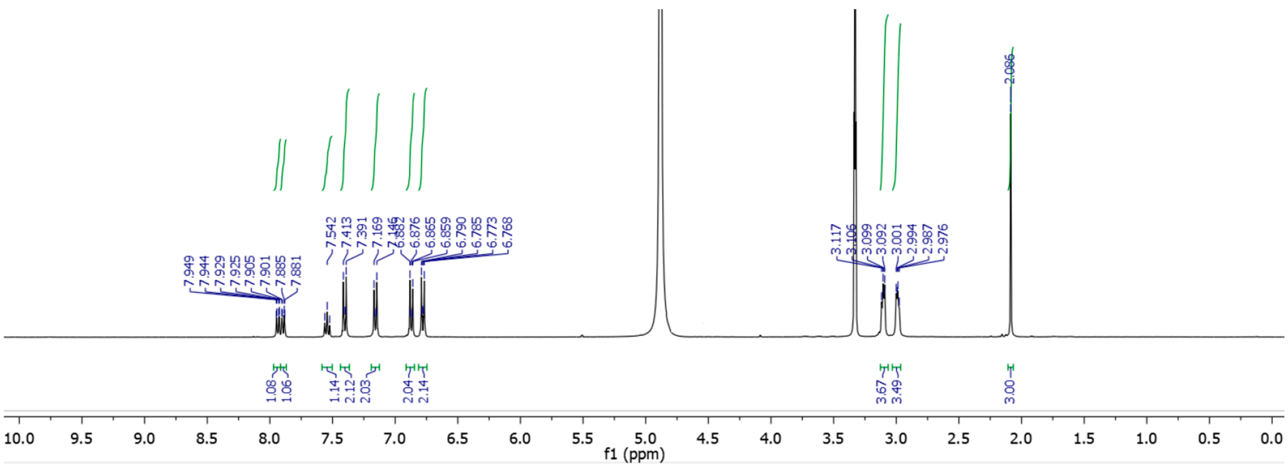

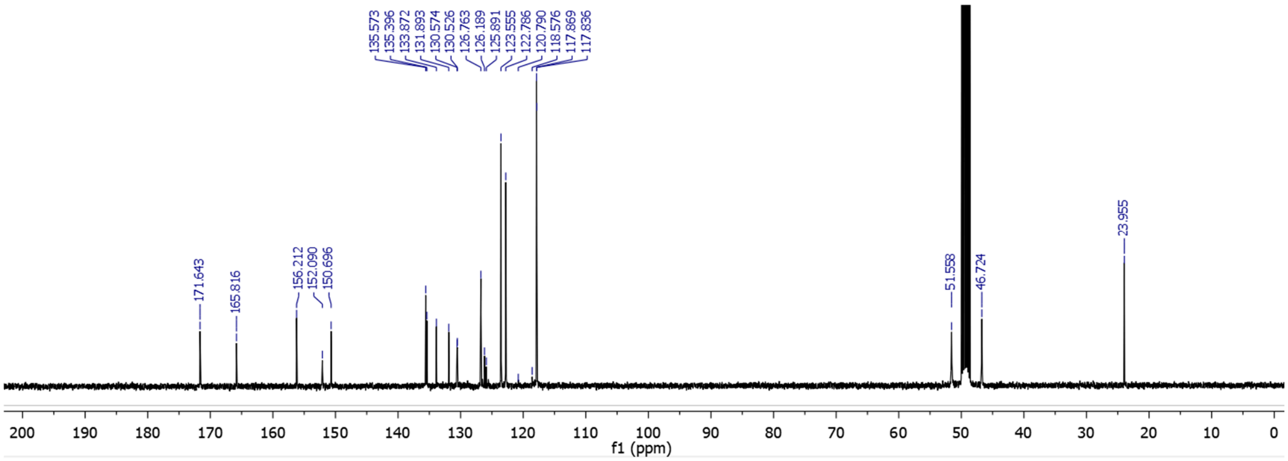

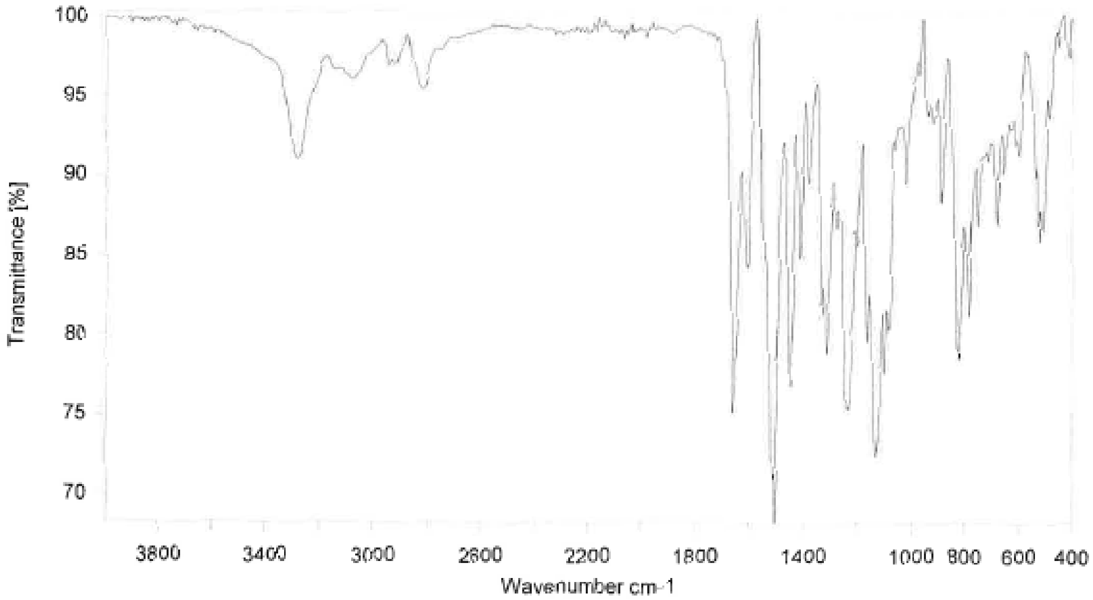

Wavelength	Absolute Intensity	Relative Intensity	Width	ii Line	< Shoulder
3283.1262	0.911	0.089	109.7286	27.830801	0
3076.8521	0.961	0.019	121.8876	4.377832	0
2945.1011	0.970	0.017	48.5787	4.872376	0
2816.5137	0.955	0.035	65.1770	10.709121	0
2293.9602	0.989	0.009	160.5845	2.242952	0
2062.3680	0.988	0.010	31.1750	2.930982	0
1978.4494	0.988	0.010	9.1176	3.180558	0
1883.1156	0.990	0.007	60.7506	2.001702	0
1650.2302	0.752	0.248	36.3010	77.238091	0
1604.5257	0.843	0.097	73.0976	18.745792	0
1503.9134	0.682	0.320	45.2050	99.045113	0
1445.4115	0.767	0.162	19.1106	48.368584	0
1406.0462	0.850	0.086	16.8389	24.308987	0
1373.3084	0.895	0.058	18.9191	16.373861	0
1328.9746	0.814	0.046	211.8378	5.405037	0
1313.0079	0.788	0.135	40.6666	33.923756	0
1274.7522	0.869	0.019	521.6699	3.452604	0
1231.5142	0.755	0.178	45.3602	52.065670	0
1197.1156	0.857	0.025	63.3560	3.233154	0
1161.0296	0.796	0.057	218.5120	9.764098	0
1126.1342	0.725	0.246	98.3207	73.287987	0
1096.3447	0.776	0.041	7.5839	12.053605	0
1082.0705	0.804	0.021	151.2001	3.924213	0
1012.4469	0.895	0.054	113.1926	11.731009	0
962.8897	0.965	0.019	30.2351	2.910037	0
935.4702	0.939	0.027	197.6942	2.371448	0
916.7386	0.934	0.036	319.0167	4.822699	0
881.3598	0.884	0.097	15.9631	28.423620	0
821.6732	0.786	0.215	71.7156	66.876137	0

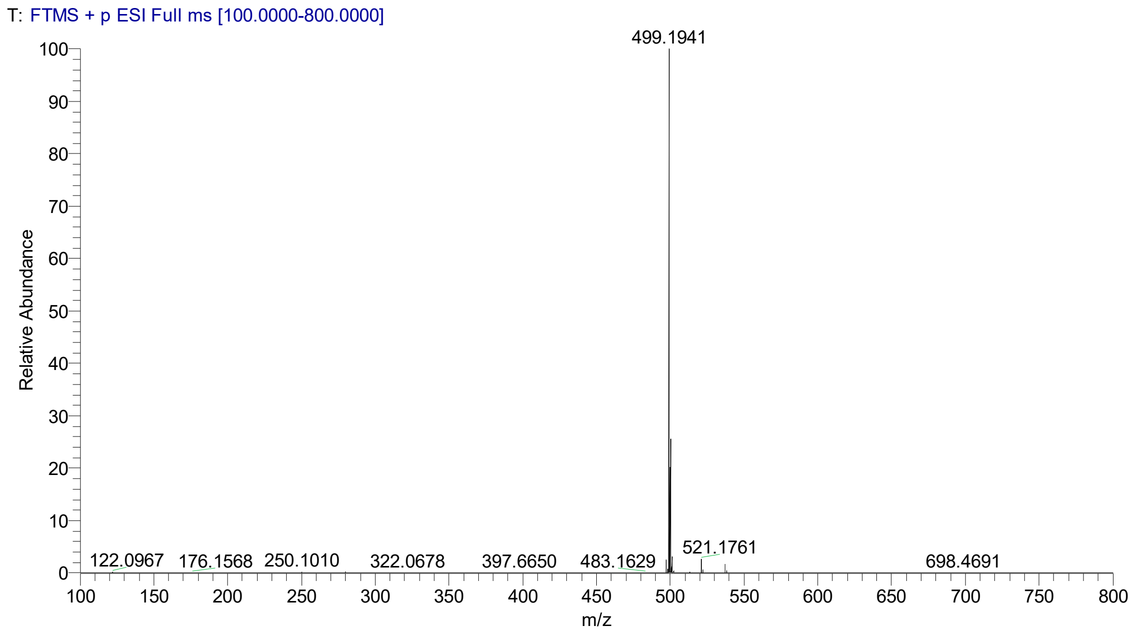
T: FTMS + p ESI Full ms [100.0000-800.0000]
499.1941
Relative Abundance
122.0967
176.1568
250.1010
322.0678
397.6650
483.1629
521.1761
698.4691
m/z

Figure S23. FTIR and HRMS spectra, 1H NMR at 400 MHz and 13C NMR at 100 MHz spectra for compound **64** ($CDCl_3$)

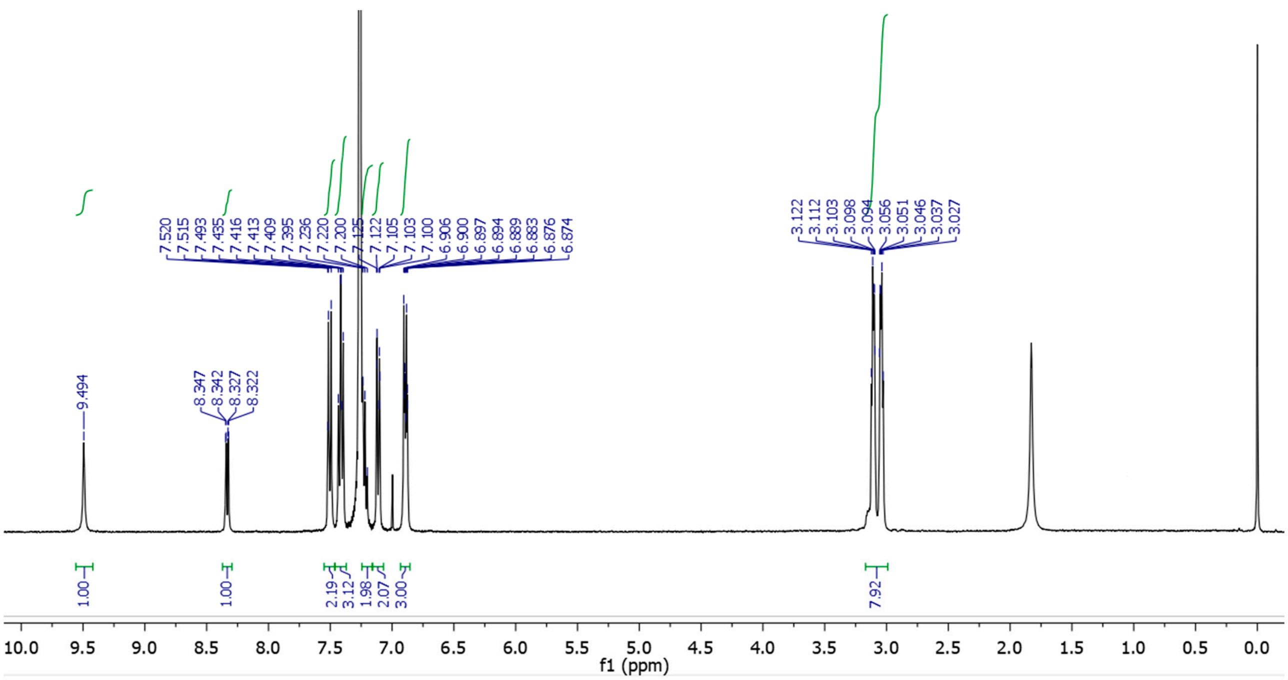

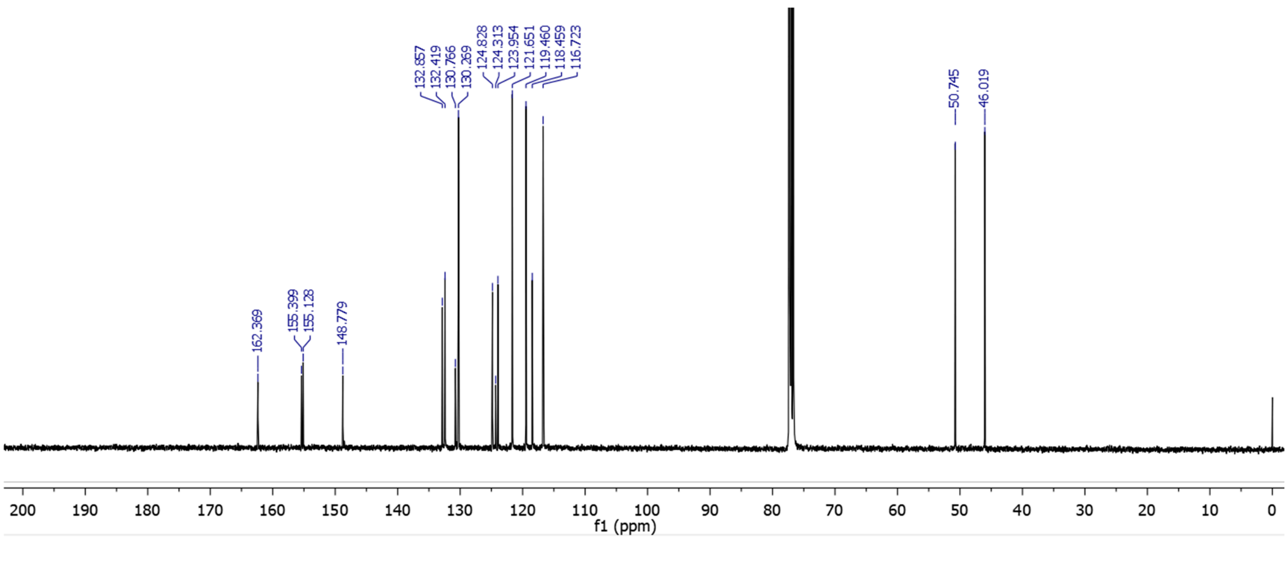

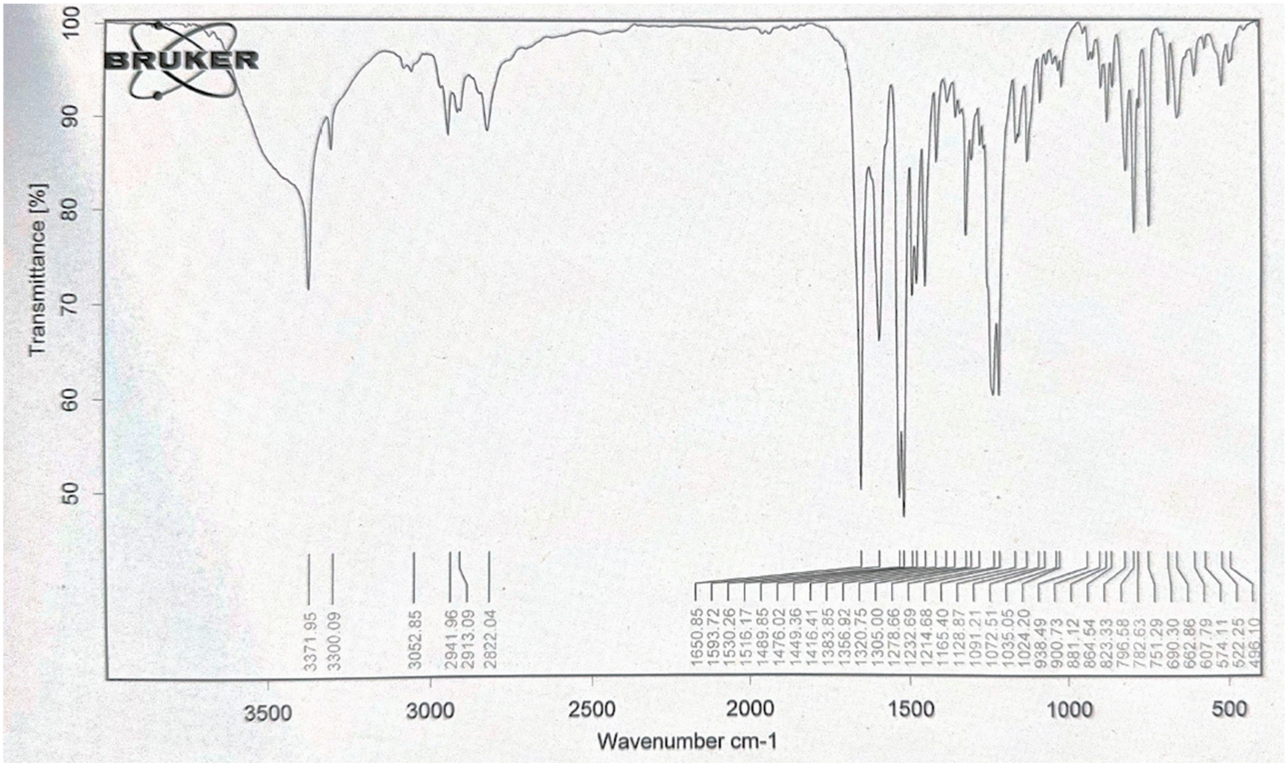

BRUKER
Transmittance [%]
100
90
80
70
60
50
3371.95
3300.09
3052.85
2941.96
2913.09
2822.04
1650.85
1593.72
1530.26
1516.17
1489.85
1476.02
1449.36
1416.41
1383.85
1356.92
1320.75
1305.00
1278.66
1232.69
1214.68
1165.40
1128.87
1091.21
1072.51
1035.05
1024.20
938.49
900.73
881.12
864.54
823.33
796.58
782.63
751.29
690.30
662.86
607.79
574.11
522.25
496.10
3500
3000
2500
2000
1500
1000
500
Wavenumber cm-1

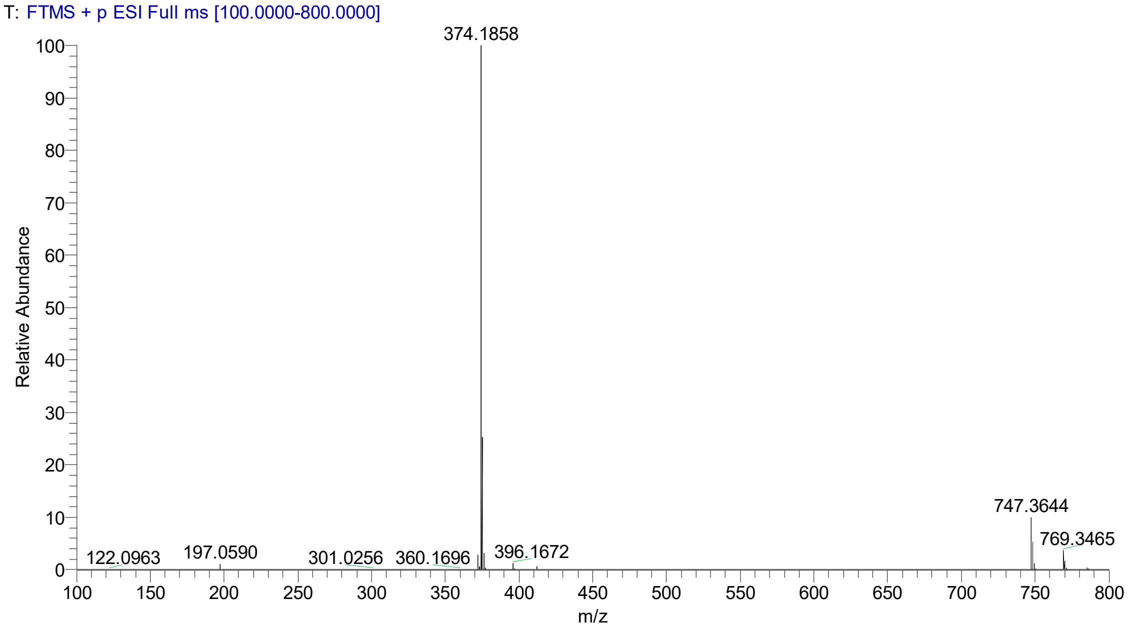

T: FTMS + p ESI Full ms [100.0000-800.0000]
374.1858
Relative Abundance
100
90
80
70
60
50
40
30
20
10
0
122.0963
197.0590
301.0256
360.1696
396.1672
747.3644
769.3465
100
150
200
250
300
350
400
450
500
550
600
650
700
750
800
m/z

Figure S24. FTIR and HRMS spectra, 1H NMR at 400 MHz and 13C NMR at 100 MHz spectra for compound **65** ($CDCl_3$)

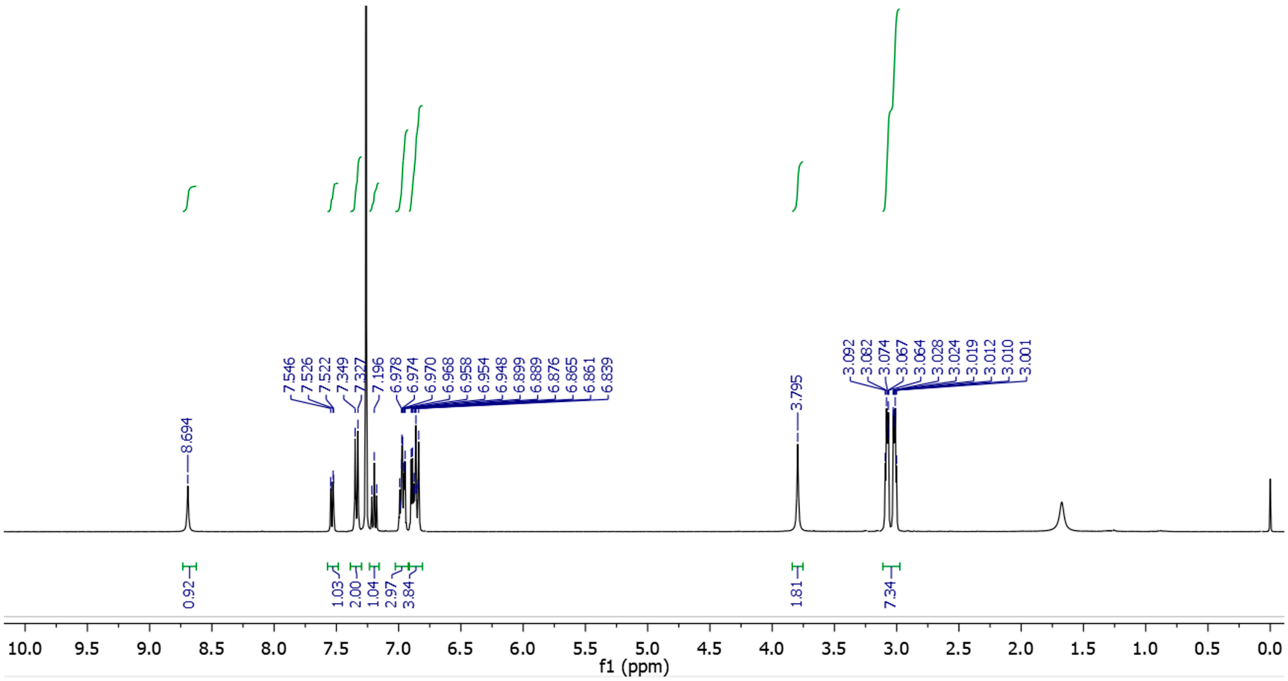

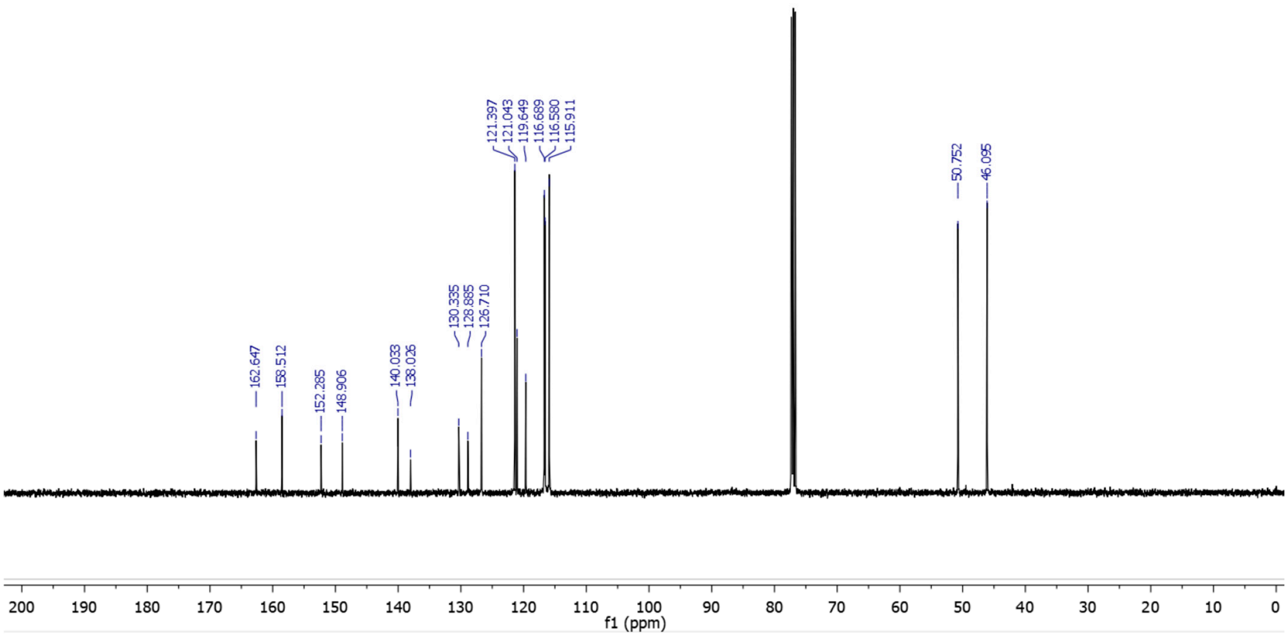

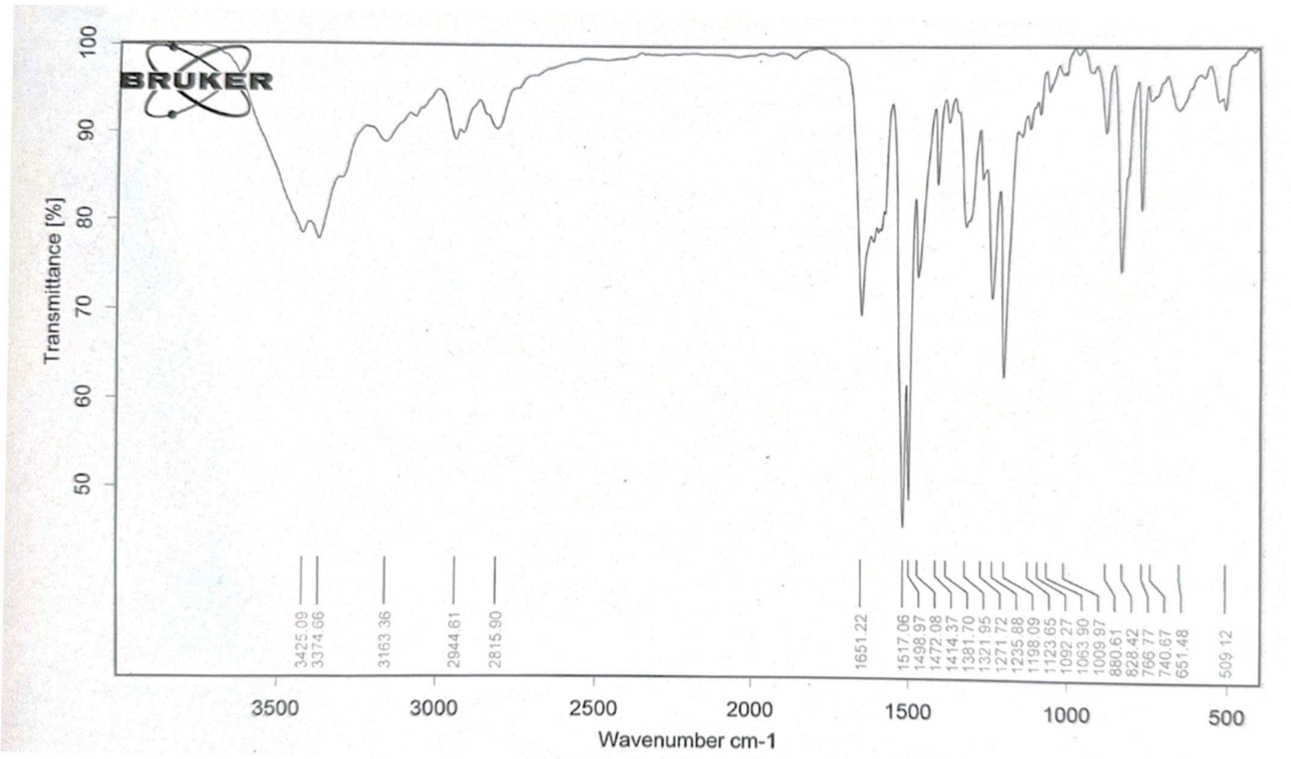

BRUKER
Transmittance [%]
Wavenumber cm-1
3425.09
3374.66
3163.36
2944.61
2815.90
1651.22
1517.06
1498.97
1472.08
1414.37
1381.70
1321.95
1271.72
1235.88
1198.09
1123.65
1092.27
1063.90
1009.97
880.61
828.42
766.77
740.67
651.48
509.12

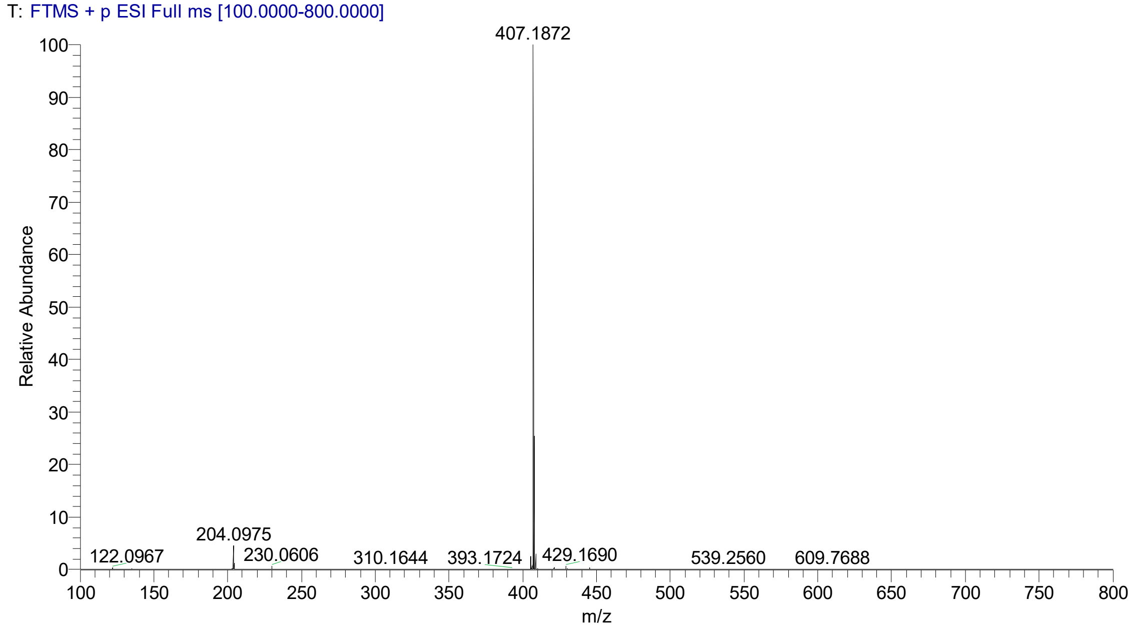

T: FTMS + p ESI Full ms [100.0000-800.0000]
Relative Abundance
m/z
407.1872
204.0975
122.0967
230.0606
310.1644
393.1724
429.1690
539.2560
609.7688

Figure S25. FTIR and HRMS spectra, 1H NMR at 400 MHz and 13C NMR at 100 MHz spectra for compound **66** (DMSO-d_6)

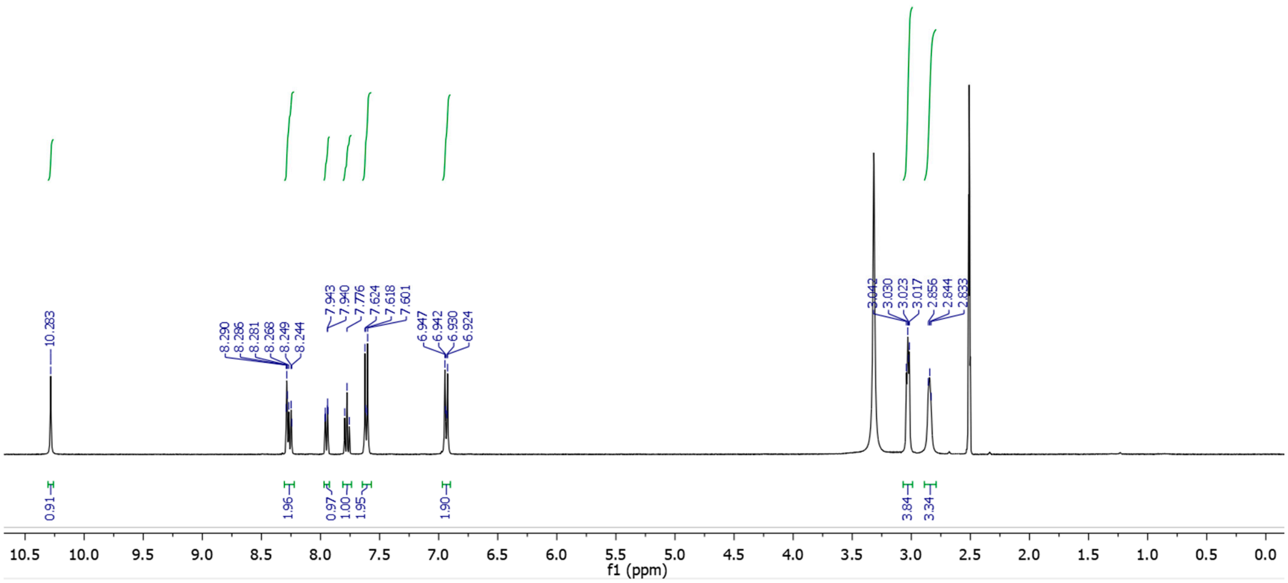

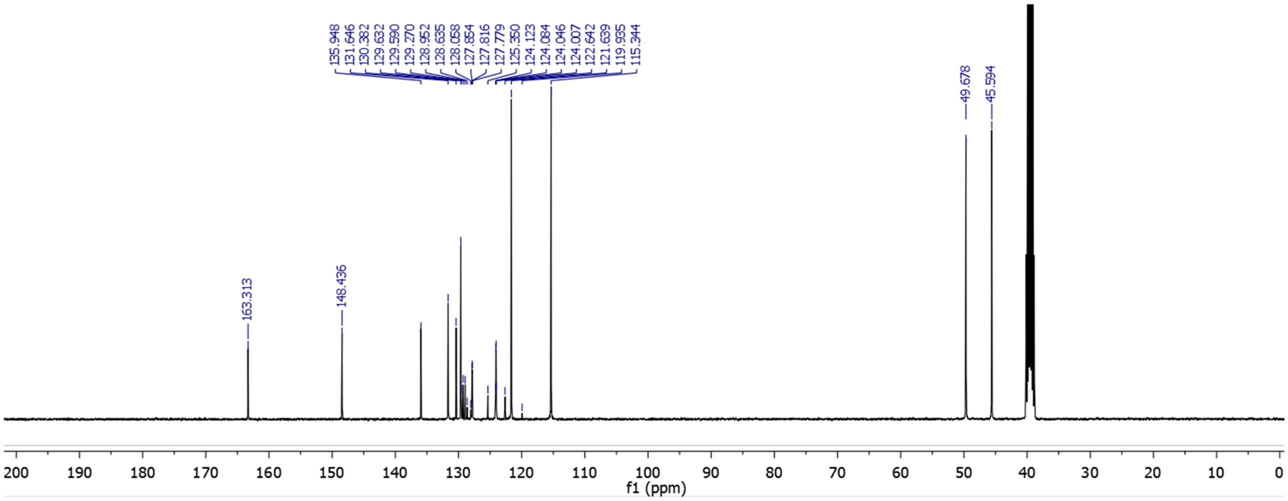

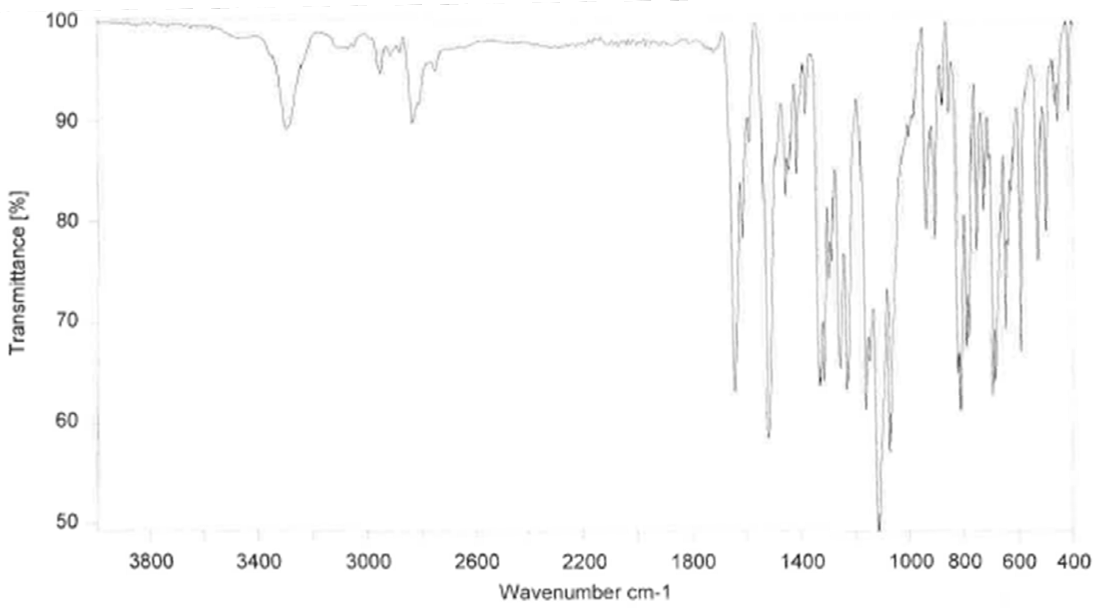

Wavelength	Absolute Intensity	Relative Intensity	Width	if Line < Shoulder	
3294.6878	0.893	0.099	69.6837	19.158466	0
3071.1391	0.973	0.016	87.4246	3.099871	0
2950.4608	0.949	0.038	25.8724	7.102750	0
2834.9426	0.898	0.092	51.3832	17.749454	0
1717.1602	0.970	0.018	70.2284	2.789280	0
1641.4829	0.632	0.368	25.9528	72.264755	0
1615.3232	0.783	0.080	60.8348	11.283943	0
1588.9661	0.879	0.047	78.3877	5.068496	0
1520.8345	0.586	0.385	29.8371	75.203178	0
1455.5140	0.826	0.101	22.5640	18.253551	0
1442.5136	0.852	0.029	76.1553	2.310123	0
1415.5428	0.847	0.096	8.9381	17.921637	0
1383.4721	0.907	0.055	9.1489	10.087735	0
1330.3116	0.637	0.286	34.5290	42.443695	0
1314.7962	0.642	0.084	27.3428	12.097741	0
1296.4352	0.744	0.081	8.4763	14.113645	0
1286.7675	0.762	0.041	41.6631	4.502797	0
1256.2936	0.655	0.151	16.7931	22.655540	0
1228.8615	0.632	0.300	46.8718	57.074566	0
1163.1614	0.614	0.132	30.2225	19.933493	0
1147.3957	0.662	0.035	26.6655	5.121392	0
1114.0817	0.492	0.510	116.9856	99.716240	0
1071.9560	0.571	0.169	9.3064	32.448460	0
1000.2844	0.885	0.022	192.2440	2.605477	0
938.1396	0.793	0.157	552.0563	20.294580	0
905.8951	0.785	0.212	18.4600	41.104351	0
877.9305	0.916	0.058	204.1968	8.152616	0
852.8913	0.909	0.069	12.0587	10.204072	0
819.9800	0.649	0.046	20.3796	4.504505	0
811.1745	0.612	0.388	25.0767	76.185356	0
790.0726	0.676	0.166	18.9076	26.104761	0
779.8343	0.683	0.092	19.0338	9.346799	0
752.7343	0.772	0.156	10.9867	27.055855	0
727.2372	0.811	0.097	9.3784	18.662539	0
695.2019	0.628	0.322	32.6104	60.930317	0
683.6349	0.642	0.061	5.9123	10.721626	0
650.1512	0.695	0.180	9.4772	33.931736	0
626.0752	0.831	0.026	118.9658	2.364295	0
593.9441	0.671	0.250	9.8828	48.070156	0
527.9558	0.763	0.202	13.3597	38.203686	0
499.4099	0.791	0.136	10.0819	24.801802	0
[illegible]	[illegible]	[illegible]	[illegible]		

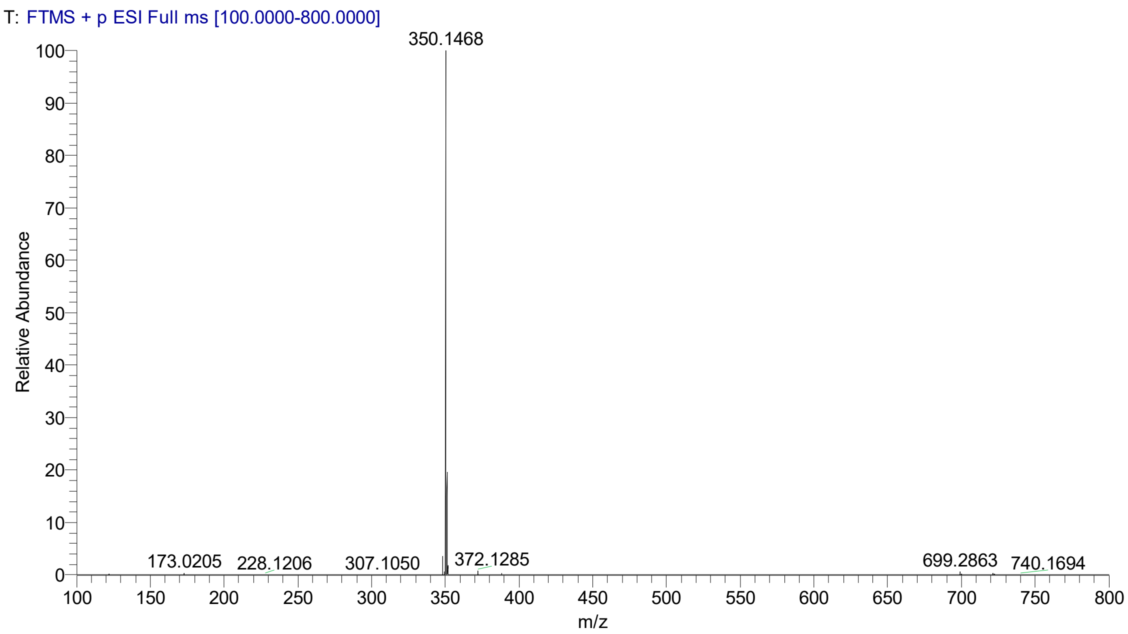
T: FTMS + p ESI Full ms [100.0000-800.0000]
350.1468
173.0205
228.1206
307.1050
372.1285
699.2863
740.1694
Relative Abundance
m/z

Figure S26. FTIR and HRMS spectra, 1H NMR at 400 MHz and 13C NMR at 100 MHz spectra for compound **67** (DMSO-d6)

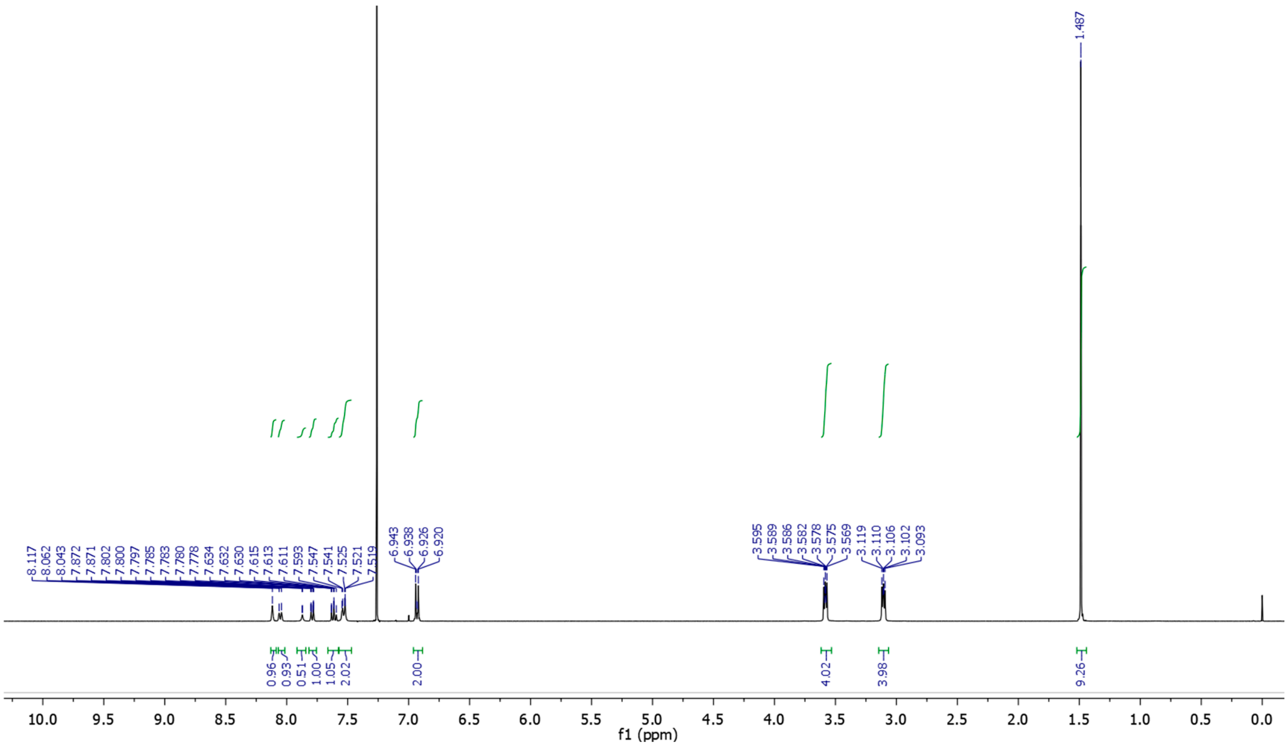

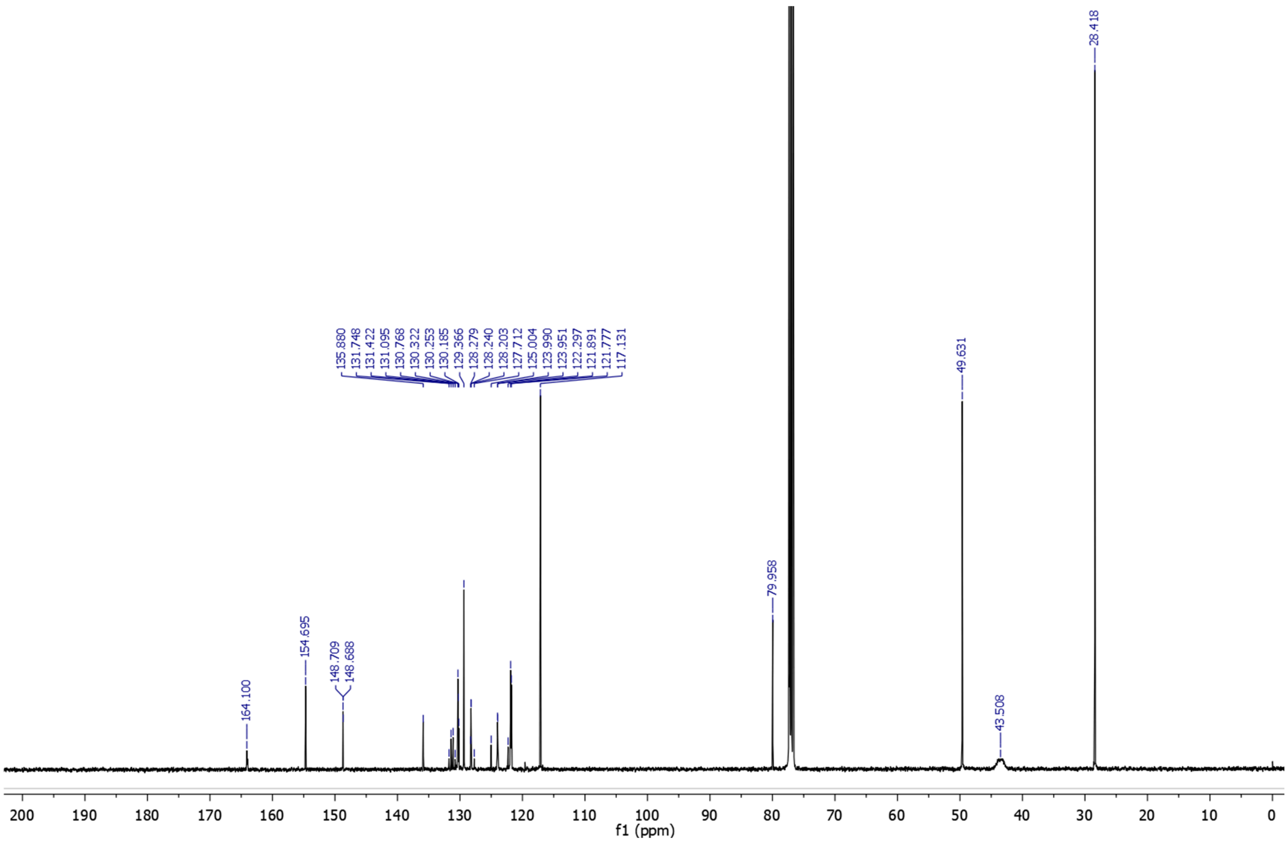

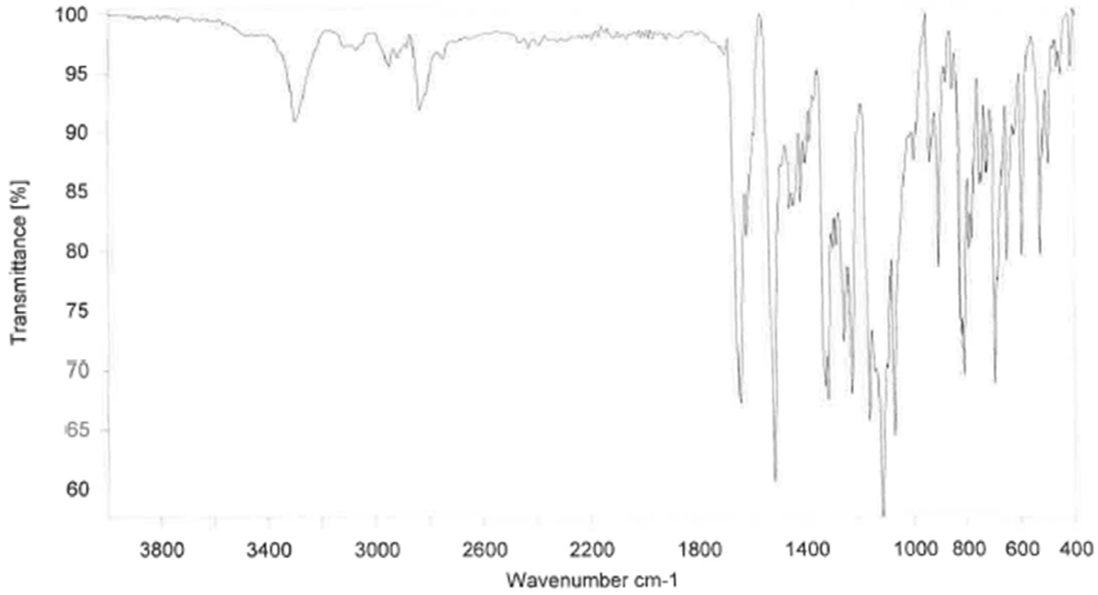

Wavelength	Absolute Intensity	Relative Intensity	Width	if Line < Shoulder	
3297.8256	0.910	0.087	79.7145	18.873699	0
3069.0359	0.970	0.016	83.1088	3.443493	0
2950.5250	0.956	0.028	34.5027	6.420379	0
2835.5331	0.919	0.069	54.1484	15.745001	0
2428.5929	0.971	0.016	65.7057	3.472650	0
1697.9034	0.965	0.014	26.8320	3.305395	0
1643.4476	0.672	0.328	36.7811	76.629173	0
1615.3609	0.812	0.058	118.5947	8.531566	0
1514.4360	0.606	0.383	29.4324	80.965813	0
1455.9050	0.835	0.064	28.4057	12.858622	0
1442.9868	0.838	0.019	22.6499	2.737810	0
1415.8286	0.840	0.066	8.2886	13.963799	0
1400.4376	0.873	0.027	234.8937	4.579582	0
1383.7251	0.891	0.033	7.7729	6.352624	0
1328.0058	0.686	0.071	33.8856	6.193793	0
1315.2731	0.675	0.271	35.1000	58.377949	0
1297.2936	0.802	0.018	5.7465	3.300424	0
1285.9254	0.804	0.026	6.2838	5.674622	0
1256.0325	0.724	0.081	15.3654	14.746900	0
1229.3335	0.680	0.206	125.6611	35.713646	0
1164.0068	0.658	0.088	11.1102	17.927284	0
1115.8872	0.577	0.425	92.0092	98.899254	0
1071.2066	0.644	0.152	10.2044	34.814892	0
996.2340	0.876	0.042	155.8277	5.670348	0
938.0649	0.874	0.088	103.6589	9.919373	0
904.8918	0.786	0.206	17.0579	46.512470	0
878.5457	0.941	0.023	173.9726	3.357582	0
853.2323	0.936	0.039	10.3236	7.151153	0
820.7056	0.729	0.022	16.1182	3.561569	0
811.4107	0.696	0.264	23.8694	58.491737	0
790.1850	0.802	0.079	157.0816	13.891254	0
779.9384	0.809	0.046	73.2455	5.363392	0
752.2377	0.856	0.081	17.4825	15.751420	0
744.5253	0.857	0.026	16.7556	2.282968	0
726.6638	0.866	0.056	10.7402	12.609242	0
695.3261	0.688	0.315	26.1873	72.936180	0
650.1792	0.792	0.135	9.3449	30.871317	0
594.4905	0.795	0.159	10.4580	36.286449	0
528.4859	0.797	0.189	15.4224	43.022892	0
499.3528	0.873	0.070	9.4769	14.904325	0

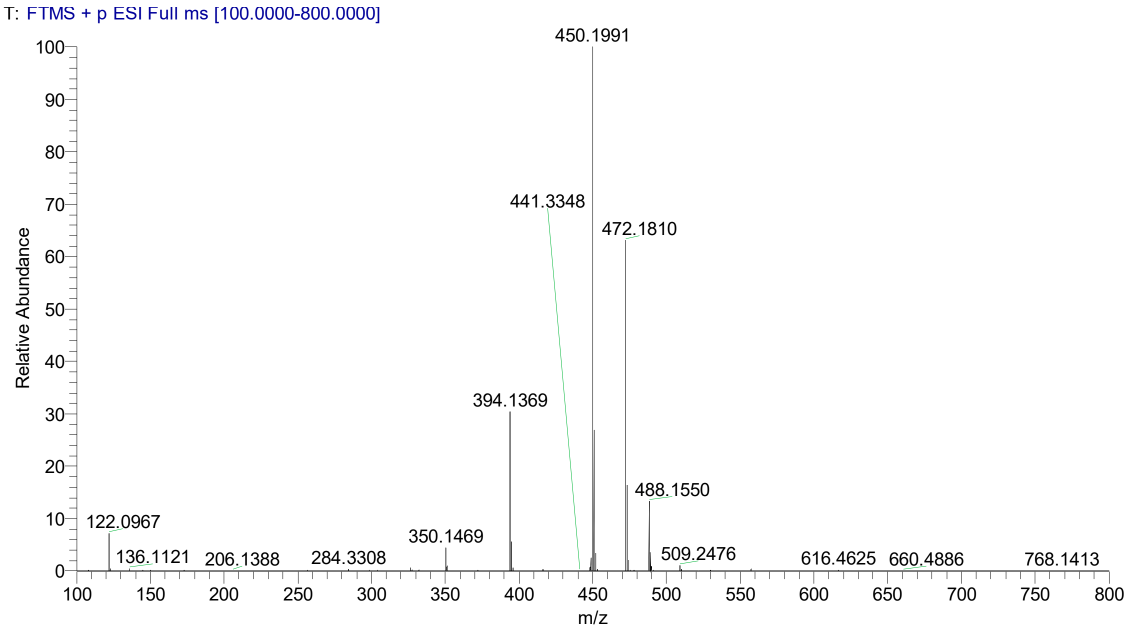
T: FTMS + p ESI Full ms [100.0000-800.0000]
450.1991
441.3348
472.1810
394.1369
488.1550
122.0967
136.1121
206.1388
284.3308
350.1469
509.2476
616.4625
660.4886
768.1413
Relative Abundance
m/z
